# Effects of Hydroxylated Mephedrone Metabolites on Monoamine Transporter Activity *in vitro*

**DOI:** 10.3389/fphar.2021.654061

**Published:** 2021-04-09

**Authors:** Marco Niello, Daniela Cintulová, Philip Raithmayr, Marion Holy, Kathrin Jäntsch, Claire Colas, Gerhard F. Ecker, Harald H. Sitte, Marko D. Mihovilovic

**Affiliations:** ^1^ Institute of Pharmacology, Medical University, Vienna, Austria; ^2^ Institute of Applied Synthetic Chemistry, TU Wien, Vienna, Austria; ^3^ Department of Pharmaceutical Chemistry, University of Vienna, Vienna, Austria

**Keywords:** mephedrone, cathinones, metabolism, psychostimulants, monoamines, transporters, addiction, enantiomers

## Abstract

Mephedrone is a largely abused psychostimulant. It elicits the release of monoamines via the high affinity transporters for dopamine (DAT), norepinephrine (NET) and serotonin (SERT). Stereoselective metabolic reactions are involved in the inactivation and the elimination of its chemical structure. However, during these processes, several structures are generated and some of them have been reported to be still pharmacologically active. In this study 1) we have newly synthetized several putative mephedrone metabolites, 2) compared their activity at monoamine transporters, 3) generated quantitative structure activity relationships, and 4) exploited the chemical structure of the putative metabolites to screen a urine sample from a drug user and dissect mephedrone metabolism. We have found that most of the tested metabolites are weak inhibitors of monoamine transporters and that all of them are more potent at DAT and NET in comparison to SERT. The only exception is represented by the COOH-metabolite which shows no pharmacological activity at all three monoamine transporters. The enantioselectivity of mephedrone and its metabolites is present mainly at SERT, with only minor effects at DAT and NET being introduced when the β-keto group is reduced to an OH-group. Importantly, while at DAT the putative metabolites did not show changes in inhibitory potencies, but rather changes in their substrate/blocker profile, at SERT they showed mainly changes in inhibitory potencies. Molecular modeling suggests that the hydrophobic nature of a specific SERT subpocket may be involved in such loss of affinity. Finally, the assessment of the putative metabolites in one urine sample of mephedrone user displayed two previously uncharacterized metabolites, 4-COOH-nor-mephedrone (4-COOH-MC) and dihydro-4- nor-mephedrone (dihydro-4-MC). These results confirm and expand previous studies highlighting the importance of the stereochemistry in the pharmacodynamics of phase-1 metabolites of mephedrone, established their structure-activity relationships at DAT, NET and SERT and pave the way for a systematic dissection of mephedrone metabolic routes. Given the number of structures found having residual and modified pharmacological profiles, these findings may help in understanding the complex subjective effects of administered mephedrone. Moreover, the dissection of mephedrone metabolic routes may help in developing new therapies for treating psychostimulants acute intoxications.

## Introduction

Mephedrone (MEPH) is a drug of abuse belonging to the family of synthetic cathinones. Similarly to other synthetic cathinones, such as methcathinone and 3,4-methylenedioxypyrovalerone (MDPV), it possesses stimulant properties (Marusich et al., 2012; Baumann et al., 2018). The chemical structure of MEPH has been reported as early as 1929, but first reports on its recreational use appeared only in 2007 as emerging member of the family of new psychoactive substances (NPS; Kelly 2011; Pedersen et al., 2013). NPS, also known as designer drugs, are typically synthetic in nature and easily accessible alternatives to well-established and scheduled drugs. Because they do not appear on the list of illicit drugs, NPS can be regularly sold online or in the street if labeled as ‘not for human consumption’ (Baumann and Volkow 2016; Baumann et al., 2018; Luethi and Liechti, 2020). If some NPS disappear right after being found and banned, others become established within the drug market contributing to the addiction plague (United Nations Office, 2016). Despite being banned in most of the western countries around 2010 (Green et al., 2014), MEPH became an established substance of abuse in some regions (Hockenhull et al., 2016; Ordak et al., 2018).

Drug users have reported that MEPH shows entactogenic and subjective effects similar to those elicited by 3,4-methylendioxymethylamphetamine (MDMA, “ecstasy”; Schifano et al., 2011). Mephedrone interacts mainly with the dopamine, norepinephrine and serotonin transporters (DAT, NET and SERT, respectively; Mayer et al., 2016b; Mayer et al., 2019). DAT, NET and SERT are membrane proteins involved in the re-uptake of the respective neurotransmitters back into the pre-synaptic terminal after quantal release (Kristensen et al., 2011), limiting neurotransmitter diffusion and regulating the neurotransmitter binding to the receptors (Cragg and Rice 2004). Given their important role in brain signaling, they are target of several pharmacological agents, some clinically in use (e.g. antidepressants) and some misused (e.g. psychostimulants) (Kristensen et al., 2011; Niello et al., 2020). These drugs can interact either as blocker, i.e. they bind and they block the transport process, or as releasers, i.e. they are re-uptaken by the transporter and once into the intracellular space they revert its function through a series of complex events (Sitte and Freissmuth 2015).

Synthetic cathinones are characterized by the presence of a chiral center and exist therefore in two different stereoisomers, which are often characterized by different pharmacological properties. In the case of mephedrone, stereospecific effects have been reported both *in vitro* and *in vivo*. While both the R- and S-enantiomers of mephedrone are releasers at DAT and NET with similar potency, S-mephedrone is a releaser of SERT with 40 times higher potency than R-mephedrone (Gregg et al., 2015; Mayer et al., 2016b; Mayer et al., 2019). Moreover, only R-mephedrone resulted in conditioned place preference and produced greater intracranial self-stimulation and sensitization to repetitive movements (Gregg et al., 2015). Consistently, in rats, R-mephedrone was more easily self-administered than S-mephedrone (Philogene-Khalid et al., 2017).

Mephedrone has been shown to be metabolized through three main hepatic mechanisms involving the cytochrome P450 2D6: 1) N-demethylation, 2) hydroxylation, 3) reduction of the β–keto-group (Meyer et al., 2010; Pedersen et al., 2013). We have recently shown that some phase I metabolites of mephedrone are pharmacologically active at monoamine transporters and may be in part responsible of its entactogenic and psychostimulant effects. Systemic administration of nor-mephedrone, the N-demethylated metabolite of mephedrone, results in increased extracellular serotonin but in a blunted increase of extracellular dopamine in rat nucleus accumbens (Mayer et al., 2016a). Given the recent finding that mephedrone undergoes stereospecific metabolism (Castrignanò et al., 2017), we have then tested the enantiomers of specific mephedrone metabolites to investigate the stereoselective pharmacology of mephedrone. We have found that the S-enantiomers of both nor-mephedrone and 4-OH-mephedrone were far more potent than the corresponding R-enantiomers and that the R-enantiomers were also pronouncedly less effective as SERT-releasers (Mayer et al., 2019).

Following up on these studies, we have hypothesized that through the complex metabolism of mephedrone, other metabolites may participate in this effect and could be used for quantitative structure-activity relationships at human monoamine transporters. Therefore, in the current study 1) we have synthesized other potential mephedrone metabolites in an enantiomeric pure form and 2) investigated their stereo-pharmacology at DAT, NET and SERT using uptake-inhibition and transporter electrophysiology in HEK293 cells; 3) we provide quantitative structure-activity relationships at SERT and 4) we have analyzed human urine samples for the presence of the investigated stereoisomers.

## Materials and Methods

### Cell Culture

Human embryonic kidney 293 cells (HEK293 cells) were cultivated in Dulbecco’s Modified Eagle Medium (DMEM), containing 10% heat-in- activated fetal calf serum (FCS), streptomycin (100 μg x 100 ml−1) and penicillin (100 U × 100 ml−1). Geneticin (50 μg x mL−1) was added to select HEK293 cells stably expressing the human isoforms of DAT, NET and SERT as described earlier (Mayer et al., 2016a). Cell lines were maintained in a sub-confluent state in humidified atmosphere (37°C, 5% CO_2_). For uptake inhibition experiments cells expressing the desired transporter were seeded the days before the experiment (40,000 cells per well) or two days before the experiments (20,000 cells per well) onto poly-D-lysine (PDL) coated 96-well plates 24 h prior to the experiment. In the case of transporter-electrophysiology cells were cultivated in 10 cm dishes and 24 h before the experiments they were detached, and seeded at low density onto PDL-coated 3 cm dishes.

### Uptake-Inhibition Experiments

The experiments have been conducted as described previously (Maier et al., 2018). In brief, the day of experiment the cell culture medium was replaced with Krebs-HEPES buffer (KHB: 120 mM NaCl, 25 mM HEPES, 5mM KCl, 5 mM D-glucose, 1.2 mM CaCl2, and 1.2 mM MgSO4 and, pH adjusted to 7.3). After 10 min, the cells were incubated for 6 min with increasing concentrations of the substance of interest. Then, tritiated substrates (20 nM [^3^H]MPP + for DAT and NET and 100 nM [^3^H] 5-HT for SERT) were added. After 3 min (DAT and NET) or 1 min (SERT), the tritiated substrates were aspirated and the cells were washed with 300 µL of ice-cold KHB and lyzzed with 200 µL of 1% sodium dodecyl sulfate (SDS). The radioactivity was determined with a beta-scintillation counter. Nonspecific uptake was determined in the presence of 30 μM cocaine (DAT, NET) or 10 μM paroxetine (SERT) and subtracted. Uptake in the absence of the substance of interest was defined as 100% uptake. The IC50 values were determined by non-linear regression fits (GraphPad Prism version 5.0; log(inhibitor) vs. response (Y= Bottom + (Top-Bottom)/(1 + 10ˆ (X-LogIC50));

### Transporter Electrophysiology

Transporter electrophysiology was conducted by the means of whole cell patch clamp in HEK293 cells stably expressing DAT or SERT. Substrate-induced currents were recorded under voltage clamp (−60 mV). Cells were continuously superfused with a physiological external solution that contains 140 mM NaCl, 2.5 mM CaCl2, 2 mM MgCl2, 20 mM glucose and 10 mM HEPES, pH = 7.4. The pipette solution contained 133 mM potassium gluconate, 6 mM NaCl, 1 mM CaCl2, 0.7 mM MgCl2, 10 mM HEPES, 10 mM EGTA, pH = 7.2. Currents elicited by mephedrone 30 μM, were measured at room temperature (20–24°C) using an Axopatch 700B amplifier and pClamp 11.2 software (MDS Analytical Technologies). All the solutions perfused onto the cell were applied using a DAD-12 superfusion system and a 8-tube perfusion manifold (ALA Scientific Instruments), which allowed for rapid solution exchange. Current traces were filtered at 1 kHz and digitized at 10 kHz using a Digidata 1,550 (MDS Analytical Technologies). Current amplitudes in response to substrate application were quantified using Clampfit 10.2 software (Molecular Devices). For the analysis, passive holding currents were subtracted, and the traces were filtered using a 100-Hz digital Gaussian low-pass filter.

### Quantitative Structure-Activity Relationships

Due to the structure of the data set and the fact that it is composed of stereoisomers, we decided to perform a Free-Wilson Analysis. Thus, every structural variation is encoded with an indicator variable rather than physicochemical properties. Based on MEPH, the structural variations are 1) N-Me or else, 2) 4-OH or else, 3) C=O or else. Stereochemistry is encoded with two additional variables (1R-COH or else and 2R-Me or else). This leads to a data set of 24 compounds described by 5 descriptors. This data set was subject to multiple linear regression analysis performed in MS Excel for Mac using the analysis tool. In a backward selection process the analysis started with all 5 descriptors and the least significant one based on the *p*-value was eliminated from the equation till only descriptors with a *p*-value < 0.05 remained in the equation. This led to the following equations: hSERT: pIC_50_ = 4.28 + 0.62 C=O – 0.62.2R-Me – 0.92.4-OH, *n* = 24, *r* ^2^ = 0.80; hDAT: pIC_50_ = 4.81 + 0.81 C=O – 0.62.4-OH, *n* = 24, *r* ^2^ = 0.71; hNET: pIC_50_ = 4.58 + 0.82 C=O – 0.35.4-OH, *n* = 24, *r* ^2^ = 0.74; Mephedrone analogs at hSERT: pIC_50_ = 5.01–1.16.2R-Me – 0.62.4-OH, *n* = 8, *r* ^2^ = 0.97. As can be seen, in all three transporters the both reduction of the carbonyl group and the hydroxylation of the 4-methyl group leads to a decrease of activity. With the exception of hSERT, there are no significant trends with respect to the absolute configuration of the stereocenters. In case of hSERT, the (2R)-methyl analogs are less active than their corresponding (2S9-stereoisomers. However, it should be noted that the overall variance of IC_50_ values in the data set is only close to two orders of magnitude, which is the absolute minimum required for QSAR analyses. Thus, the equations should be seen as trends rather than mathematical correlations.

### Docking Studies

We conducted docking experiments of the studied compounds in an 5HT-bound occluded conformation of the serotonin transporter built in a former study (Hellsberg et al., 2018). This conformation was chosen as it is more likely to accommodate small molecule ligands. We used the QM-Polarized ligand docking (QPLD) implemented in the Schrödinger suite (QM-Polarized Ligand Docking protocol; Glide, Schrödinger, LLC, New York, NY, United States, 2020; Jaguar). First, the ligands were prepared with the LigPrep tool from Schrödinger using the default parameters, except for the pH set a 7 +- 0.5 (Schrödinger release 2020–4: LigPrep, Schrödinger, LLC, New York, NY, United States, 2020.). The receptor was prepared with the Protein Preparation Wizard implemented in Maestro (Schrödinger release 2020–4: Maestro, Schrödinger, LLC, New York, NY, United States, 2020.). The docking grid of the receptor was generated with Glide with the box enclosing the coordinates of the bound 5-HT. After the initial Glide docking, performed in Extra Precision mode, the partial charges of the docked ligands were calculated with Jaguar. Finally, the ligands with the new charges were redocked in Extra Precision. For the analysis, the poses were clustered using the interaction fingerprint calculation panel of Schrödinger. The stereoisomers were clustered separately. The 86 poses of the two 4-OH-MC enantiomers were clustered in 13 groups, while the 83 poses of the dihydro-4-OH-MMC, dihydro-4-MC and dihydro-4-OH-MC diastereoisomers were clustered in 11 groups.

### Profiling of Mephedrone Enantiomers in Human Urine Samples

The biological specimen, human urine, was provided by the Department of Chemical Analytics, Seibersdorf Laboratories GmbH. The sample was taken from a subject postmortem in a fatal case of drug overdose. Routine forensic analysis of urine revealed that among other drugs, large amounts of mephedrone were present in the system of the subject. Given this fact, we assumed that also the metabolites would be detectable in sufficient quantity. To evaluate the presence of any metabolites whatsoever in the urine sample we designed an achiral separation protocol for liquid chromatography and we coupled it with triple quadrupole mass spectrometer. The advantage of LC-MS/MS system is that even if two substances have the same retention time, they can still be differentiated by their fragmentation pattern. More in details, the samples for urine analysis were analyzed using a CTC HTS PAL autosampler (CTC Analytics) and a Thermo Surveyor LC system (Thermo) interfaced to a TSQ Quantum Discovery Max triple quadrupole mass spectrometer (Thermo). A C18 security guard cartridge (4 × 2 mm) (Phenomenex) was used for sample clean-up and the analytical HPLC column was a Kinetex 1.7 μm C18 100Å 150 × 2.1 mm (Phenomenex) and Cortecs T3, 2.7 μm C18 120Å 100 × 2.1 mm (Waters). Column selection was performed by a Maylab Mistraswitch column selector (Maylab Analytical Instruments). The solvents were 0.2% formic acid in water (A) and acetonitrile (B). The temperature of the analytical column was maintained at 40°C. The mass spectrometer was equipped with heated electrospray ionization (ESI) source and was operated in positive ionization mode with a spray voltage set at 4500V. The capillary temperature was adjusted to 320°C. The sheath and auxiliary gas (nitrogen) flow rate was 25 and 10 arbitrary units, respectively. The system was operated in selected ion monitoring (srm) mode with argon as the collision gas at a pressure of 1.5 mTorr. For the achiral separation, stem solutions were prepared by weighing the reference substances in volumetric flasks and filling them up with MeOH (99.9%; HPLC grade), creating average concentration of 1 mg/ml. Then dilution solutions were prepared as follows: 10 μL of stem solution was filled up to 5 ml MeOH, creating dilution 1:1,000. Samples were prepared from dilution solutions by taking out 25 μL and mixing it together with 25 μL of ISP, 50 μL ISS and filled up to 1 ml with Milli-Q water. The final sample solvent was water/MeOH 9/1. For the enzymatic workup, phosphate buffer (1 ml, 0.8M, pH = 7), β-glucuronidase/arylsulfatase (25 μL) and β-glucuronidase (25 μL) were added to the solutions. The solutions were heated at 50°C during 1 h upon which EtOAc (7 ml) was added. The solution was put into shaker for 10min, following centrifugation for 5 min. The organic phase was separated and evaporated into dryness in stream of pressurized nitrogen. The residue was taken up in 150 μL MQ-water/MeOH 9/1 + 0.4% formic acid, heated at 50°C for 5 min and subjected to LC-MS analysis.

## Results

### Stereoisomers of Phase-I Metabolites Directly Interact With Monoamine Transporters

**FIGURE 1:**
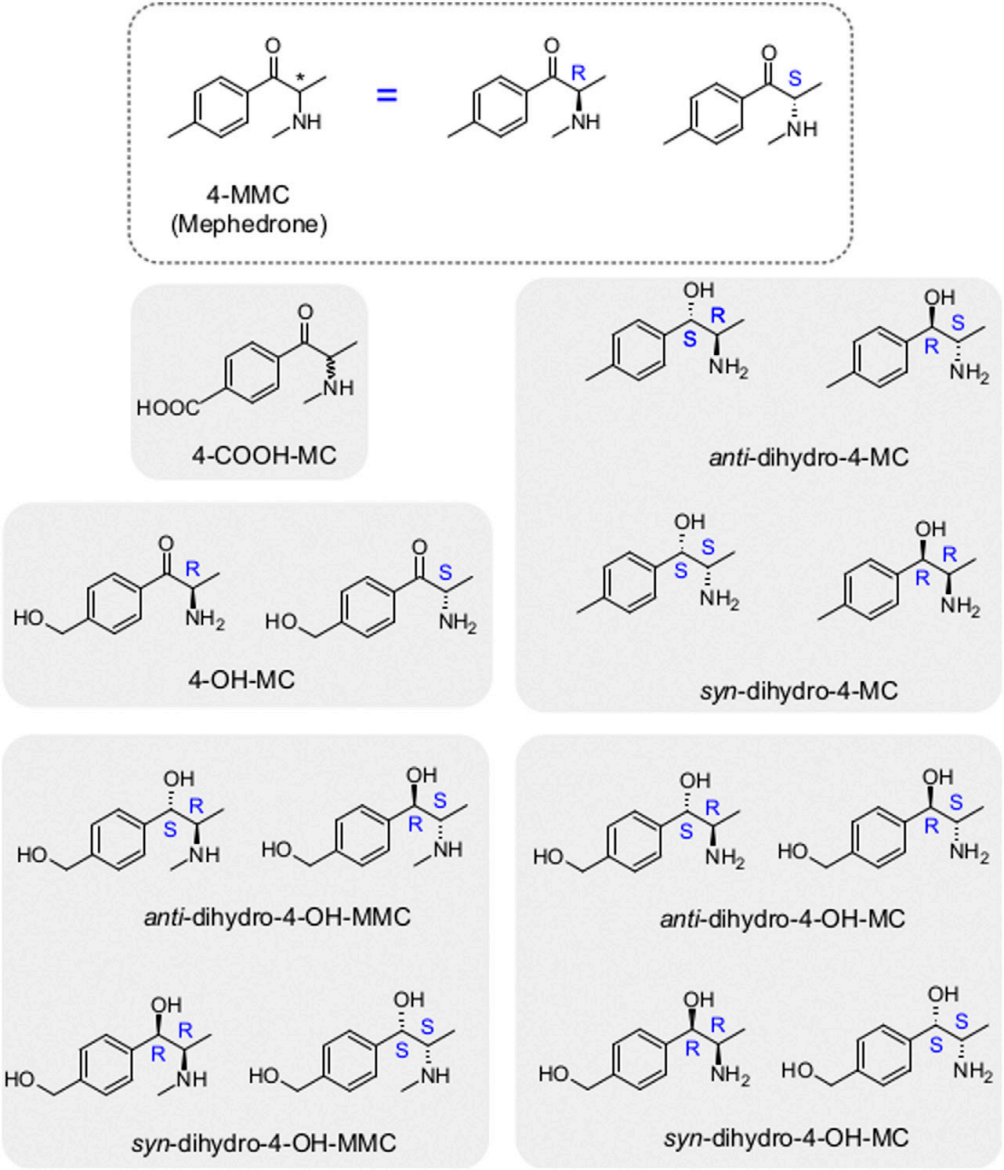
Mephedrone metabolites analyzed in the current study. On top is highlighted mephedrone structure with its stereocenter (asterisk) which leads to the two enantiomers (R- and S-mephedrone). The metabolites analyzed in the current study are highlighted by the grey rectangles in the background with the enantiomers of the same metabolite being grouped within the same rectangle. Note that in the case of the 4-COOH-MC only the racemate was tested.

**FIGURE 2:**
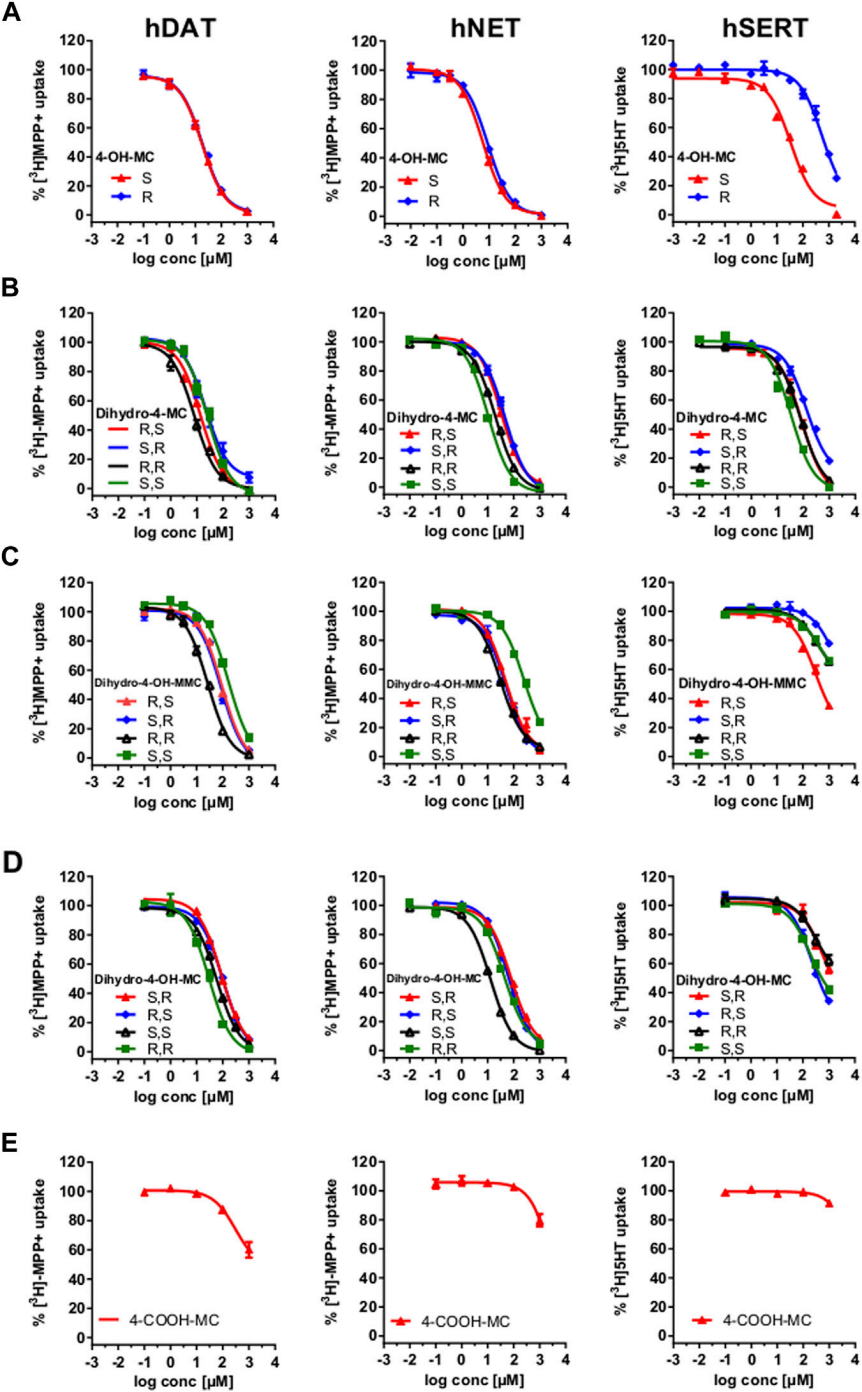
Uptake-Inhibition of the synthesized enantiomers at hDAT **(left)**, hNET **(middle)** and hSERT **(right)**. Uptake of [^3^H]MPP+ (DAT and NET expressing cells) and [^3^H]5-HT (SERT expressing cells) was inhibited with increasing concentrations of the indicated test drugs. Data are shown as the mean ± SEM obtained from 3-5 individual experiments, performed in triplicate. **(A)** uptake-inhibition of 4-OH-MC enantiomers; **(B)** uptake-inhibition of dihydro-4 -MC enantiomers; **(C)** uptake-inhibition of Dihydro-4-OH-MMC enantiomers; **(D)** uptake-inhibition of Dihydro-4-OH-MC enantiomers; uptake-inhibition of the racemic 4-COOH-MC.

Phase I metabolism of MEPH occurs through different routes which involve 1) demethylation, 2) benzylic oxidation and 3) carbonyl reduction. We have synthesized the different metabolites that can possibly derive from MEPH phase I metabolism (see Supplementary material for the chemical synthesis and Figure 1 for the chemical structures) and tested them in HEK293 cells stably expressing the human DAT, NET or SERT. Uptake-inhibition experiments showed that MEPH metabolites display different activities at DAT, NET and SERT (Figure 2 and Table 1). With the only exception of S-4-OH-MC, R-4-OH-MC and dihydro-(S,S)-4-MC at NET and dihydro-(R,R)-4-MC at DAT, the tested metabolites showed a reduced potency in inhibiting monoamine transporters compared to mephedrone (Figure 2 and Table 1). Despite of their general weak inhibitory potency, all the tested metabolites are more potent on DAT/NET in comparison to SERT, with the dihydro-(R,R)-4-MC showing inhibitory activity at DAT/NET in the range of the parent drug (IC50 = 1 to 10 µM). In addition, while the S- and the R-4-OH-MC did not show any effect of enantioselectivity at DAT and NET, they showed a pronounced enantiodifferentiation at SERT, even if at concentrations that may not be pharmacologically relevant (S-4-OH-MC IC50 = 40 µM (34.01–47.12); R-4-OH-MC IC50 = 678 µM (583–788)). Moreover, the reduction of the β-carbonyl group to an OH-group, introduces minor enantioselective features at DAT and NET too. Importantly, the introduction of the COOH-moiety abolished the activity at all the transporters. In this case, the stereochemistry was not tested.

**TABLE 1:** Effect of tested mephedrone metabolites on monoamine transporter mediated uptake. IC_50_ values are represented in μM as the mean (95% confidence interval) obtained from non-linear regression. NET/DAT ratio = (DAT IC_50_)/(NET IC_50_). Values > 1 indicate greater NET selectivity. DAT/SERT ratio = (SERT IC_50_)/(DAT IC_50_). Values > 1 indicate greater DAT selectivity. In the right column is highlighted if the metabolites were detected (+), not detected (−) or not determined (n.d.) in the urine samples analyzed. Only *syn-* and *anti-*configurations could be separated.

Compound	IC_50_ (µM)			Selectivity ratio		Detected in urine samples
	DAT	NET	SERT	NET/DAT	DAT/SERT	
Cocaine^†^ Ilic et al. (2020)	1.21 ± 0.8^†^ (0.72–2.01)	1.03^†^ (0.53–2.00)	8.7^†^ (7.44–12.26)	1.2	7.25	n.d
D-Amphetamine^†^ Ilic et al. (2020)	3.60^†^ (2.13–6.16)	0.85^†^ (0.56–1.08)	151.4^†^ (101.5–225.9)	4	42.06	n.d
Mephedrone^#^ Niello et al. (2019) ^§^ Mayer et al. (2016b)	3.01^#^ (2.34–3.67)	2.77^§^ (1.92–3.97)	8.74^#^ (6.74–10.73)	1.09	2.90	**+**
R-4-OH-MC	19.83 (17.16–22.93)	9.07 (7.69–10.69)	678.1 (583.4–788.1)	2.19	34.19	−
S-4-OH-MC	19.23 (16.57–22.32)	5.97 (5.23–6.83)	40.03 (34.01–47.12)	3.22	2.08	
Dihydro-(R,S)-4-MC	13.51 (11.72–15.58)	32.35 (28.13–37.20)	66.86 (53.64–83.35)	0.42	4.94	**+**
Dihydro-(S,R)-4-MC	15.46 (13.71–17.43)	41.80 (36.54–47.81)	178.3 (157.8–201.3)	0.37	11.53	
Dihydro-(R,R)-4-MC	8.05 (6.39–10.23)	18.10 (16.29–20.11)	74.58 (62.52–88.82)	0.44	9.26	**+**
Dihydro-(S,S)-4-MC	22.63 (18.45–27.75)	9.51 (8.44–10.71)	32.24 (25.98–38.53)	2.37	1.42	
Dihydro-(R,S)-4-OH-MC	99.14 (89.24–110.1)	61.98 (55.87–68.75)	383.4 (315.2–466.5)	1.60	3.86	−
Dihydro-(S,R)-4-OH-MC	91.23 (83.66–99.46)	83.39 (74.93–92.80)	1,157 (875.7–1,530)	1.09	12.68	
Dihydro-(R,R)-4-OH-MC	28.29 (22.94–34.90)	46.50 (40.34–53.60)	1,370 (1,047–1794)	0.61	48.42	−
Dihydro-(S,S)-4-OH-MC	59.23 (52.27–67.12)	12.56 (11.57–13.64)	588 (499.9–691.7)	4.71	9.92	
Dihydro-(R,S)-4-OH-MMC	84.49 (70.42–101.4)	53.92 (46.07–63.11)	496.2 (422–582.1)	1.57	5.87	−
Dihydro-(S,R)-4-OH-MMC	77.03 (62.55–94.87)	49.85 (46.07–63.11)	3,158 (2,540–3,928)	1.55	40.99	
Dihydro-(R,R)-4-OH-MMC	25.62 (22.15–29.64)	36.95 (33.36–40.93)	1735 (1,423–2,144)	0.69	67.72	−
Dihydro-(S,S)-4-OH-MMC	160.8 (140.4–184.2)	290.6 (259.8–325.1)	1848 (1,518–2,250)	0.55	11.49	
(rac)-4-COOH-MC	1,466 (1,133–1897)	3,059 (2,177–4,298)	11,432 (6,590–19,833)	0.48	7.80	**+**

### Phase-I Metabolism Shapes the Affinity and the Substrate/Blocker Profile of Mephedrone Metabolites at DAT and SERT

**FIGURE 3:**
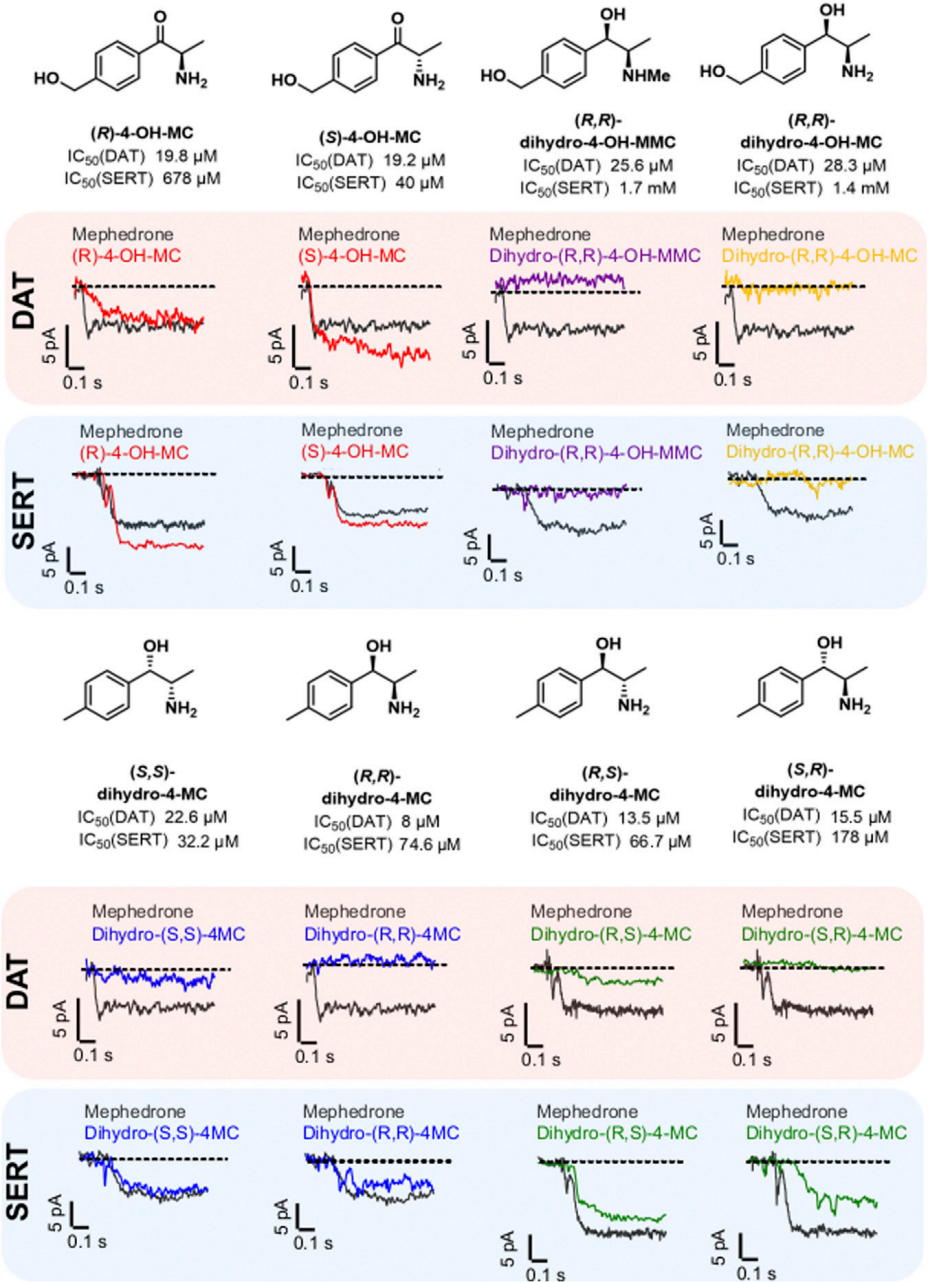
Chemical structure, IC50 values and representative traces of transporter-mediated currents for mephedrone and the selected metabolites. All the metabolites showing an IC50 < 30 µM in one of the two transporters were tested for their ability to elicit transporter-mediated currents. For each of the selected metabolites, representative traces for DAT and SERT are shown below the respective chemical structure. DAT-mediated currents are highlighted by a light-red background, while SERT-mediated currents are highlighted with a light-blue background. The different enantiomers of a specific metabolite are in the same color. All the compounds, including mephedrone, are applied at 30 µM.

**FIGURE 4:**
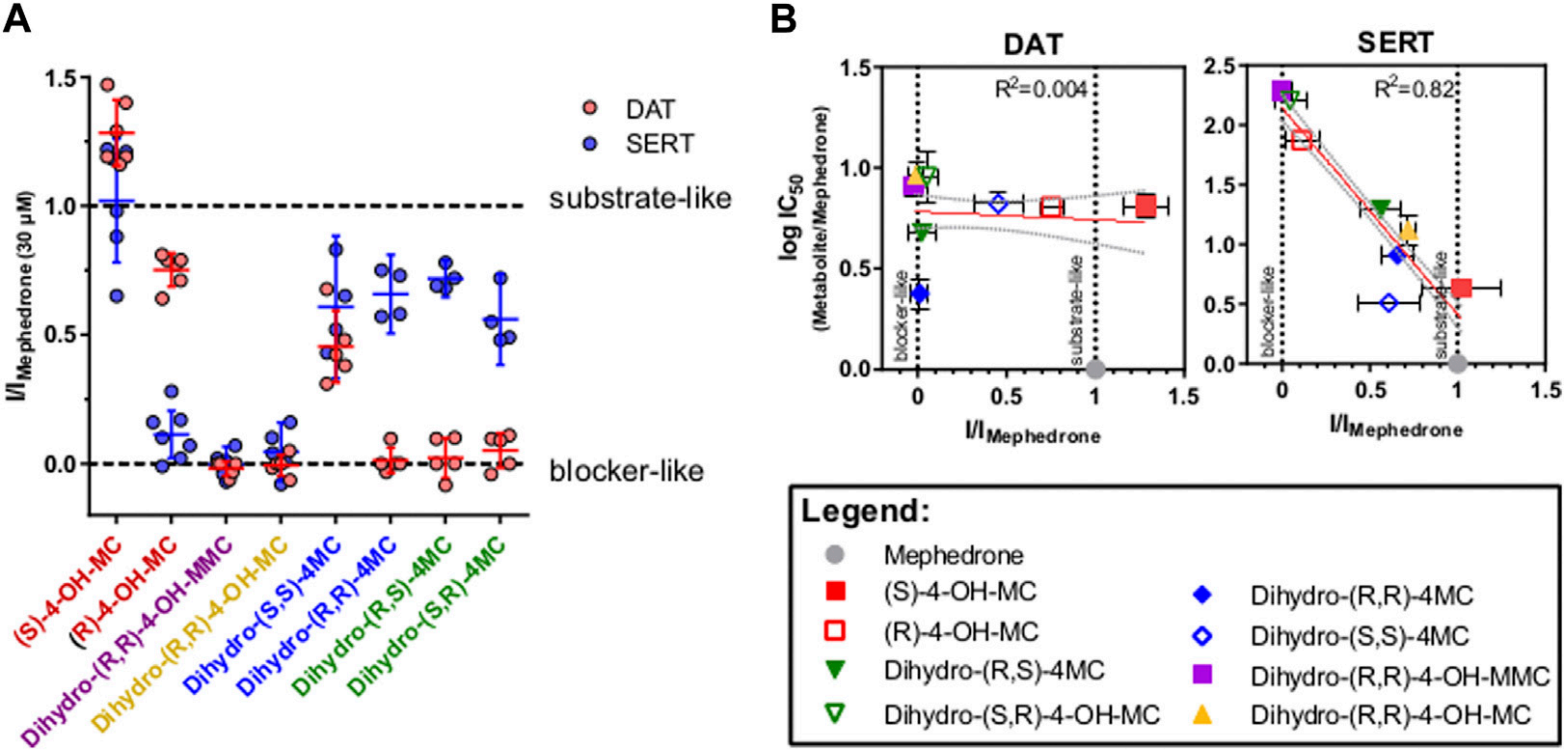
DAT- and SERT-mediated currents of selected metabolites. **(A)** normalized steady-state current amplitude as (I_metabolite 30µM_/I_Mephedrone 30µM_). Data are represented as mean and 95% confidence interval, with dots representing individual cells (red = DAT and blue = SERT). On the *x* axis, the two enantiomers of the same metabolite are depicted with the same color. **(B)** correlation analysis for changes in current profile and in IC50s. IC50 values of the different metabolites, obtained in the uptake-inhibition experiments, are normalized to the IC50 of mephedrone. The Log10 of this ratio is plotted against the current ratio and subjected to linear regression. The linear fit of the data is represented by the orange line with the grey dashed lines depicting the 95% confidence interval. For DAT **(middle)** there is no linear correlation (*R* ^2^ = 0.004, *F* = 0.299, *p* < 0.586). For SERT **(right)** the two measures are linearly correlating (*R* ^2^ = 0.82, *F* = 397.8, *p* < 0.0001).

Phase I metabolism can change mephedrone pharmacology by generating metabolites with different affinities for their targets or by generating metabolites with a different substrate/blocker profile. Transporter-mediated currents are small currents elicited by monoamine transporters in heterologous expression systems during transport process. Despite their existence *in vivo* has been a matter of debate (Gerstbrein and Sitte, 2006), they represent a practical tool for studying the transport cycle in heterologous expression systems because of the high time-resolution and the access to the intracellular compartment offered by the whole-cell patch-clamp technique. Under physiological conditions (i.e. 140 mM extracellular Na^+^ and 140 mM intracellular K^+^), transporter-mediated currents represents the transporter moving through the whole transport cycle (Hasenhuetl et al., 2019). As such, they can be used as information of the transport event without the need of synthesizing substances in a radioactive form. Given that 1) the DAT/SERT selectivity can be used as a rough indication of addictive properties of psychostimulants (Wee et al., 2005) and 2) metabolism may affect the substrate/blocker profile of MEPH, we have compared all the substances with an IC50 of less than 30 μM at DAT and/or SERT (Figure 3) for their ability to induce transporter-mediated currents. Every substance analyzed was applied to HEK293 cells stably expressing DAT or SERT at a concentration of 30 µM. Similarly to mephedrone, the application of S- and R-4-OH-MC elicited both DAT- and SERT mediated currents (Figure 3, red traces). Instead, dihydro-(S,S)-4MC, dihydro-(R,R)-4 MC (blue traces), dihydro-(R,S)-4-MC (green traces) elicited only SERT-mediated currents. dihydro-(R,R)-4-OH-MC and the dihydro-(R,R)-4-OH-MMC did not elicit any DAT or SERT-mediated current. To extrapolate the effect of metabolism on the substrate/blocker profile, the steady-state current of the resulting drugs was then normalized to the one of MEPH 30 µM to account for expression differences. Compounds showing a ratio close to 1 are transported with similar efficiency to MEPH, while those with a ratio close to 0 show impaired transport efficiency (Figure 4A). To account for reduced ratios due to the lack of affinity we have correlated the current ratio obtained through transporter electrophysiology with the IC50 obtained through the uptake-inhibition experiments normalized to the MEPH IC50 (metabolite IC50/MEPH IC50). As shown in Figure 4B, while different metabolic reactions do not elicit pronounced changes in the affinity at DAT, they do change the transport efficiency, resulting in a flat correlation between these two measures (*R* ^2^ = 0.004, *F* = 0.299, *p* < 0.586). On the other hand, at SERT, the effect on transport-mediated currents is linearly correlating with the change in affinity (*R* ^2^ = 0.82, *F* = 397.8, *p* < 0.0001). These data suggest that mephedrone effects may be elicited by a mixture of chemical species acting in a very heterogenous manner. Understanding which components are responsible for which effects will enable to dissect its complex pharmacology.

### Molecular Modeling Studies of Mephedrone Metabolites

Given the pronounced differences observed in our uptake-inhibition experiments, we have attempted to define structure-activity relationships of the mephedrone metabolites. As the data set is characterized by a few distinct structural modifications and in order to allow the inclusion of information on stereochemistry, we performed a Free-Wilson analysis (Kubinyi, 1976). In this type of QSAR analysis, all variations are encoded as indicator variables (for the complete data matrix see Supplementary Table S1). After backwards variable selection, we obtained reasonably significant QSAR equations for all three transporters, outlining identical trends: 1) reduction of the carbonyl group leads to decrease of activity, and 2) hydroxylation of the 4-methyl group also leads to e decrease of activity. With the exception of hSERT, information on stereochemistry did not consistently contributed to the variance in activity, thus the respective indicator variables did not remain in the final equation. In hSERT, the (2 S)-analogs are more active than their (2R)-stereoisomers.

**FIGURE 5:**
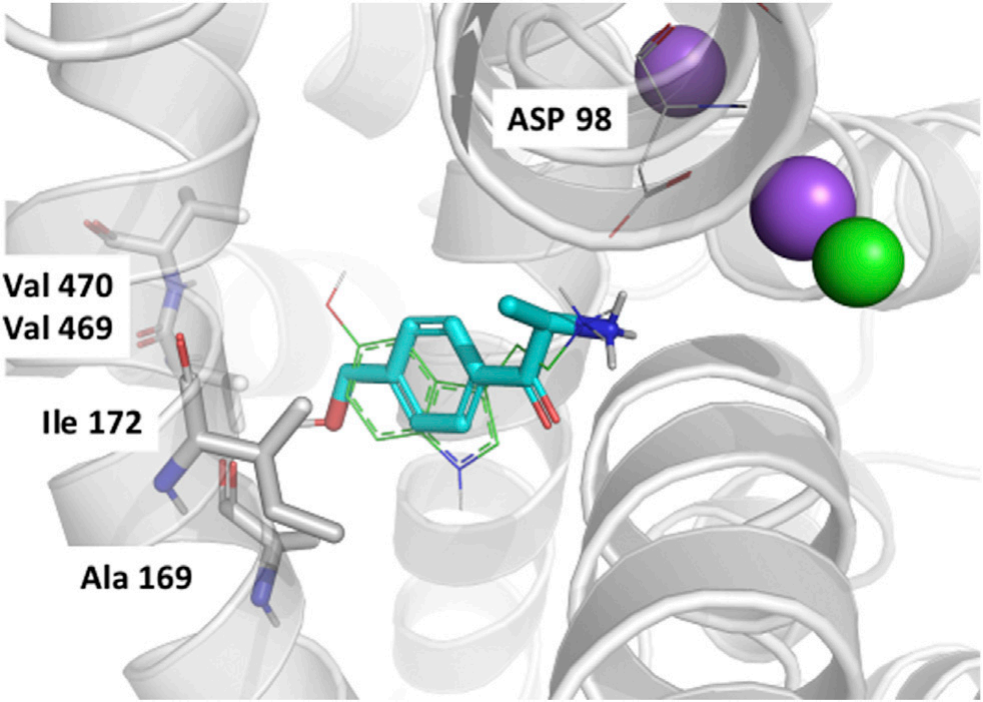
QSARs. Docking pose of R-4-OH-MC in the outward occluded model of SERT bound to 5HT (Hellsberg et al., 2018). The hSERT is represented in transparent gray cartoons, with the residues constituting subsite B shown in sticks, and labeled. The sodium and chloride ions are represented in purple and green spheres, respectively. The 5HT molecule bound to the SERT model is represented in green lines, while the docked R-4-OH-MC is shown in cyan sticks. The figure has been created with Pymol (Schrodinger, L. L. C. The PyMOL Molecular Graphics System, Version 2.2.).

In order to rationalize the results of the QSAR studies we performed docking studies of the mephedrone metabolites into a previously established homology model of hSERT in the 5HT-bound occluded conformation (Hellsberg et al., 2018). Unfortunately, the docking experiments were not able to explain the enantioselectivity differences observed in SERT. However, our poses help rationalizing why the 4-CH_2_OH analogs are considerably less active than the respective 4-CH_3_ derivatives. In fact, the hydroxy group points directly toward a hydrophobic subpocket (Figure 5). Subpocket B is mainly constituted of conserved hydrophobic residues, which could explain the decrease of activity of the hydroxy-mephedrone in the three monoamine transporters.

### Detection of MEPH Metabolites in Human Urine Samples

**FIGURE 6:**
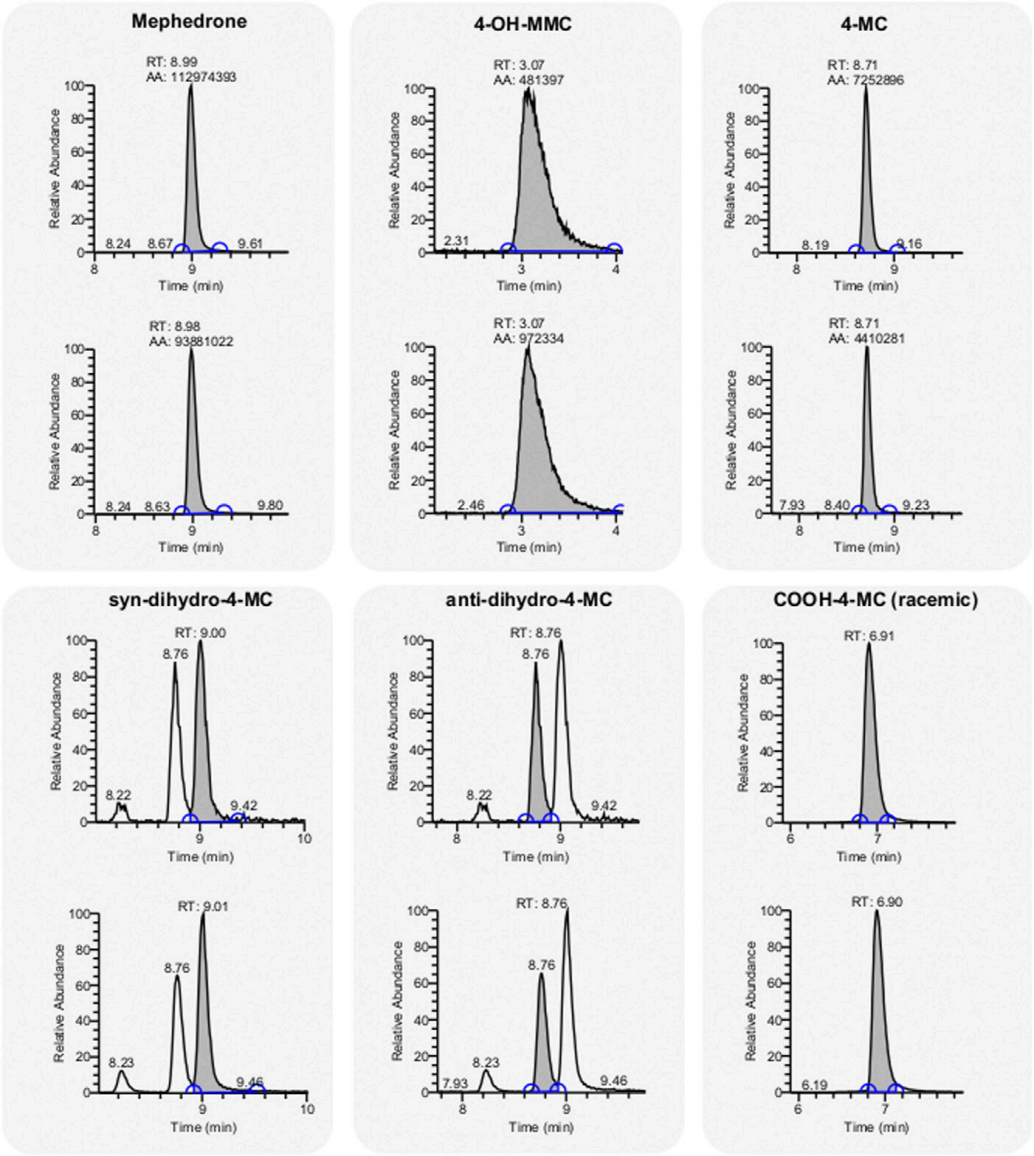
Detection of MEPH metabolites in a human urine sample. Chromatograms of Mephedrone and the detected metabolites (4-OH-MMC, 4-MC, 4-COOH-MC, dihydro-4-MC (anti and syn). The analysis was repeated two times obtaining similar results (grey background, top and bottom).

If on one hand the synthesis of putative metabolites allows the establishment of structure activity relationships, on the other hand, it provides pure material to evaluate their presence in human urine samples and determine their pharmacological relevance. To determine the presence of any metabolites in the urine sample, we have designed an achiral separation protocol and coupled the liquid chromatography with triple quadrupole mass spectrometer (LCMS/MS). With the LCMS/MS system two substances with the same retention time, can still be differentiated by their fragmentation pattern. Since metabolites carrying a primary alcohol functionality can form glucuronide and sulfate conjugates, we performed an enzymatic workup of the urine sample in order to cleave them. Apart from the parental drug, we detected 4-OH-MMC and 4-MC (nor-meph) which we have both already identified in our previous publication (Mayer et al., 2019), and the two previously uncharacterized metabolites 4-COOH-MC and dihydro-4-MC (Figure 6). The amino alcohols were found in both syn- and anti-configuration in a 1:1 ratio (Figure 6). The 4-OH-MMC was the only metabolite with benzyl alcohol feature that was detected in human urine. All the others benzyl alcohols, namely 4-OH-MC, dihydro-4-OH-MC and dihydro-4-OH-MMC, were not found, even after enzymatic hydrolysis of potential glutaryl or sulfate conjugates.

## Discussion

The aim of the present study was to determine the pharmacological activity and enantioselectivity of different phase I metabolites of mephedrone, together with their structure-activity relationships and their overall pharmacological relevance.

In accordance with our previous study (Mayer et al., 2019), we found that dihydro-(R,R)-4-MC retains similar potency to mephedrone at DAT, with the dihydro-(R,S)- and the dihydro-(S,R)-4-MC showing only minor changes in their IC50 at DAT compared to mephedrone. Only the carboxylation of mephedrone moiety dramatically impaired the activity of the metabolites at all three monoamine transporters. Contrary to recent studies showing that increasing the bulkiness of the *para-*substitutent, may increase the activity at SERT (Bonano et al., 2015; Sakloth et al., 2015), the introduction of the HO-CH_2_ decreases the activity on SERT, highlighting the complexity of rationally designing drugs with specific transporter activity. Another important finding of our analysis is that the reduction of the β-keto group to the β-hydroxyl group introduces an additional stereocenter conferring a modest DAT/NET stereoselectivity.

These data indicate that subtle changes in chemical properties (i.e., hydroxylation and carbonyl reduction) of mephedrone render the metabolites inactive, which fits with the known role of the hepatic biotransformation reactions to detoxify xenobiotics (Liska, 1998). Moreover, it has also been shown that subtle modifications of cathinone structures can lead to hybrid compounds, behaving as substrate at one transporter and as blocker at another (Saha et al., 2015), or to partial releasers (Niello et al., 2019).

Therefore, by using transporter-mediated currents in HEK293 cells stably expressing DAT or SERT we have investigated if metabolism can modify to some extent the substrate/blocker profile of these drugs. Transporter-mediated currents have been extensively used in the recent past to study monoamine transporters, and even if their existence in native tissues is still a matter of debate, they represent an important tool for obtaining important pharmacological information without the need of synthesizing radiolabeled compounds (Gerstbrein and Sitte, 2006). Given the relatively large number of compounds tested in this study and the focus on the effect of metabolism on mephedrone moiety, we did not extrapolate dose-response curves and therefore we cannot conclude if metabolism convert the full-releaser mephedrone in a partial substrate. Future work will be dedicated to this aspect. However, given that the metabolites tested for transporter-mediated currents where all having an IC50 < 30µM, we can conclude that the absence of DAT-mediated current seen for the metabolites, and especially for the higher affinity dihydro-(R,R)-4-MC and the dihydro-(S,R)-4-MC, is most likely due to their activity as blockers of DAT. To further investigate the effect of metabolism on the substrate/blocker profile of the putative metabolites, we have correlated the change in the IC50s of the compounds at DAT and SERT relative to mephedrone with the changes in transporter-mediated currents elicited by the metabolites in comparison to mephedrone. We could see that while at SERT the loss in affinity and in transport efficiency are linearly correlating, at DAT the changes in the current profiles do not show any correlation with the changes in affinity. This suggest that mephedrone metabolism is more largely affecting the substrate/blocker profile of the resulting moieties at DAT, while at SERT it rather reduces their affinities. The conversion of Mephedrone in metabolites that cannot be transported by DAT limits the interaction of these drugs with the intracellular compartment, possibly reducing the monoamine depletion observed in the case of different amphetamines and congeners (Sitte and Freissmuth, 2015). The reason behind that is still unknown, and future studies will be required to address this point.

At SERT instead, the lack of current due to the impaired interaction between the metabolites and their target, is in line with our computational data. The reduction of cathinone β-ketone to an OH- group and the R,R-configuration of the resulting metabolites drastically impairs the affinity at hSERT. Especially, we found that the 4-CH2OH analogs are considerably less active than the respective 4-CH_3_ derivatives and that this is possibly due to the placement of the hydroxyl group into the hydrophobic subpocket B, described as playing a major role in substrate specificity (Navratna and Gouaux, 2019). Unfortunately, we could not provide a binding model for the different enantiomers since docking procedures could not detect differences in poses between the tested enantiomers.

While the analysis of the putative metabolites can provide important information regarding structure-activity relationships, the translational validity of our data stands on the presence of such chemical structures in the body of mephedrone users. From this point of view, having the putative metabolites in a pure chemical form, allowed us to look for their presence in a urine sample of a mephedrone user. Consistently with a recent study showing a stereoselective metabolism of mephedrone (Castrignanò et al., 2017), we were able to detect the 4-OH-MMC, 4-MC, 4-COOH-MC and both the *anti*- and the *syn*-dihydro-4-MC. Interestingly, we did not find in the urine sample analyzed here the dihydro-4-MMC which was characterized in our previous study (Mayer et al., 2019). While in the case of dihydro-4-MMC found in our previous study the *syn:anti* ratio was approximately 2:1 (Mayer et al., 2019), the dihydro-4-MC detected here is present in 1:1 *syn:anti* ratio suggesting that the stereoselective nature of the metabolic process may vary across different metabolites and/or across different subjects.

Most of the metabolites identified in this and our previous studies (Mayer et al., 2019) were also detected in a recent study in rats (Linhart et al., 2016). However, in this study conjugated metabolites such as the succinyl, adipyl and glutaryl conjugates of 4-MC, as well as 4-MC carboxylated on position 3’ and 4-COOH analogs of 4-MC, dihydro-4-MC and dihydro-4-MMC were also found (Linhart et al., 2016). The different results in the metabolite identifications may be due to different factors.

In our study we did not detect any 4-OH-MC or dihydro-4-OH-MC, whereas Linhart et al. did and report them to be minor metabolites, in relative abundance 0.1–0.2%. The low abundance of 4-OH-MC and all stereoisomers of dihydro-4-OH-MC might be one of the reasons why they were not detected in our study.

Inter-species differences may also account for these findings. 4-OH-MC could be detected in rat urine but not in human urine (Meyer et al., 2010). However, a comparative study on enzymes of CYP family among various species (humans, dogs, rats, mice and monkeys) highlights that CYP 2E1, the main enzyme responsible of MEPH metabolism, does not show significant differences between species (Pedersen et al., 2013). However, other isoforms like CYP-1A, -2C, -2D and -3 A display interspecies differences which include expression, organ specificity and catalytic activity. Further studies need to be conducted to clarify this point.

According to a previous study (Olesti et al., 2019) mephedrone plasma concentrations range between 16 and 222 ng h/ml (∼0.090–1.25 μM h), depending on different factors including polymorphisms of the CYP2D6. Among the metabolites, COOH-MMC was the most abundant, followed by the nor-MMC and dihydro-MMC. Plasma nor-mephedrone reached 25% of the plasma mephedrone, while dihydro-mephedrone reached 10% of the plasma mephedrone. According to these values it seems unlikely that metabolites with IC50 around 10uM could exert any action in the CNS. However, 1) they might still be involved in peripheric effects, or 2) they might still act centrally if generated locally in the CNS. CYP’s are not exclusively expressed in the liver; various CYP isoforms have been detected in the nervous system (Miksys and Tyndale, 2002; Ferguson and Tyndale 2011). For instance, hippocampal and striatal CYP2D6 (Chinta et al., 2002) may generate these metabolites locally, where by gaining access to MATs and monoamine receptors, they may partially shape the overall effects of mephedrone.

**FIGURE 7:**
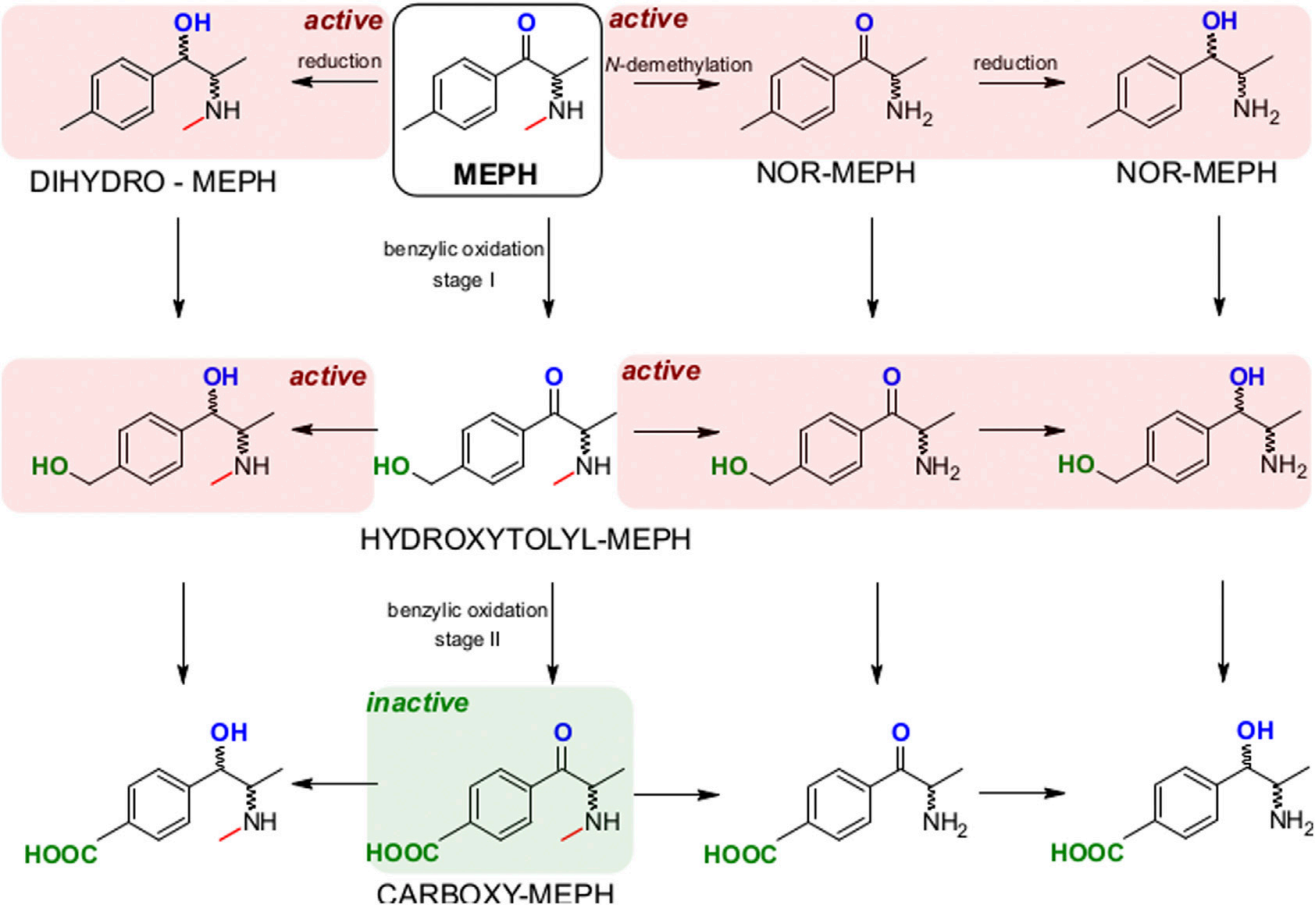
Model of MEPH metabolism with the pathways leading to still active or inactive metabolites. N‐demethylation and reduction lead to active metabolites (red background) which are still active at DAT and NET. Instead, the benzylic oxidation leads to the carboxy‐MEPH (4‐COOH‐MC, green background) which is inactive at DAT, NET and SERT.

In any case, altogether our data allow modeling the complexity of mephedrone metabolism (Figure 7). Our preliminary map highlights that while the N-demethylation and the reduction of mephedrone are producing metabolites with a complete, or only moderately reduced, activity at monoamine transporter, the benzylic oxidation quickly leads to the 4-COOH-MC, which we have found inactive at DAT, NET and SERT. Based on our study, the development of pharmacological treatments shifting mephedrone metabolism toward the benzylic oxidation route may be of help in developing future treatments for mephedrone acute intoxications. Indeed, mephedrone use has been reported being increased world-wide and with an increased binge use in some countries, causing intoxication and possibly lead to fatalities (Hockenhull et al., 2016; Ordak et al., 2018).

In addition, the development of structure-activity relationships for monoamine transporter can provide important information for the development of future medications. Indeed, transporter pharmacology has recently obtained renewed attention thanks to the emergence of atypical inhibitors (Reith et al., 2015), partial releasers (Rothman et al., 2012; Hasenhuetl et al., 2019) and allosteric modulators (Niello et al., 2020). Discovering innovative pharmacological tools to effectively modulate their functions may help in developing new medications for treating different medical conditions.

## Data Availability

The raw data supporting the conclusions of this article will be made available by the authors, without undue reservation.
