# Supplementary material for: Effects of Hydroxylated Mephedrone Metabolites on Monoamine Transporter Activity in vitro
Source: Front Pharmacol. 2021 Apr 9;12:654061. doi: 10.3389/fphar.2021.654061 (PMC8063026; doi:10.3389/fphar.2021.654061)

# SUPPLEMENTARY INFORMATION

## Chemical synthesis

Unless noted otherwise, all reagents were purchased from commercial suppliers and used without further purification. DCM, $Et_2O$, dioxane, MeOH, THF and toluene intended for water-free reactions were pre-distilled and then desiccated on $Al_2O_3$ columns (PURESOLV, Innovative Technology). For all other solvents, quality grade is given in the reaction procedures.

### NMR spectroscopy

NMR spectra were recorded on a Bruker *AC 200* ($^1$H: 200 MHz, $^{13}$C: 50 MHz) and Bruker *Avance Ultrashield 400* ($^1$H: 400 MHz, $^{13}$C: 101 MHz) and Bruker *Avance IIIHD 600* spectrometer equipped with a Prodigy BBO cryo probe ($^1$H: 600 MHz, $^{13}$C: 151MHz). Chemical shifts are given in parts per million (ppm) and were calibrated with internal standards of deuterium labeled solvents $CDCl_3$ ($^1$H 7.26 ppm, $^{13}$C 77.16 ppm), MeOD ($^1$H 3.31 ppm, $^{13}$C 49.00 ppm), $D_2O$ ($^1$H 4.80 ppm, $^{13}$C N/A ) and DMSO-$d_6$ ($^1$H 2.50 ppm, $^{13}$C 39.52 ppm). NMR assignments of unknown compounds were confirmed by $^1$H - $^1$H COSY, $^1$H - $^{13}$C, HSQC and $^1$H - $^{13}$C, HMBC and by comparison to predicted spectra. Proton multiplicities are denoted by the following abbreviations: s (singlet), brs (broad singlet), d (doublet), dd (doublet of a doublet), ddd (doublet of a doublet of a doublet), t (triplet), dt (doublet of a triplet), q (quartet), dq (doublet of a quartet), p (quintet), hep (septet), m (multiplet). Coupling constants (*J*) are presented in Hz (Hertz). Carbon multiplicities (suppressed CH coupling) are denoted by the following abbreviations: s (singlet), d (doublet), t (triplet) and q (quartet). In case of fluoro structures the coupling constant is denoted generally as “xy, $^zJ_{C,F}$ = …Hz“ whereby x represents the multiplicity of the CH coupling, y the multiplicity of the CF coupling and z the order of spin-spin coupling.

### Chromatographic methods

TLC was performed using silica gel 60 aluminum plates containing fluorescent indicator from Merck and detected either with UV light at 254 nm or by charring in ninhydrin solution (300 mg ninhydrin, 3 mL acetic acid, 100 mL butanol), 2,4-dinitrophenylhydrazine (0.8 g 2,4-dinitrophenylhydrazine, 200 mL 2N HCl, 2 mL EtOH) or potassium permanganate (1 g $KMnO_4$, 6.6 g $K_2CO_3$, 100 mg NaOH, 100 mL $H_2O$ in 1M NaOH) with heating.

Column chromatography was performed on a Büchi Sepacore Flash System (2 x Büchi Pump Module C-605, Büchi Pump Manager C-615, Büchi UV Photometer C-635, Büchi Fraction Collector C-660) or standard manual glass columns using silica gel from Merck (40-63 µm) using LP or *n*-hexane and DCM, $Et_2O$ or EtOAc mixtures.

GC/MS spectra were measured on a Thermo Trace 1300 / ISQ LT (single quadrupole MS (EI)) using a standard capillary column BGB 5 (30 m x 0.25 mm ID).

Enantiomeric excess was determined *via* normal phase HPLC with a ChiralPak AS-H (250 mm x 4.6 mm ID) or or IB (250 mm x 4.6 mm ID) column on a Thermo Scientific/Dionex Ultimate 3000 HPLC using mixtures of *n*-hexane/*n*-heptane and *i*PrOH/EtOH. Basic analytes were eluted using 0.1 vol% diethylamine.

Enantiomeric excess of some hydrochloride salts was determined *via* reverse phase Nexera X2® UHPLC system (Shimadzu®, Kyoto, Japan) with Astec® Cyclobond I 2000 Ac column (250 mm x 4.6 mm) using mixtures of water/buffer (pH adjusted to 4-6, 20 mM $NH_4OAc$ or $(NH_4)_2HPO_4$) and MeOH/MeCN.

**Melting point**

Melting points were determined by a Leica Galen III Kofler microscope and a Büchi Melting Point B-545 device.

**HR-MS**

An Agilent 6230 LC TOFMS mass spectrometer equipped with an Agilent Dual AJS ESI-Source was used for the analysis. The mass spectrometer was connected to a liquid chromatography system of the 1100/1200 series from Agilent Technologies, Palo Alto, CA, USA. The system consisted of a 1200SL binary gradient pump, a degasser, column thermostat, and an HTC PAL autosampler (CTC Analytics AG, Zwingen, Switzerland). A silica-based Phenomenex C-18 Security Guard Cartridge was used as stationary phase. Data evaluation was performed using Agilent MassHunter Qulitative Analysis B.07.00. Identification was based on peaks obtained from extracted ion chromatograms (extraction width ± 20 ppm).

**Specific rotation**

Specific rotation $[\alpha]_D^{20}$ was determined using an MCP 500 polarimeter from Anton Paar by the following equation: $[\alpha]_D^{20} = 100*\alpha/[c]*l$; c in [g/100 mL], l in [dm].

## General remarks to the synthesis

The main metabolic pathway of mephedrone (**Supplementary Figure 1**) includes *N*-demethylation, oxidation of the *para*-methyl moiety first to a primary alcohol and then further to a carboxylic acid, and finally reduction of the keto group to a secondary alcohol.

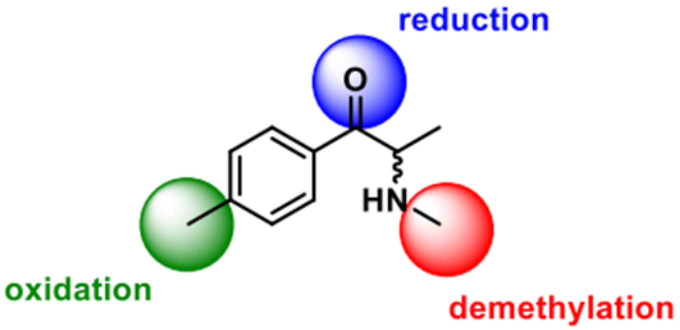


**Supplementary figure 1**

Methcathinones in general can be obtained via the well-established pathway (**Supplementary figure 2**). For the synthesis of mephedrone metabolites we utilized (or slightly modified) the same synthetic pathway as for the methcathinones. The common building block for all mephedrone metabolites was the chiral α-amino ketone (**Supplementary figure 3**) obtained via coupling of Weinreb amides either with the corresponding aromatic Grignard reagent or with aryllithium reagents. The desired metabolites were then obtained after stereoselective reduction of ketone moiety or oxidation of para-methyl substituent on the phenyl ring.

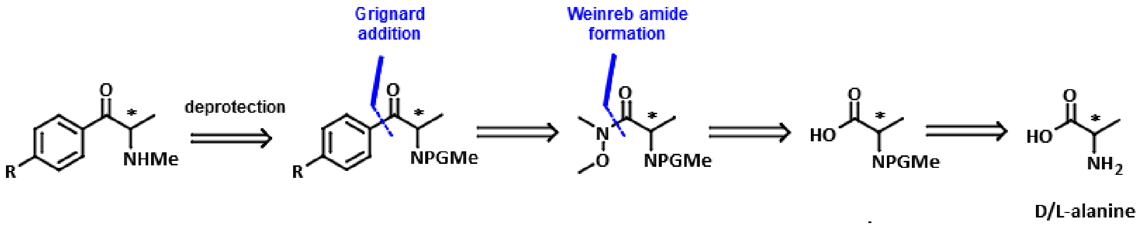


**Supplementary figure 2:** General retrosynthetic analysis of methcathinones.

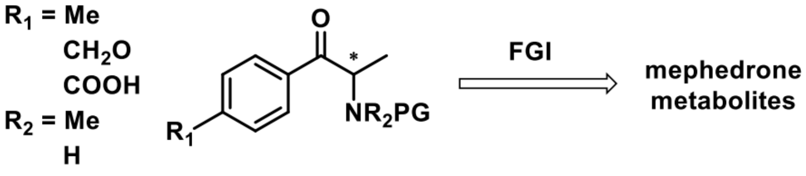

## Supplementary figure 3

## GENERAL PROCEDURES

### General procedure A: Preparation of Grignard reagents

A three-neck flask equipped with condenser, thermometer, septum and magnetic stirring bar was evacuated and dried using a hot-air gun. After cooling to room temperature it was flushed with argon and charged with magnesium turnings (1 equiv.). Usually 1 - 2 mL of solvent were added, followed by dropwise addition of neat aryl bromide. Change of color and the formation of an exotherm indicated start of the reaction, upon which the rest of the solvent was added (the amount of solvent was calculated to give the final 1M or 0.5M solution of Grignard reagent). The remaining aryl bromide was slowly added dropwise, and the reaction was stirred until magnesium was fully dissolved.

**4-Tolylmagnesium bromide**

| | |
|---|---|
| **Concentration, solvent** | 1M in THF |
| **Reaction time** | Dropwise addition of 1-bromo-4-methylbenzene over 15 min (first at rt, then under cooling), 1h heated to 50 °C |
| **Appearance** | Dark brown solution |

**4-Trifluoromethylphenylmagnesium bromide**

| | |
|---|---|
| **Concentration, solvent** | 0.5M in THF |
| **Reaction time** | Dropwise addition of 1-bromo-4-(trifluoromethyl) benzene under cooling, 45 min at rt. |
| **Appearance** | Dark red solution |

**General procedure B: Addition of Grignard reagents to alanine-derived Weinreb amides**

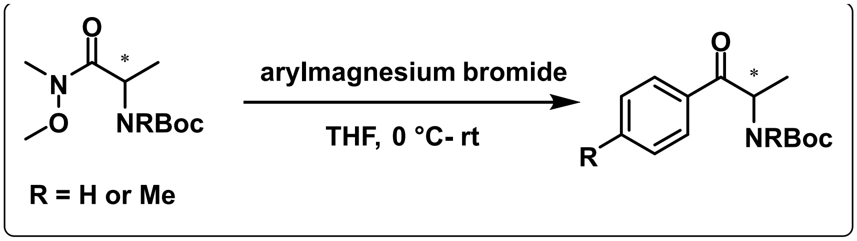


In an oven-dried three-neck round bottom flask equipped with septum, thermometer and magnetic stirring bar, the corresponding Weinreb amide (1 equiv.) was dissolved in dry THF (c = 0.14M) and cooled to 0 °C. A solution of arylmagnesium bromide (1 M or 0.5M, 3 equiv.) was added dropwise. The amount of solvent used to dissolve the starting material was adjusted to give a final concentration of 1 mmol/10 mL. The reaction was maintained at 0 °C for one hour, warmed to rt and stirred for 0.5 - 2 h until full conversion was indicated by TLC. After completion the reaction was cooled to 0 °C and quenched by addition of satd. $NH_4Cl$ and water. The mixture was extracted with three times with $Et_2O$, dried over anh. $MgSO_4$ and evaporated.

**General procedure C: Addition of (4-(((tert-butyl dimethyl silyl)oxy)methyl)phenyl)lithium to alanine-derived Weinreb amides**

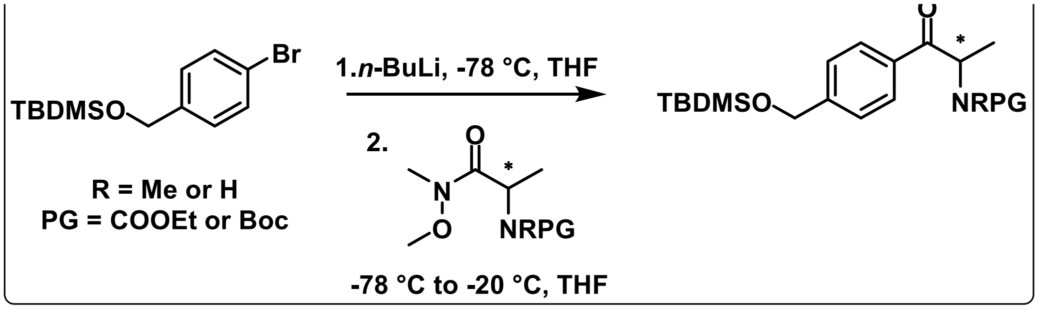


In a three-neck round bottom flask equipped with septum, thermometer and a magnetic stirring bar ((4-bromobenzyl)oxy)(tert-butyl)dimethylsilane (2 equiv.) was dissolved in dry THF (c = 0.1M) under argon. After cooling to -78 °C (internal temperature), *n*-BuLi (2.5 M in hexanes, 2 equiv.) was added dropwise via a syringe over 15 min. After stirring for 1 hour at this

temperature the corresponding Weinreb amide (1 equiv. dissolved in THF) was added at -78 °C dropwise over 15 min. The mixture was allowed to warm to -30 °C over the course of 1 hour and kept at this temperature until TLC showed full conversion. The reaction was quenched with $NH_4Cl$ and water, allowed to warm to rt and extracted twice with EtOAc. The combined organic phases were washed with brine, dried over anh. $MgSO_4$ and evaporated under reduced pressure. The pure product was obtained after flash column chromatography on silica using LP/ EtOAc mixtures.

**General procedure D: Stereoselective reduction of ketones with $NaBH_4$**

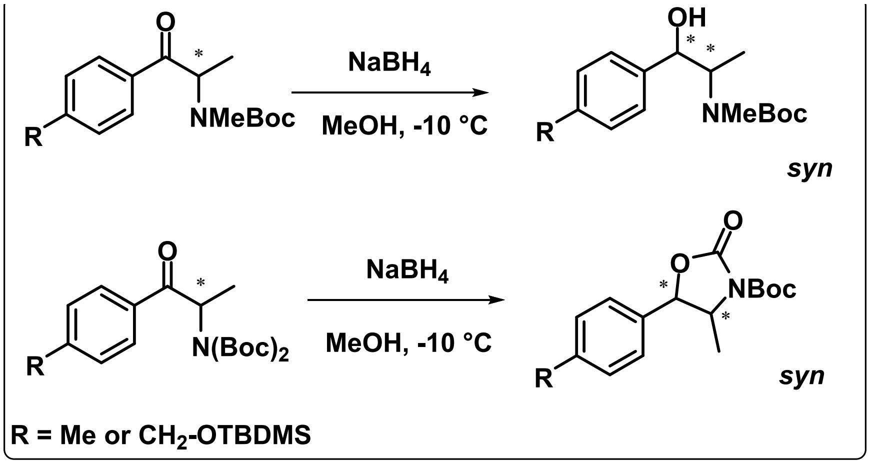


An oven dried three-neck round bottom flask equipped with magnetic stirring bar and thermometer was set under argon and charged with the corresponding ketone (1 equiv.) dissolved in dry MeOH (c = 0.07M). The mixture was then cooled to -10 °C. Sodium borohydride (2 equiv.) was added in one portion and the reaction was stirred at that temperature until TLC showed full consumption of the starting material. The reaction was quenched by addition of water (whereas a colorless precipitate was formed), concentrated *in vacuo* and lyophilized. The solid residue was taken up in a small amount of water (up to 5 mL) and extracted three times with EtOAc. The combined organic layers were washed with brine, dried over anh. $MgSO_4$ and evaporated. The pure *syn* diastereoisomer was then obtained via flash column chromatography on silica using LP/EtOAc mixtures.

**General procedure E: Stereoselective reduction of ketones with $Zn(BH_4)_2$**

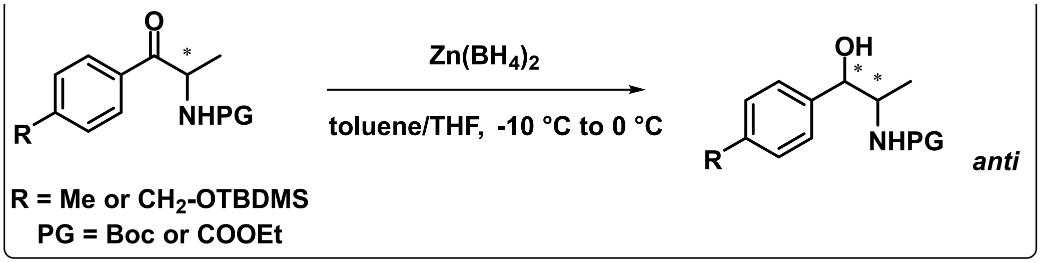


**Preparation of $Zn(BH_4)_2$ reagent**: A three-neck round bottom flask was dried under argon using a hot gun and equipped with a reflux condenser. It was then charged with 4.18 g (83 mmol) of freshly fused $ZnCl_2$ and 2.59 g (66 mmol) of $NaBH_4$, followed by addition of dry THF (50 mL) using a double-ended needle. The mixture was stirred for 24 hours at room temperature. The supernatant was decanted, transferred to a Schlenk tube under argon and kept in the fridge where it was found to be stable over several months. The concentration of the supernatant was estimated to be 0.66 M in $Zn(BH_4)_2$ according to the literature protocol and it was used as such for further reactions.An oven dried three-neck round bottom flask equipped with a thermometer and magnetic stirring bar was set under argon, charged with the corresponding ketone (1 equiv.) dissolved in dry toluene (c = 0.04M) and cooled to -10 °C. Then $Zn(BH_4)_2$ (0.66 M in THF, 1.1 to 1.3 equiv.) was added dropwise. The reaction mixture was allowed to warm to 0 °C and when TLC indicated full conversion, it was quenched by addition of ice water and extracted three times with EtOAc. The combined organic layers were washed with brine, dried over anh. $MgSO_4$ and evaporated. The pure *anti* diastereoisomer was then obtained via flash column chromatography on silica using LP/EtOAc mixtures.

**General procedure F: Boc- and TBDMS-deprotection of methcathinones and mephedrone metabolites containing a keto-group**

In an 8-mL screw-cap vial Boc-protected compounds (1 equiv.) were dissolved in a small amount of HPLC-grade dioxane and cooled to 0 °C, upon which they were treated with pre-cooled 6 M HCl in $H_2O$/dioxane = 4/1 (this solution was prepared by mixing 50 mL of conc. HCl and 20 mL of dioxane and diluting the mixture to 100 mL with deionized water) to give a 0.2 M solution. The reaction mixture was stirred at 0 °C for 7 to 24 h. After completion of the reaction a transparent solution was obtained. The reaction mixture was extracted multiple times with $Et_2O$ or EtOAc and the aqueous layer was evaporated in a stream of pressurized air. The residue

was taken up in deionized water, filtered through a syringe filter and lyophilized to obtain the desired hydrochlorides.

**General procedure G: Boc- and TBDMS-deprotection of mephedrone metabolites containing a secondary hydroxyl group**

An oven-dried 8-mL screw-cap vial was charged with Boc-protected mephedrone metabolites (1 equiv.) and set under argon atmosphere. Then dry DCM (c=0.1M) was added, the mixture was cooled to 0 °C and trifluoroacetic acid (15 equiv.) was added dropwise. The reaction mixture was stirred at 0 °C and after TLC indicated full conversion the volatiles were removed under a flow of pressurized air. The oily residue was taken up in aq. NaOH solution (1M saturated with NaCl) in an amount to create approximately 0.1 M solution. It was then extracted multiple times with EtOAc, the organic phases were combined, dried over anh. $MgSO_4$ and filtered. Then excess of HCl (2M in $Et_2O$ or 1M in EtOAc) was added under stirring, whereas a colorless precipitate was formed. The solvent was evaporated under reduced pressure and the residue was thoroughly washed multiple times with $Et_2O$. It was then taken up in deionized water, filtered through a syringe filter and lyophilized to afford the corresponding hydrochlorides.

**(*R*/*S*)-*tert*-(Butoxycarbonyl)-alanine (1)**

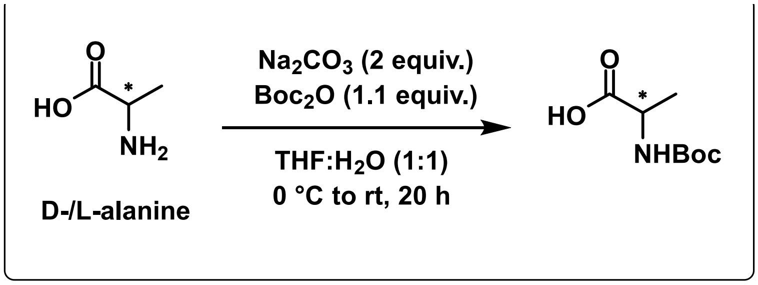


In a round bottom flask (*D*)- or (*L*)-alanine (4 g, 45 mmol, 1 equiv.) was dissolved in 1:1 mixture $H_2O$:THF (60 mL) along with $Na_2CO_3$ (9.5 g, 90 mmol, 2 equiv.). The mixture was cooled down to 0 °C and then $Boc_2O$ (10.8 g, 50 mmol, 1.1 equiv.) was slowly added and the reaction was allowed to warm up to rt and stirred for 20 h. Additional 30 mL of water were added to facilitate stirring of otherwise slurry mixture. After this time pH was adjusted to 2 by careful addition of 1M HCl under cooling. The mixture was extracted with EtOAc (4 x 100 mL), combined organic

phases were washed with brine, dried over $MgSO_4$ and solvent was removed under reduced pressure, yielding the desired product as colorless solid in **99 %** (*R*), resp. **87 % yield** (*S*). The product was used as such without further purification.

**$^1$H NMR** (400 MHz, DMSO-$d_6$) Mixture of rotamers: δ 1.21 (d, *J* = 7.3 Hz, 3H), 1.30 – 1.44 (m, 9H), 3.77 – 3.97(m, 1H), 6.66 – 6.74 (m, 1H), 7.06 (d, *J* = 7.5 Hz, 1H), 12.37 (s, 1H). **$^{13}$C NMR** (101 MHz, DMSO-$d_6$) δ 17.1, 28.2, 48.8, 77.9, 155.3, 174.7; m.p 79 – 82 °C, (*R*): $[\alpha]_D^{20}$ = +25.2° (*c* 1, MeOH), (*S*): $\alpha_D^{20}$ = -24.3° (*c* 1, MeOH).

**(*R/S*)-*N*-(Ethoxycarbonyl)alanine (2)**

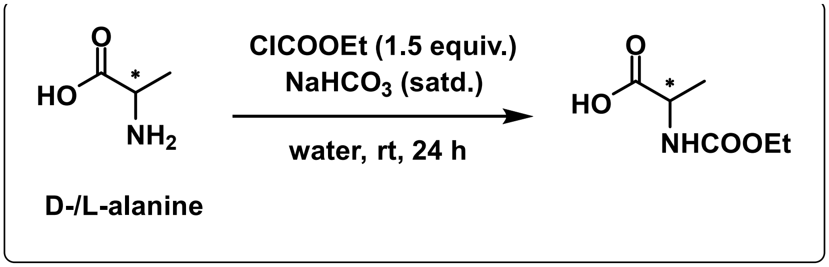


In a round bottom flask (*L*)- or (*D*)-alanine (1.5 g, 16.8 mmol, 1 equiv.) was dissolved in satd. aq. $NaHCO_3$ (75 mL). Ethyl chloroformate (2.4 mL, 25.3 mmol, 1.5 equiv.) was added and the reaction mixture was stirred at rt for 24 h. After this time the reaction mixture was washed with $Et_2O$ (2 x 30 mL), cooled to 0 °C and carefully acidified with 2M HCl to pH 1. The aqueous layer was extracted with EtOAc (3 x 50 mL). The combined organic phases were dried over $MgSO_4$, filtered and evaporated *in vacuo* to give the desired product as colorless viscous oil in **78 %** (*R*), resp. **96 % yield** (*S*). The product was used as such without further purification.

**$^1$H NMR** (400 MHz, DMSO-$d_6$) δ 1.15 (t, *J* = 7.2 Hz, 3H), 1.24 (d, *J* = 7.4 Hz, 3H), 3.93 – 4.01 (m, 2H), 7.39 (d, *J* = 7.6 Hz, 1H), 12.47 (brs, 1H); **$^{13}$C NMR** (101 MHz, DMSO-$d_6$) 14.6, 17.1, 49.1, 59.7, 155.9, 174.5, (*R*): $[\alpha]_D^{20}$ = +15.9° (*c* 1, DCM), (*S*): $\alpha_D^{20}$ = -15° (*c* 1, DCM).

**(*R*/*S*)-*N*-(*tert*-Butoxycarbonyl)-*N*-methyl-alanine (3)**

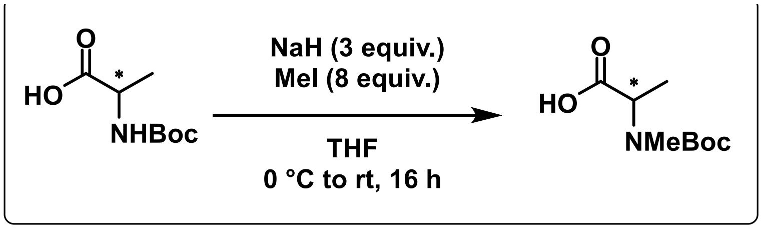


In an oven-dried round bottom flask (*L*)- or (*D*)-*N*-Boc-alanine (2 g, 10.6 mmol, 1 equiv.) was dissolved in dry THF (35 mL) under argon and cooled to 0 °C. Then sodium hydride (1.4 g, 31.8 mmol, 3 equiv., 60% in mineral oil) was added in three portions and the reaction was stirred for 1 h at 0 °C. Then neat MeI (5.3 mL, 84.8 mmol, 8 equiv.) was added dropwise via syringe at 0 °C and the reaction mixture was allowed to warm to room temperature and let stir for 16 h. The reaction was quenched with water (100 mL) and extracted with $Et_2O$ (100 mL). The organic phase was separated and washed with satd. $NaHCO_3$ (80 mL). The aqueous phases were then combined, pH was adjusted to 2 by careful addition of 2M HCl under cooling and extracted with EtOAc (3 x 80 mL). The combined organic layers were then washed with satd. $Na_2S_2O_3$ (3 x 50 mL), dried over anh. $MgSO_4$ and the solvent was evaporated under reduced pressure, furnishing the desired product as colorless solid in **80 %** (*R*), resp. **91 % yield** (*S*). The product was used as such without further purification which was used as such without need for further purification.

**$^1$H NMR** (400 MHz, DMSO-$d_6$) Mixture of rotamers ≈ 60:40: δ 1.24 –1.33 (m, 3H), 1.32 – 1.45 (m, 9H), 2.73 (m, 3H), 4.54 (m, 0.5H), 4.27 (m, 0.5H); **$^{13}$C NMR** (101 MHz, DMSO-$d_6$): δ 14.6, 15.2, 27.9, 28.0, 30.6, 31.5, 53.2, 54.8, 79.0, 154.6, 155.0, 173.3, 173.4; m.p 89 – 92 °C, (*R*): $[\alpha]_D^{20}$ = +45.6° (*c* 1, DCM), (*S*): $\alpha_D^{20}$ = -42.5° (*c* 92, DCM).

**(R)/(S)-*tert*-Butyl (1-(methoxy(methyl)amino)-1-oxopropan-2-yl)(methyl)carbamate (4)**

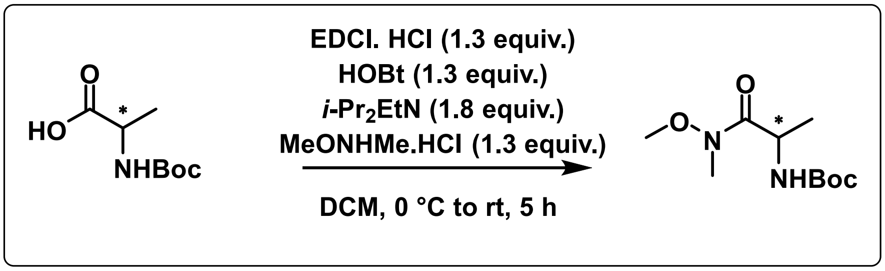


An oven-dried three-neck round bottom flask was charged with (*D*)- or (*L*)-*N*-Boc-alanine (4 g, 21.2 mmol, 1 equiv.) and dry DCM (85 mL) under argon and then 1-hydroxybenzotriazol (4.2 g, 27.5 mmol, 1.3 equiv.), *N,O*-dimethylhydroxylamine hydrochloride (2.7 g, 27.5 mmol, 1.3 equiv.) and *i*-$Pr_2EtN$ (5.5 mL, 31.8 mmol, 1.5 equiv.) were added successively. The reaction mixture was cooled to 0 °C and *N*-ethyl-*N′*-(3-dimethylaminopropyl)carbodiimide hydrochloride (5.3 g, 27.5 mmol, 1.3 equiv.) was added in one portion. The reaction was allowed to warm to rt and stirred for 5 h when TLC indicated full conversion. The reaction was quenched by addition of water (100 mL) and extracted with DCM (3 x 75 mL). The combined organic layers were washed with 1M HCl (100 mL), satd. $NaHCO_3$ (100 mL) and brine (100 mL), dried over anh. $MgSO_4$ and evaporated, yielding the desired product as off-white crystals in **85 %** (*R*), resp. **99 % yield** (*S*). The product was used as such without further purification which was used as such without need for further purification.

**$^1$H NMR** (400 MHz, $CDCl_3$) δ 1.28 (d, *J* = 7.0 Hz, 3H), 1.41 (s, 9H), 3.18 (s, 3H), 3.74 (s, 3H), 4.73 – 4.56 (m, 1H), 5.24 (d, *J* = 8.1 Hz, 1H); **$^{13}$C NMR** (101 MHz, $CDCl_3$) 18.8, 28.5, 32.3, 46.63, 61.7, 79.6, 155.3, 173.8; m.p 146 – 147 °C, (*R*): $[\alpha]_D^{20}$ = +28.5° (*c* 0.95, MeOH), (*S*): $\alpha_D^{20}$ = -25.3° (*c* 1, MeOH).

**(R)/(S)-*tert*-Butyl (1-(methoxy(methyl)amino)-1-oxopropan-2-yl)(methyl)carbamate (5)**

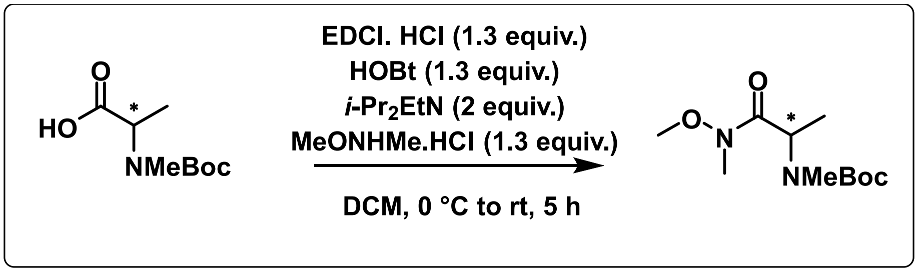


(*D*)- or (*L*)-*N*-Me-*N*-Boc-alanine (1.5 g, 7.4 mmol, 1 equiv.) was dissolved in dry DCM (30 mL) under argon and then 1-hydroxybenzotriazol (1.5 g, 9.6 mmol, 1.3 equiv.), *N,O*-dimethylhydroxylamine hydrochlorid (0.94 g, 9.6 mmol, 1.3 equiv.) and *i*-$Pr_2EtN$ (2.3 mL, 13.3 mmol, 2 equiv.) were added successively. The reaction mixture was cooled to 0 °C and EDCI hydrochloride (1.8 g, 9.6 mmol, 1.3 equiv.) was added. The reaction was allowed to warm to rt and stirred for 5 h. The reaction was quenched by addition of water (50 mL) and extracted with DCM (3 x 50 mL). The combined organic layers were washed with 1M HCl (50 mL), satd. $NaHCO_3$ (50 mL) and brine (50 mL), dried over anh. $MgSO_4$ and evaporated, yielding the desired product as pale yellow oil in **96 %** (*R*), resp. **99 % yield** (*S*). The product was used as such without further purification which was used as such without need for further purification.
**$^1$H NMR** (400 MHz, DMSO-$d_6$) Mixture of rotamers ≈ 60:40: δ 1.16 – 1.25 (m, 3H), 1.31 –1.47 (m, 9H), 2.56 – 2.72 (m, 3H), 3.09 (s, 3H), 3.67 (s, 3H), 4.77 (q, *J* = 6.3 Hz, 0.5H), 4.99 (q, *J* = 6.3 Hz, 0.5H); **$^{13}$C NMR** (101 MHz, DMSO-$d_6$): δ 14.4, 14.7, 28.0, 29.7, 49.7, 51.2, 61.1, 79.0, 154.4, 154.8,171.9; (*R*): $[\alpha]_D^{20}$ = +57.6° (*c* 1, DCM), (*S*): $\alpha_D^{20}$ = -53.5° (*c* 1.06, DCM).

**(*R*)/(*S*)-Ethyl(1-(methoxy(methyl)amino)-1-oxopropan-2-yl)carbamate (6)**

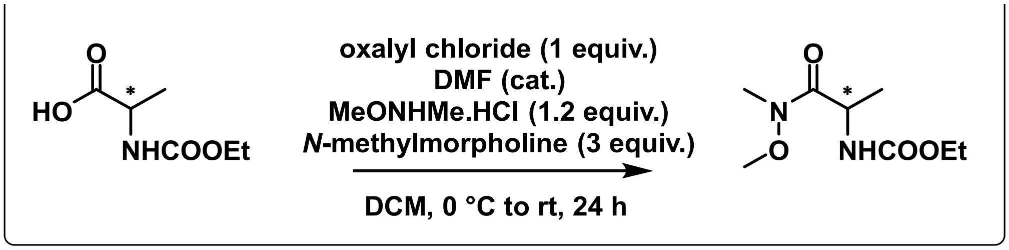


(*D*)- or (*L*)-*N*-(ethoxycarbonyl)alanine (2 g, 12.4 mmol, 1 equiv.) was dissolved in dry DCM (25 mL) in an oven-dried argon-filled round bottom flask equipped with a septum and a magnetic stirring bar. Oxalyl chloride (1 mL, 12.4 mmol, 1 equiv.) was added via syringe, followed by 3 drops of DMF. The reaction mixture was stirred overnight at rt. Then *N,O*-dimethylhydroxylamine hydrochloride (1.45 g, 14.9 mmol, 1.2 equiv) was added and the mixture was cooled to 0 °C upon which *N*-methylmorpholine (4 mL, 37.2 mmol, 3 equiv.) was added dropwise. TLC indicated full conversion after additional 1 h at rt. The reaction mixture was diluted with $Et_2O$ (20 mL) and successively washed with 1M HCl (50 mL), satd. $NaHCO_3$ (50 mL) and brine (50 mL). The organic layer was dried over anh. $MgSO_4$, filtered and evaporated. The residue was subjected to column chromatography (DCM/MeOH = 98/2), yielding the desired product colorless solid in **57 %** (*R*), resp. **65 % yield** (*S*).

**$^1$H NMR** (400 MHz, $CDCl_3$): δ 1.23 (t, *J* = 7.1 Hz, 3H), 1.32 (d, *J* = 6.9 Hz, 3H), 3.20 (s, 3H), 3.76 (s, 3H), 4.09 (q, *J* = 7.1 Hz, 2H), 4.71 (t, *J* = 7.6 Hz, 1H), 5.41 (d, *J* = 8.6 Hz, 1H); **$^{13}$C NMR** (101 MHz, $CDCl_3$): δ 14.7, 18.7, 32.3, 47.0, 61.0, 61.7, 156.1, 173.5; m.p 46 – 47 °C, (*R*): $[\alpha]_D^{20}$ = +3.1° (*c* 0.94, DCM), (*S*): $\alpha_D^{20}$ = -2.61° (*c* 0.85, DCM).

**(*R*)/(*S*)-*tert*-Butyl methyl(1-oxo-1-(p-tolyl)propan-2-yl)carbamate (7)**

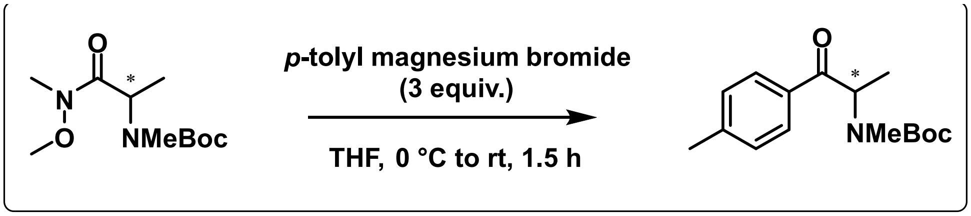


The compound was prepared according to **General procedure B**. The desired compound was obtained after purification (LP/EtOAc = 95/5, 90 g $SiO_2$) as off-colorless solid in **82%** (*R*), resp. **80%** (*S*) yield.

**$^1$H-NMR (400 MHz, DMSO-*d*$_6$)**: Mixture of rotamers ≈ 50:50: δ 1.18 – 1.43 (m, 12H), 2.36 (s, 3H), 2.62 (s, 1.5H), 2.79 (s, 1.5H), 5.08 (q, *J* = 6.7 Hz, 0.5H), 5.42 (q, *J* = 6.8 Hz, 0.5H), 7.22 – 7.37 (m, 2H), 7.70 – 7.84 (m, 2H); **$^{13}$C-NMR (101 MHz, DMSO-*d*$_6$)**: Mixture of rotamers ≈ 50:50: δ 13.3, 13.8, 21.1, 27.7, 27.9, 30.2, 31.7, 55.1, 57.3 (d, C2), 79.3, 79.4, 128.0, 129.1, 132.8, 133.1, 143.2, 143.4, 153.8, 154.6, 198.6, 198.7; m.p. 55 – 57 °C, (*R*): $[\alpha]_D^{20}$ = 148° (*c* 1.1, DCM), >99% ee, (*S*): $[\alpha]_D^{20}$ = -144° (*c* 0.9, DCM), >99% ee; HPLC method: Hexane/EtOH = 99.7/0.3, 1mL/min, 25 °C, 30min, AS-H.

**(*R*)/(*S*)-*tert*-Butyl (1-oxo-1-(p-tolyl)propan-2-yl)carbamate (8)**

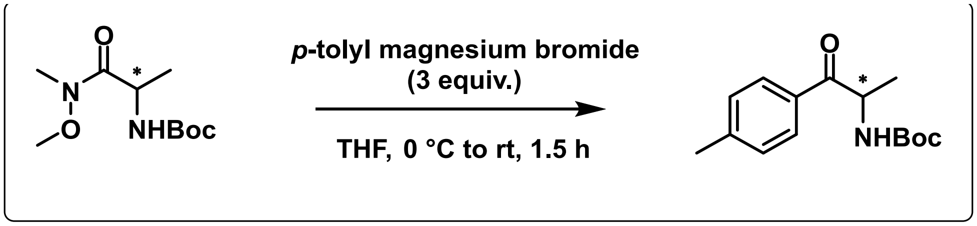


The compound was prepared according to **General procedure B**. The desired compound was obtained after purification (LP/EtOAc = 95/5, 90 g $SiO_2$, followed by re-crystallization from ligroin) as colorless crystals in **61%** (*R*), resp. **58%** (*S*) yield.

**$^1$H-NMR (400 MHz, $CDCl_3$)**: δ 1.39 (d, *J* = 7.1 Hz, 3H), 1.45 (s, 9H), 2.42 (s, 3H), 5.26 (p, *J* = 7.2 Hz), 5.58 (d, *J* = 7.6 Hz, 1H), 7.28 (d, *J* = 7.7 Hz, 2H), 7.87 (d, *J* = 8.2 Hz, 2H); **$^{13}$C-NMR (101 MHz, $CDCl_3$)**: δ 20.3, 21.9, 28.5, 51.1, 79.8, 128.9, 129.7 (d, C3' & C5'), 131.8, 144.8, 155.3, 199.1; m.p. 101 – 103 °C, (*R*): $\alpha_D^{20}$ = -8.4° (*c* 0.95, DCM), ee n.d., (*S*): $[\alpha]_D^{20}$ = +8.7° (*c* 1.2, DCM), ee n.d.

**((4-Bromobenzyl)oxy)(tert-butyl)dimethylsilane (9)**

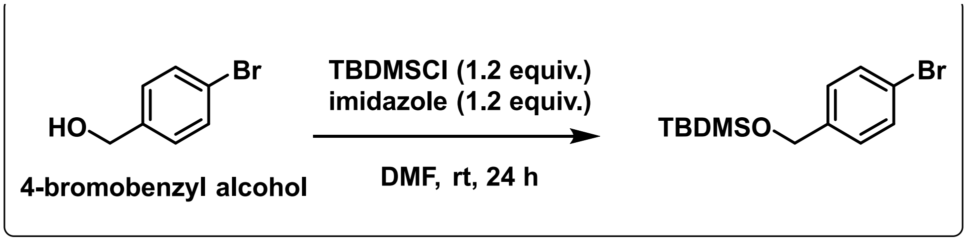


In an oven-dried round bottom flask imidazole (2.2 g, 32.4 mmol, 1.2 equiv.) and *tert*-butyldimethylsilyl chloride (4.9 g, 32.4 mmol, 1.2 equiv.) in DMF (10 mL) were subsequently added to a solution of 4-bromobenzyl alcohol (5 g, 27 mmol, 1 equiv.) in dry DMF (80 mL). The resulting mixture was stirred at rt for 16 hours under argon. When TLC indicated full conversion, the mixture was poured into ice-cold water (200 mL) and extracted with $Et_2O$ (3 x 70 mL). The combined organic phases were washed with satd. aq. $NaHCO_3$ (2 x 100 mL) and brine (100 mL), dried over anh. $MgSO_4$, filtered and concentrated *in vacuo*, yielding a colorless oil in quantitative yield. The product was further used as such without purification.

**$^1$H-NMR (400 MHz, $CDCl_3$)**: δ 0.10 (s, 6H), 0.94 (s, 9H), 4.68 (s, 2H), 7.16 – 7.24 (m, 2H), 7.41 – 7.49 (m, 2H), **$^{13}$C-NMR (101 MHz, $CDCl_3$)**: δ -5.2, 18.5, 26.1, 64.5, 120, 127.9, 131.4, 140.6 ppm.

**(*R*)/(*S*)-*tert*-Butyl(1-(4-(((tert-butyldimethylsilyl)oxy)methyl)phenyl)-1-oxopropan-2-yl)carbamate (10)**

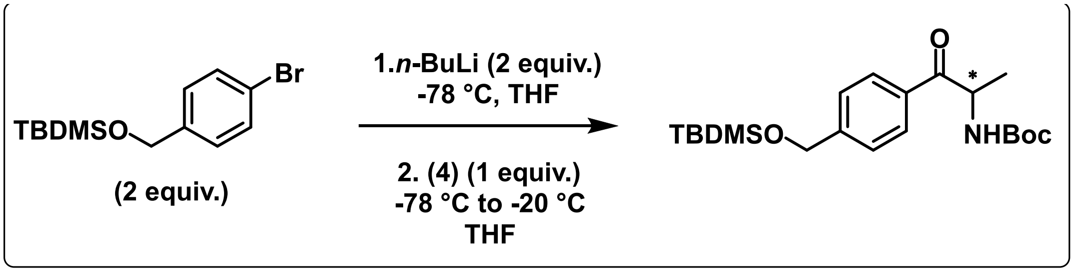


The compound was prepared according to **General procedure C**. The desired compound was obtained after purification (LP/EtOAc = 95/5, 90 g $SiO_2$) as colorless solid in **76%** (*R*), resp. **56%** (*S*) yield.

**$^1$H-NMR (400 MHz, $CDCl_3$)**: δ 0.11 (s, 6H), 0.95 (s, 9H), 1.39 (d, *J* = 7.1 Hz, 3H), 1.45 (s, 9H), 4.80 (s, 2H), 5.28 (p, *J* = 6.8 Hz, 1H), 5.57 (d, *J* = 7.6 Hz, 1H), 7.44 (d, *J* = 8.1 Hz, 2H), 7.94 (d, *J* = 8.1 Hz, 2H); **$^{13}$C-NMR (101 MHz, $CDCl_3$)**: δ -5.2, 18.5, 20.2, 26.0, 28.5, 51.2, 64.6, 79.8, 126.2, 128.9, 133.0, 148.0, 155.3, 199.2; m.p. 77 – 78 °C, $\alpha_D^{20}$ = -6.0° (*c* 0.86, DCM), >99% ee, (*S*): $\alpha_D^{20}$ = +6.34° (*c* 1, DCM), >99% ee; HPLC method: Hexane/IPA = 99.7/0.3, 1mL/min, 25 °C, 60min, IB; HR-MS-ESI: Calc.[M+H]: 394.2408, Found [M+H]: 394.2398 (Diff.: 2.62 ppm).

**(*R*)/(*S*)-*tert*-Butyl (1-(4-(((tert-butyldimethylsilyl)oxy)methyl)phenyl)-1-oxopropan-2-yl) (methyl)carbamate (11)**

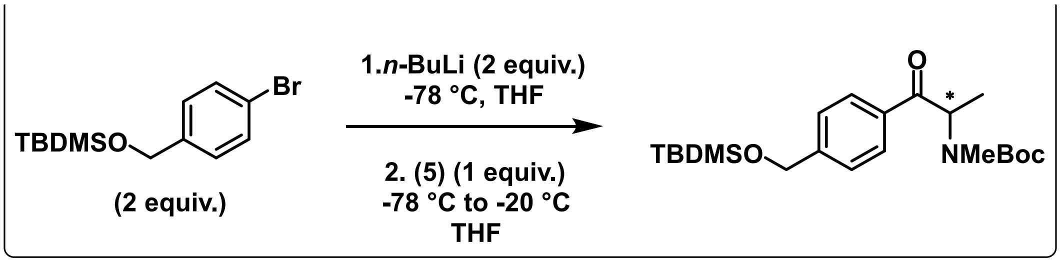


The compound was prepared according to **General procedure C**. The desired compound was obtained after purification (LP/EtOAc 97/3, 90 g $SiO_2$) as colorless oil in **81%** (*R*), resp. **76%** (*S*) yield.

**$^1$H-NMR (400 MHz, $CDCl_3$)**: Mixture of rotamers ≈ 60:40: δ 0.10 (s, 6H), 0.94 (s, 9H), 1.24 – 1.54 (m, 12H), 2.60 (s, 1.8H), 2.79 (s, 1.2H), 4.78 (s, 2H), 5.18 (q, *J* = 6.2, 0.4H), 5.69 (q, *J* = 6.9 Hz, 0.6H), 7.38 (d, *J* = 7.9 Hz, 2H), 7.77 – 8.24 (m, 2H); **$^{13}$C-NMR (101 MHz, $CDCl_3$)**: Mixture of rotamers ≈ 60:40: δ -5.2, 13.4, 13.9, 18.5, 26.0, 28.5, 29.6, 30.8, 54.7, 57.1, 64.7, 80.3, 80.8, 126.0, 128.4, 128.7, 134.3, 147.2, 155.6, 198.7, 199.6; (*R*): $\alpha_D^{20}$ = +106° (*c* 0.64, DCM), >99% ee, (*S*): $\alpha_D^{20}$ = -112° (*c* 0.84, DCM), >99% ee; HPLC method: Hexane/IPA = 99.9/0.1, 1mL/min, 25 °C, 60min, IB; HR-MS-ESI: Calc.[M+Na]: 430.2384**,** Found [M+Na]: 430.2391 (Diff.: -1.58 ppm).

**(*R*)/(*S*)-Ethyl(1-(4-(((*tert*-butyldimethylsilyl)oxy)methyl)phenyl)-1-oxopropan-2-yl)carbamate (12)**

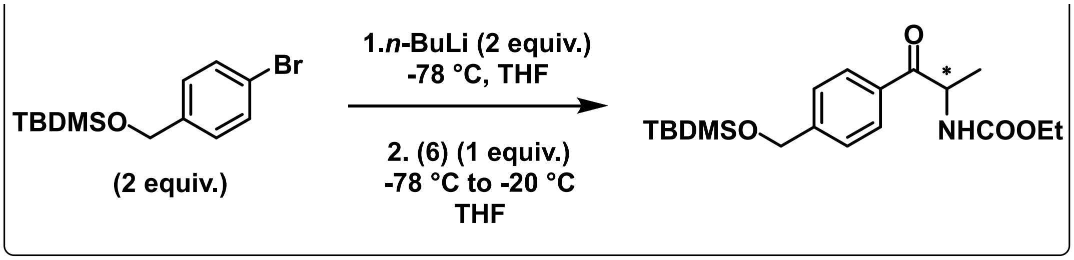


The compound was prepared according to **General procedure C**. The desired compound was obtained after purification (LP/EtOAc 97/3 → 90/1, 90 g $SiO_2$) as colorless oil in **56%** (*R*), resp. **51%** (*S*) yield.

**$^1$H-NMR (400 MHz, $CDCl_3$)**: δ 0.11 (s, 6H), 0.95 (s, 9H), 1.26 (t, *J* = 7.1 Hz, 3H), 1.42 (d, *J* = 7.1 Hz, 3H), 4.13 (q, *J* = 7.1 Hz, 2H), 4.80 (s, 2H), 5.32 (p, *J* = 7.2 Hz, 1H), 5.68 – 5.79 (m, 1H), 7.38 – 7.50 (m, 2H), 7.95 (d, *J* = 8.0 Hz, 2H); **$^{13}$C-NMR (101 MHz, $CDCl_3$)**: δ -5.2, 14.8, 18.5, 20.3, 26.0, 51.6, 61.1, 64.5, 126.2, 128.9, 132.8, 148.1, 156.0, 198.9; (*R*): $\alpha_D^{20}$ = -4.7° (*c* 0.64, DCM), >99% ee, (*S*): $\alpha_D^{20}$ = +4.0° (*c* 0.49, DCM), >99% ee; HPLC method: Hexane/IPA = 99.7/0.3, 1mL/min, 25 °C, 60min, IB; HR-MS-ESI: Calc.[M+Na]: 388.1915**,** Found [M+Na]: 388.1923 (Diff.: -2.23 ppm).

**(*R*)/(*S*)-bis-*tert*-Butyl (1-oxo-1-(p-tolyl)propan-2-yl)carbamate (13)**

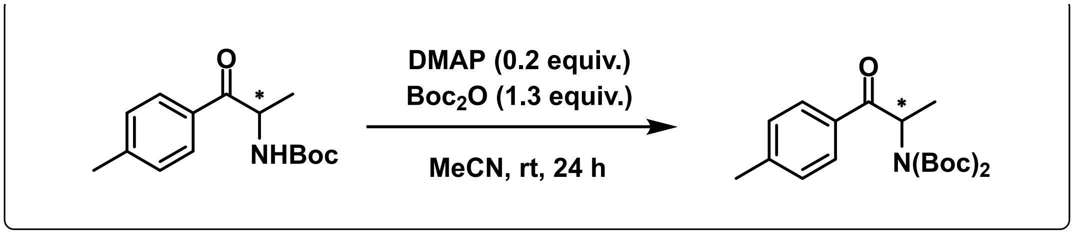


An oven-dried round-bottom flask was charged with (*R*)- or (*S*)-*tert*-butyl-(1-oxo-1-(p-tolyl)propan-2-yl)carbamate, (1.3 g, 4.9 mmol, 1 equiv.) and dry MeCN (16 mL), then 4-dimethylaminopyridine (60 mg, 0.5 mmol, 0.1 equiv.) was added, followed by $Boc_2O$ (1.2 g, 5.4 mmol, 1.1 equiv.) and the mixture was stirred at rt for 6 h. TLC still indicated presence of starting material, so additional 0.1 equiv. of DMAP and 0.2 equiv. of $Boc_2O$ were added and the reaction was stirred for further 24 h, however, no full conversion was achieved. The dark yellow reaction mixture was carefully evaporated at room temperature and the oily residue was partitioned between $Et_2O$ (40 mL) and aq. 1M $KHSO_4$ (40 mL). The organic layer was washed with aq. 1M $KHSO_4$ (40 mL), satd. $NaHCO_3$ (40 mL) and brine (40 mL), dried over anh. $MgSO_4$, filtered and concentrated *in vacuo*. The desired compound was obtained after purification (LP/EtOAc 95/5 → 92/8, 90 g $SiO_2$) as colorless solid in **58%** (*R*), resp. **61%** (*S*) yield.

**$^1$H-NMR (400 MHz, $CDCl_3$)**: δ 1.42 (s, 18H) 1.52 (d, *J* = 6.6 Hz, 3H), 2.38 (s, 3H), 5.45 (q, *J* = 6.6 Hz, 1H), 7.19 (d, *J* = 7.9 Hz), 7.65 (d, *J* = 8.2 Hz, 2H); **$^{13}$C-NMR (101 MHz, $CDCl_3$)**: δ 15.0, 21.7, 28.0, 57.7, 83.5, 128.1, 129.1, 133.6, 143.1, 151.8, 197.7; m.p. 78 – 81 °C, (*R*): $\alpha_D^{20}$ = +83.3° (*c* 1, MeOH), ee n.d., (*S*): $\alpha_D^{20}$ = -82.1° (*c* 1, MeOH), ee n.d., HR-MS-ESI: Calc.[M+Na]: 386.1938**,** Found [M+Na]: 386.1945 (Diff.: -2.73 ppm).

**(*R*)/(*S*)-bis-*tert*-Butyl(1-(4-(((tert-butyldimethylsilyl)oxy)methyl)phenyl)-1-oxopropan-2-yl)carbamate (14)**

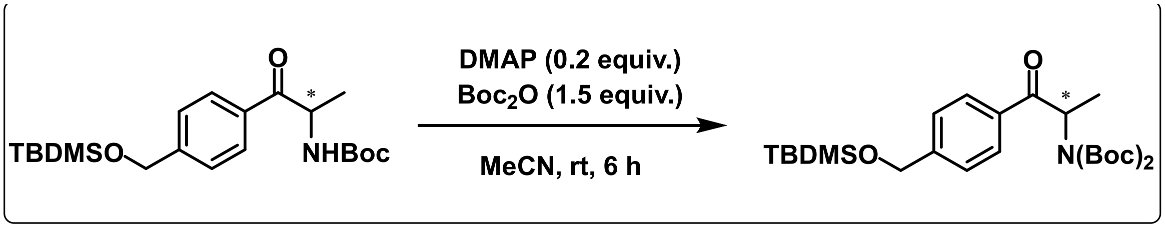


The title compound was prepared identically to procedure in the previous section, however, 1.5 equiv. $Boc_2O$ was used. The desired compound was obtained after purification (LP/EtOAc 95/5 → 92/8, 90 g $SiO_2$) as colorless oil in **59%** (*R*), resp. **56%** (*S*) yield.

**$^1$H-NMR (400 MHz, $CDCl_3$)**: δ 0.09 (s, 6H), 0.93 (s, 9H), 1.41 (s, 18H), 1.53 (d, *J* = 6.6 Hz, 3H), 4.77 (s, 2H), 5.47 (q, *J* = 6.6 Hz, 1H), 7.34 (d, *J* = 8.0 Hz, 2H), 7.71 (d, *J* = 8.3 Hz, 2H); **$^{13}$C-NMR (101 MHz, $CDCl_3$)**: δ -5.1, 14.9, 18.5, 26.0, 28.0, 57.7, 64.7, 83.6, 125.8, 128.1, 135.1, 146.2, 151.9, 198.0; (*R*): $\alpha_D^{20}$ = +60.8° (*c* 1, DCM), ee n.d., (*S*): $\alpha_D^{20}$ = -64.4° (*c* 0.9, DCM), ee n.d., HR-MS-ESI: Calc.[M+Na]: 516.2752**,** Found [M+Na]: 516.2755 (Diff.: -0.66 ppm).

**(1*R*,2*R*)/(1*S*,2*S*)-*tert*-Butyl(1-(4-(((tert-butyldimethylsilyl)oxy)methyl)phenyl)-1-hydroxypropan-2-yl)(methyl)carbamate (15)**

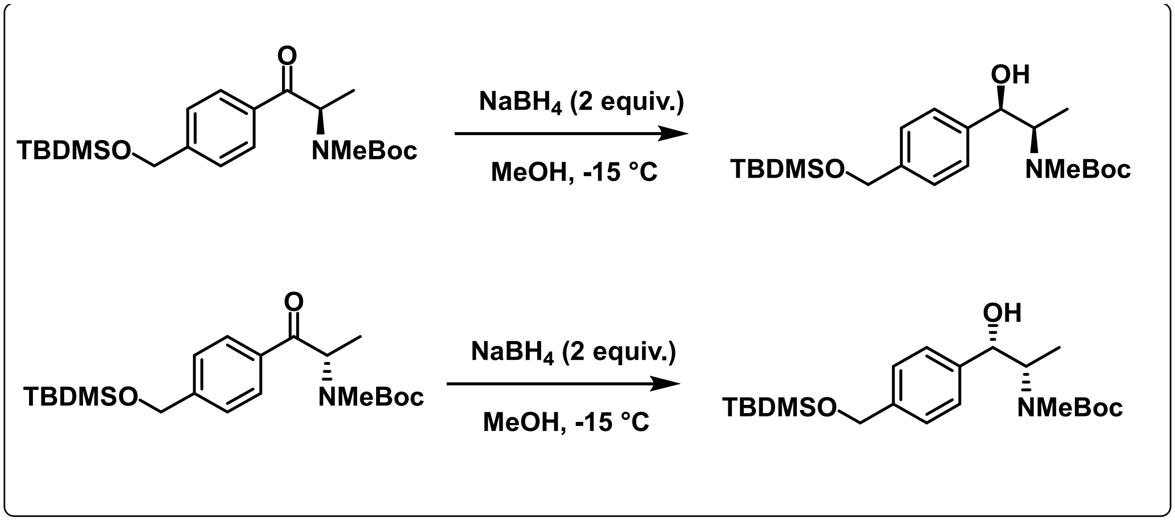

The compound was prepared according to **General procedure D**. The desired compound was obtained after purification (LP/EtOAc 4/1, 10 g $SiO_2$) as colorless viscous oil in **94%** (*R,R*), resp. **95%** (*S,S*) yield.

**$^1$H-NMR (400 MHz, $CDCl_3$)**: δ 0.09 (s, 6H), 0.93 (s, 9H), 1.02 (brs, 3H), 1.47 (s, 9H), 2.72 (brs, 3H), 4.01 – 4.29 (m, 1H), 4.55 (d, *J* = 8.3 Hz, 1H), 4.73 (s, 2H), 7.30 (s, 4H); **$^{13}$C-NMR (101 MHz, $CDCl_3$)**: δ -5.1, 14.8, 18.6, 26.1, 28.6, 31.4, 58.7, 64.9, 76.7, 80.2, 126.3, 126.7, 141.1, 157.8; (*R,R*): $\alpha_D^{20}$ = -58.7.1° (*c* 0.64, DCM), >94% ee, (*S,S*): $\alpha_D^{20}$ = +62.4° (*c* 0.6, DCM), 99% ee; HPLC method: Hexane/IPA = 99.7/0.3, 1mL/min, 25 °C, 40 min, IB; HR-MS-ESI: Calc.[M+Na]: 432.2541**,** Found [M+Na]: 432.2534 (Diff.: 1.41 ppm).

**(1*R*,2*R*)/(1*S*,2*S*)-*tert*-Butyl-4-methyl-2-oxo-5-(p-tolyl)oxazolidine-3-carboxylate (16)**

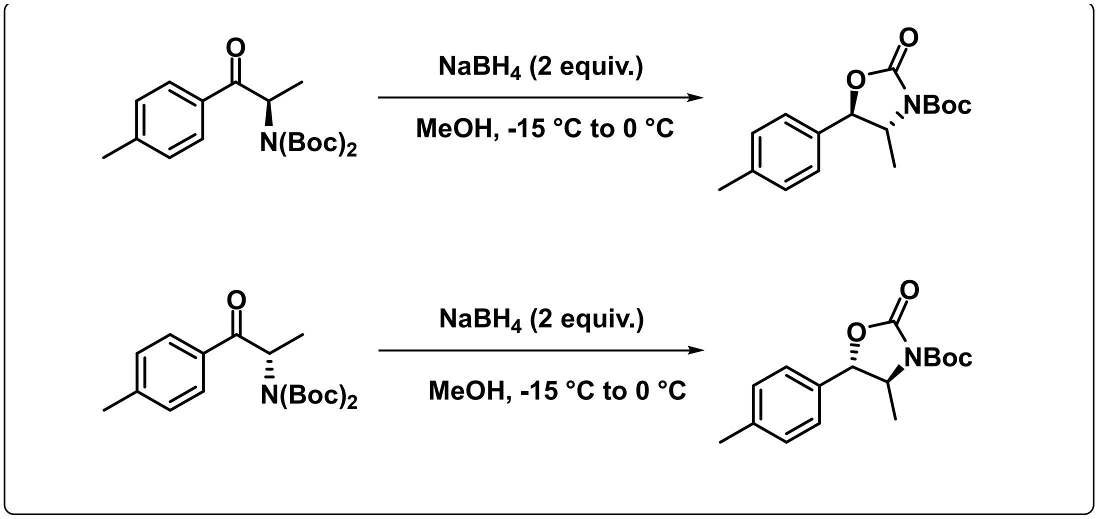


The compound was prepared according to **General procedure D** (reaction scale: 2.5 mmol). The desired compound was obtained after purification (LP/EtOAc 15/1, 35 g $SiO_2$) as colorless solid in **63%** (*R,R*), resp. **69%** (*S,S*) yield.

**$^1$H-NMR (400 MHz, $CDCl_3$)**: δ 1.50 – 1.57 (m, 12H), 2.36 (s, 3H), 4.14 (dd, *J* = 6.2, 5.0 Hz, 1H), 4.94 (d, *J* = 5.0 Hz, 1H), 7.21 (s, 4H), **$^{13}$C-NMR (101 MHz, $CDCl_3$)**: δ 19.8, 21.3, 28.1, 59.1, 81.2, 84.0, 125.6, 129.8, 134.6, 139.3, 149.5, 152.0; m.p. 78 – 79 °C; (*R,R*): $\alpha_D^{20}$ = +23.1° (*c* 0.41, MeOH), 90% ee, (*S,S*): $\alpha_D^{20}$ = -26.5° (*c* 0.62, MeOH), 93% ee; HPLC method: Hexane/IPA

= 90/10, 1mL/min, 25 °C, 30 min, AS-H; HR-MS-ESI: Calc.[M+Na]: 314.1363**,** Found [M+Na]: 314.1372 (Diff.: -2.96 ppm)

**(1*R*,2*R*)/(1*S*,2*S*)-*tert*-Butyl-5-(4-(((tertbutyldimethylsilyl)oxy)methyl)phenyl)-4-methyl-2-oxooxazolidine-3-carboxylate (17)**

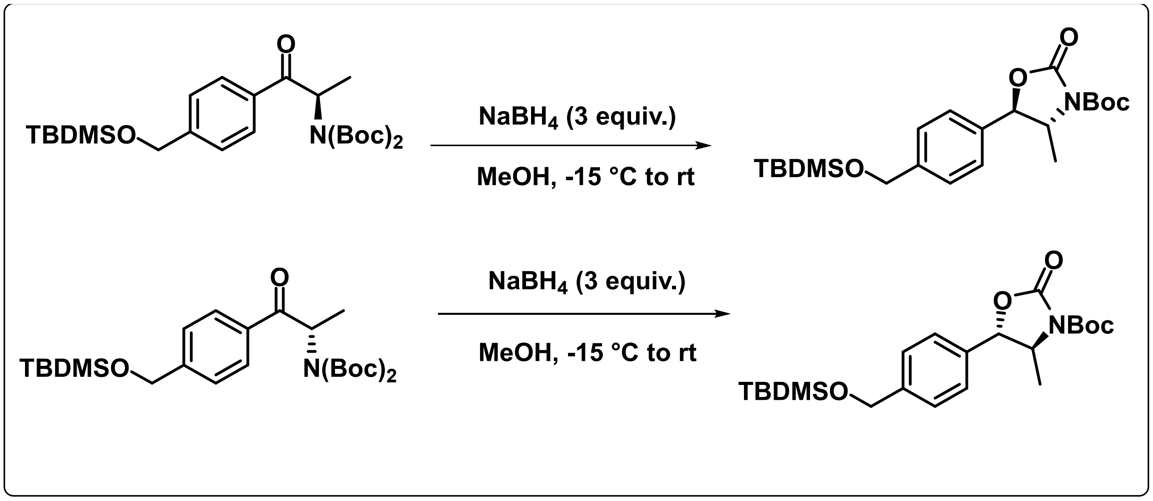


The compound was prepared according to **General procedure D** (reaction scale: 0.8 mmol). The desired compound was obtained after purification (LP/EtOAc 10/1, 25 g $SiO_2$) as colorless solid in **73%** (*R,R*), resp. **67%** (*S,S*) yield.

**$^{1}$H-NMR (400 MHz, $CDCl_3$)**: δ 0.10 (s, 6H), 0.94 (s, 9H), 1.37 – 1.65 (m, 12H), 4.15 (qd, J = 6.2, 4.9 Hz, 1H), 4.75 (s, 2H), 4.97 (d, J = 4.9 Hz, 1H), 7.29 (d, J = 8.2 Hz, 2H), 7.37 (d, J = 7.9 Hz, 2H), **$^{13}$C-NMR (101 MHz, $CDCl_3$)**: δ -5.1, 18.6, 19.9, 26.1, 28.1, 59.1, 64.6, 81.1, 84.1, 125.6, 126.7, 136.2, 142.9, 149.5, 152.0; m.p. 101 – 102 °C; (*R,R*): $\alpha_D^{20}$ = +20.1° (*c* 0.1, DCM)**,** 98% ee, (*S,S*): $\alpha_D^{20}$ = -19.2° (*c* 0.96, DCM), 97% ee; HPLC method: Heptane/IPA = 95/5, 1mL/min, 25 °C, 60 min, AS-H; HR-MS-ESI: Calc.[M+Na]: 444.2177**,** Found [M+Na]: 444.2178 (Diff.: -0.23 ppm)

**(1*R*,2*S*)/(1*S*,2*R*)-*tert*-Butyl (1-hydroxy-1-(p-tolyl)propan-2-yl)carbamate (18)**

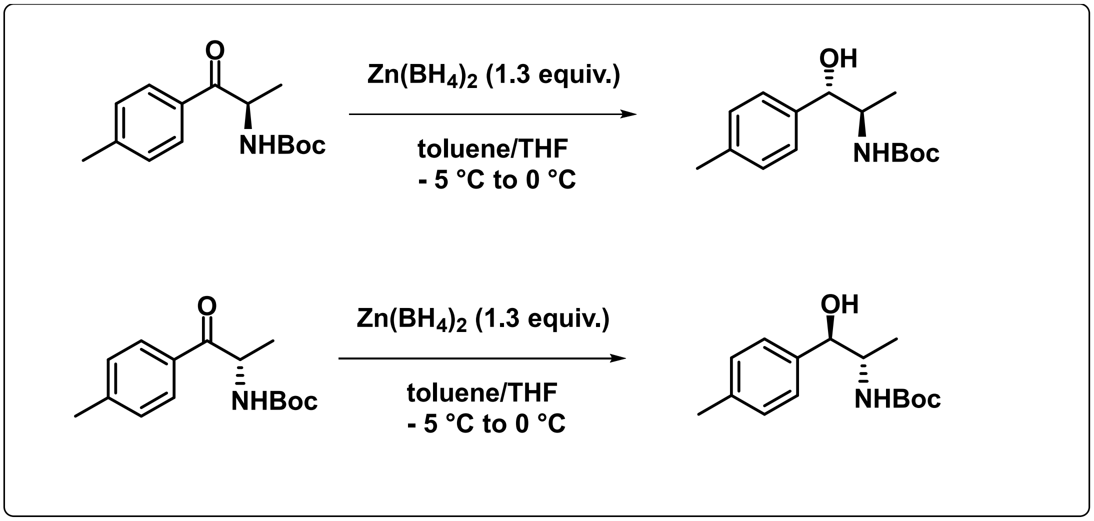


The compound was prepared according to **General procedure E** (reaction scale: 0.7 mmol). The desired compound was obtained after purification (LP/EtOAc 15/1 → 6/1**,** 25 g $SiO_2$) as colorless solid in **58%** (*R,S*), resp. **64%** (*S,R*) yield.

**$^1$H-NMR (400 MHz, $CDCl_3$)**: δ 0.97 (d, *J* = 6.9 Hz, 3H), 1.45 (s, 9H), 2.34 (s, 3H), 3.03 (brs, 1H), 3.97 (brs, 1H), 4.68 (s, 1H), 4.80 (d, *J* = 2.9 Hz, 1H), 7.15 (d, *J* = 8.0 Hz, 2H), 7.22 (d, *J* = 8.0 Hz, 2H) ppm, **$^{13}$C-NMR (101 MHz, $CDCl_3$)**: δ 15.0, 21.2, 28.5, 52.1, 79.8, 126.4, 128.9, 137.2, 137.9, 156.5; m.p. 130 – 131 °C; (*R,S*): $\alpha_D^{20}$ = -42.1° (*c* 0.11, DCM), >99% ee, (*S,R*): $\alpha_D^{20}$ = +48.5° (*c* 0.98, DCM), 99% ee; HPLC method: Hexane/EtOH = 99/1, 1mL/min, 25 °C, 60 min, AS-H.

**(1*R*,2*S*)/(1*S*,2*R*)-Ethyl(1-(4-(((*tert*-butyldimethylsilyl)oxy)methyl)phenyl)-1-hydroxypropan-2-yl)carbamate (19)**

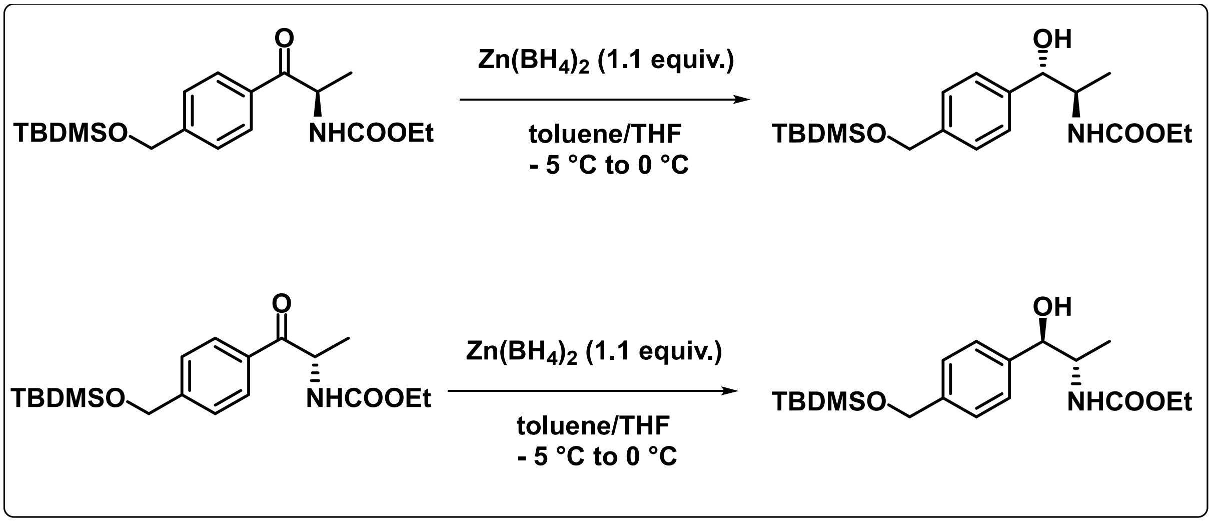


The compound was prepared according to **General procedure E** (reaction scale: 1.5 mmol). The desired compound was obtained after purification (LP/EtOAc 4/1**,** 25 g $SiO_2$) as colorless oil in **83%** (*R,S*), resp. **74%** (*S,R*) yield.

**$^1$H-NMR (400 MHz, $CDCl_3$)**: δ 0.00 (s, 6H), 0.84 (s, 9H), 0.89 (d, *J* = 6.9 Hz, 3H), 1.16 (t, *J* = 7.1 Hz, 3H), 2.46 (brs, 1H), 3.93 (brs, 1H), 4.03 (q, *J* = 7.1 Hz, 2H), 4.64 (s, 2H), 4.72 (d, *J* = 8.4 Hz, 1H), 4.77 (d, *J* = 3.1 Hz, 1H), 7.21 (s, 4H), **$^{13}$C-NMR (101 MHz, $CDCl_3$)**: δ -5.1, 14.3, 14.8, 18.6, 26.1, 52.4, 61.2, 64.9, 76.5, 126.1, 126.3, 139.5, 141.0, 157.0; (*R,S*): $\alpha_D^{20}$ = -43.1° (*c* 0.75, DCM), 99% ee, (*S,R*): $\alpha_D^{20}$ = +45.1° (*c* 0.57, DCM), 99% ee; HPLC method: Hexane/EtOH = 99/1, 1mL/min, 25 °C, 60 min, AS-H, HR-MS-ESI: Calc.[M+H]: 368.2252**,** Found [M+H]: 368.2254 (Diff.: -0.57 ppm).

**(1*R*,2*S*)/(1*S*,2*R*)-*tert*-Butyl (1-(4-(((*tert*-butyldimethylsilyl)oxy)methyl)phenyl)-1-hydroxypropan-2-yl)carbamate (20)**

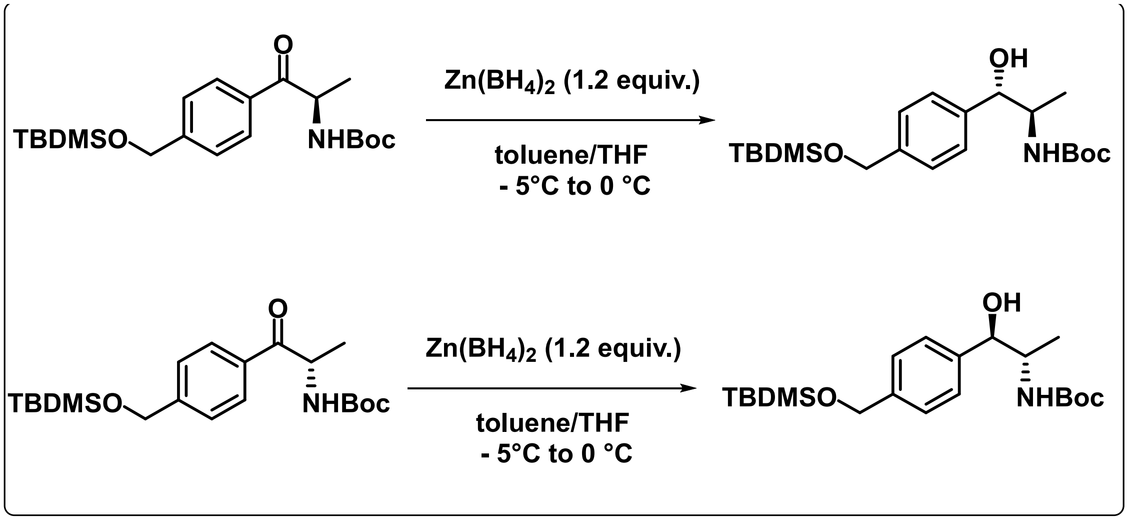


The compound was prepared according to **General procedure E** (reaction scale: 0.6 mmol). The desired compound was obtained after purification (LP/EtOAc 15/1 → 10/1 → 4/1**,** 15 g $SiO_2$) as colorless oil in **80%** (*R,S*), resp. **64%** (*S,R*) yield.

**$^1$H-NMR (400 MHz, $CDCl_3$)**: δ 0.10 (s, 6H), 0.94 (s, 9H), 0.98 (d, *J* = 6.9 Hz, 3H), 1.46 (s, 9H), 4.00 (brs, 1H), 4.57 (brs, 1H), 4.73 (s, 2H), 4.84 (d, *J* = 2.9 Hz, 1H), 7.30 (s, 4H), **$^{13}$C-NMR (101 MHz, $CDCl_3$)**: δ -5.2, 15.1, 18.6, 26.1, 28.5, 52.2, 64.9, 77.0, 77.4, 79.9, 126.0, 126.4, 139.5, 140.9, 156.6; (*R,S*): $\alpha_D^{20}$= +36.4° (*c* 1, DCM), >99% ee, (*S,R*): $\alpha_D^{20}$= -34.1° (*c* 1.4, DCM), >99% ee; HPLC method: Hexane/EtOH = 99/1, 1mL/min, 25 °C, 60 min, AS-H, HR-MS-ESI: Calc. [M+H]: 396.2565**,** Found [M+H]: 396.2553 (Diff.: 3.9 ppm).

**rac-*tert*-Butyl (1-(4-(hydroxymethyl)phenyl)-1-oxopropan-2-yl)(methyl)carbamate (21)**

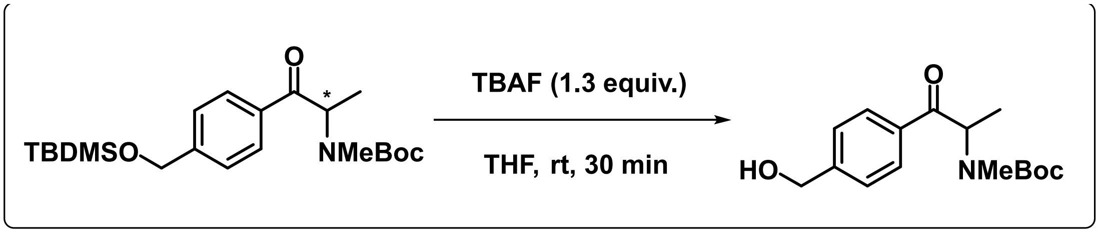

To a solution of *tert*-butyl (1-(4-(((tert-butyldimethylsilyl)oxy)methyl)phenyl)-1-oxopropan-2-yl)(methyl)carbamate **(13)** (400 mg, 0.1 mmol, 1 equiv.) in anhydrous THF (10 mL) TBAF (1 M in THF, 1.3 mL, 1.3 mmol, 1.3 equiv.) was added under argon at rt. After stirring for 30 min at this temperature, the reaction mixture was partitioned between EtOAc (10 mL) and satd. $NaHCO_3$ (10 mL). The aqueous layer was extracted with EtOAc (3 x 20 mL) and combined organic layers were combined, dried over anh. $MgSO_4$ and concentrated *in vacuo*. The desired compound was obtained after purification (LP/EtOAc 2/1, 50 g $SiO_2$) as pale yellow oil in **94%** yield. Remark: in the course of reaction it came to the expected racemization of the substrate in presence of TBAF.

**$^1$H-NMR (400 MHz, $CDCl_3$)**: Mixture of rotamers ≈ 60:40: δ 1.01 – 1.62 (m, 12H), 2.61 (s, 1.8H), 2.77 (s, 1.2H), 4.76 (s, 2H), 5.18 (q, J = 6.9 Hz, 0.4H), 5.66 (q, *J* = 6.9 Hz, 0.6H), 7.42 (d, *J* = 7.7 Hz, 2H), 7.85 – 8.03 (m, 2H); **$^{13}$C-NMR (101 MHz, $CDCl_3$**: δ 13.4, 13.8, 28.5, 29.7, 30.8, 54.8, 57.1, 64.8, 80.4, 80.9, 126.8, 128.6, 128.9, 134.8, 135.0, 146.4, 154.8, 155.7, 199.5 ppm; HR-MS-ESI: Calc.[M+Na]: 316.1519, Found [M+Na]: 316.1522 (Diff.: -0.7 ppm).

**rac-4-(Methylalanyl)benzoic acid hydrochloride (22)**

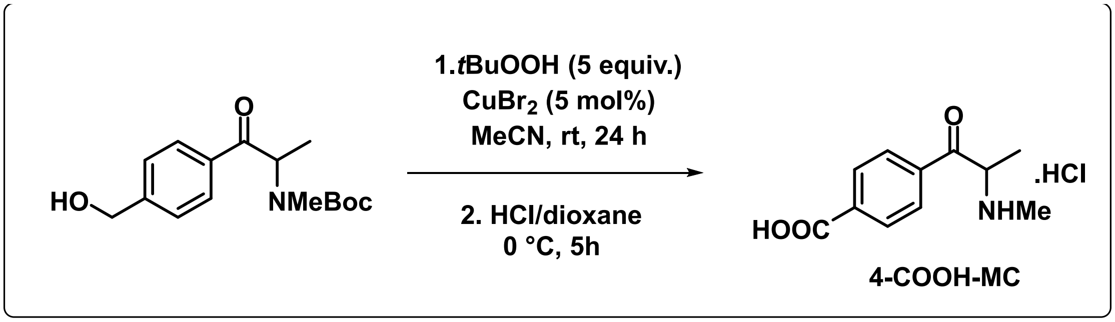


To a stirring solution of **(23)** (50 mg, 0.17 mmol, 1 equiv.) and $CuBr_2$ (2 mg, 0.0085 mmol, 0.05 equiv.) in MeCN (0.43 mL) *tert*-BuOOH (0.1 mL, 0.85 mmol, 5 equiv., 70% in water) was slowly added. The reaction was stirred for 24 hours until TLC indicated full conversion of the alcohol. The volatiles were then removed *in vacuo* and the residue was treated with satd. $NaHCO_3$ (3 mL) and extracted with EtOAc (3 mL). The aqueous phase was then acidified with 1M HCl to pH 2 and extracted with EtOAc (3 x 5 mL). The combined organic phases were then dried over anh. $MgSO_4$ and concentrated *in vacuo*. The product was carried without further purification to the next step.

In an 8 mL screw-cap vial the crude carboxylic acid (20 mg, 0.07 mmol, 1 equiv.) from the previous step was dissolved in HPLC grade dioxane (0.3 mL), cooled to 0 °C, treated with 6M HCl in $H_2O$/dioxane 4:1 (0.35 mL) and stirred for 5 h at 0 °C. The reaction mixture was extracted with $Et_2O$ (2 x 2 mL) and the aqueous layer was evaporated in a stream of pressurized air. The residue was taken up in deionized water, filtered and lyophilized. The desired compound was obtained as colorless solid in **58%** yield.

**$^1$H-NMR (400 MHz, $D_2O$)**: δ 1.62 (d, *J* = 7.2 Hz, 3H), 2.84 (s, 3H), 5.15 (q, *J* = 7.3 Hz, 1H), 8.09 (d, *J* = 8.2 Hz, 2H), 8.16 (d, *J* = 8.2 Hz); **$^{13}$C-NMR (101 MHz, $D_2O$)**: δ 14.9, 30.9, 59.9, 129.0, 130.2, 135.5, 135.8, 169.3, 197.1 ppm; m.p. decomposition > 210 °C; HR-MS-ESI: Calc.[M+H]: 208.0968, Found [M+H]: 208.0979 (Diff.: -5.33 ppm).

**(*R*)/(*S*)-2-(Methylamino)-1-(p-tolyl)propan-1-one hydrochloride, mephedrone, 4-MMC (23)**

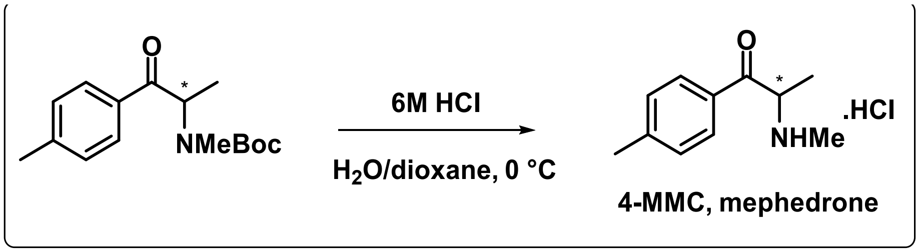


The compound was prepared according to **General procedure F** (reaction scale: 0.4 mmol). The desired compound was obtained as colorless solid in **97%** (*R*), resp. **97%** (*S*) yield.

**$^1$H-NMR (400 MHz, $D_2O$)**: δ 1.61 (d, *J* = 7.3 Hz, 3H), 2.45 (s, 3H), 2.81 (s, 3H), 5.08 (q, *J* = 7.3 Hz, 1H), 7.46 (d, *J* = 8.1 Hz, 2H), 7.92 (d, *J* = 8.2 Hz, 2H); **$^{13}$C-NMR (101 MHz, $D_2O$)**: δ 15.5, 21.0, 31.0, 59.6, 129.0, 129.6, 129.9, 147.4, 197.1 ppm; m.p. decomposition >180 °C, (*R*): $\alpha_D^{20}$ = +40.3° (*c* 0.7, $H_2O$), 97% ee, (*S*): $\alpha_D^{20}$ = -41.5° (*c* 0.8, $H_2O$), 98% ee; HPLC method: Heptane/IPA = 97/3 + 0.1% DEA, 1mL/min, 25 °C, AS-H ($t_S$= 8.2 min, $t_R$= 15.1 min).

**(*R*)/(*S*)-2-amino-1-(p-tolyl)propan-1-one, nor-mephedrone, 4-MC (24)**

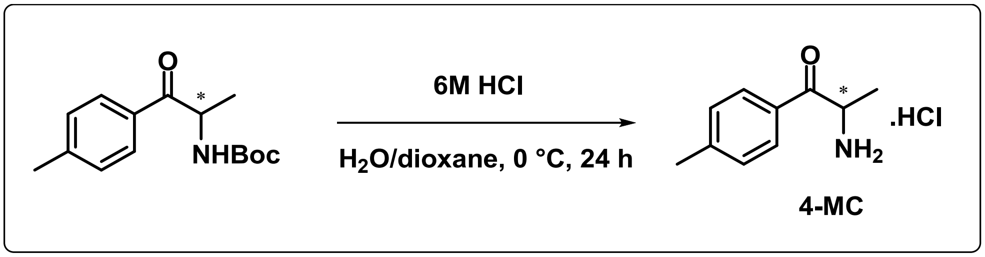


The compound was prepared according to **General procedure F** (reaction scale: 3 mmol). The desired compound was obtained as colorless solid in **92%** (*R*), resp. **83%** (*S*) yield.

**$^1$H-NMR (400 MHz, $D_2O$)**: δ 1.60 (d, J = 7.3 Hz, 3H), 2.44 (s, 3H), 5.17 (q, *J* = 7.3 Hz, 1H), 7.44 (d, *J* = 8.0 Hz, 2H), 7.92 (d, *J* = 8.2 Hz, 2H); **$^{13}$C-NMR (101 MHz, $D_2O$)**: δ 16.8, 20.9, 51.8, 129.0, 129.6, 129.8, 147.1, 197.6; m.p. decomposition >180 °C, (*R*): $\alpha_D^{20}$ = +40.3° (*c* 0.7, $H_2O$), >99% ee, (*S*): $\alpha_D^{20}$ = -42.5° (*c* 0.8, $H_2O$), >99% ee; HPLC method: Heptane/IPA = 96.9/3 + 0.1% DEA, 1mL/min, 25 °C, AS-H ($t_S$= 23.9 min, $t_R$= 28.7 min).

**(*R*)/(*S*)-2-Amino-1-(4-(hydroxymethyl)phenyl)propan-1-one, 4-OH-MC (25)**

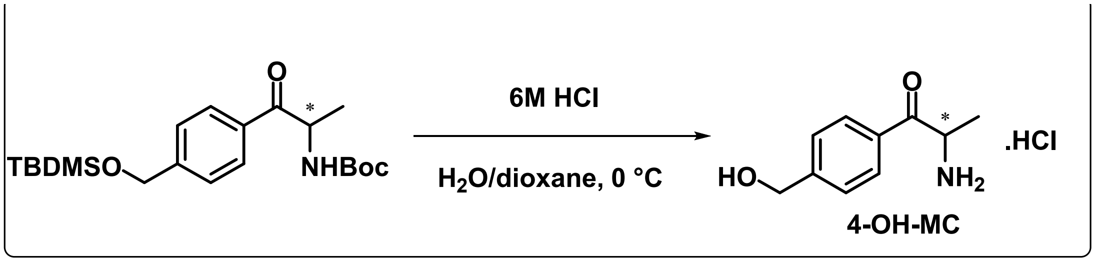


The compound was prepared according to **General procedure F** (reaction scale: 0.3 mmol). The desired compound was obtained as colorless solid in **64%** (*R*), resp. **89%** (*S*) yield.

**$^1$H-NMR (400 MHz, $D_2O$)**: δ 1.53 - 1.60 (m, *J* = 7.33 Hz), 4.75 (s, 2H), 5.20 (q, *J* = 7.31 Hz, 1H), 7.58 (d, *J* = 8.18 Hz, 2H), 8.02 (d, *J* = 8.31 Hz, 2H); **$^{13}$C-NMR (101 MHz, $D_2O$)**: δ 16.7, 51.9, 63.1, 127.4, 129.2, 131.4, 147.9, 197.6 ppm; m.p. 185 – 188 °C, (*R*): $\alpha_D^{20}$ = -33.8° (*c* 0.86, MeOH), 91% ee, (*S*): $\alpha_D^{20}$= +37.9° (*c* 0.71, MeOH), 92% ee; HPLC method: Hexane/EtOH = 89.9/10 + 0.1% DEA, 1mL/min, 25 °C, AS-H ($t_S$= 22.9 min, $t_R$= 20.1 min); HR-MS-ESI: Calc. [M+H]: 180.1024**,** Found [M+H]: 180.1019 (Diff.: -2.56 ppm).

**(1*R*,2*S*)/(1*S*,2*R*)-2-Amino-1-(p-tolyl)propan-1-ol hydrochloride, *anti*-dihydro-4-MC (26)**

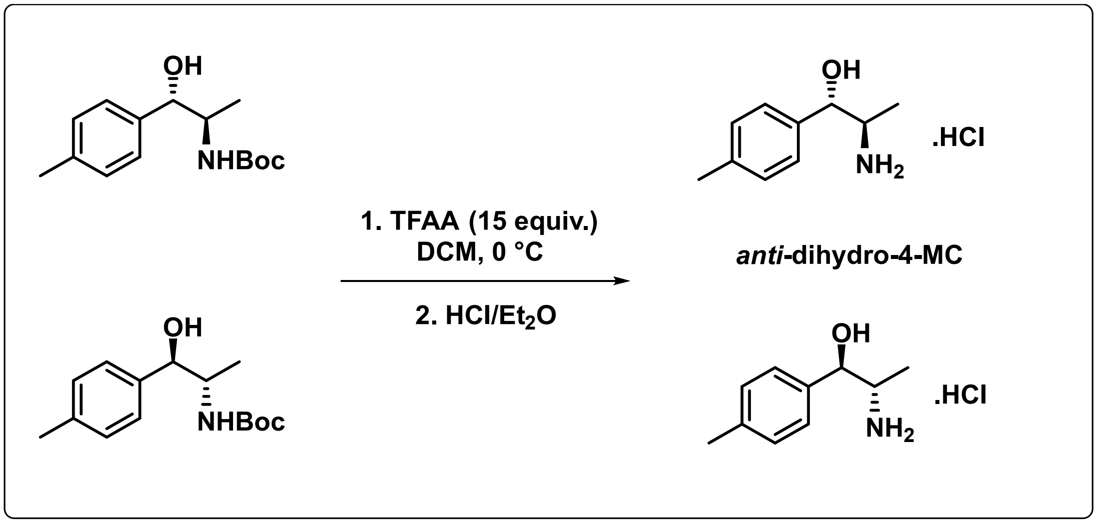


The compound was prepared according to **General procedure G** (reaction scale: 0.2 mmol). The desired compound was obtained as colorless solid in **70%** (1*R*,2*S*), resp. **86%** (1*S*,2*R*) yield.

**$^1$H-NMR (400 MHz, $D_2O$)**: δ 1.17 (d, *J* = 6.8 Hz, 3H), 2.33 (s, 3H), 3.71 – 3.50 (m, 1H), 4.90 (d, *J* = 4.6 Hz, 1H), 7.29 (s, 4H); **$^{13}$C-NMR (101 MHz, $D_2O$)**: δ 12.5, 20.1, 52.1, 72.9, 126.3, 129.4, 135.3, 138.8 ppm; m.p. decomposition >200 °C, (1*R*,2*S*): $\alpha_D^{20}$= -45.9° (*c* 0.26, MeOH), 99% ee, (1*S*,2*R*): $\alpha_D^{20}$ = +48.7° (*c* 0.31, MeOH), 99% ee; UPLC method: aq. NH4OAc (20 mM, pH = 4.5)/ MeOH = 94/6, 0.8mL/min, 30 °C, Cyclobond I 2000 Ac ($t_{S,R}$= 8.5 min, $t_{R,S}$= 8.2 min); HR-MS-ESI: Calc.[M+H]: 166.1226, Found [M+H]: 166.1232 (Diff.: -3.15 ppm).

**(1*R*,2*R*)/(1*S*,2*S*)-1-(4-(Hydroxymethyl)phenyl)-2-(methylamino)propan-1-ol hydrochloride, *syn*-dihydro-4-OH-MMC (27)**

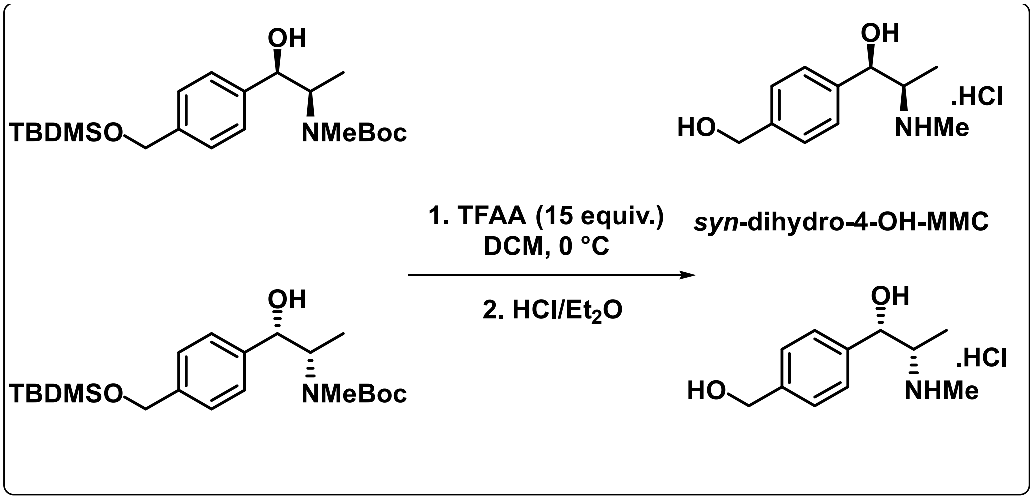


The compound was prepared according to **General procedure G** (reaction scale: 0.3 mmol). The desired compound was obtained as colorless solid in **61%** (1*R*,2*R*), resp. **65%** (1*S*,2*S*) yield.

**$^1$H-NMR (400 MHz, $D_2O$)**: δ 1.12 (d, *J* = 6.7 Hz, 3H), 2.77 (s, 3H), 3.55 (dq, *J* = 9.0, 6.8 Hz, 1H), 4.66 (s, 2H), 4.70 (d, *J* = 9.1 Hz, 1H), 7.45 (s, 4H); **$^{13}$C-NMR (101 MHz, $D_2O$)**: δ 11.6, 29.7, 59.8, 63.4, 73.8, 127.3, 127.9, 138.6, 141.0 ppm; m.p. decomposition >210 °C, (1*R*,2*R*): $\alpha_D^{20}$= -53.0° (*c* 0.53, MeOH), 95% ee, (1*S*,2*S*): $\alpha_D^{20}$ = +48.1° (*c* 0.49, MeOH), >99% ee; UPLC method: aq. $(NH_4)_2HPO_4$ (20 mM, pH = 4.2), 0.1mL/min, 10 °C, Cyclobond I 2000 Ac ($t_{S,S}$= 58.1 min, $t_{R,R}$= 60.0 min); HR-MS-ESI: Calc.[M+H]: 196.1332**,** Found [M+H]: 196.1336 (Diff.: -1.95 ppm).

**(1*R*,2*R*)/(1*S*,2*S*)-2-amino-1-(p-tolyl)propan-1-ol hydrochloride, *syn*-dihydro-4-MC (28)**

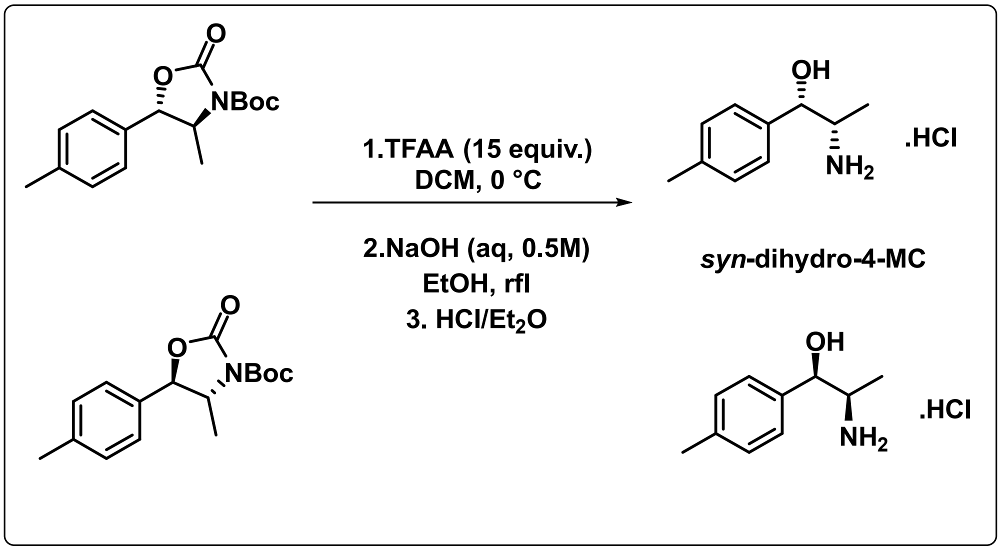


Boc-protected oxazolidines (1 equiv.) were dissolved in dry DCM (c=0.1M) under argon atmosphere, cooled to 0 °C and trifluoroacetic acid (15 equiv.) was added dropwise. The reaction mixture was stirred for 3 h at 0 °C and after TLC indicated full conversion the volatiles were removed under flow of pressurized air. The oily residue was taken up in aq. NaOH solution (1M saturated with NaCl) in an amount to create approximately 0.1M solution. It was then extracted multiple times with EtOAc, the organic phases were combined, dried over anh. $MgSO_4$, filtered and evaporated under reduced pressure.

The crude deprotected carbamate (1 equiv.) was dissolved in aqueous NaOH (0.5M) and EtOH (ratio 2:1, c= 0.04 M) and heated to reflux until TLC showed full conversion of starting material. The reaction was cooled to room temperature and extracted twice with EtOAc. The combined organic phases were dried over anh. $MgSO_4$ and evaporated *in vacuo*. The solid residue was taken up in diethyl ether (c=0.1M) and treated with excess of ethereal hydrochloric acid, whereas colorless solid precipitated. The organic phase was evaporated in a stream of pressurized air, washed twice with diethyl ether, dried *in vacuo*, dissolved in deionised water, filtered and lyophilized. The desired compounds were obtained as colorless solid in **76%** (1*R*,2*R*), resp. **88%** (1*S*,2*S*) yield.

**$^1$H-NMR (400 MHz, $D_2O$)**: δ 1.14 (d, *J* = 6.7 Hz, 3H), 2.36 (s, 3H), 3.60 (dq, *J* = 8.6, 6.8 Hz), 4.62 (d, *J* = 8.6 Hz, 1H), 7.11 – 7.53 (m, 4H); **$^{13}$C-NMR (101 MHz, $D_2O$)**: δ 14.5, 20.2, 52.7, 74.7,

126.9, 129.5, 136.3, 139.2 ppm; m.p. 219 – 221 °C, (1*R*,2*R*): $\alpha_D^{20}$ = -38.2° (*c* 0.75, MeOH), 96% ee, (1*S*,2*S*): $\alpha_D^{20}$ = +37.5° (*c* 0.89, MeOH), 90% ee; HPLC method: Heptane/IPA = 99.4/0.5 + 0.1% DEA, 1mL/min, 25 °C, AS-H ($t_{S,S}$= 86.5 min, $t_{R,R}$= 98.7 min); HR-MS-ESI: Calc.[M+H]: 166.1226**,** Found [M+H]: 166.1228 (Diff.: -1.01 ppm).

**(1*R*,2*R*)/(1*S*,2*S*)-2-Amino-1-(4-(((tert-butyldimethylsilyl)oxy)methyl)phenyl)propan-1-ol, *syn*-dihydro-4-OH-MC (29)**

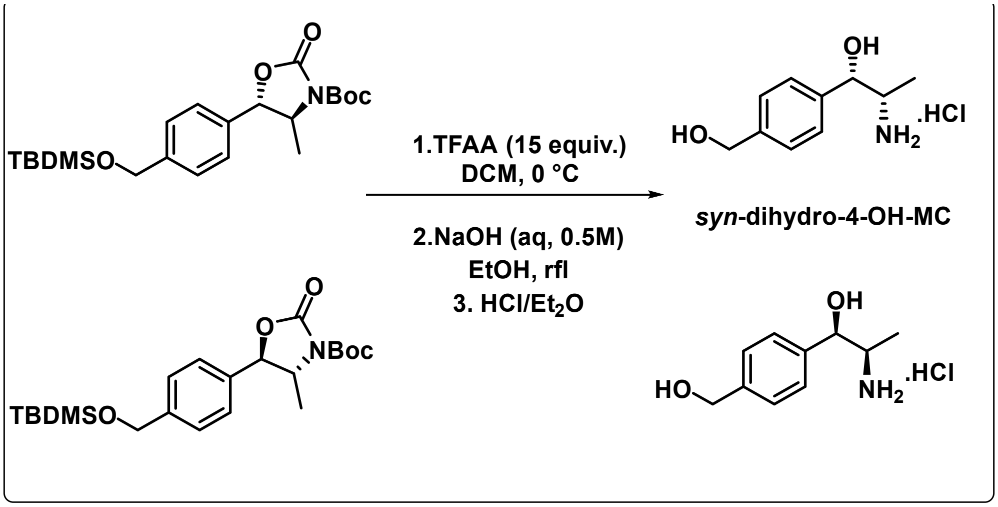


The compound was prepared according to procedure for **(28),** (reaction scale: 0.3 mmol) and obtained as colorless solid in **53%** (1*R*,2*R*), resp. **44%** (1*S*,2*S*) yield.

**$^1$H-NMR (400 MHz, MeOD)**: δ 1.11 (d, *J* = 6.8 Hz, 3H), 3.37 (qd, *J* = 8.4, 6.8 Hz, 1H), 4.51 (d, *J* = 8.6 Hz, 1H), 4.62 (s, 2H), 7.40 (s, 4H); **$^{13}$C-NMR (101 MHz, MeOD)**: δ 15.6, 54.6, 64.8, 76.2, 128.0, 128.3, 141.0, 143.3 ppm; m.p. decomposition > 200 °C, (1*R*,2*R*): $\alpha_D^{20}$ = -38.1° (*c* 0.5, MeOH), 98% ee, (1*S*,2*S*): $\alpha_D^{20}$ = +40.2° (*c* 0.54, MeOH), 97% ee; HPLC method: Heptane/IPA = 99.4/0.5 + 0.1% DEA, 1mL/min, 25 °C, AS-H[1] ; HR-MS-ESI: Calc.[M+H]: 182.1176**,** Found [M+H]: 182.1175 (Diff.: 0.18 ppm).

[1] Since it was not possible to achieve chromatographic separation of the enantiomers, based on experience with other metabolites we assumed that simultaneous change of chirality on both chiral centers would be rather improbable. Therefore given ee is extrapolated from subjecting the metabolites to separation method utilized for corresponding diastereoisomers.

**(1*R*,2*S*)/(1*S*,2*R*)-2-(Methylamino)-1-(p-tolyl)propan-1-ol hydrochloride, *anti*-dihydro-4-MMC, dihydro-MEPH (30)**

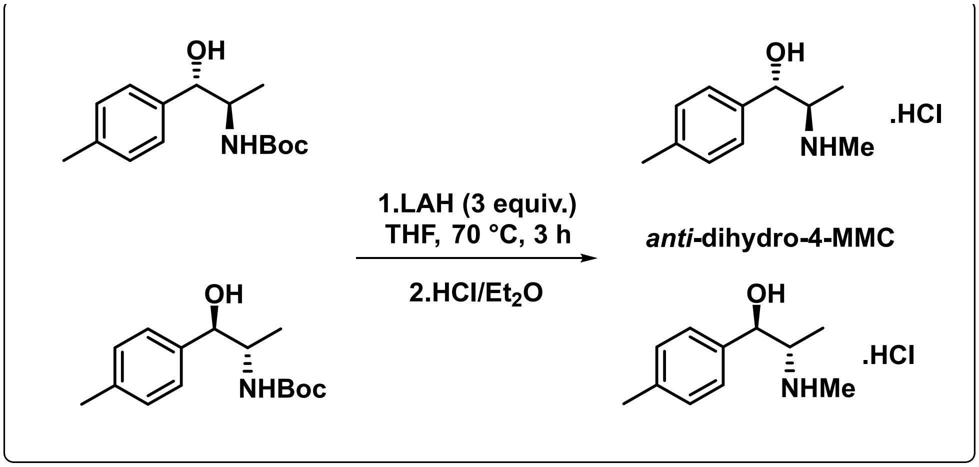


In an oven-dried 8 mL vial Boc-protected amino alcohol **(18)** (150 mg, 0.57 mmol, 1 equiv.) was dissolved in dry THF (1.2 mL) under argon and $LiAlH_4$ (64 mg, 1.7 mmol, 3 equiv.) was added in three portions. The reaction mixture was heated to 70 °C and stirred for 3 hours. The reaction mixture was cooled to 0 °C and sequentially quenched with water (60 µL) and 15 % NaOH (60 µL) and additional DCM (1 mL) was added to facilitate stirring. The colorless precipitate was let stir for 30 min at rt. The organic layer was decanted and the colorless precipitate was washed with EtOAc (2 x 2 mL), the combined organic phases were dried over anh. $MgSO_4$ and filtered, yielding light brown oil which was immediately purified via flash chromatography - $CHCl_3$/MeOH = 5/1 (+1% $Et_3N$), 3.5 g $SiO_2$.

The free base was then dissolved in dry $Et_2O$ (2 mL) and excess of ethereal HCl (2 M, 2 mL) was added dropwise and stirred for 15 min, whereas a colorless precipitate formed. It was then collected by filtration, washed with $Et_2O$ (2 x 2 mL), taken up in deionized water, filtered and lyophilized. The compound was obtained as colorless solid in **53%** (1*R*,2*S*), resp. **49%** (1*S*,2*R*) yield, whereas 25% od starting material was re-isolated.

**$^1$H-NMR (400 MHz, $D_2O$)**: δ 1.15 (d, J = 6.8 Hz, 3H), 2.36 (s, 3H), 2.78 (s, 3H), 3.48 – 3.59 (m, 1H), 5.10 (d, J = 3.7 Hz, 1H), 7.32 (s, 4H); **$^{13}$C-NMR (101 MHz, $D_2O$)**: δ 9.8, 20.1, 30.7, 59.9, 71.4, 126.1, 129.3, 135.3, 138.7 ppm; m.p. sublimation > 200 °C, (1*R*,2*S*): $\alpha_D^{20}$ = -45.5° (*c* 0.49,

MeOH), >99% ee, (1*S*,2*R*): $\alpha_D^{20}$ = +48.0° (*c* 0.61, MeOH), >99% ee; UPLC method: Heptane/IPA = 97/3 + 0.1% DEA, 1mL/min, 25 °C, AS-H ($t_{S,R}$= 10.6 min, $t_{R,S}$= 11.9 min); HR-MS-ESI: Calc.[M+H]: 182.1176**,** Found [M+H]: 182.1175 (Diff.: 0.18 ppm).

**(1*R*,2*S*)/(1*S*,2*R*)-1-(4-(Hydroxymethyl)phenyl)-2-(methylamino)propan-1-ol hydrochloride, *anti*-dihydro-4-OH-MMC (31)**

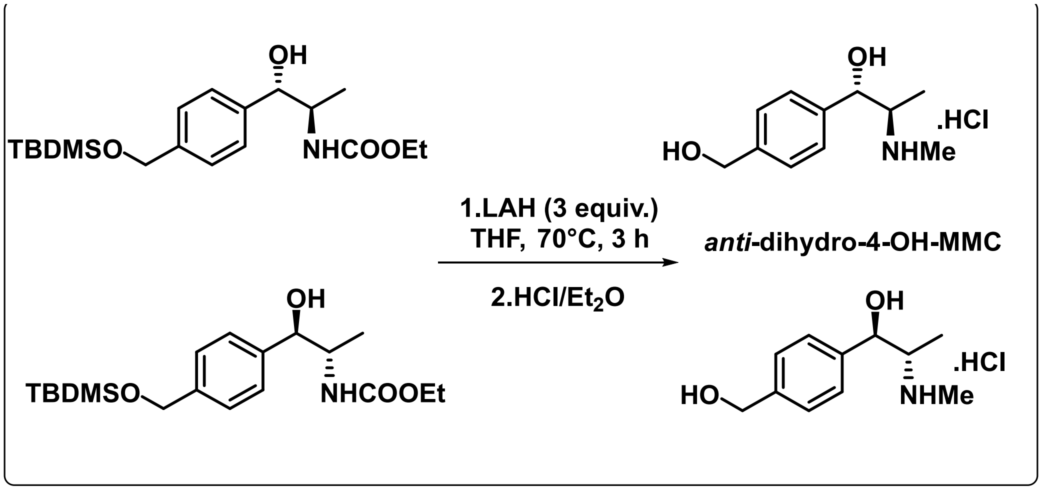


The compound was prepared according to procedure for **(30),** (reaction scale: 0.3 mmol) and obtained as colorless solid in **65%** (1*R*,2*S*), resp. **54%** (1*S*,2*R*) yield, however the crude product was subjected to treatment with ethereal HCl without flash chromatographic purification.

**$^1$H-NMR (400 MHz, $D_2O$)**: δ 1.15 (d, *J* = 6.8 Hz, 3H), 2.79 (s, 3H), 3.57 (qd, *J* = 6.8, 3.5 Hz, 1H), 4.66 (s, 2H), 5.15 (d, *J* = 3.6 Hz, 1H), 7.25 – 7.58 (m, 4H); **$^{13}$C-NMR (101 MHz, $D_2O$)**: δ 9.7, 30.7, 59.9, 63.5, 71.3, 126.3, 127.7, 137.8, 140.3 ppm; m.p. decomposition > 200 °C, (1*R*,2*S*): $\alpha_D^{20}$ = -39.4° (*c* 0.54, MeOH), >99% ee, (1*S*,2*R*): $\alpha_D^{20}$ = +40.2° (*c* 0.33, MeOH), >99% ee; UPLC method: aq. $(NH_4)_2HPO_4$ (20 mM, pH = 4.2), 0.1mL/min, 10 °C, Cyclobond I 2000 Ac[2]; HR-MS-ESI: Calc.[M+H]: 196.1332, Found [M+H]: 196.1335 (Diff.: -1.7 ppm).

[2] Since it was not possible to achieve chromatographic separation of the enantiomers, based on experience with other metabolites we assumed that simultaneous change of chirality on both chiral centers would be rather improbable. Therefore given ee is extrapolated from subjecting the metabolites to separation method utilized for corresponding diastereoisomers.

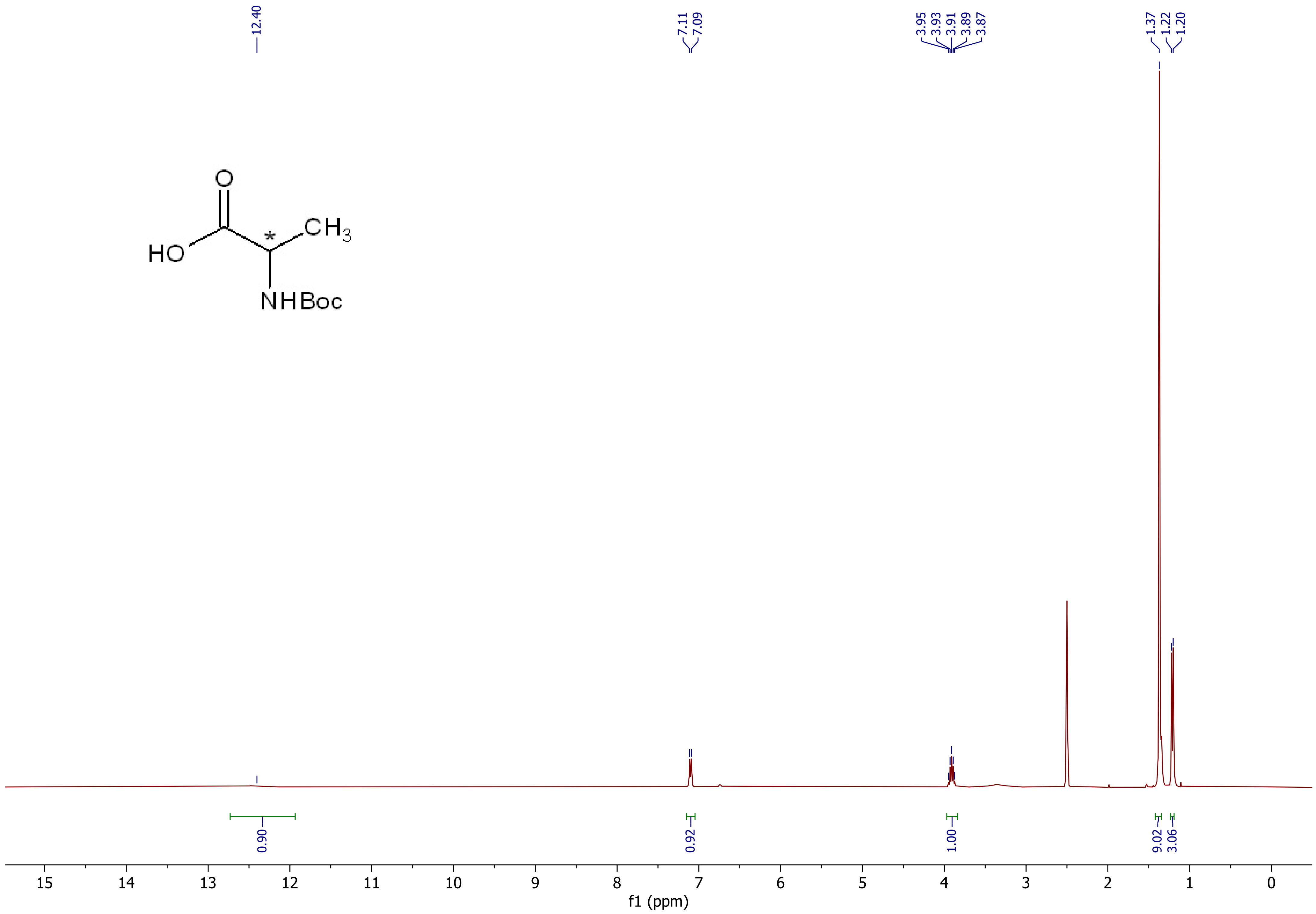

12.40
7.11
7.09
3.95
3.93
3.91
3.89
3.87
1.37
1.22
1.20
O
HO
CH3
*
NHBoc
0.90
0.92
1.00
9.02
3.06
15 14 13 12 11 10 9 8 7 6 5 4 3 2 1 0
f1 (ppm)

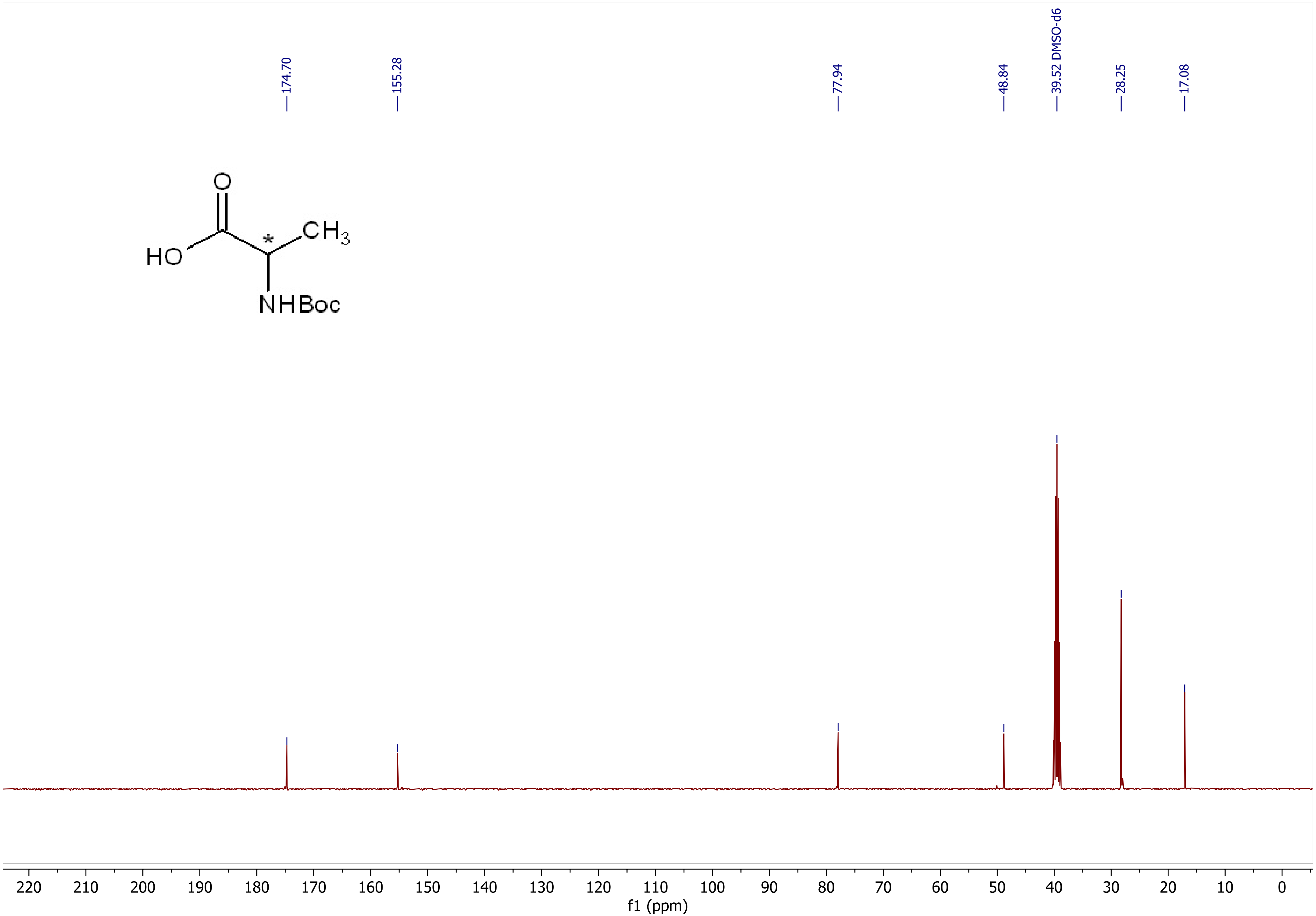

174.70
155.28
77.94
48.84
39.52 DMSO-d6
28.25
17.08
O
HO
CH3
*
NHBoc
220
210
200
190
180
170
160
150
140
130
120
110
100
90
80
70
60
50
40
30
20
10
0
f1 (ppm)

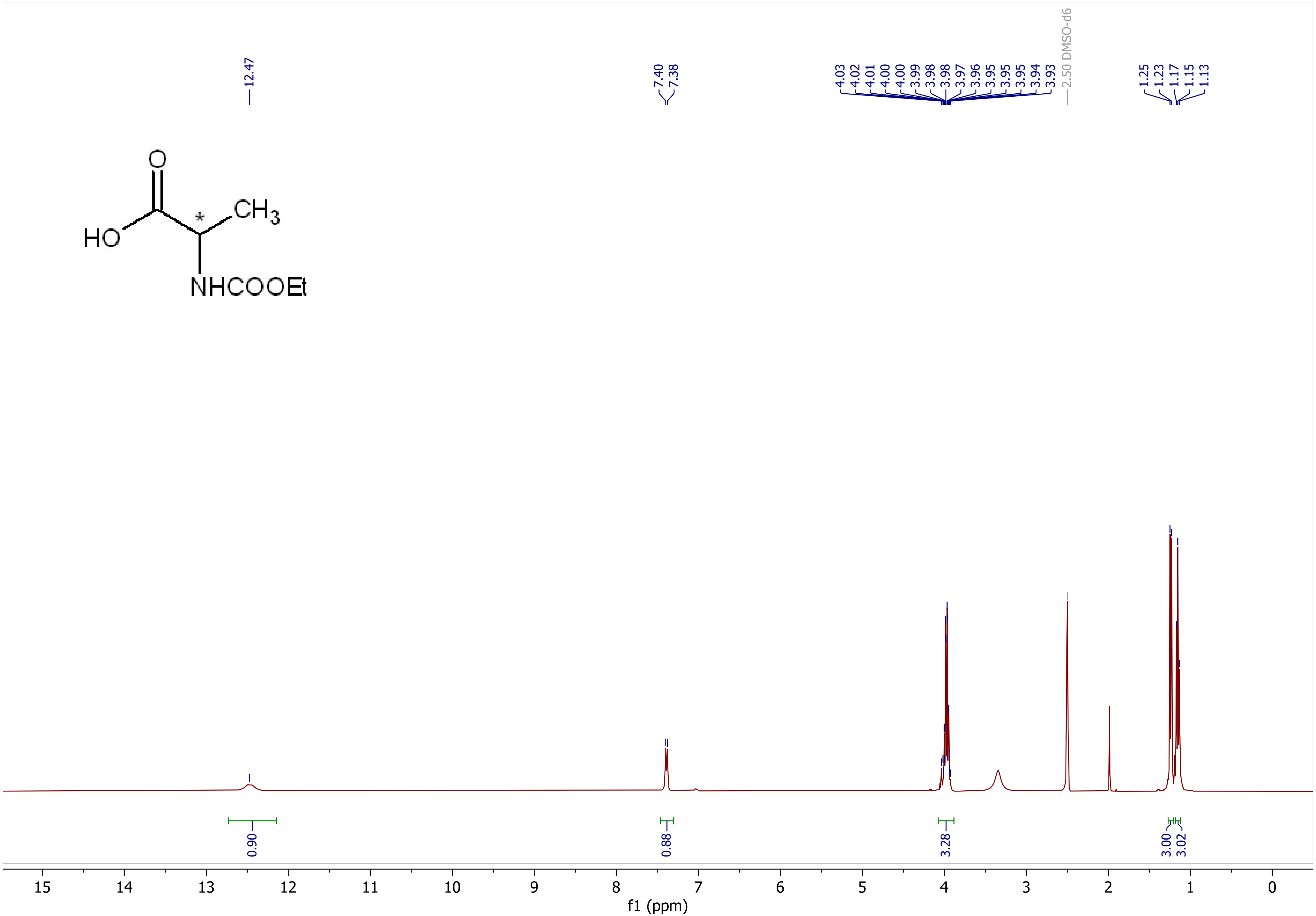

12.47
7.40
7.38
4.03
4.02
4.01
4.00
4.00
3.99
3.98
3.98
3.97
3.96
3.95
3.95
3.95
3.94
3.93
2.50 DMSO-d6
1.25
1.23
1.17
1.15
1.13
HO
O
CH3
*
NHCOOEt
0.90
0.88
3.28
3.00
3.02
15
14
13
12
11
10
9
8
7
6
5
4
3
2
1
0
f1 (ppm)

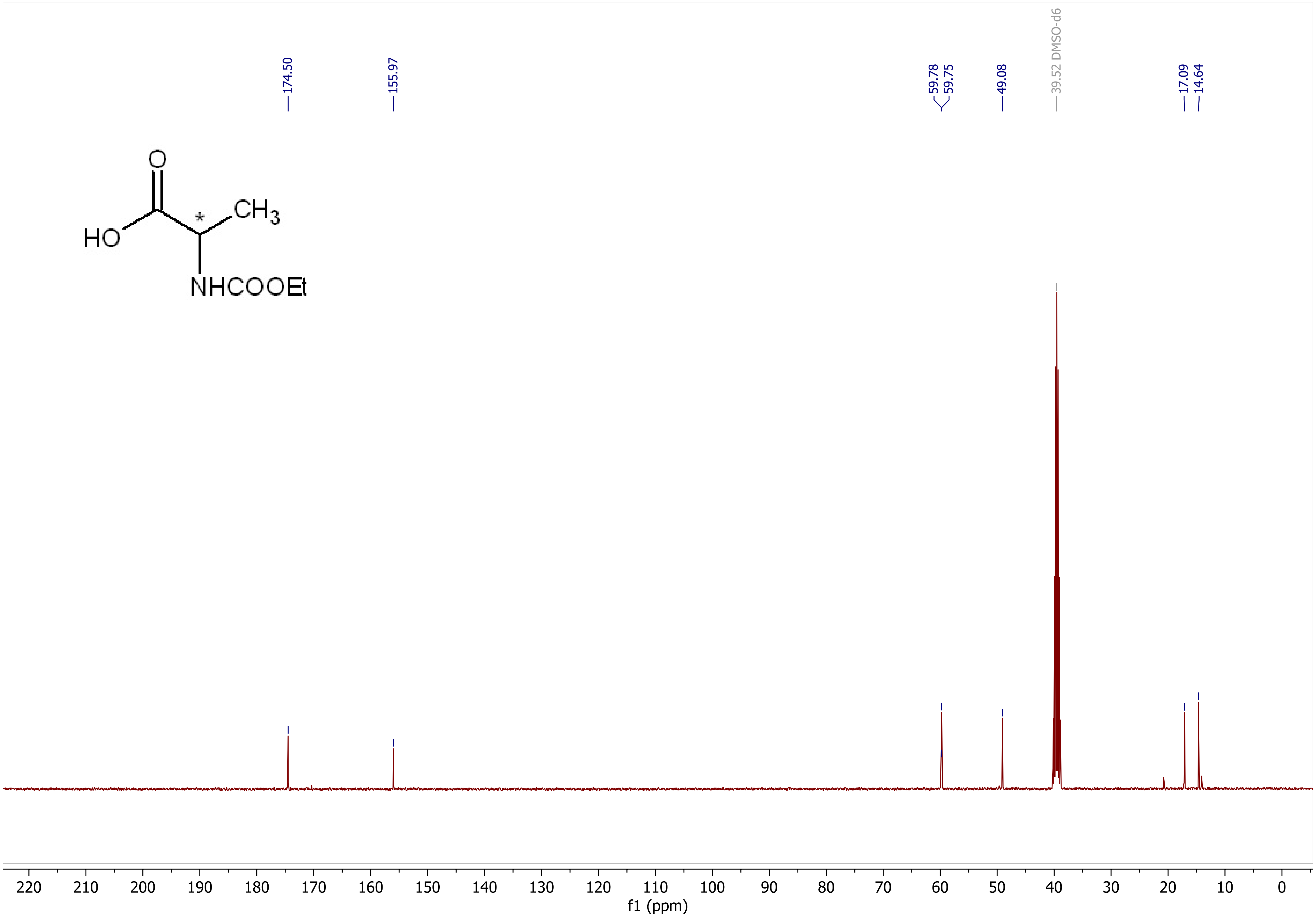

174.50
155.97
59.78
59.75
49.08
39.52 DMSO-d6
17.09
14.64
HO
O
CH3
*
NHCOOEt
220
210
200
190
180
170
160
150
140
130
120
110
100
90
80
70
60
50
40
30
20
10
0
f1 (ppm)

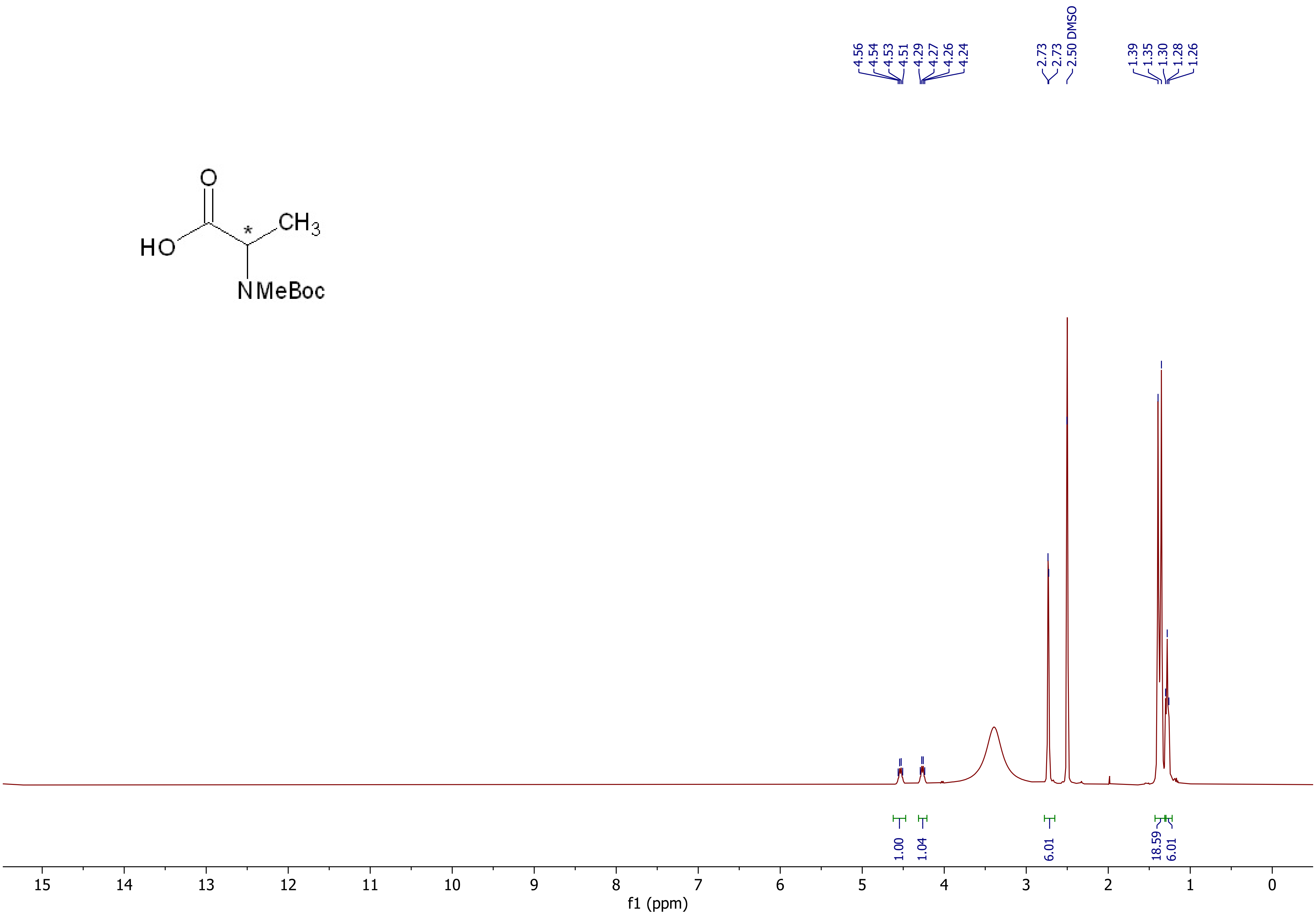
4.56
4.54
4.53
4.51
4.29
4.27
4.26
4.24
2.73
2.73
2.50 DMSO
1.39
1.35
1.30
1.28
1.26
O
HO
CH3
*
NMeBoc
1.00
1.04
6.01
18.59
6.01
15
14
13
12
11
10
9
8
7
6
5
4
3
2
1
0
f1 (ppm)

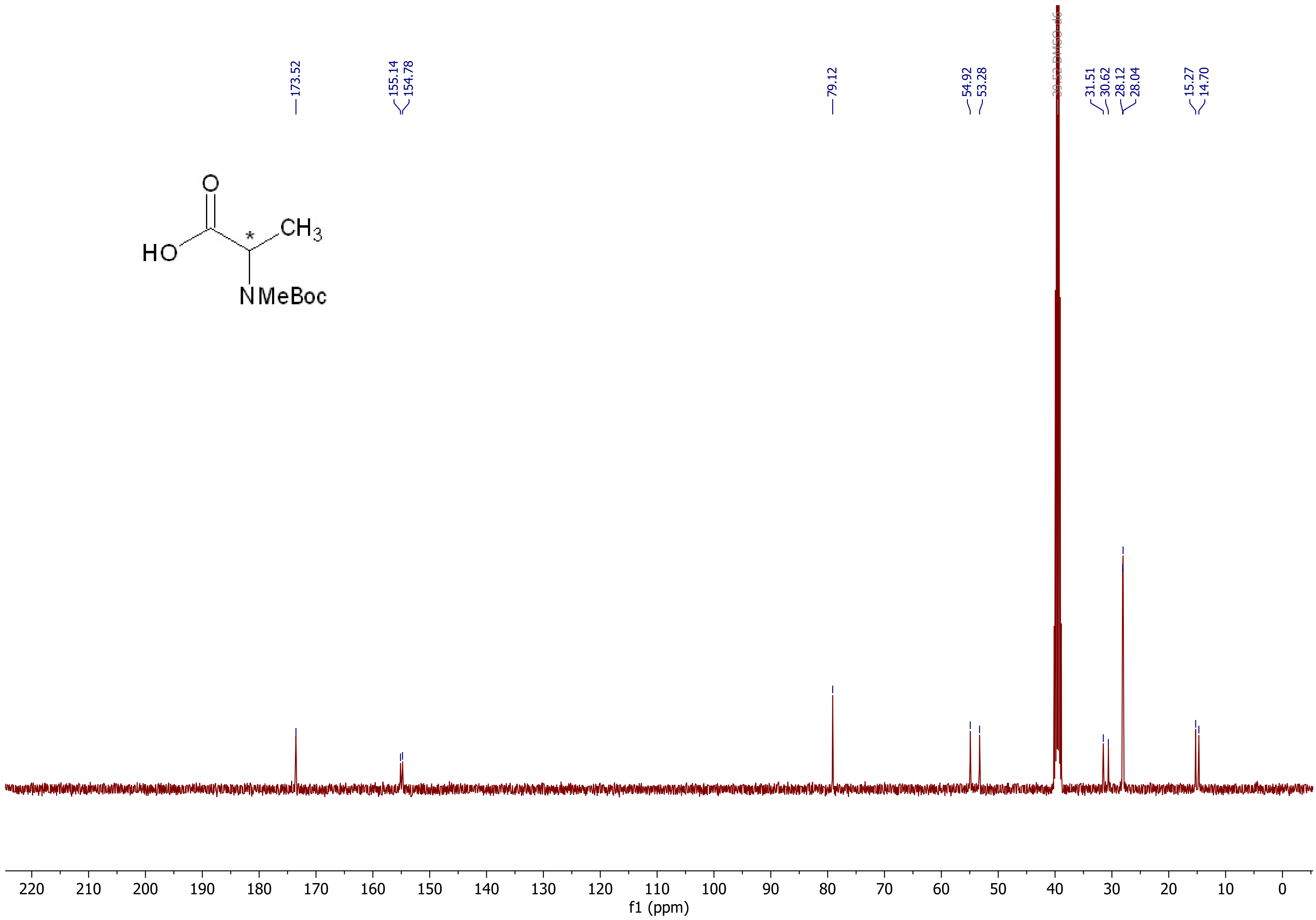

HO
O
CH3
*
NMeBoc
173.52
155.14
154.78
79.12
54.92
53.28
39.52 DMSO-d6
31.51
30.62
28.12
28.04
15.27
14.70
220
210
200
190
180
170
160
150
140
130
120
110
100
90
80
70
60
50
40
30
20
10
0
f1 (ppm)

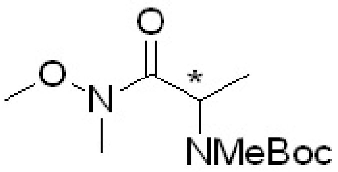

O
O
N
*
NMeBoc


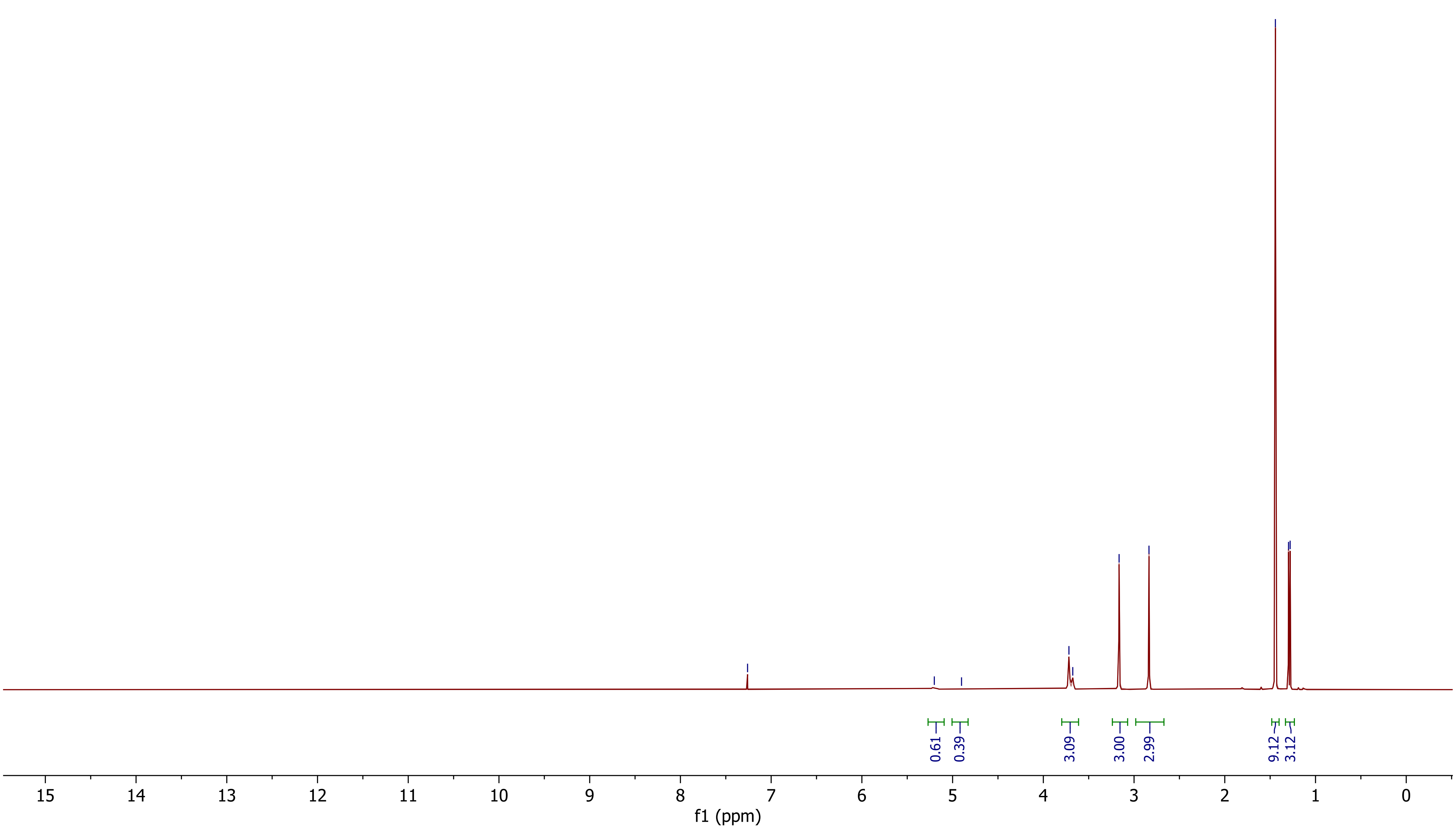

7.26 CDCl3
5.20
4.90
3.72
3.68
3.16
2.84
1.44
1.30
1.28
0.61
0.39
3.09
3.00
2.99
9.12
3.12
15 14 13 12 11 10 9 8 7 6 5 4 3 2 1 0
f1 (ppm)

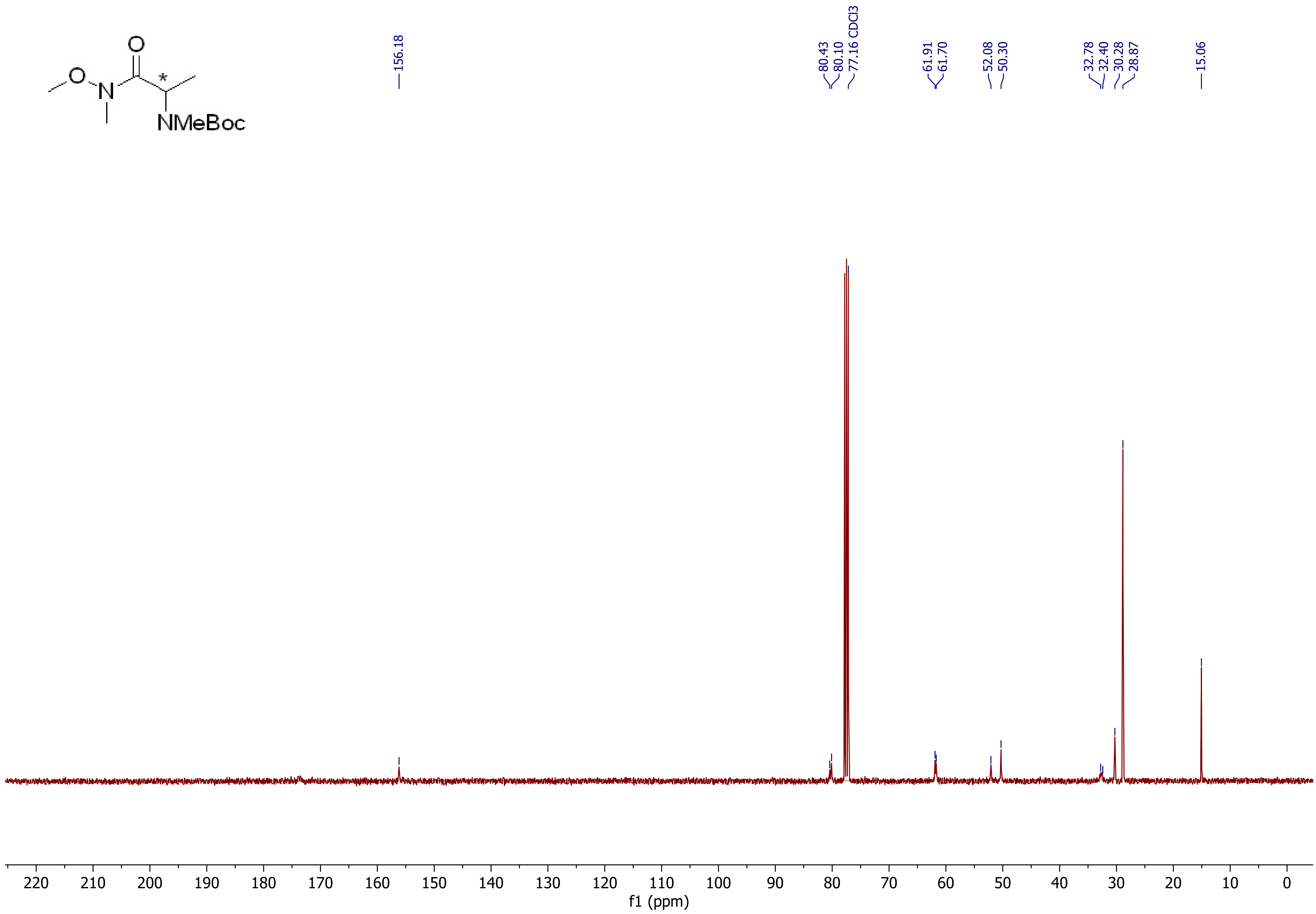
NMeBoc
156.18
80.43
80.10
77.16 CDCl3
61.91
61.70
52.08
50.30
32.78
32.40
30.28
28.87
15.06
220 210 200 190 180 170 160 150 140 130 120 110 100 90 80 70 60 50 40 30 20 10 0
f1 (ppm)

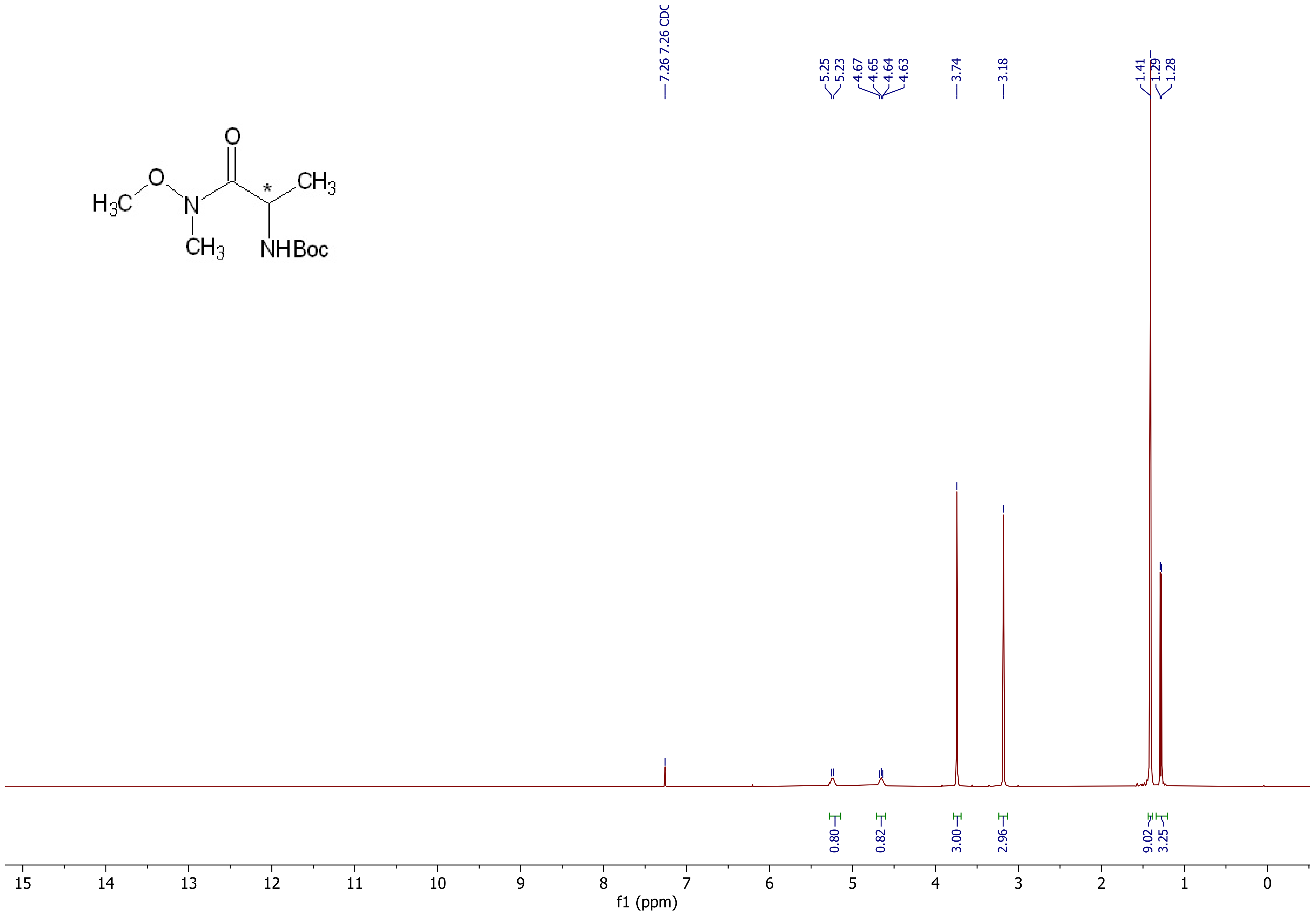

7.26 7.26 CDC
5.25
5.23
4.67
4.65
4.64
4.63
3.74
3.18
1.41
1.29
1.28
O
CH3
H3C
O
N
*
CH3
NHBoc
0.80
0.82
3.00
2.96
9.02
3.25
15
14
13
12
11
10
9
8
7
6
5
4
3
2
1
0
f1 (ppm)

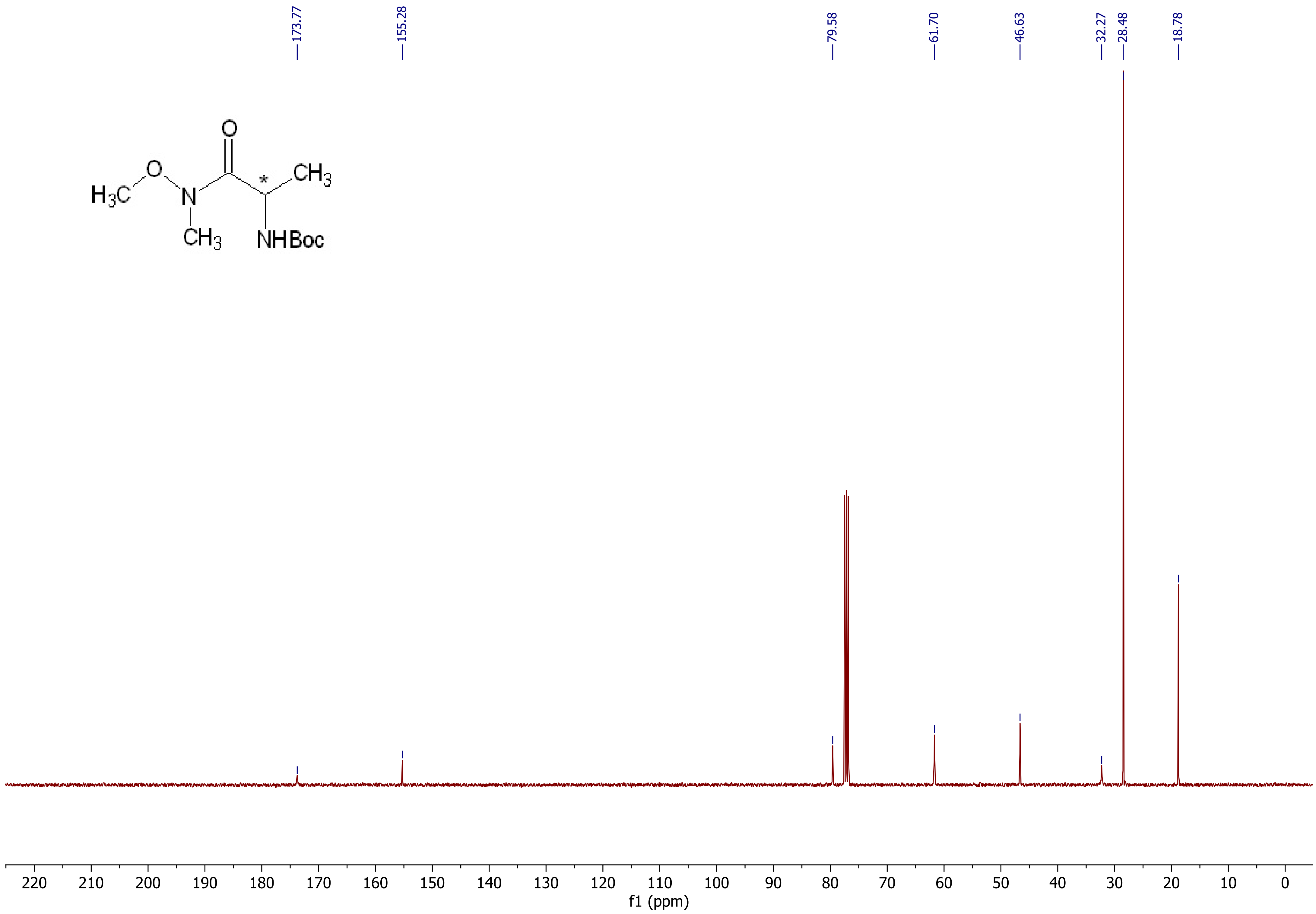

173.77
155.28
79.58
61.70
46.63
32.27
28.48
18.78
O
$H_3C$
N
$CH_3$
*
$CH_3$
NHBoc
220 210 200 190 180 170 160 150 140 130 120 110 100 90 80 70 60 50 40 30 20 10 0
f1 (ppm)

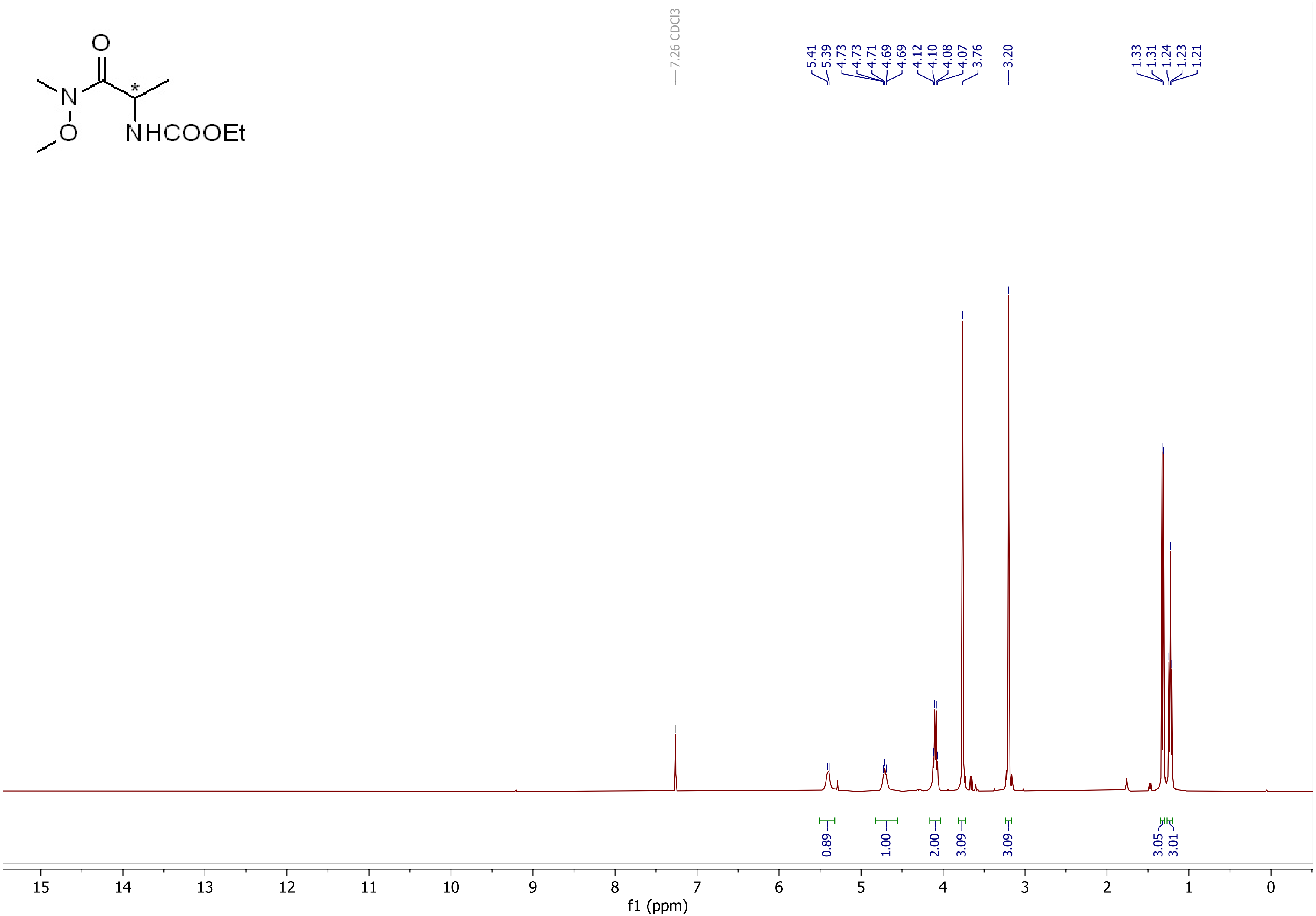

NHCOOEt
7.26 CDCl3
5.41
5.39
4.73
4.73
4.71
4.69
4.69
4.12
4.10
4.08
4.07
3.76
3.20
1.33
1.31
1.24
1.23
1.21
0.89
1.00
2.00
3.09
3.09
3.05
3.01
15
14
13
12
11
10
9
8
7
6
5
4
3
2
1
0
f1 (ppm)

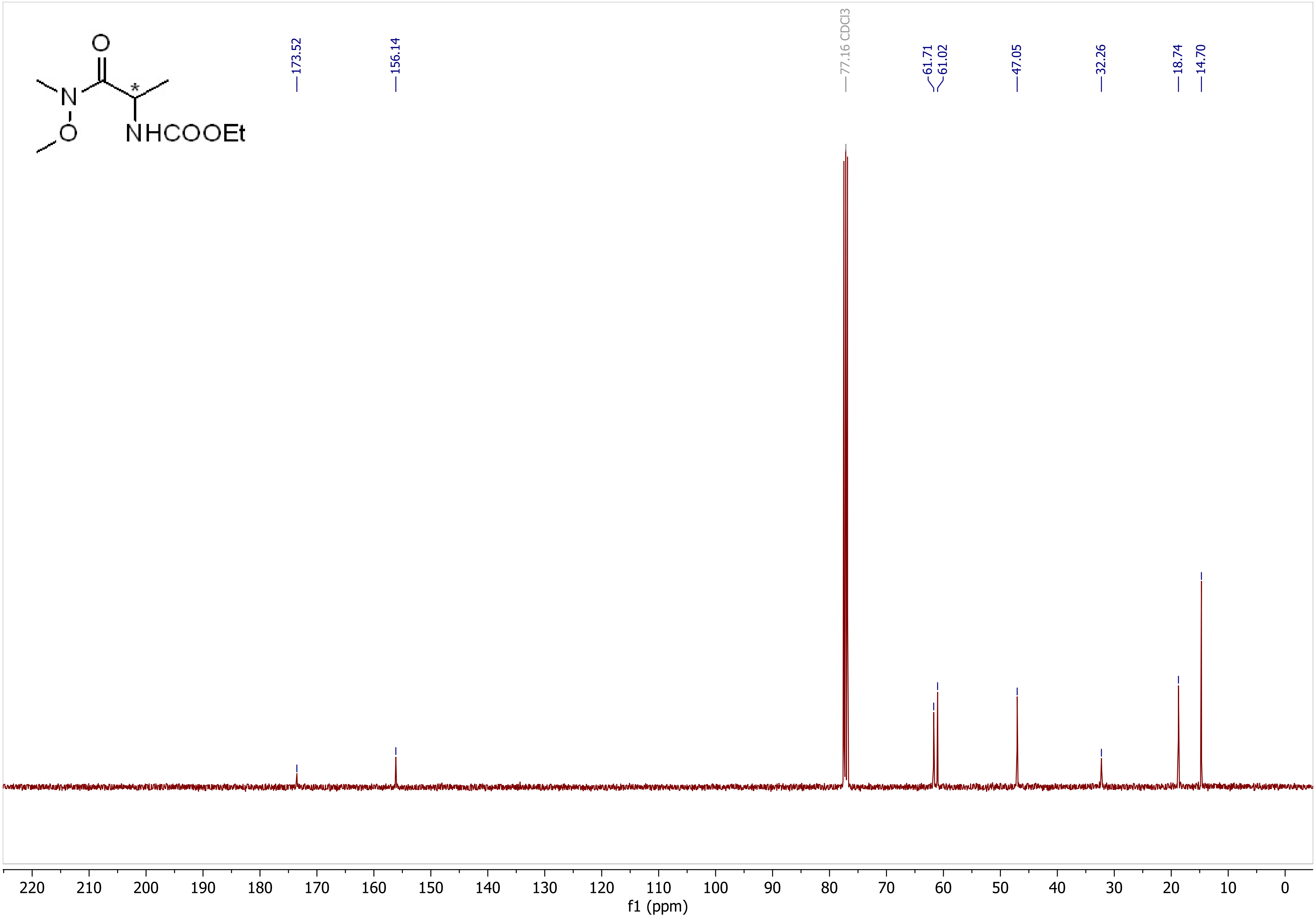

NHCOOEt
173.52
156.14
77.16 CDCl3
61.71
61.02
47.05
32.26
18.74
14.70
220 210 200 190 180 170 160 150 140 130 120 110 100 90 80 70 60 50 40 30 20 10 0
f1 (ppm)

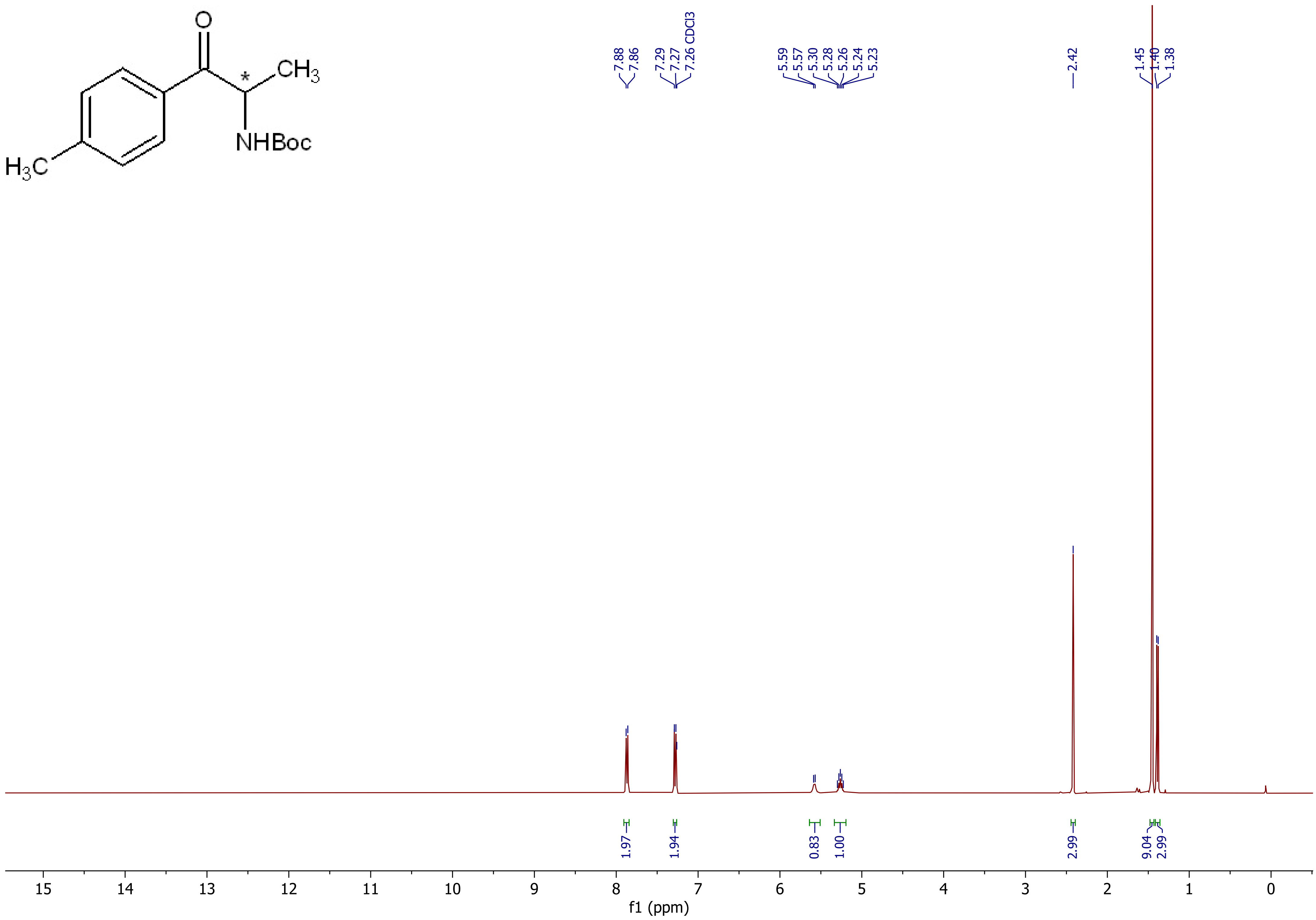
O
CH3
NHBoc
H3C
*
7.88
7.86
7.29
7.27
7.26 CDCl3
5.59
5.57
5.30
5.28
5.26
5.24
5.23
2.42
1.45
1.40
1.38
1.97
1.94
0.83
1.00
2.99
9.04
2.99
15
14
13
12
11
10
9
8
7
6
5
4
3
2
1
0
f1 (ppm)

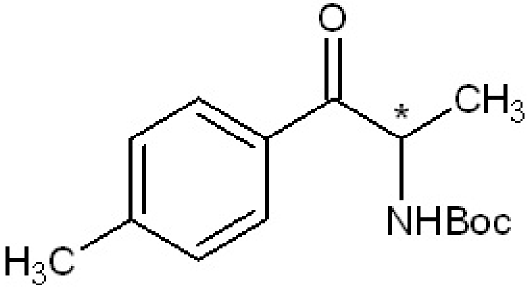

O
CH3
*
NHBoc
H3C


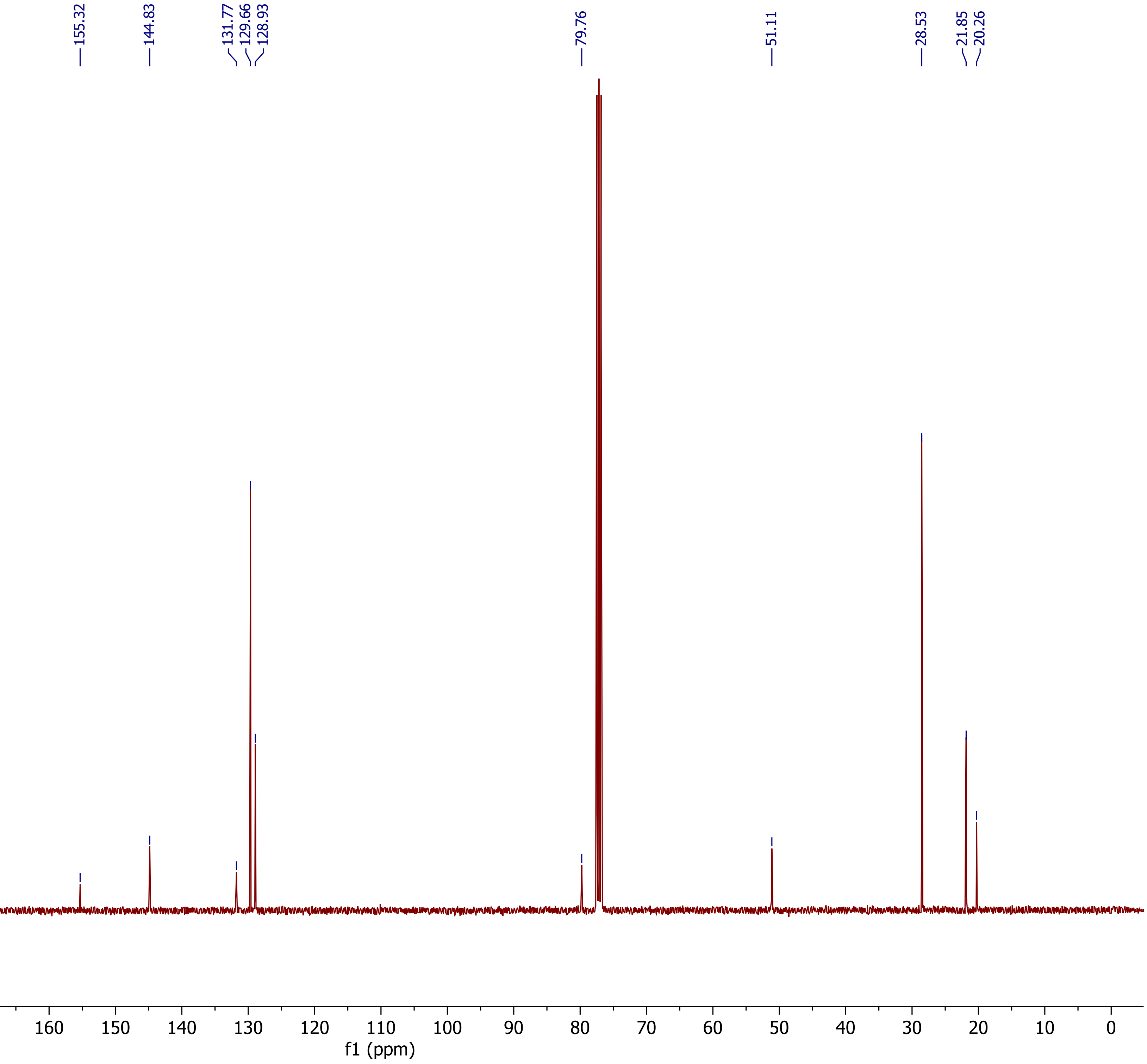

155.32
144.83
131.77
129.66
128.93
79.76
51.11
28.53
21.85
20.26
220
210
200
190
180
170
160
150
140
130
120
110
100
90
80
70
60
50
40
30
20
10
0
f1 (ppm)

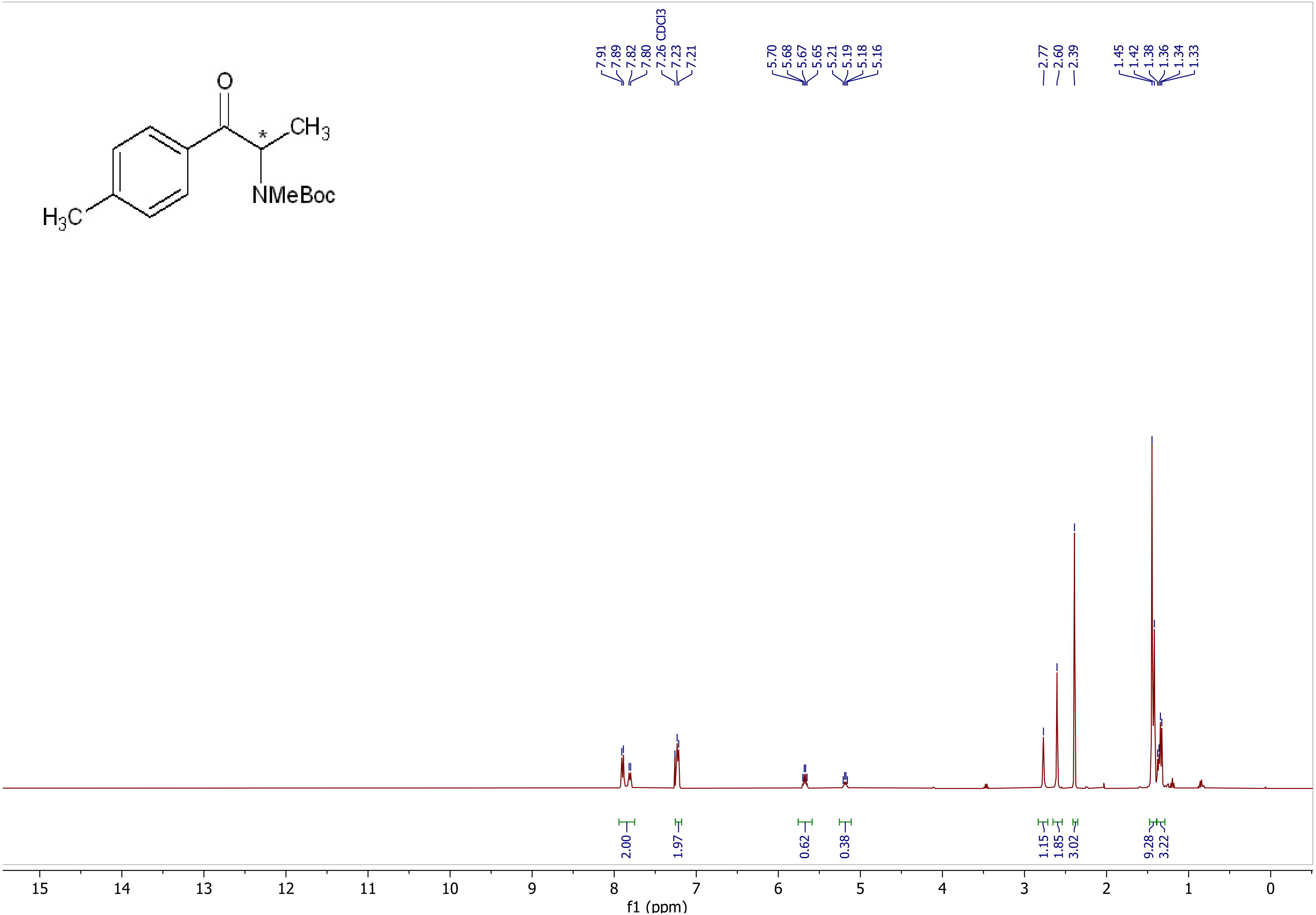

O
CH3
*
NMeBoc
H3C
7.91
7.89
7.82
7.80
7.26 CDCl3
7.23
7.21
5.70
5.68
5.67
5.65
5.21
5.19
5.18
5.16
2.77
2.60
2.39
1.45
1.42
1.38
1.36
1.34
1.33
2.00
1.97
0.62
0.38
1.15
1.85
3.02
9.28
3.22
15 14 13 12 11 10 9 8 7 6 5 4 3 2 1 0
f1 (ppm)

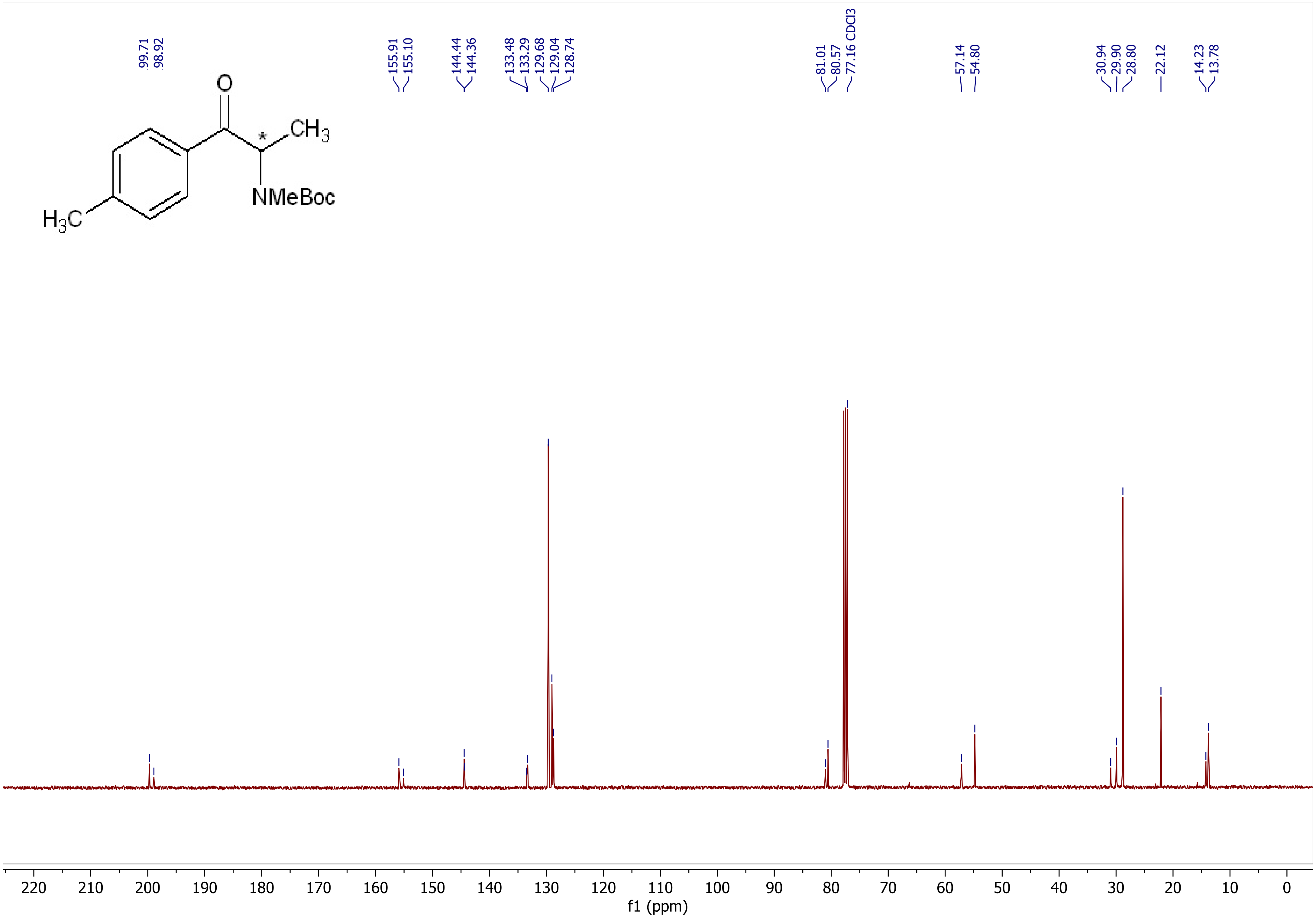

99.71
98.92
155.91
155.10
144.44
144.36
133.48
133.29
129.68
129.04
128.74
81.01
80.57
77.16 CDCl3
57.14
54.80
30.94
29.90
28.80
22.12
14.23
13.78
O
CH3
H3C
NMeBoc
*
220 210 200 190 180 170 160 150 140 130 120 110 100 90 80 70 60 50 40 30 20 10 0
f1 (ppm)

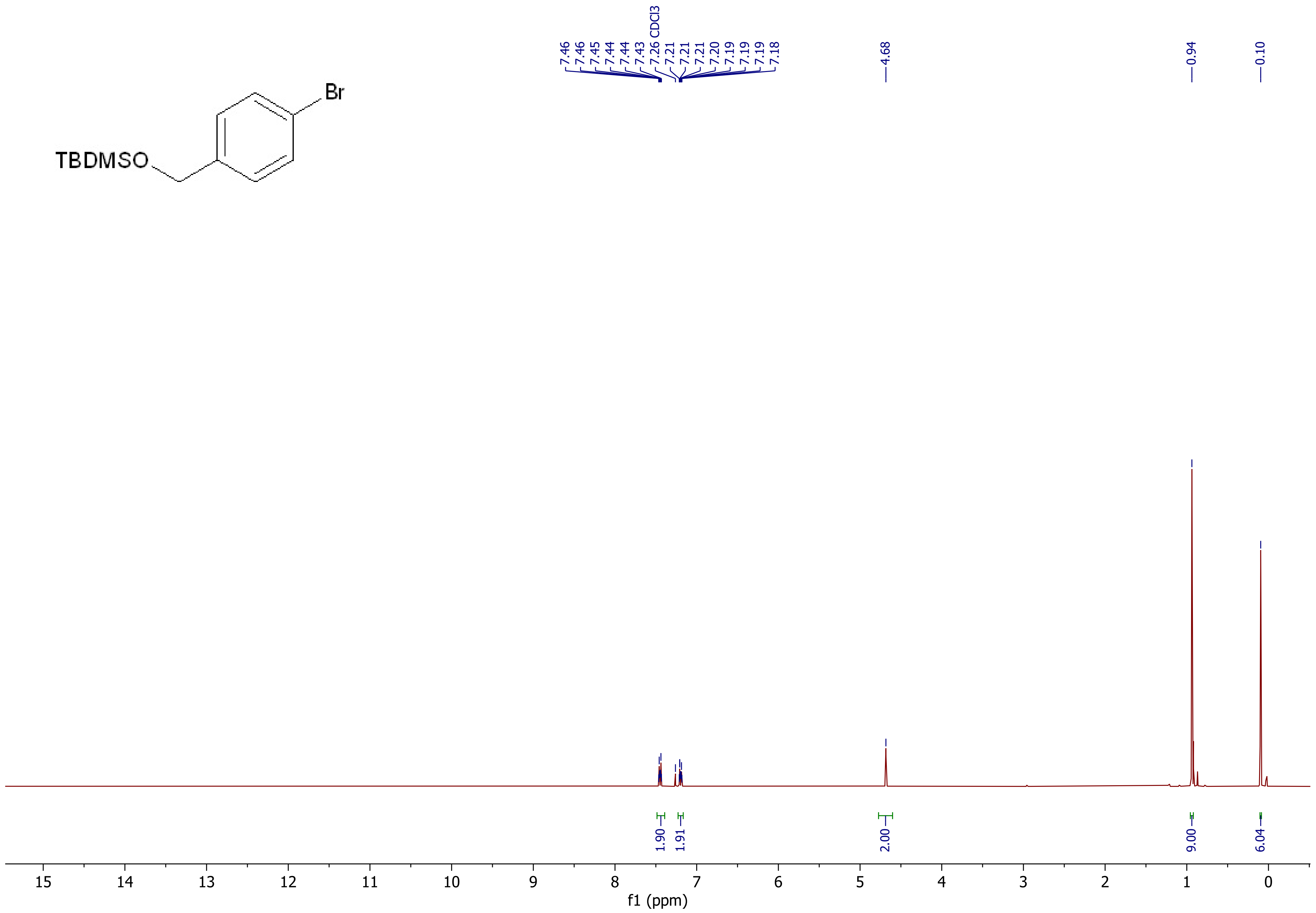

TBDMSO
Br
7.46
7.46
7.45
7.44
7.44
7.43
7.26 CDCl3
7.21
7.21
7.21
7.20
7.19
7.19
7.19
7.18
4.68
0.94
0.10
1.90
1.91
2.00
9.00
6.04
15 14 13 12 11 10 9 8 7 6 5 4 3 2 1 0
f1 (ppm)

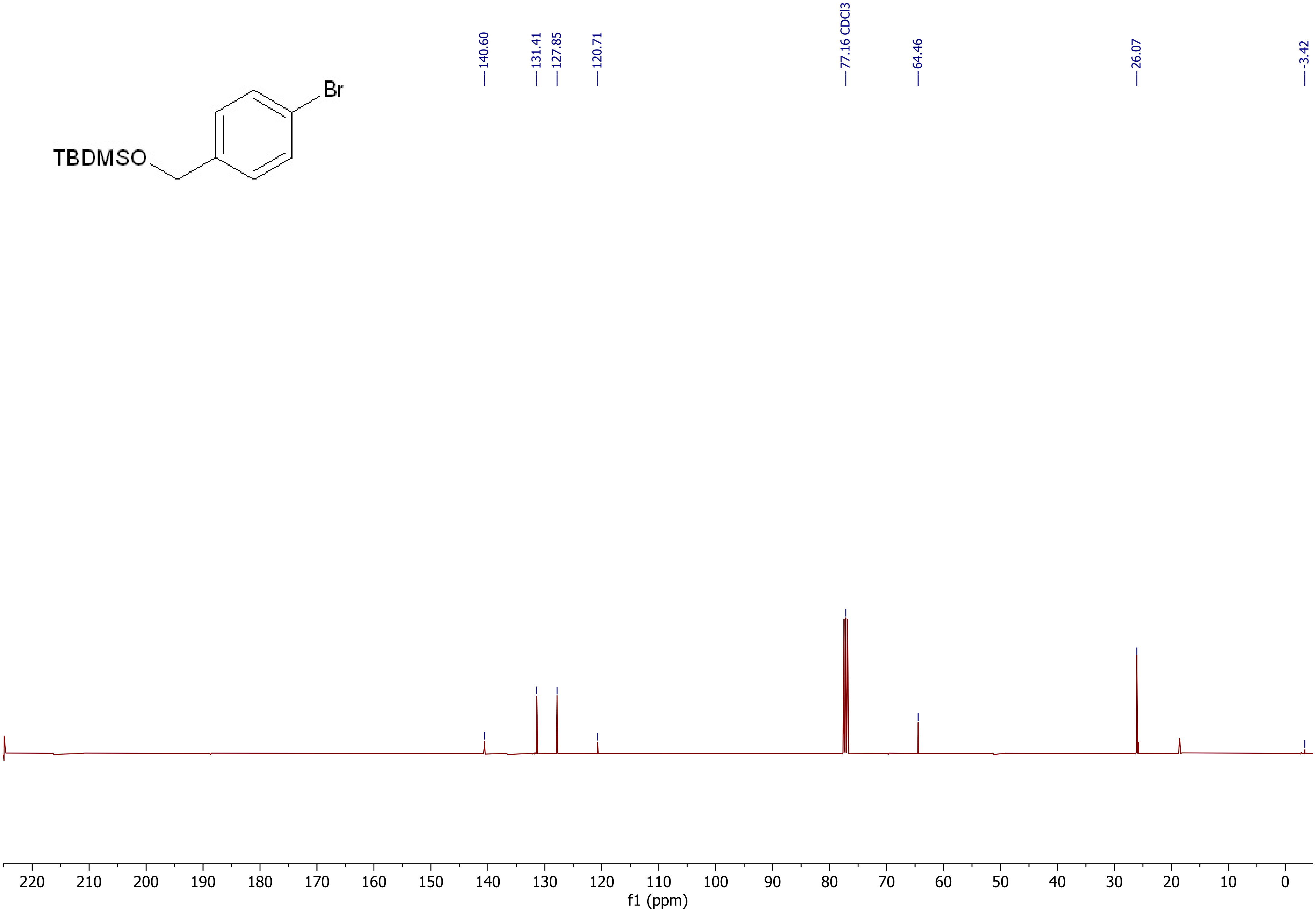

Br
TBDMSO
140.60
131.41
127.85
120.71
77.16 CDCl3
64.46
26.07
-3.42
220 210 200 190 180 170 160 150 140 130 120 110 100 90 80 70 60 50 40 30 20 10 0
f1 (ppm)

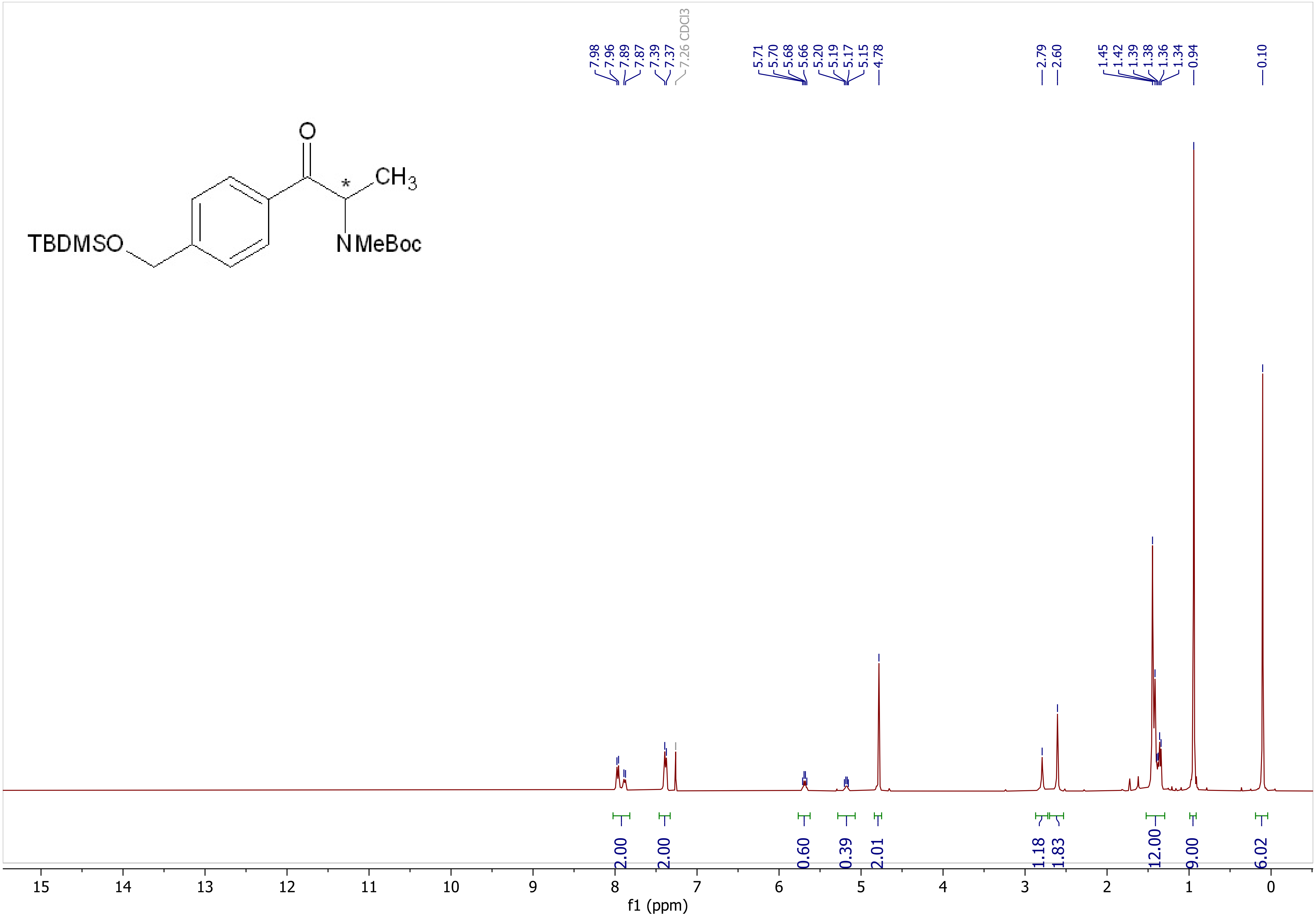

TBDMSO
O
CH3
*
NMeBoc
7.98
7.96
7.89
7.87
7.39
7.37
7.26 CDCl3
5.71
5.70
5.68
5.66
5.20
5.19
5.17
5.15
4.78
2.79
2.60
1.45
1.42
1.39
1.38
1.36
1.34
0.94
0.10
2.00
2.00
0.60
0.39
2.01
1.18
1.83
12.00
9.00
6.02
15
14
13
12
11
10
9
8
7
6
5
4
3
2
1
0
f1 (ppm)

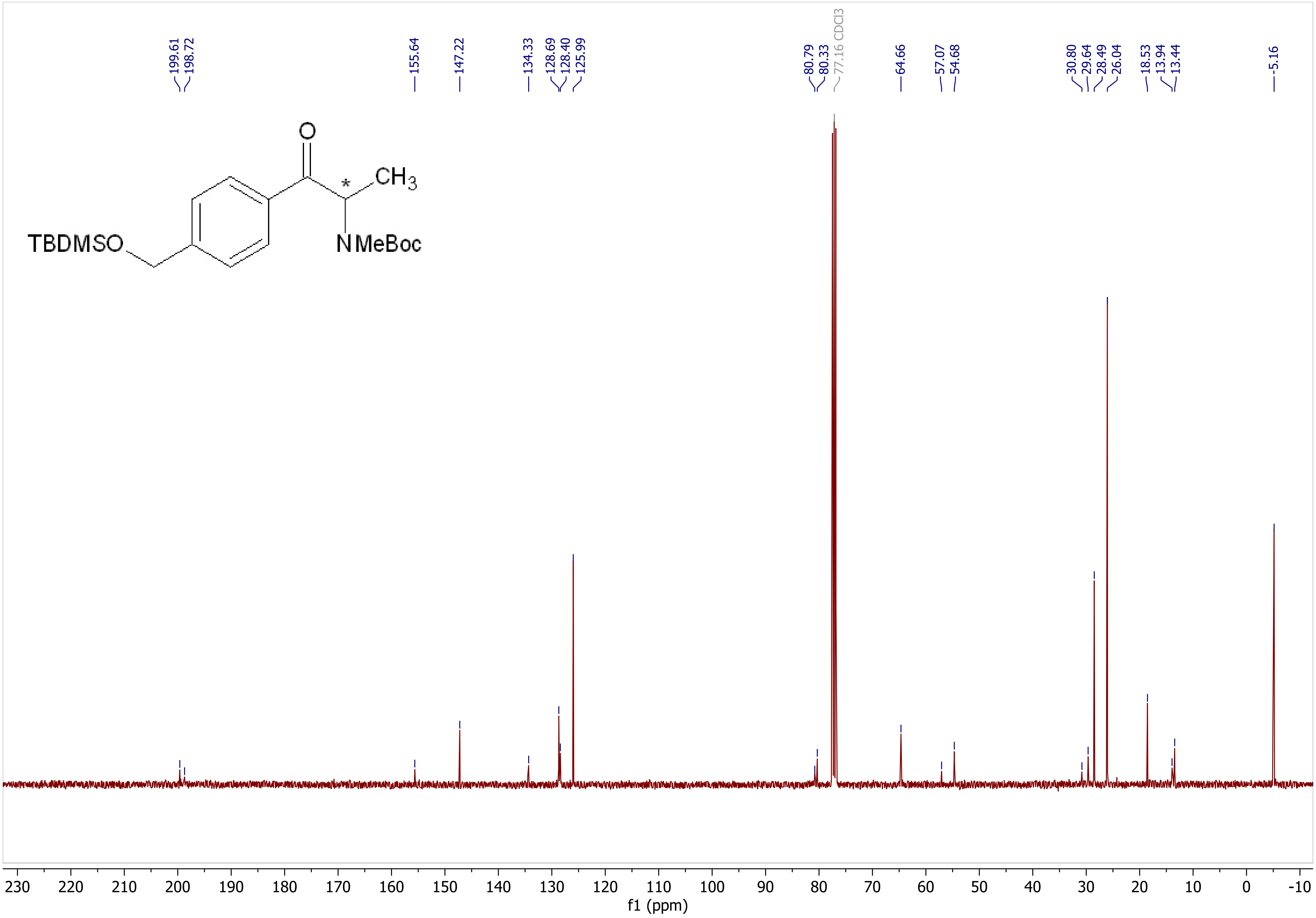

199.61
198.72
155.64
147.22
134.33
128.69
128.40
125.99
80.79
80.33
77.16 CDCl3
64.66
57.07
54.68
30.80
29.64
28.49
26.04
18.53
13.94
13.44
-5.16
O
CH3
*
TBDMSO
NMeBoc
230 220 210 200 190 180 170 160 150 140 130 120 110 100 90 80 70 60 50 40 30 20 10 0 -10
f1 (ppm)

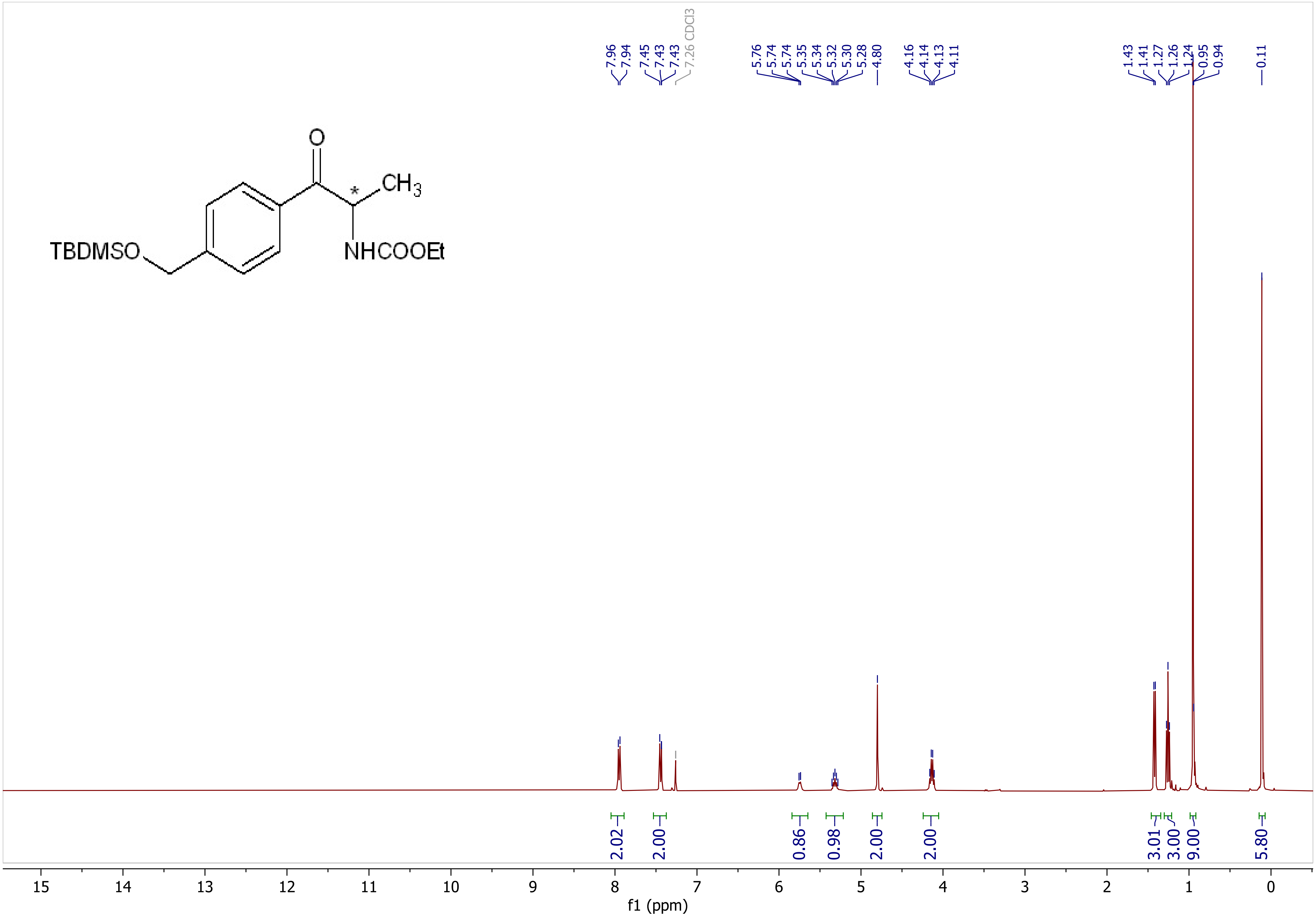

TBDMSO
O
CH3
*
NHCOOEt
7.96
7.94
7.45
7.43
7.43
7.26 CDCl3
5.76
5.74
5.74
5.35
5.34
5.32
5.30
5.28
4.80
4.16
4.14
4.13
4.11
1.43
1.41
1.27
1.26
1.24
0.95
0.94
0.11
2.02
2.00
0.86
0.98
2.00
2.00
3.01
3.00
9.00
5.80
15 14 13 12 11 10 9 8 7 6 5 4 3 2 1 0
f1 (ppm)

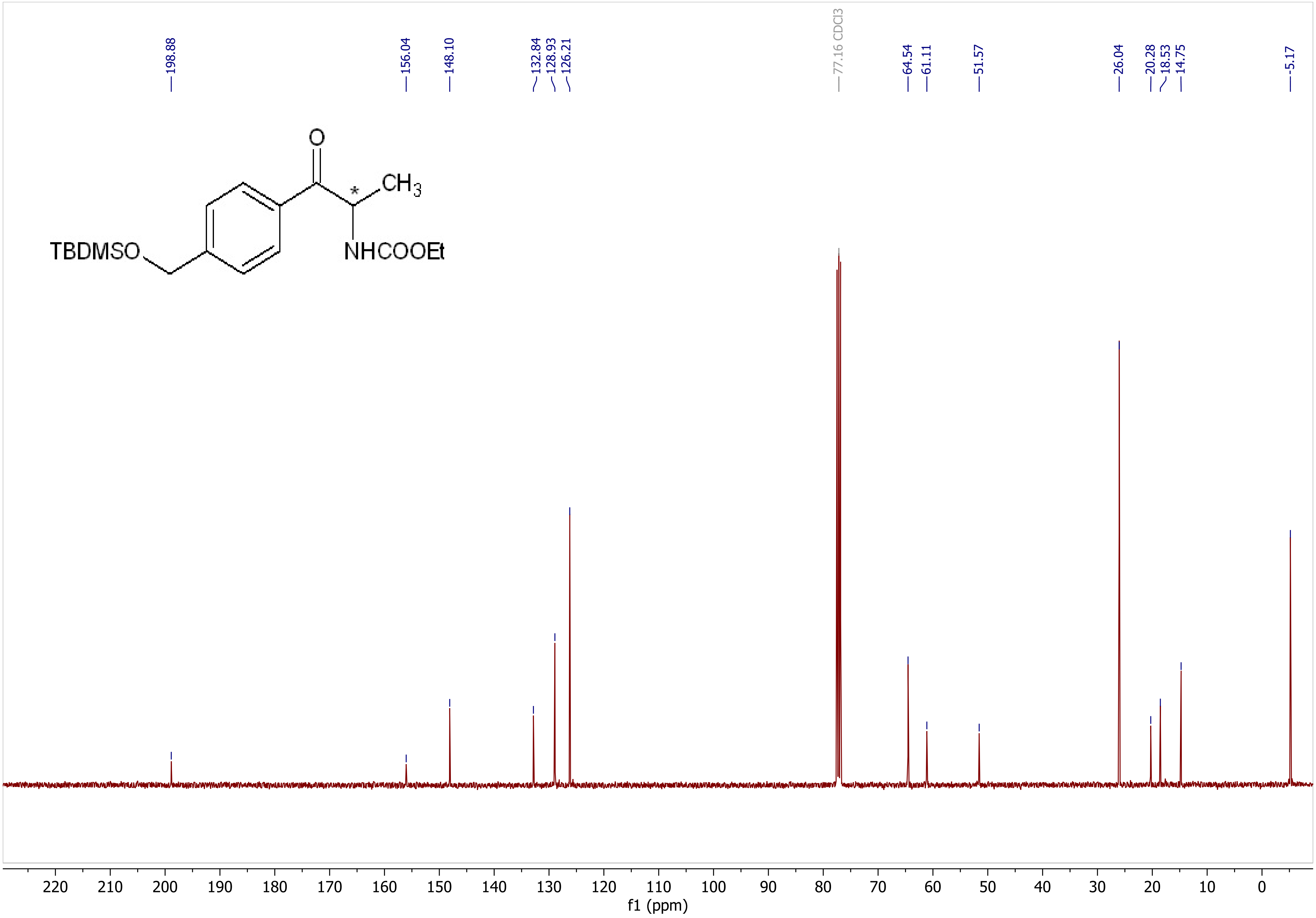
198.88
156.04
148.10
132.84
128.93
126.21
77.16 CDCl3
64.54
61.11
51.57
26.04
20.28
18.53
14.75
-5.17
O
CH3
TBDMSO
NHCOOEt
220
210
200
190
180
170
160
150
140
130
120
110
100
90
80
70
60
50
40
30
20
10
0
f1 (ppm)

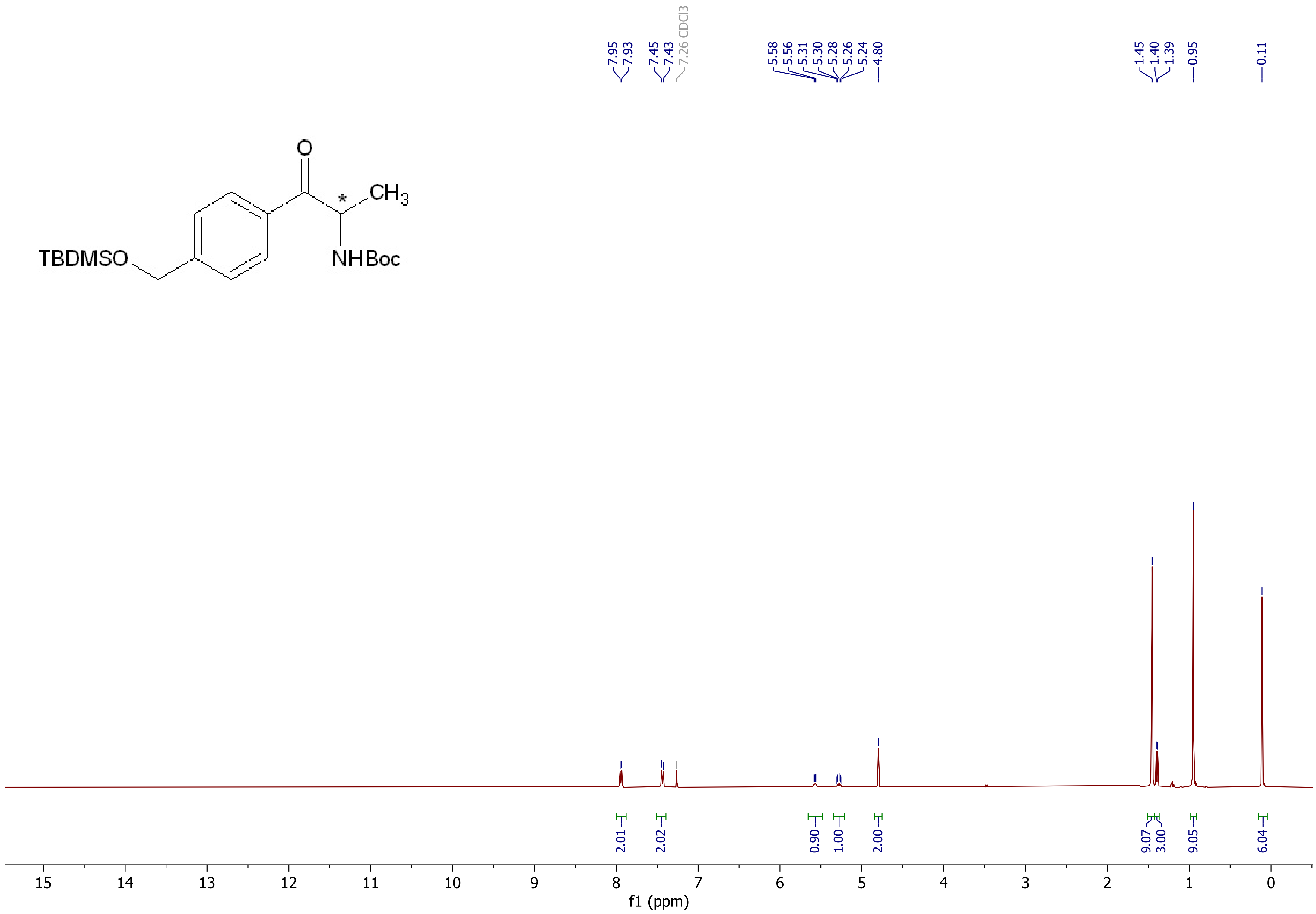

TBDMSO
O
CH3
*
NHBoc
7.95
7.93
7.45
7.43
7.26 CDCl3
5.58
5.56
5.31
5.30
5.28
5.26
5.24
4.80
1.45
1.40
1.39
0.95
0.11
2.01
2.02
0.90
1.00
2.00
9.07
3.00
9.05
6.04
15
14
13
12
11
10
9
8
7
6
5
4
3
2
1
0
f1 (ppm)

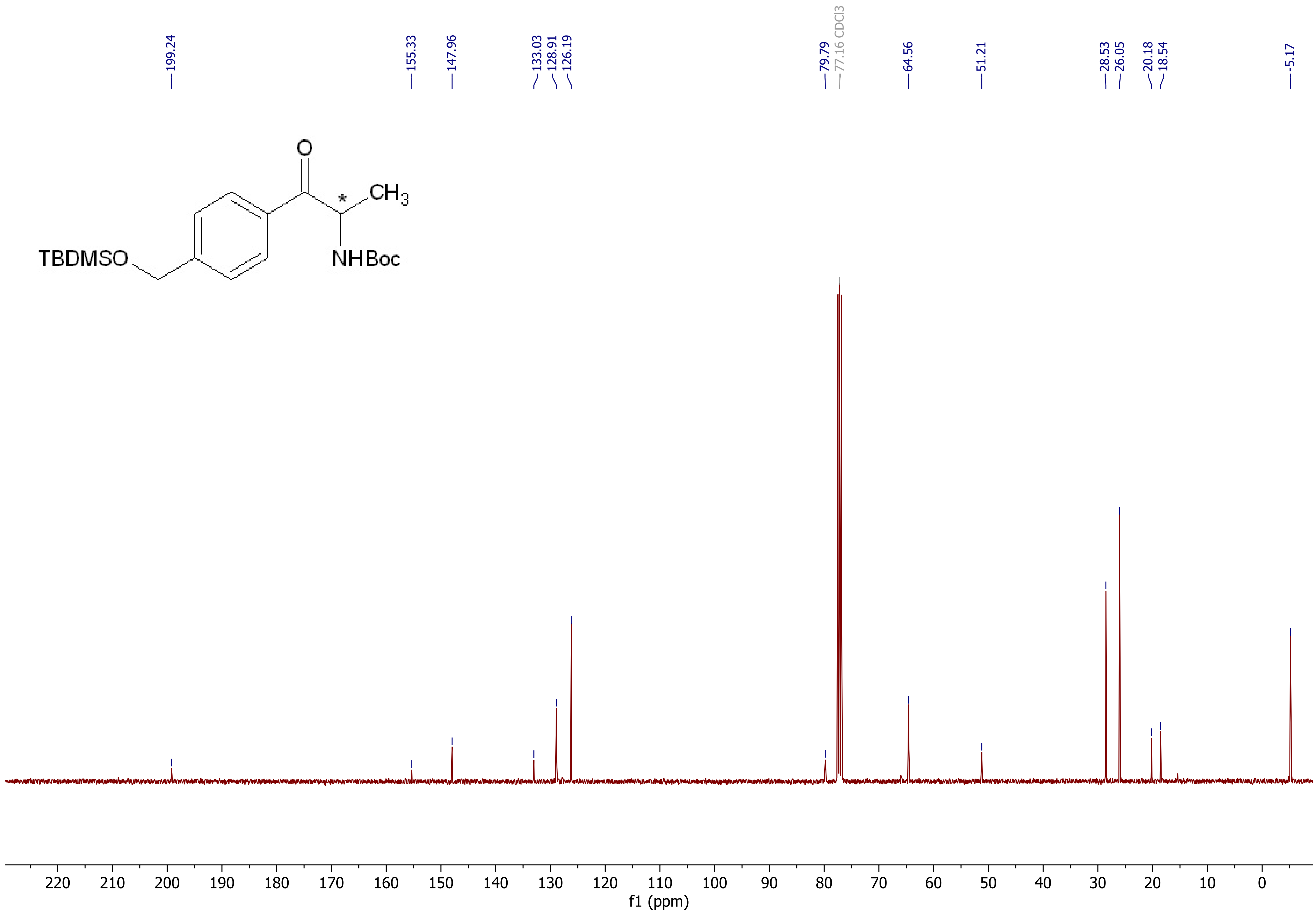

199.24
155.33
147.96
133.03
128.91
126.19
79.79
77.16 CDCl3
64.56
51.21
28.53
26.05
20.18
18.54
-5.17
O
CH3
*
TBDMSO
NHBoc
220 210 200 190 180 170 160 150 140 130 120 110 100 90 80 70 60 50 40 30 20 10 0
f1 (ppm)

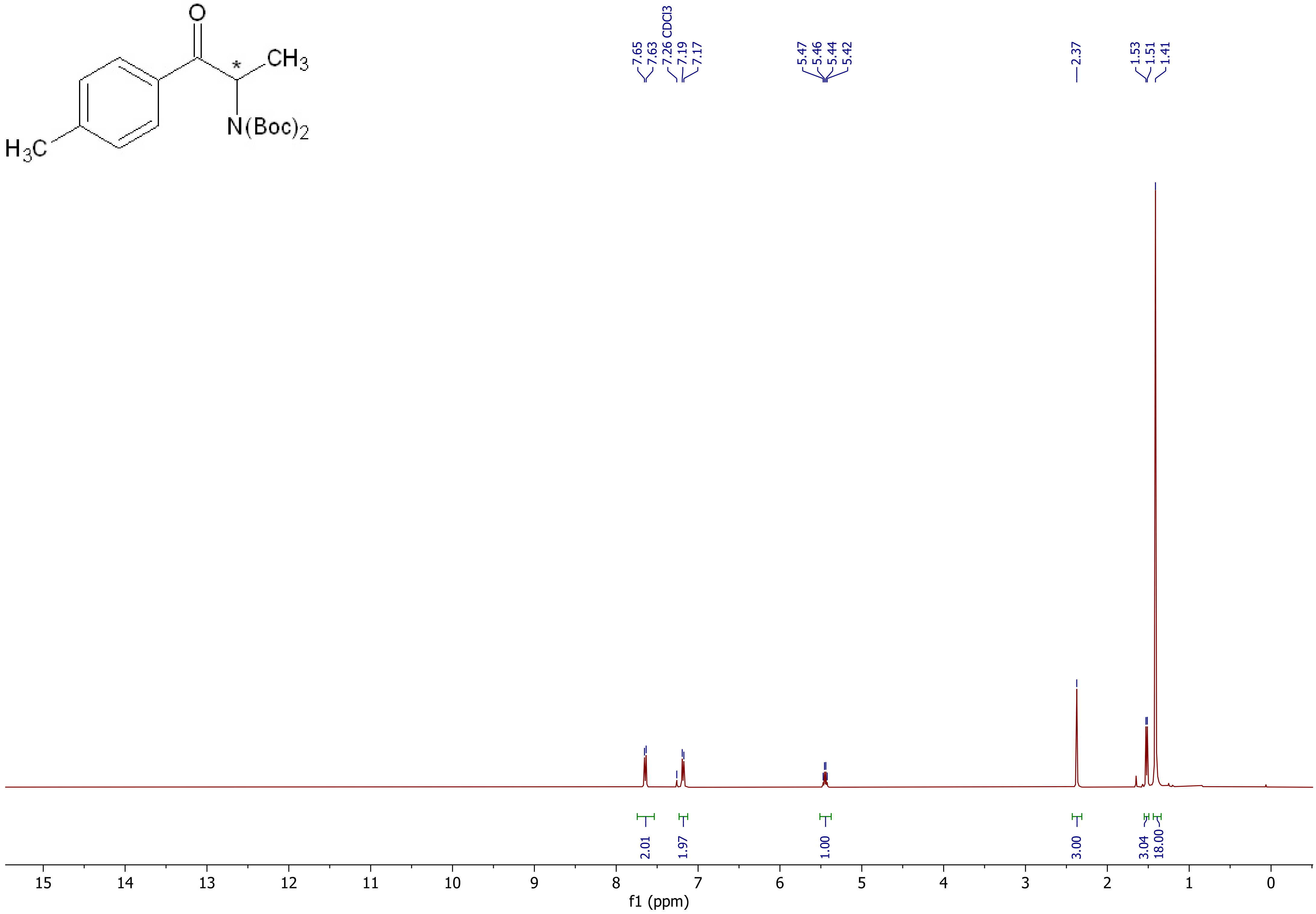

O
CH3
*
N(Boc)2
H3C
7.65
7.63
7.26 CDCl3
7.19
7.17
5.47
5.46
5.44
5.42
2.37
1.53
1.51
1.41
2.01
1.97
1.00
3.00
3.04
18.00
15
14
13
12
11
10
9
8
7
6
5
4
3
2
1
0
f1 (ppm)

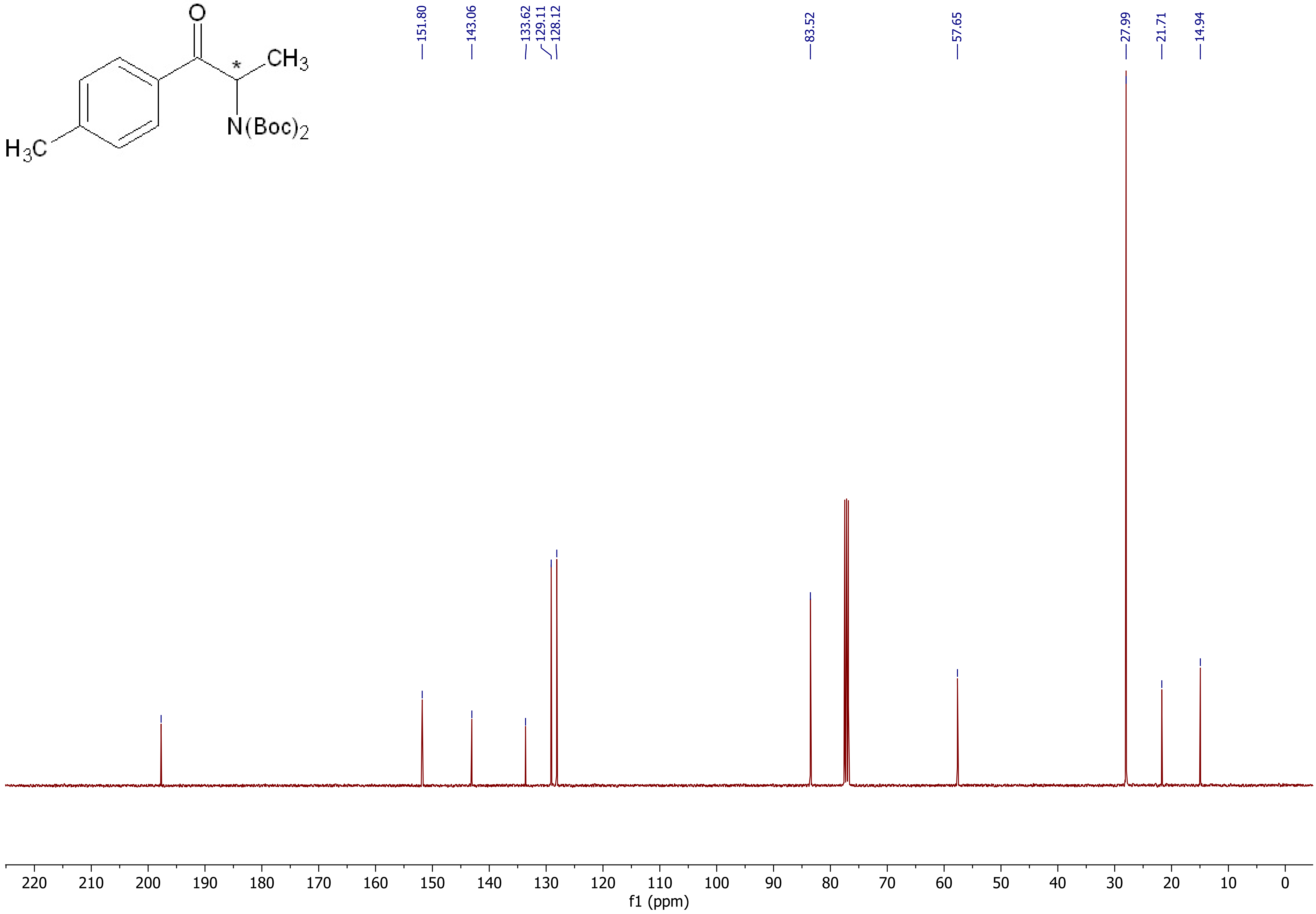

O
CH3
*
N(Boc)2
H3C
151.80
143.06
133.62
129.11
128.12
83.52
57.65
27.99
21.71
14.94
220 210 200 190 180 170 160 150 140 130 120 110 100 90 80 70 60 50 40 30 20 10 0
f1 (ppm)

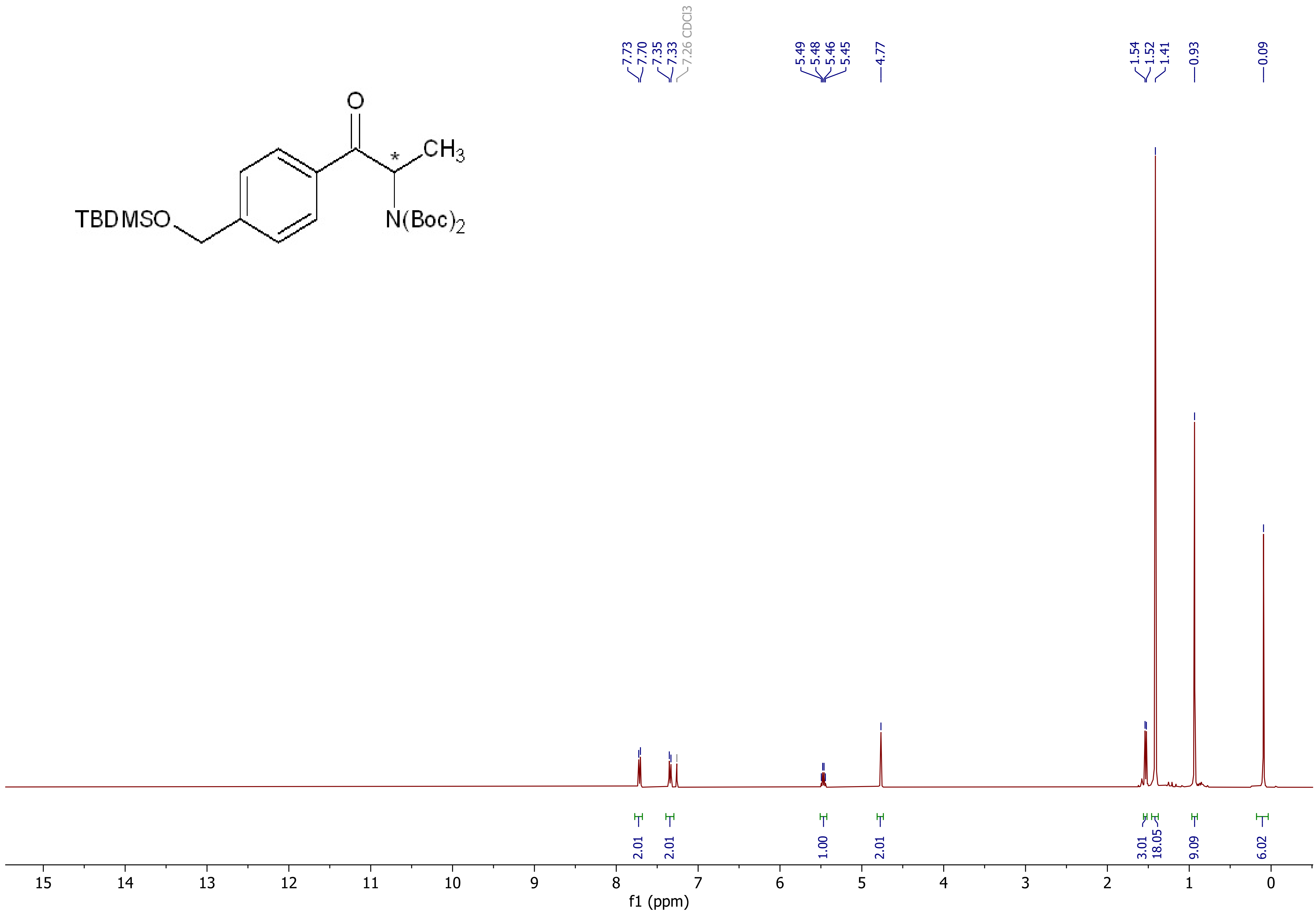

O
CH3
*
TBDMSO
N(Boc)2
7.73
7.70
7.35
7.33
7.26 CDCl3
5.49
5.48
5.46
5.45
4.77
1.54
1.52
1.41
0.93
0.09
2.01
2.01
1.00
2.01
3.01
18.05
9.09
6.02
15
14
13
12
11
10
9
8
7
6
5
4
3
2
1
0
f1 (ppm)

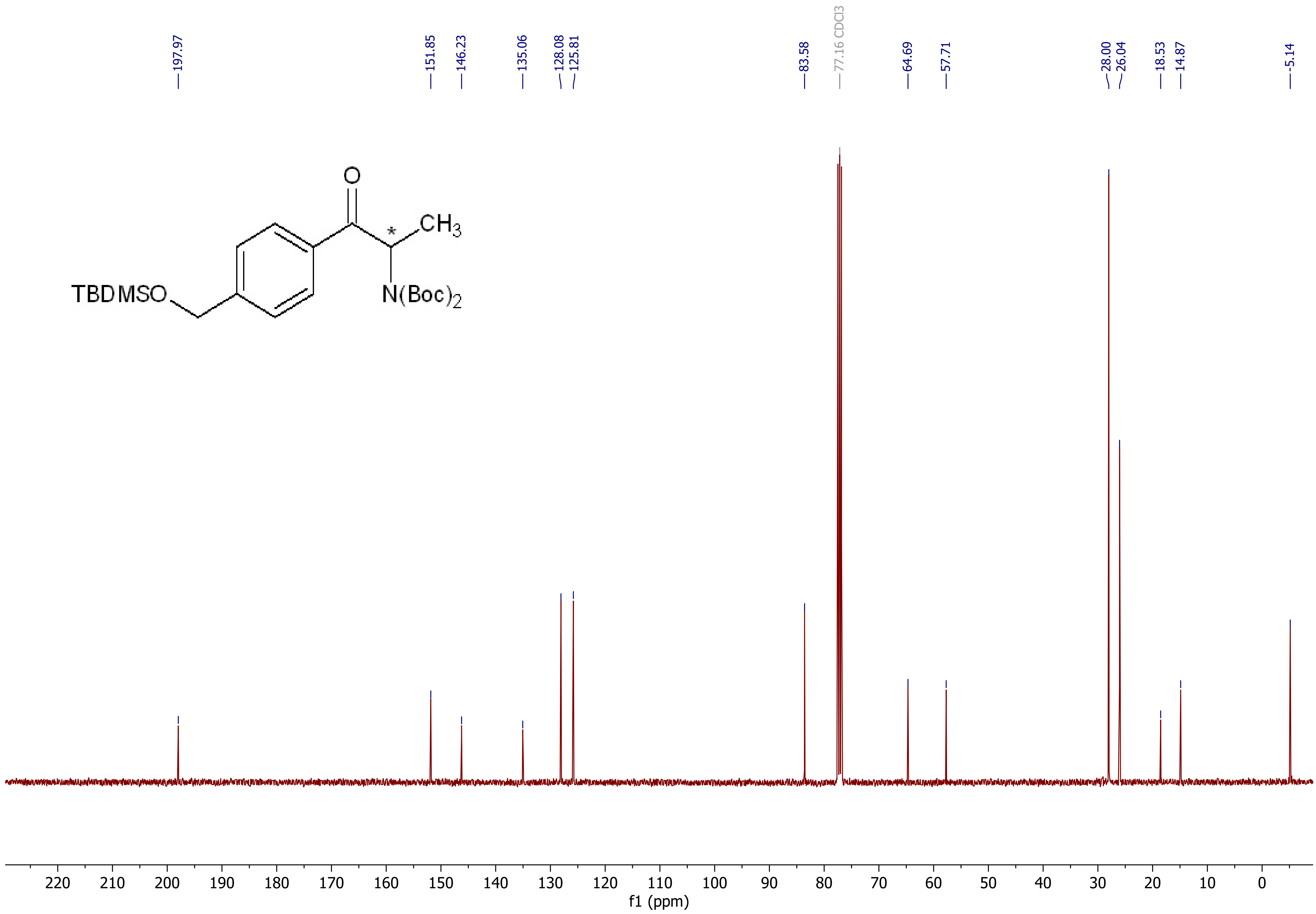

197.97
151.85
146.23
135.06
128.08
125.81
83.58
77.16 CDCl3
64.69
57.71
28.00
26.04
18.53
14.87
-5.14
TBDMSO
O
CH3
*
N(Boc)2
220 210 200 190 180 170 160 150 140 130 120 110 100 90 80 70 60 50 40 30 20 10 0
f1 (ppm)

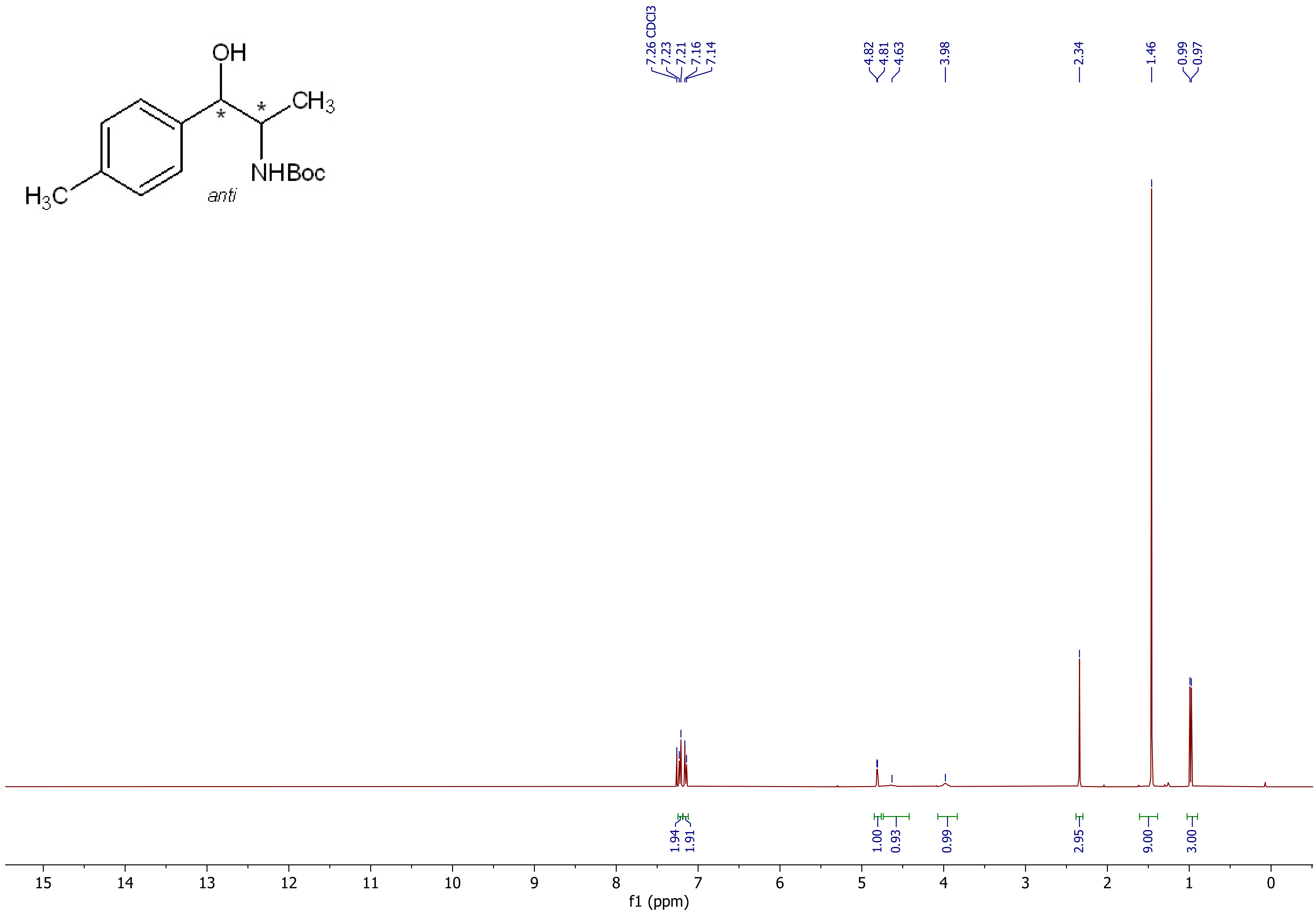

OH
CH3
H3C
NHBoc
anti
7.26 CDCl3
7.23
7.21
7.16
7.14
4.82
4.81
4.63
3.98
2.34
1.46
0.99
0.97
1.94
1.91
1.00
0.93
0.99
2.95
9.00
3.00
15 14 13 12 11 10 9 8 7 6 5 4 3 2 1 0
f1 (ppm)

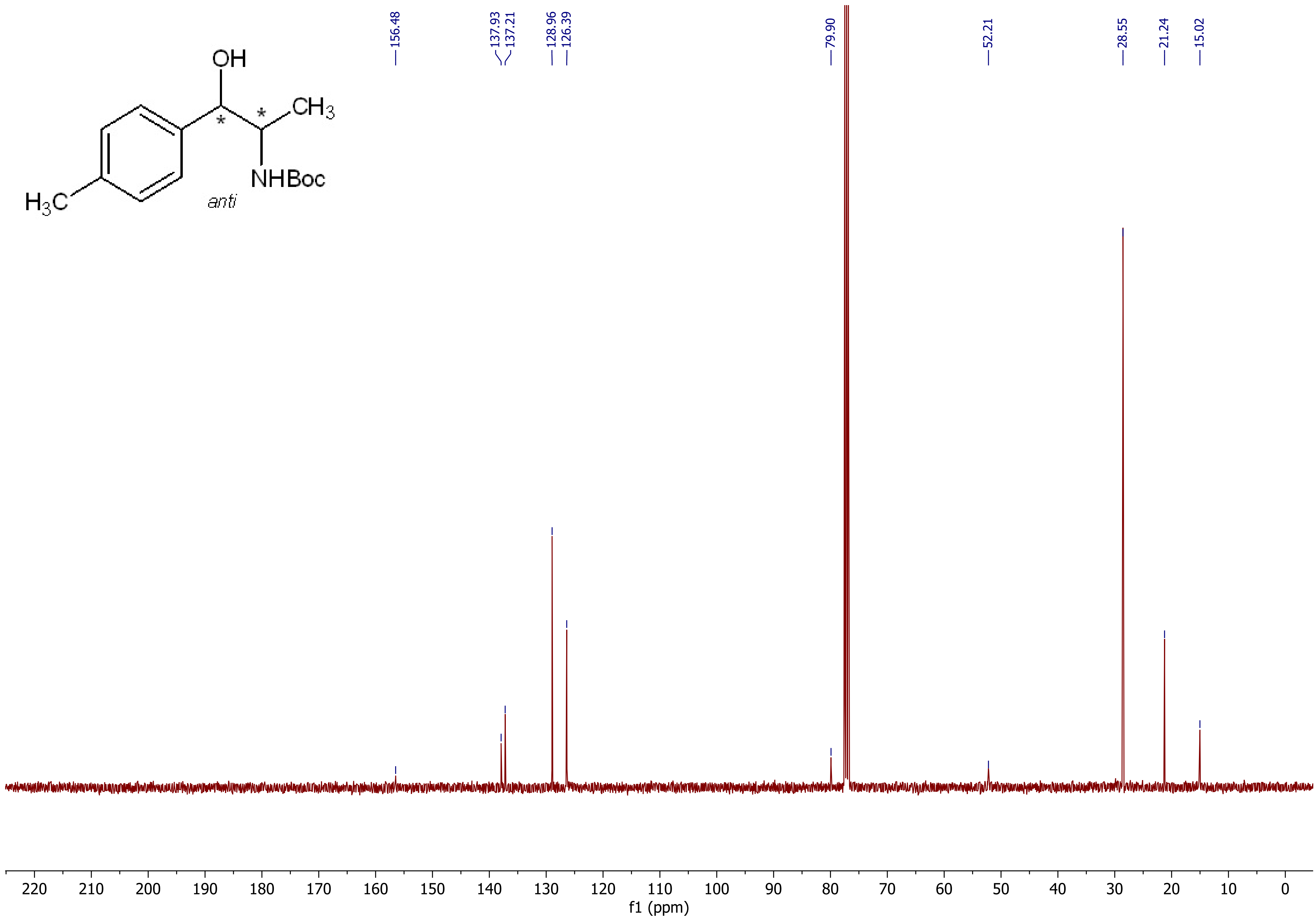

OH
CH3
NHBoc
H3C
anti
156.48
137.93
137.21
128.96
126.39
79.90
52.21
28.55
21.24
15.02
220 210 200 190 180 170 160 150 140 130 120 110 100 90 80 70 60 50 40 30 20 10 0
f1 (ppm)

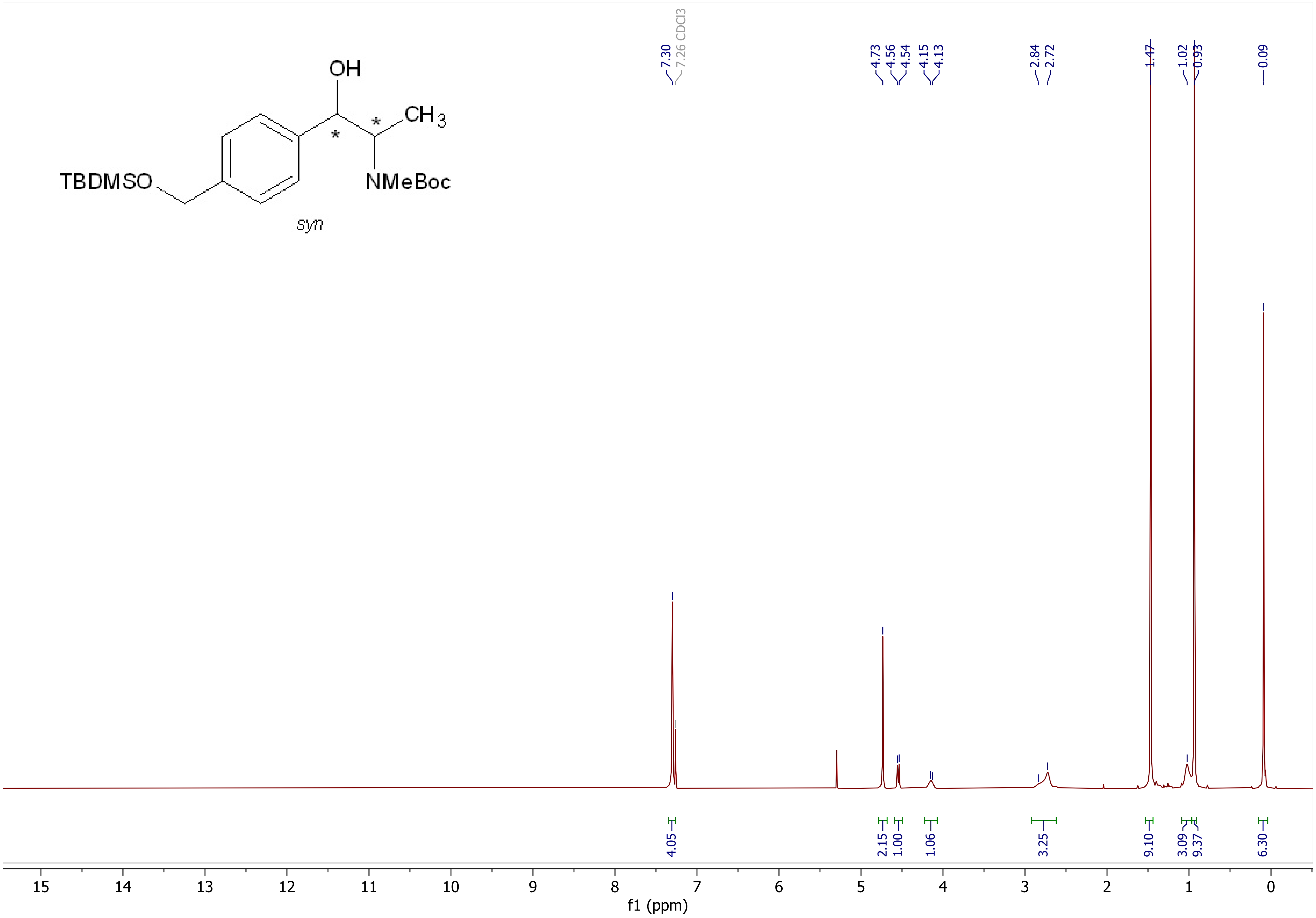

OH
CH3
TBDMSO
NMeBoc
syn
7.30
7.26 CDCl3
4.73
4.56
4.54
4.15
4.13
2.84
2.72
1.47
1.02
0.93
0.09
4.05
2.15
1.00
1.06
3.25
9.10
3.09
9.37
6.30
15 14 13 12 11 10 9 8 7 6 5 4 3 2 1 0
f1 (ppm)

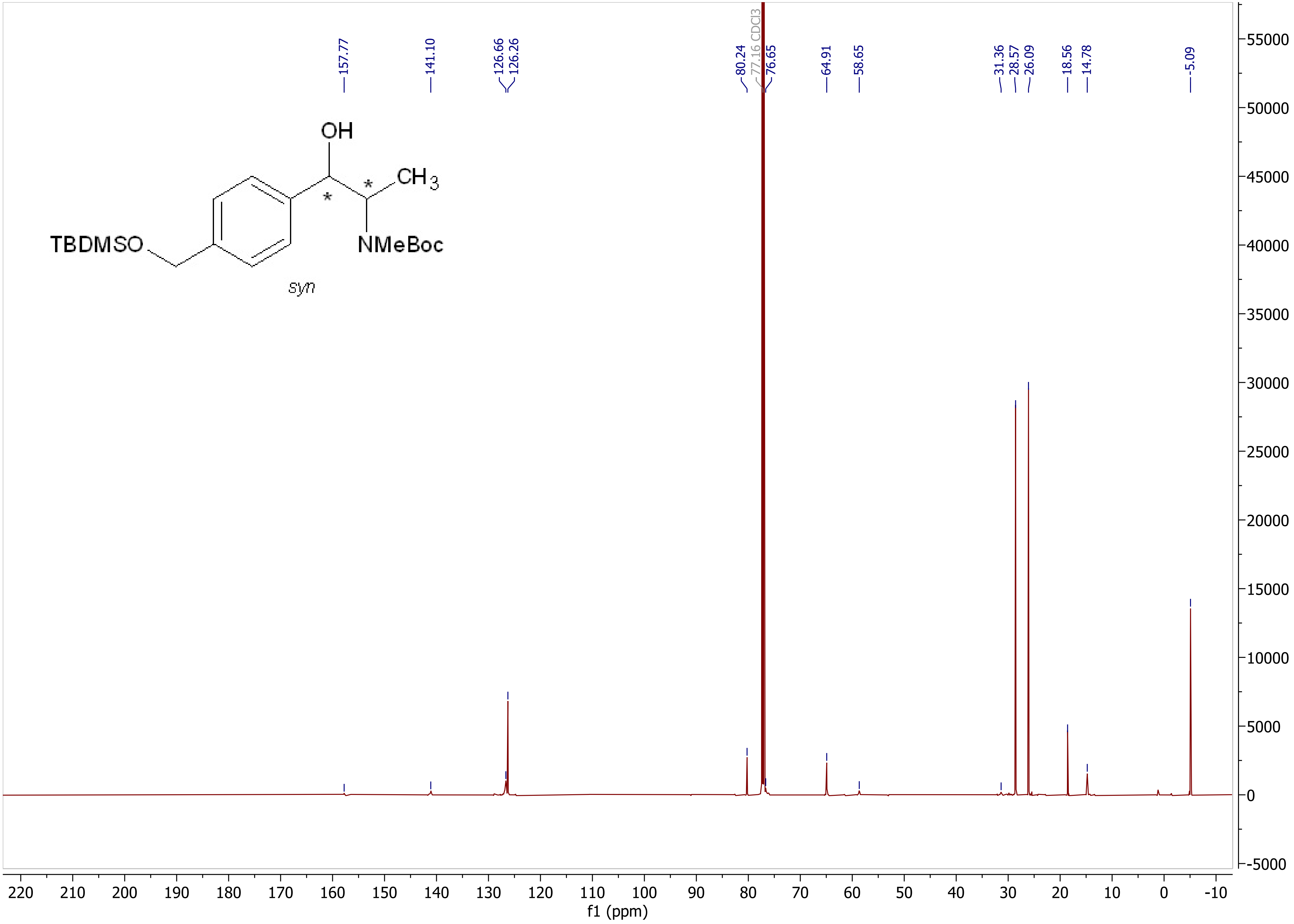
157.77
141.10
126.66
126.26
80.24
77.16 CDCl3
76.65
64.91
58.65
31.36
28.57
26.09
18.56
14.78
-5.09
OH
CH3
TBDMSO
NMeBoc
syn
f1 (ppm)

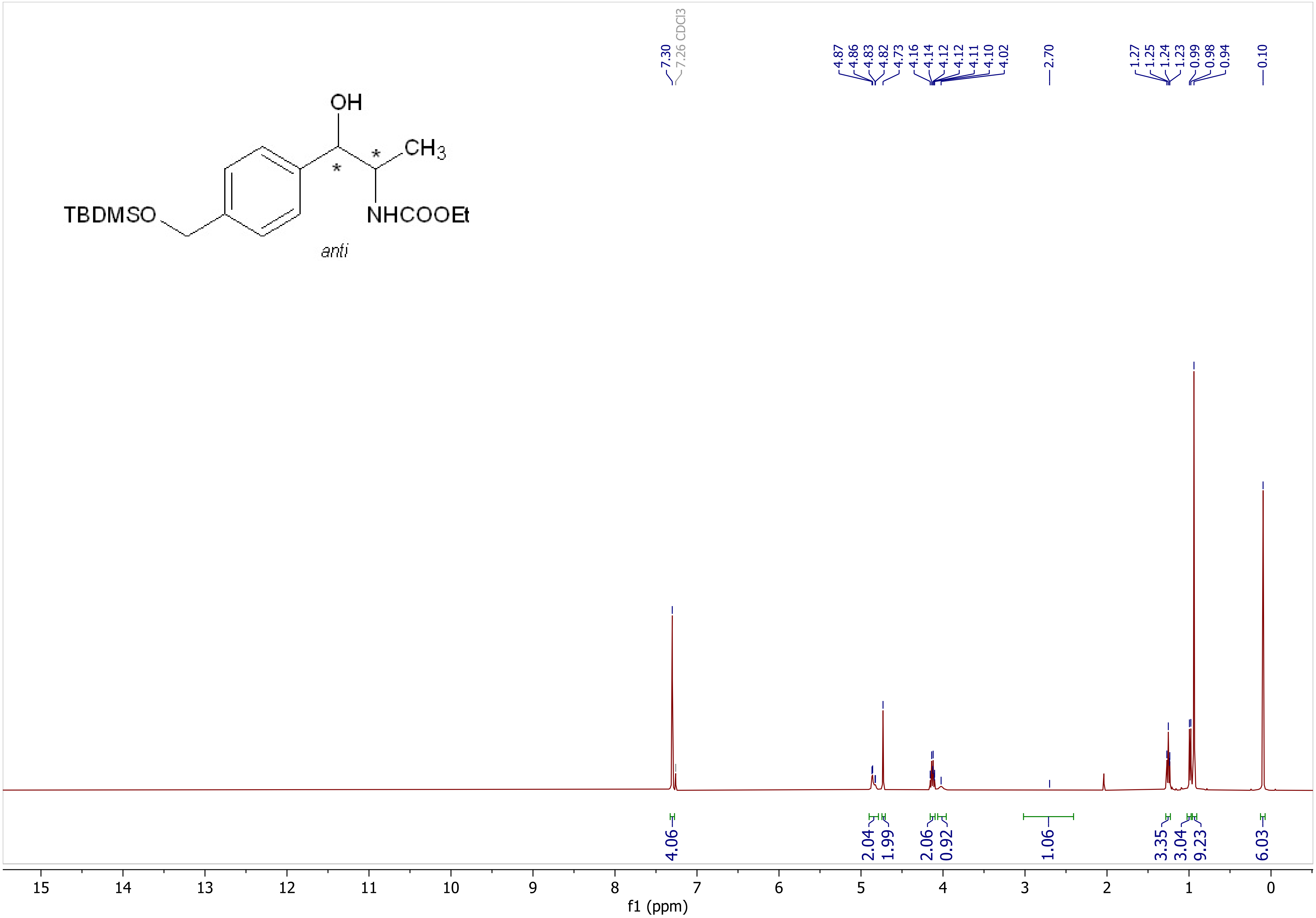

OH
CH3
TBDMSO
NHCOOEt
anti
7.30
7.26 CDCl3
4.87
4.86
4.83
4.82
4.73
4.16
4.14
4.12
4.12
4.11
4.10
4.02
2.70
1.27
1.25
1.24
1.23
0.99
0.98
0.94
0.10
4.06
2.04
1.99
2.06
0.92
1.06
3.35
3.04
9.23
6.03
15
14
13
12
11
10
9
8
7
6
5
4
3
2
1
0
f1 (ppm)

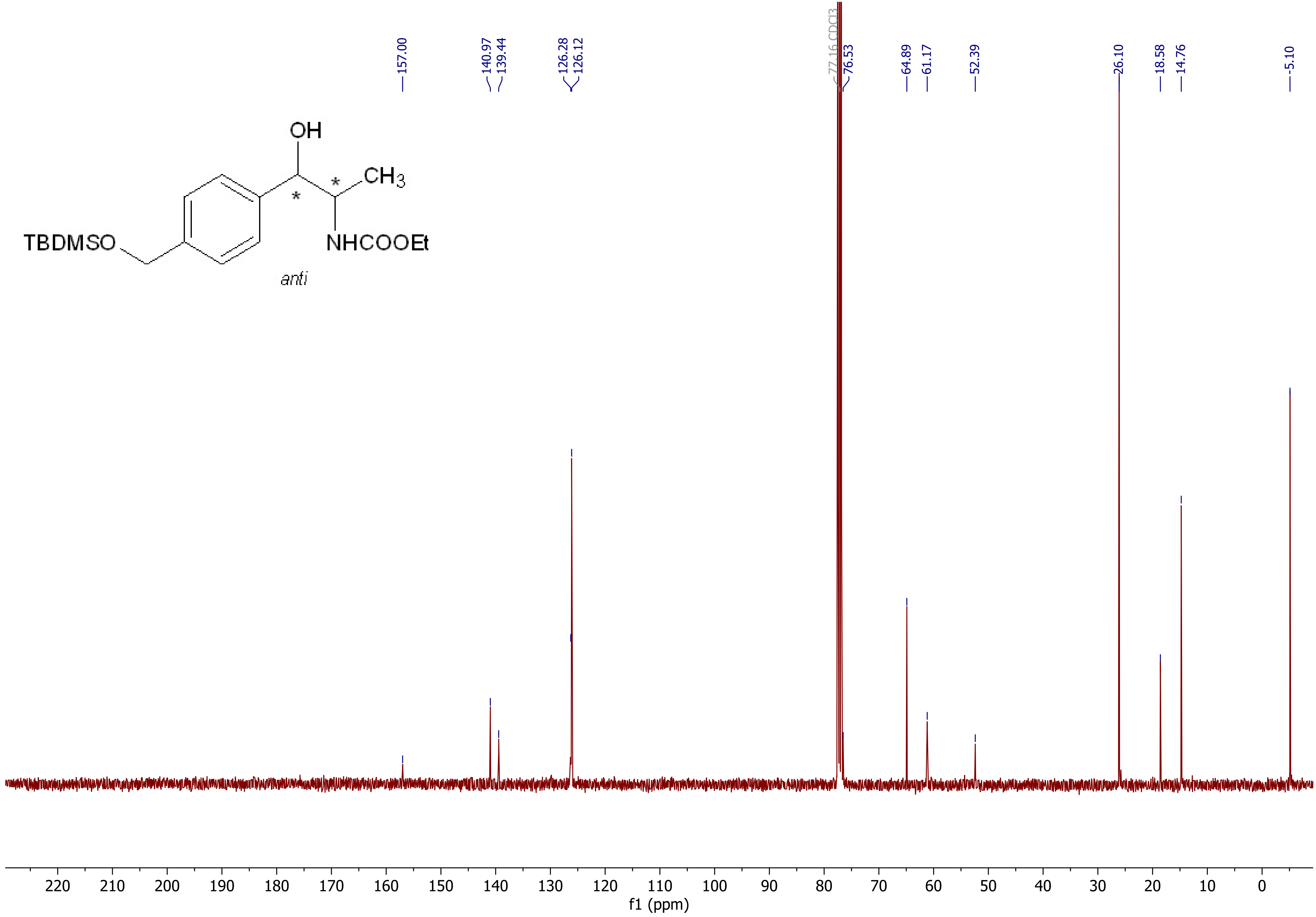

157.00
140.97
139.44
126.28
126.12
77.16 CDCl3
76.53
64.89
61.17
52.39
26.10
18.58
14.76
-5.10
OH
CH3
TBDMSO
NHCOOEt
anti
220 210 200 190 180 170 160 150 140 130 120 110 100 90 80 70 60 50 40 30 20 10 0
f1 (ppm)

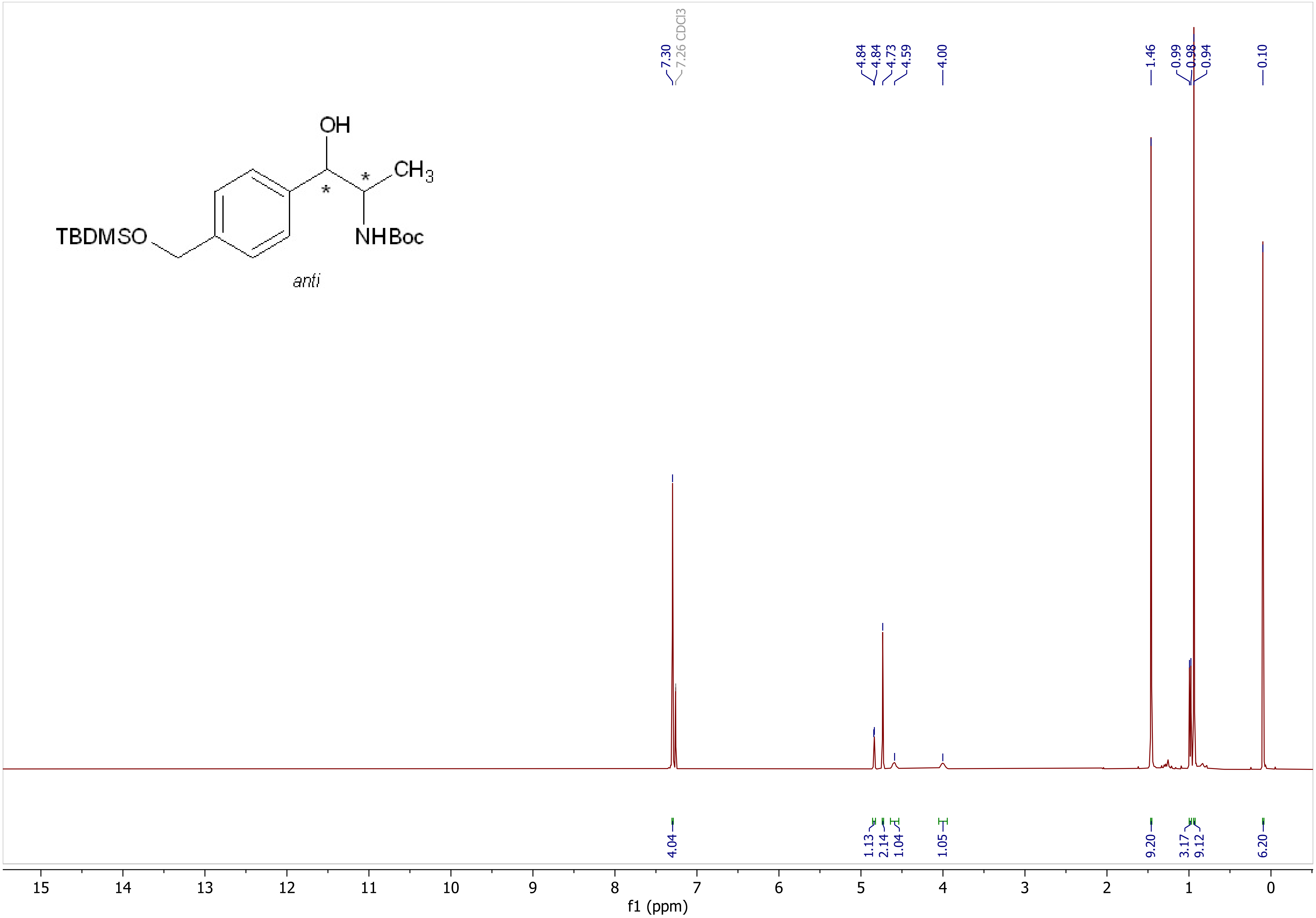

OH
CH3
TBDMSO
NHBoc
anti
7.30
7.26 CDCl3
4.84
4.84
4.73
4.59
4.00
1.46
0.99
0.98
0.94
0.10
4.04
1.13
2.14
1.04
1.05
9.20
3.17
9.12
6.20
15 14 13 12 11 10 9 8 7 6 5 4 3 2 1 0
f1 (ppm)

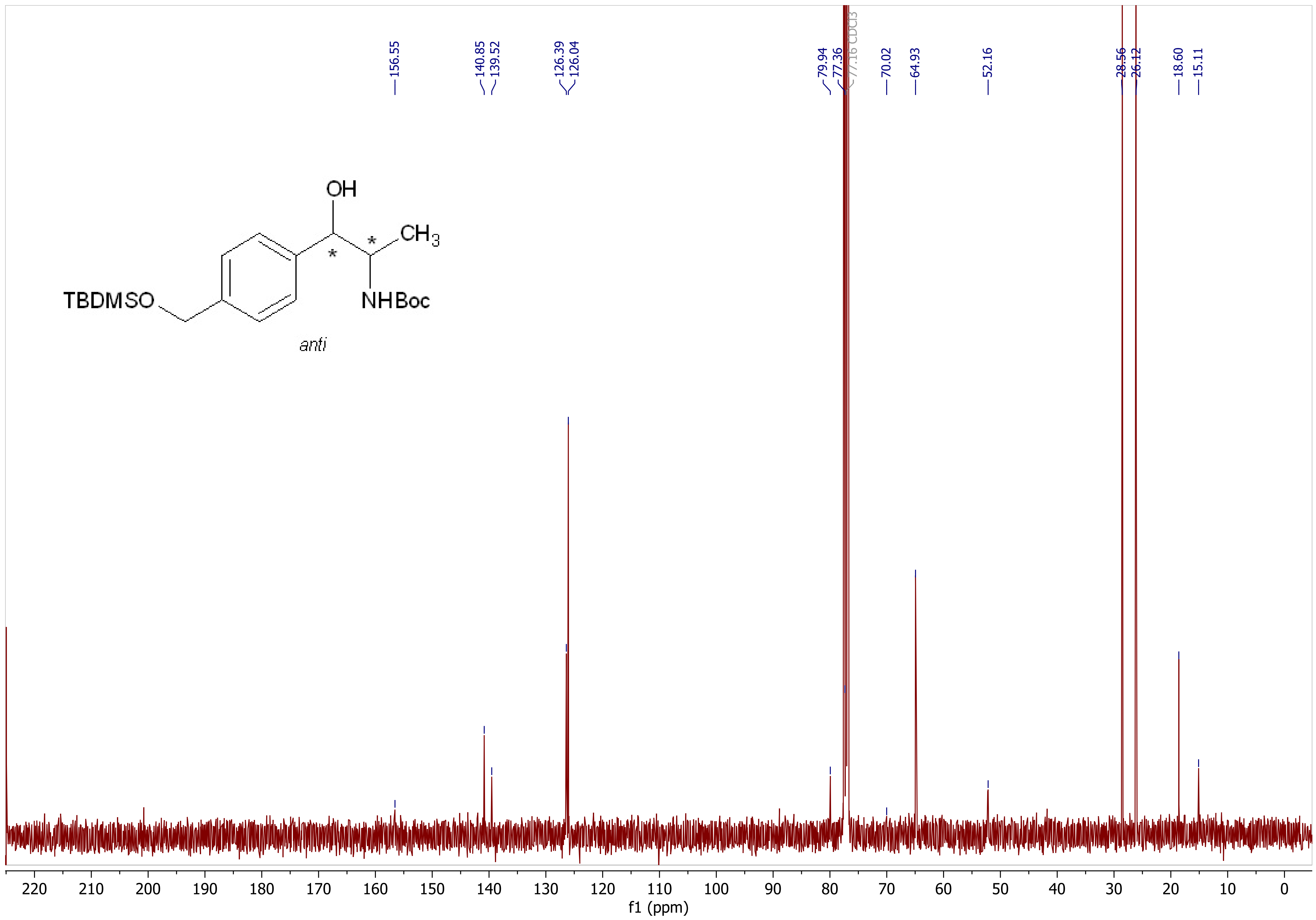
156.55
140.85
139.52
126.39
126.04
79.94
77.36
77.16 CDCl3
70.02
64.93
52.16
28.56
26.12
18.60
15.11
OH
CH3
TBDMSO
NHBoc
anti
220
210
200
190
180
170
160
150
140
130
120
110
100
90
80
70
60
50
40
30
20
10
0
f1 (ppm)

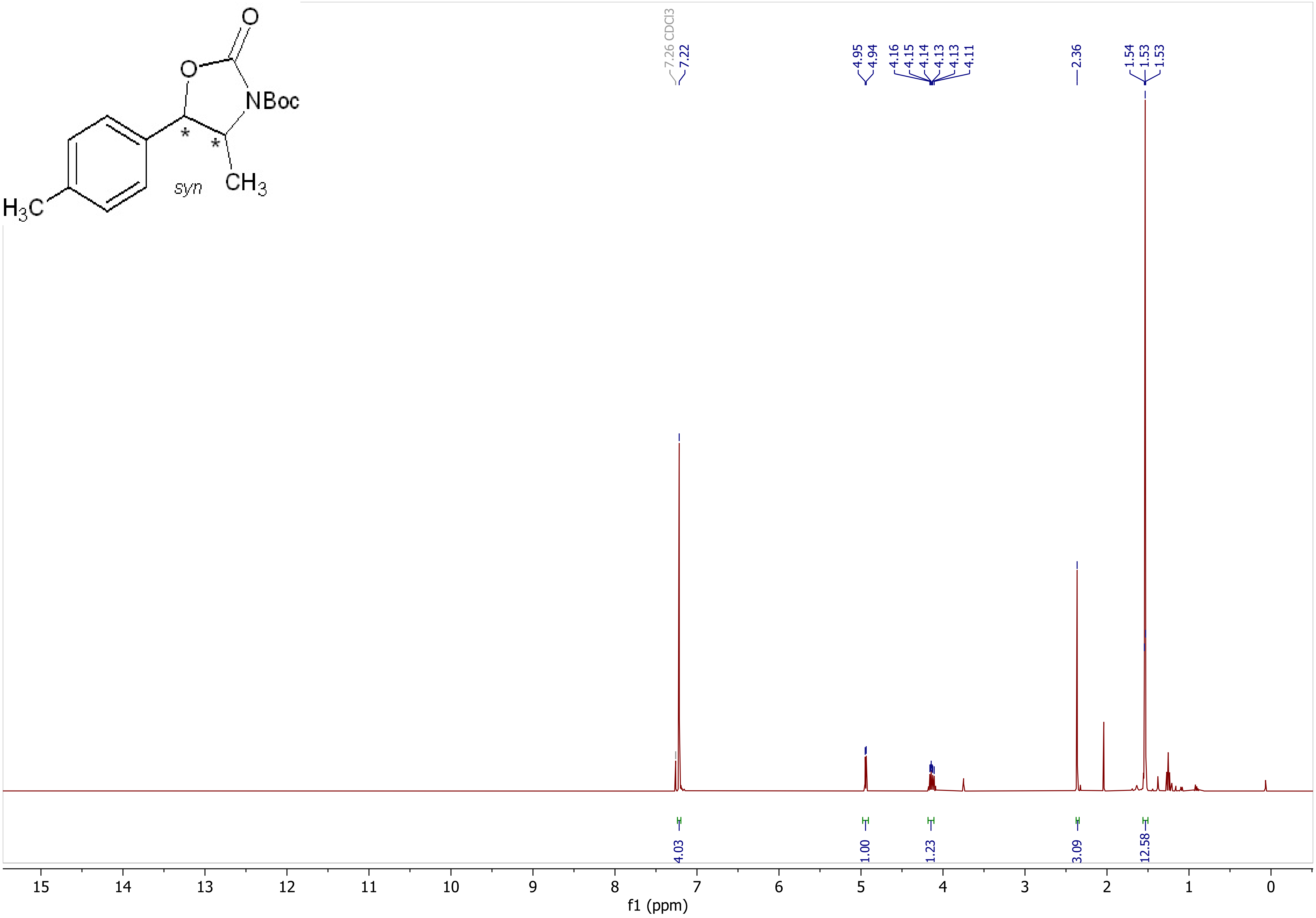

O
O
NBoc
syn
CH3
H3C
7.26 CDCl3
7.22
4.95
4.94
4.16
4.15
4.14
4.13
4.13
4.11
2.36
1.54
1.53
1.53
4.03
1.00
1.23
3.09
12.58
15
14
13
12
11
10
9
8
7
6
5
4
3
2
1
0
f1 (ppm)

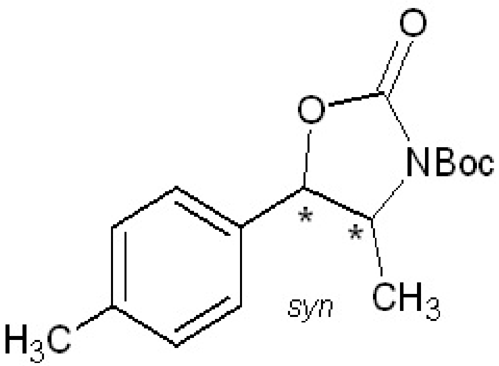

O
O
NBoc
*
*
syn
$CH_3$
$H_3C$


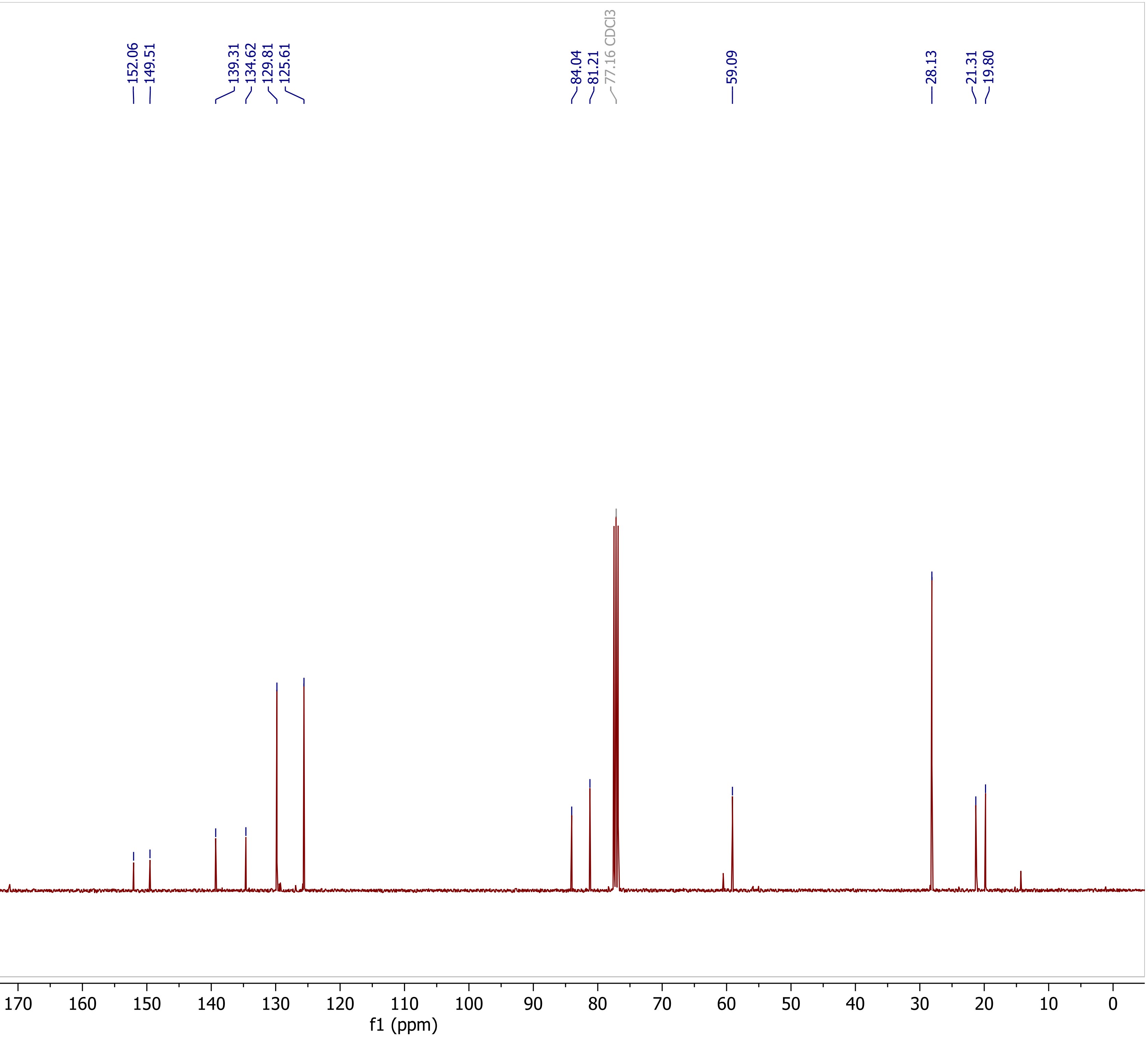

152.06
149.51
139.31
134.62
129.81
125.61
84.04
81.21
77.16 CDCl3
59.09
28.13
21.31
19.80
220 210 200 190 180 170 160 150 140 130 120 110 100 90 80 70 60 50 40 30 20 10 0
f1 (ppm)

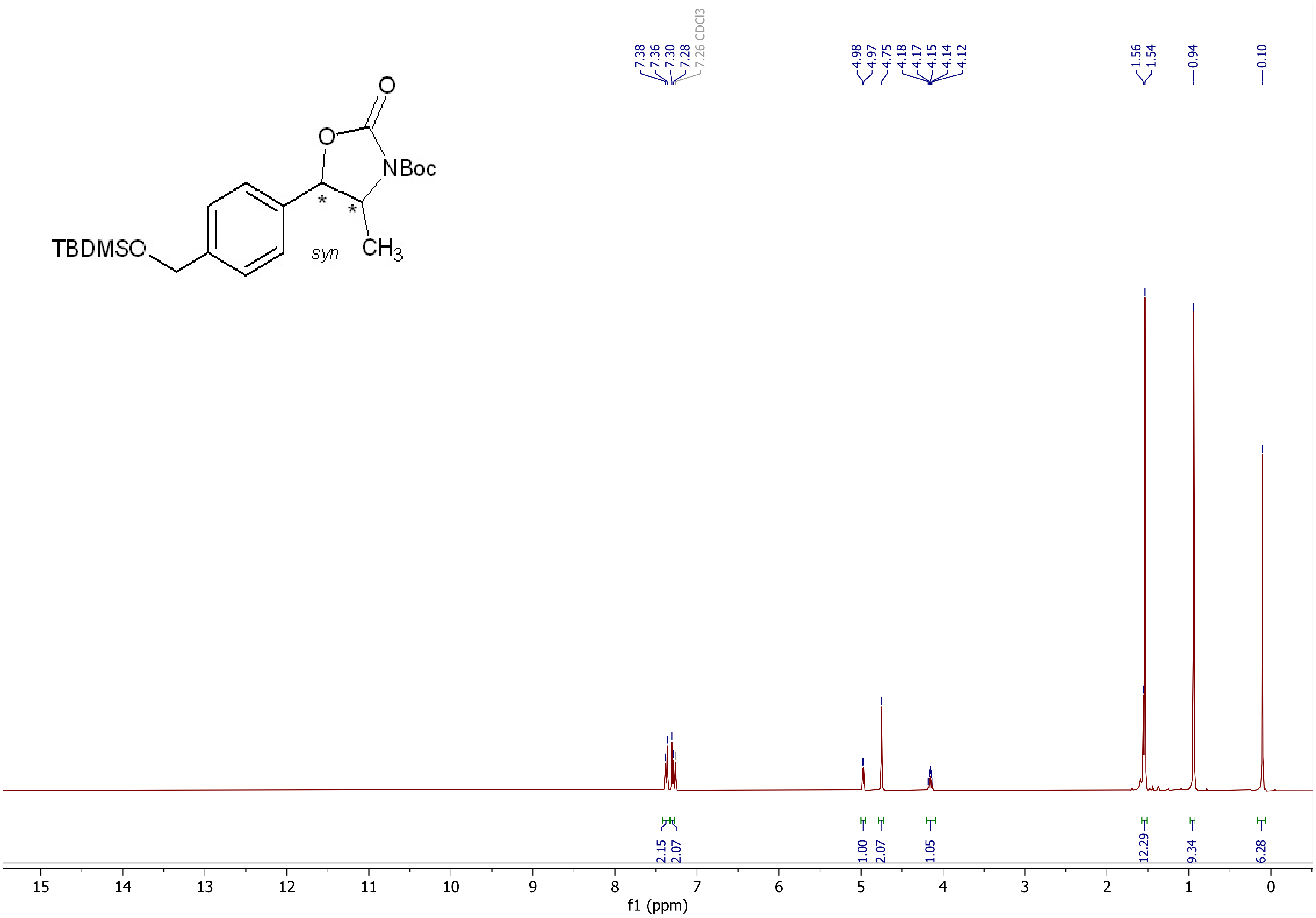

TBDMSO
NBoc
O
O
CH3
syn
7.38
7.36
7.30
7.28
7.26 CDCl3
4.98
4.97
4.75
4.18
4.17
4.15
4.14
4.12
1.56
1.54
0.94
0.10
2.15
2.07
1.00
2.07
1.05
12.29
9.34
6.28
15
14
13
12
11
10
9
8
7
6
5
4
3
2
1
0
f1 (ppm)

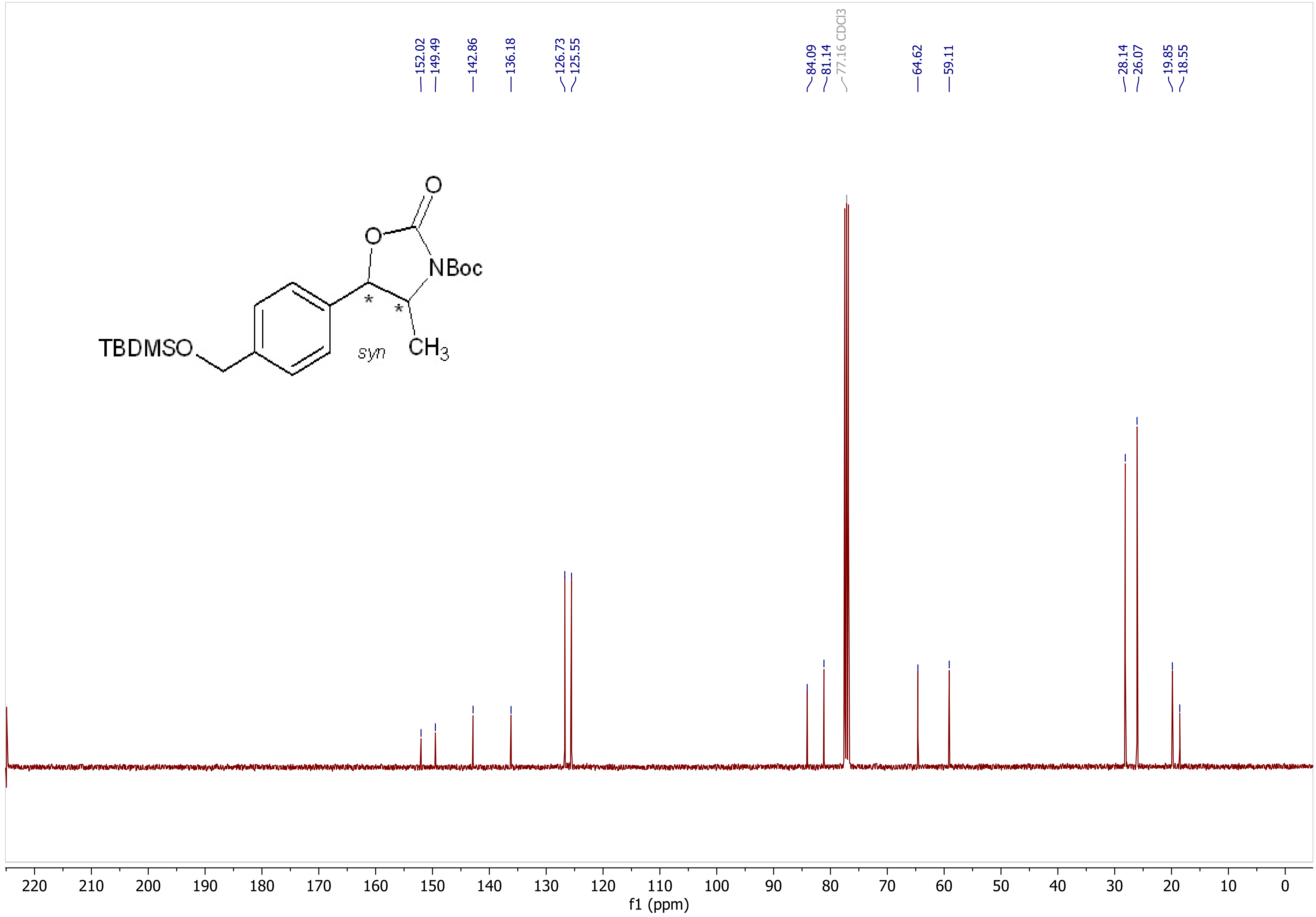

152.02
149.49
142.86
136.18
126.73
125.55
84.09
81.14
77.16 CDCl3
64.62
59.11
28.14
26.07
19.85
18.55
O
O
NBoc
TBDMSO
syn
CH3
220 210 200 190 180 170 160 150 140 130 120 110 100 90 80 70 60 50 40 30 20 10 0
f1 (ppm)

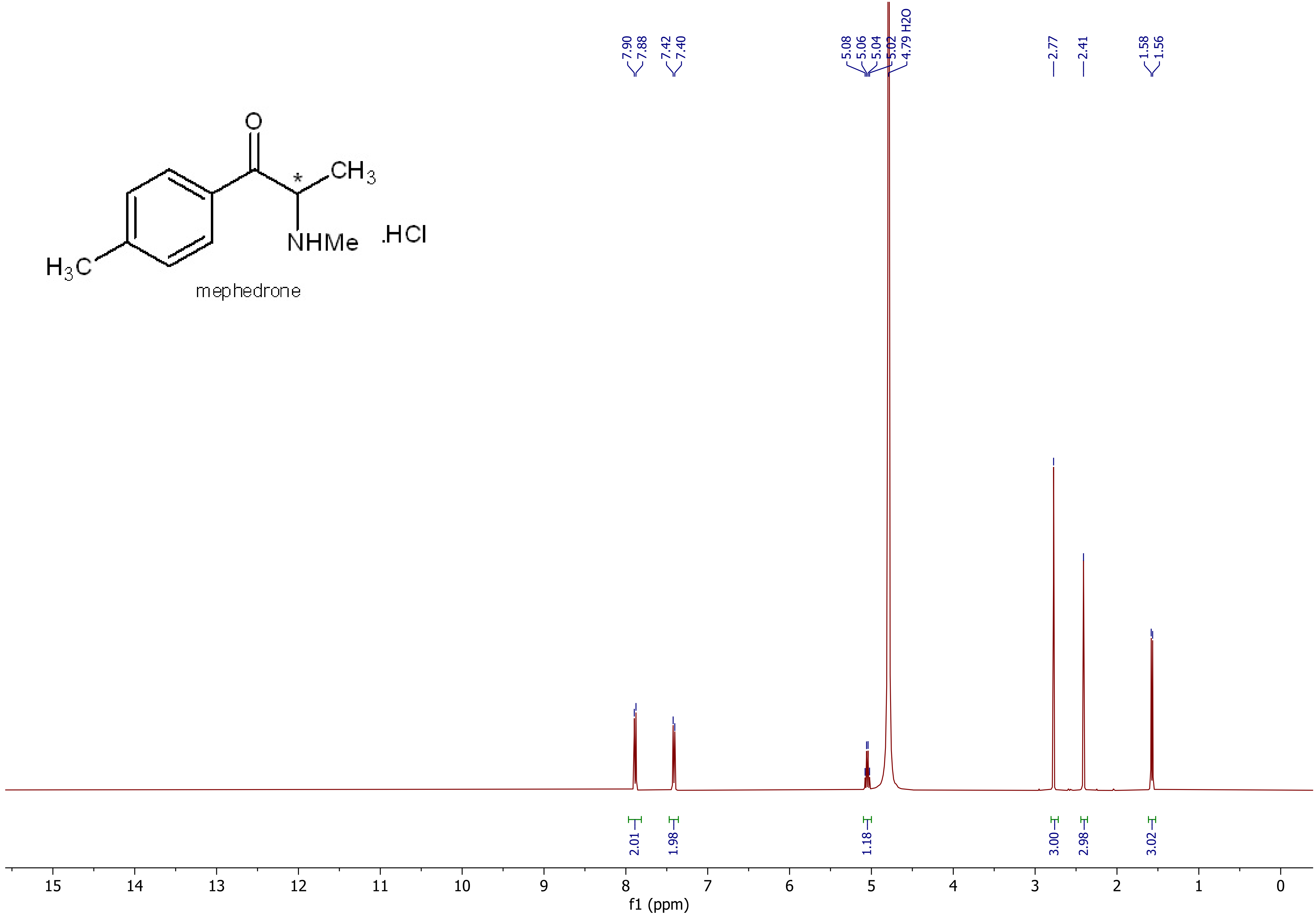

O
CH3
NHMe
.HCl
H3C
mephedrone
7.90
7.88
7.42
7.40
5.08
5.06
5.04
5.02
4.79 H2O
2.77
2.41
1.58
1.56
2.01
1.98
1.18
3.00
2.98
3.02
15
14
13
12
11
10
9
8
7
6
5
4
3
2
1
0
f1 (ppm)

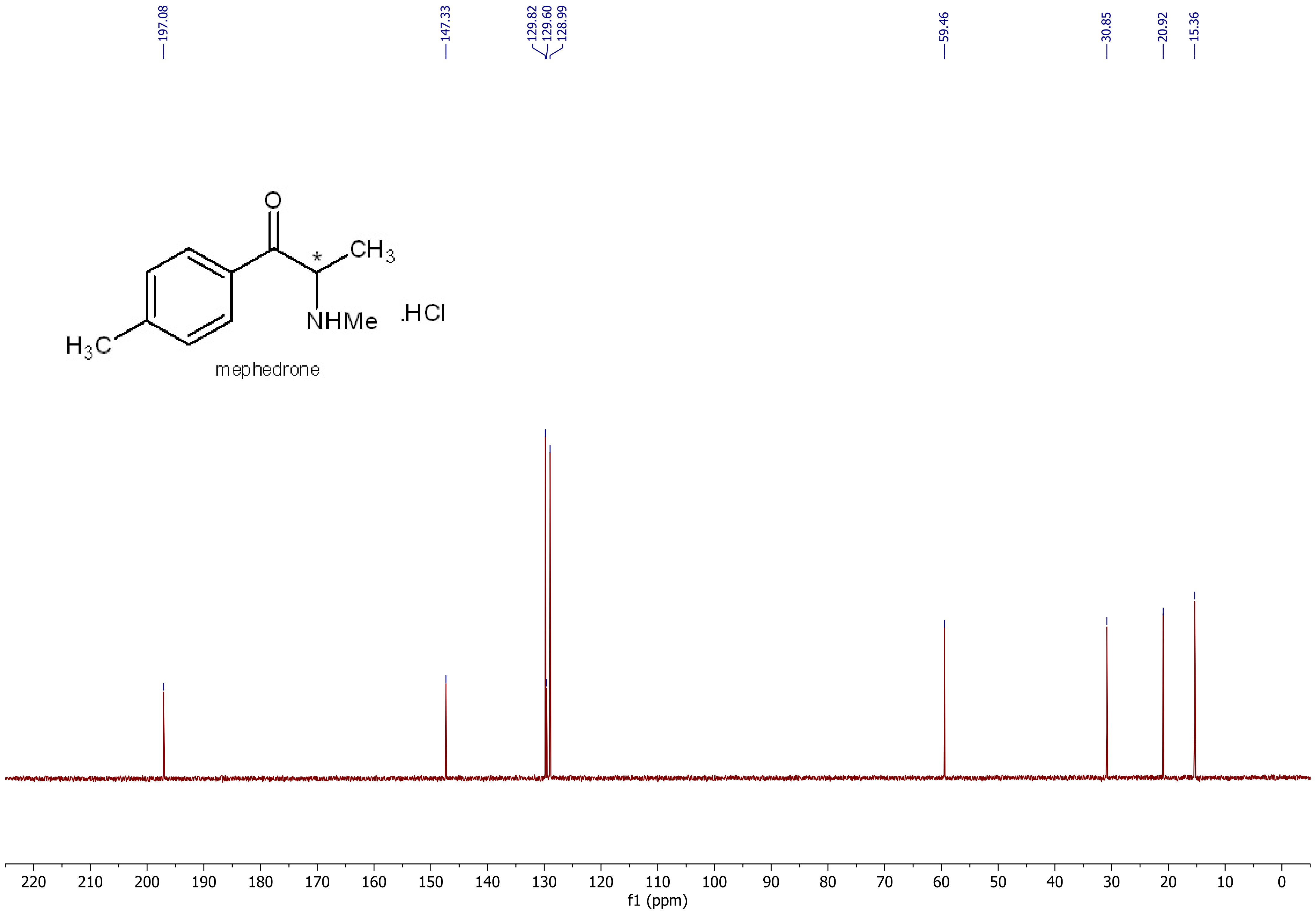
197.08
147.33
129.82
129.60
128.99
59.46
30.85
20.92
15.36
O
CH3
H3C
NHMe
.HCl
*
mephedrone
220 210 200 190 180 170 160 150 140 130 120 110 100 90 80 70 60 50 40 30 20 10 0
f1 (ppm)

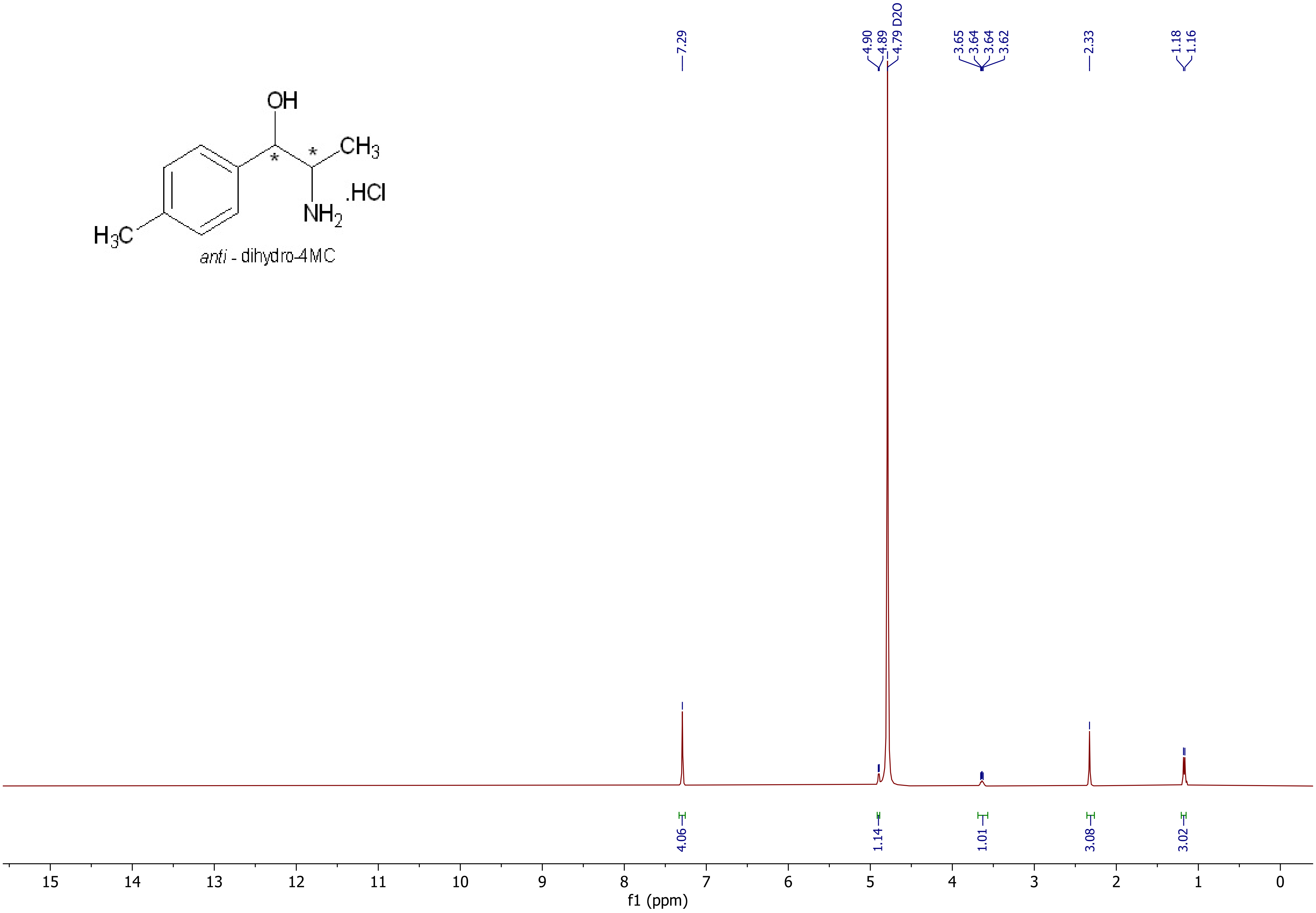

OH
$CH_3$
.HCl
$NH_2$
$H_3C$
*anti* - dihydro-4MC
7.29
4.90
4.89
4.79 D2O
3.65
3.64
3.64
3.62
2.33
1.18
1.16
4.06
1.14
1.01
3.08
3.02
15 14 13 12 11 10 9 8 7 6 5 4 3 2 1 0
f1 (ppm)

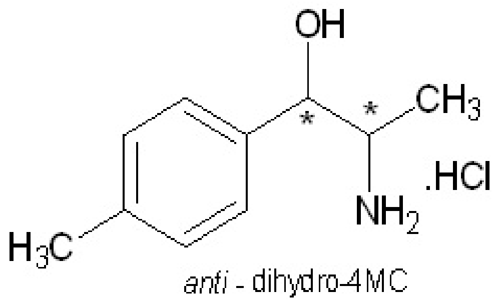


*anti* - dihydro-4MC

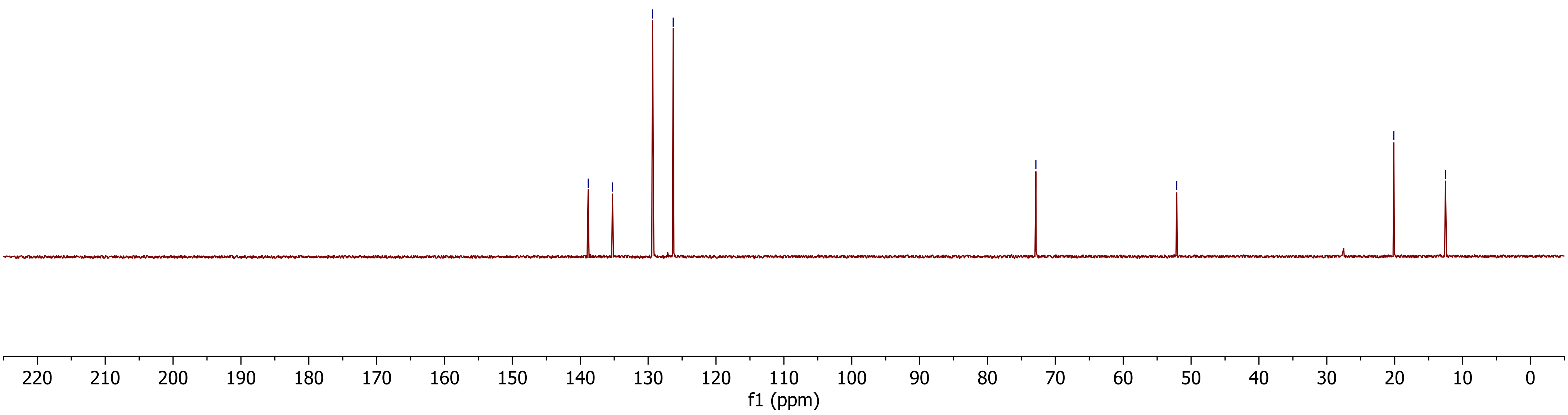

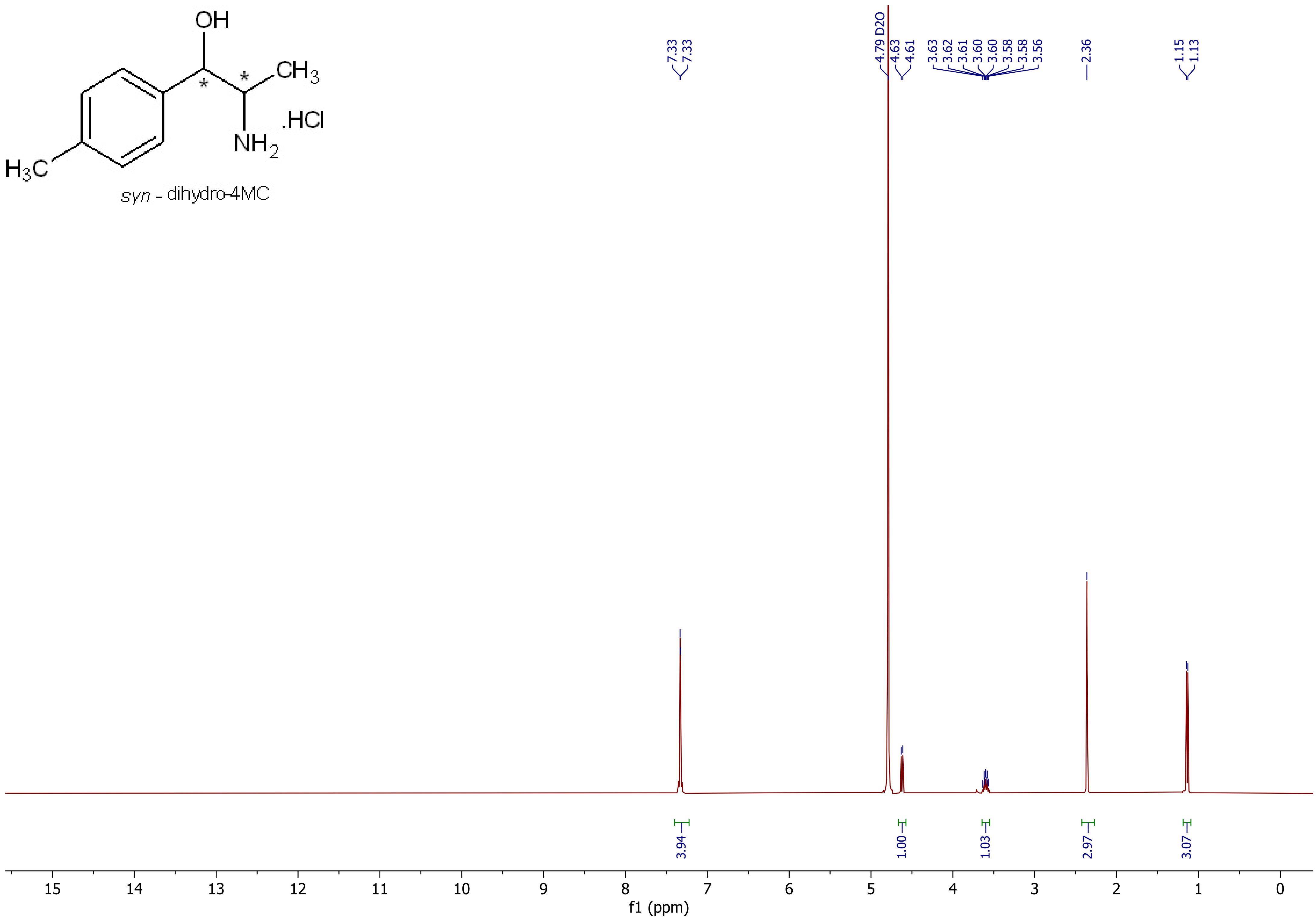
OH
CH3
.HCl
NH2
H3C
syn - dihydro-4MC
7.33
7.33
4.79 D2O
4.63
4.61
3.63
3.62
3.61
3.60
3.60
3.58
3.58
3.56
2.36
1.15
1.13
3.94
1.00
1.03
2.97
3.07
15
14
13
12
11
10
9
8
7
6
5
4
3
2
1
0
f1 (ppm)

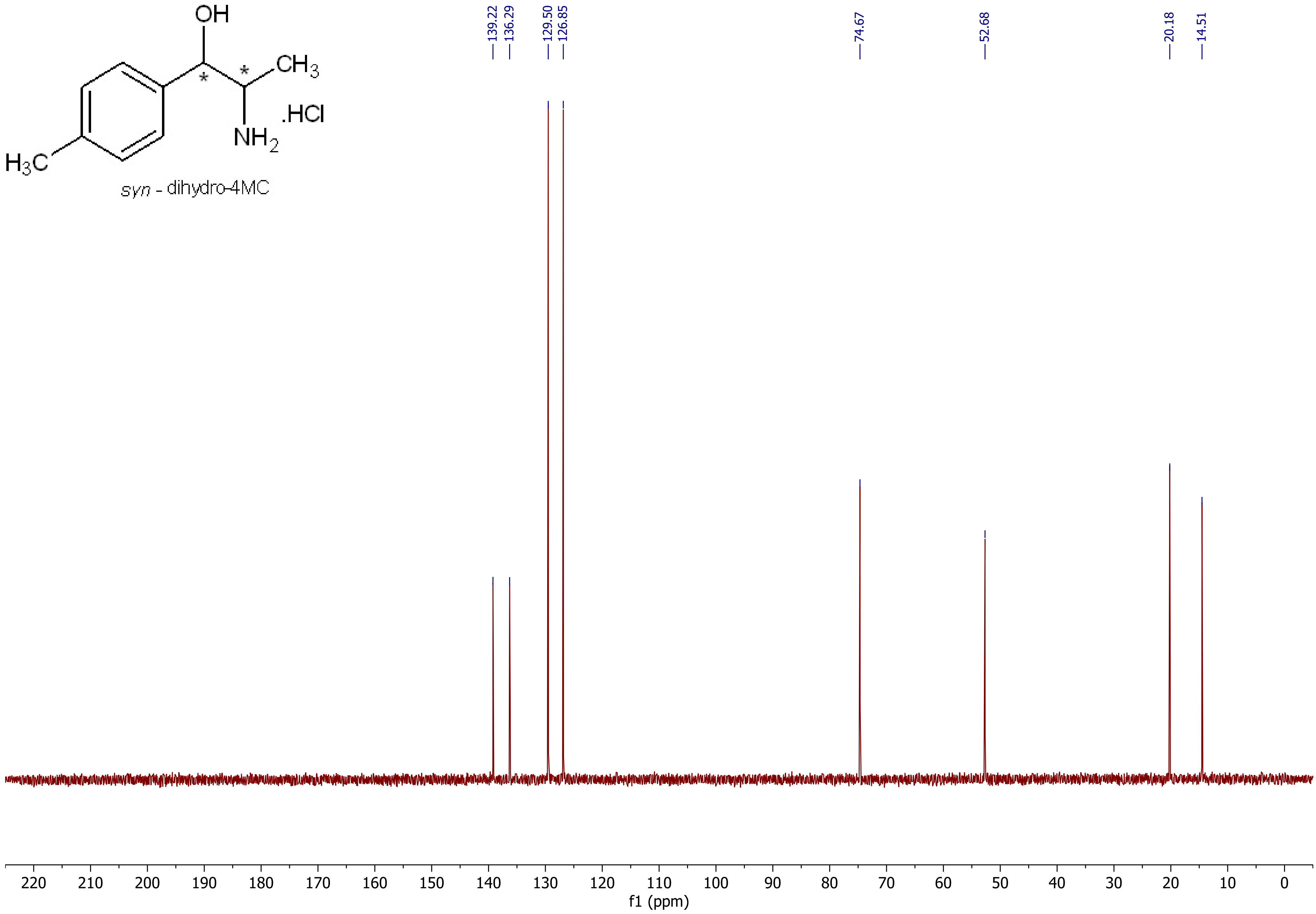

OH
CH3
.HCl
NH2
H3C
syn - dihydro-4MC
139.22
136.29
129.50
126.85
74.67
52.68
20.18
14.51
220 210 200 190 180 170 160 150 140 130 120 110 100 90 80 70 60 50 40 30 20 10 0
f1 (ppm)

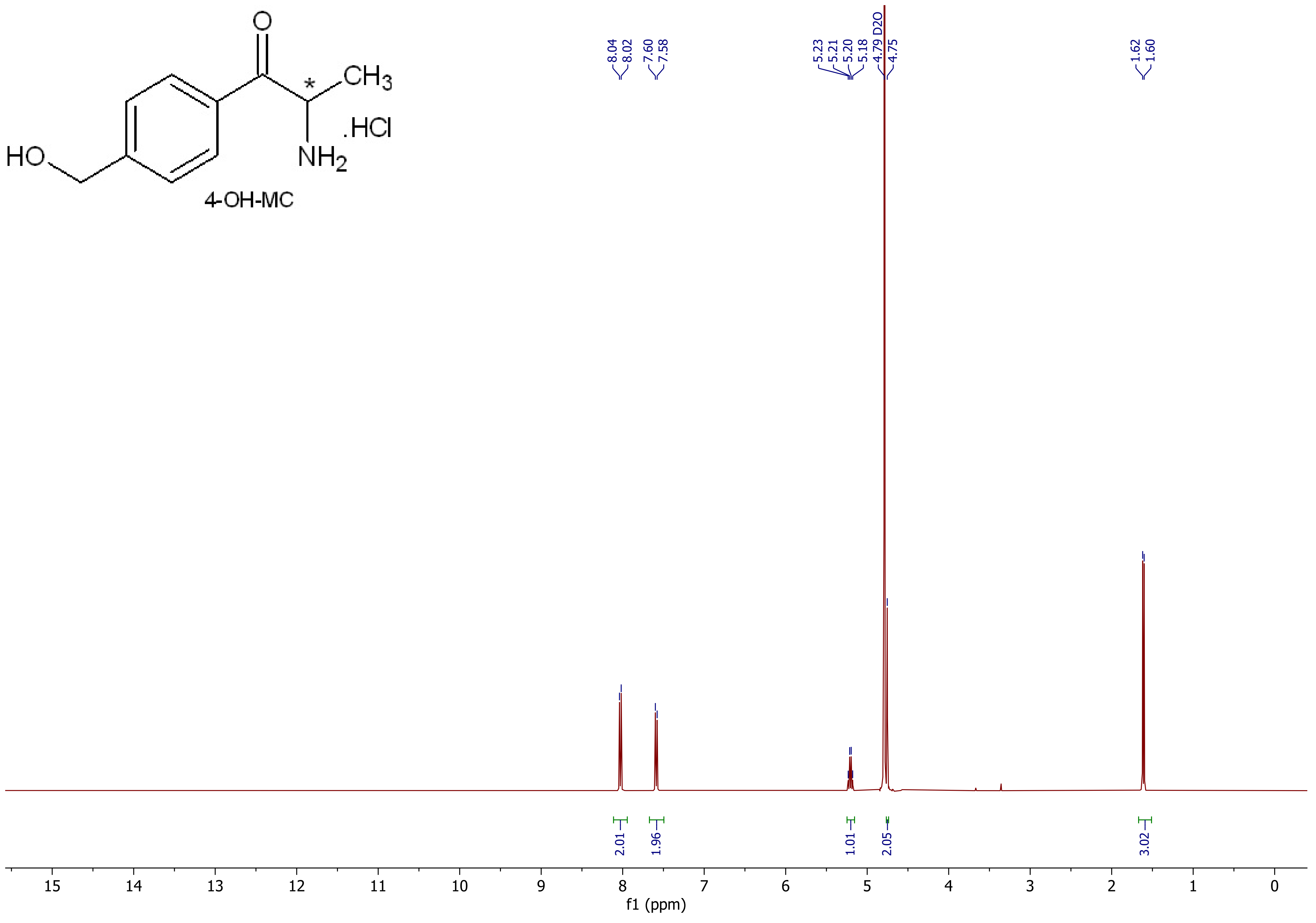

O
CH3
*
.HCl
HO
NH2
4-OH-MC
8.04
8.02
7.60
7.58
5.23
5.21
5.20
5.18
4.79 D2O
4.75
1.62
1.60
2.01
1.96
1.01
2.05
3.02
15 14 13 12 11 10 9 8 7 6 5 4 3 2 1 0
f1 (ppm)

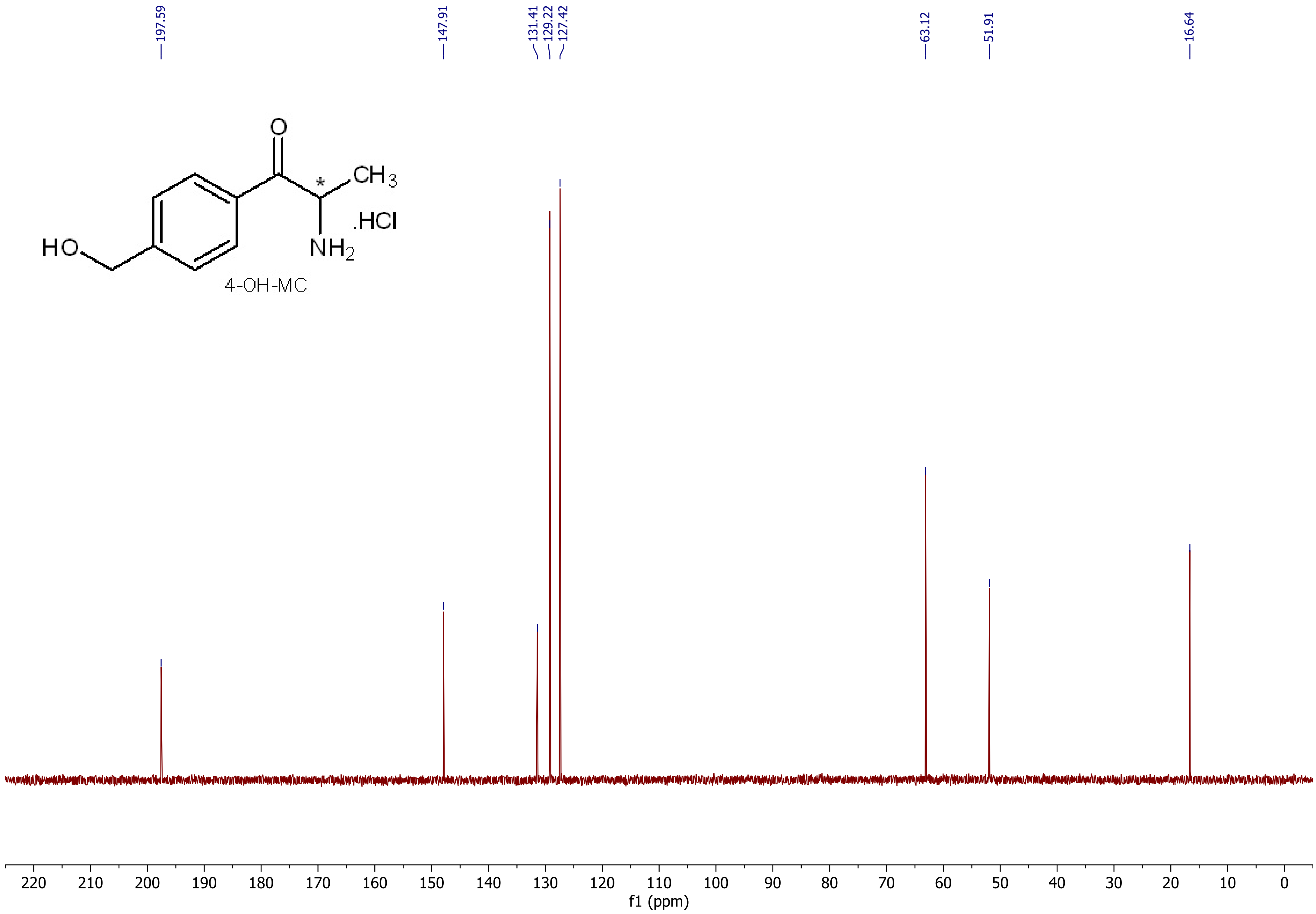

197.59
147.91
131.41
129.22
127.42
63.12
51.91
16.64
CH3
.HCl
HO
NH2
O
4-OH-MC
220 210 200 190 180 170 160 150 140 130 120 110 100 90 80 70 60 50 40 30 20 10 0
f1 (ppm)

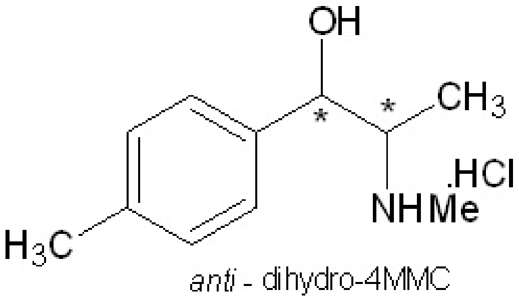

OH
CH3
.HCl
NH Me
H3C
anti - dihydro-4MMC


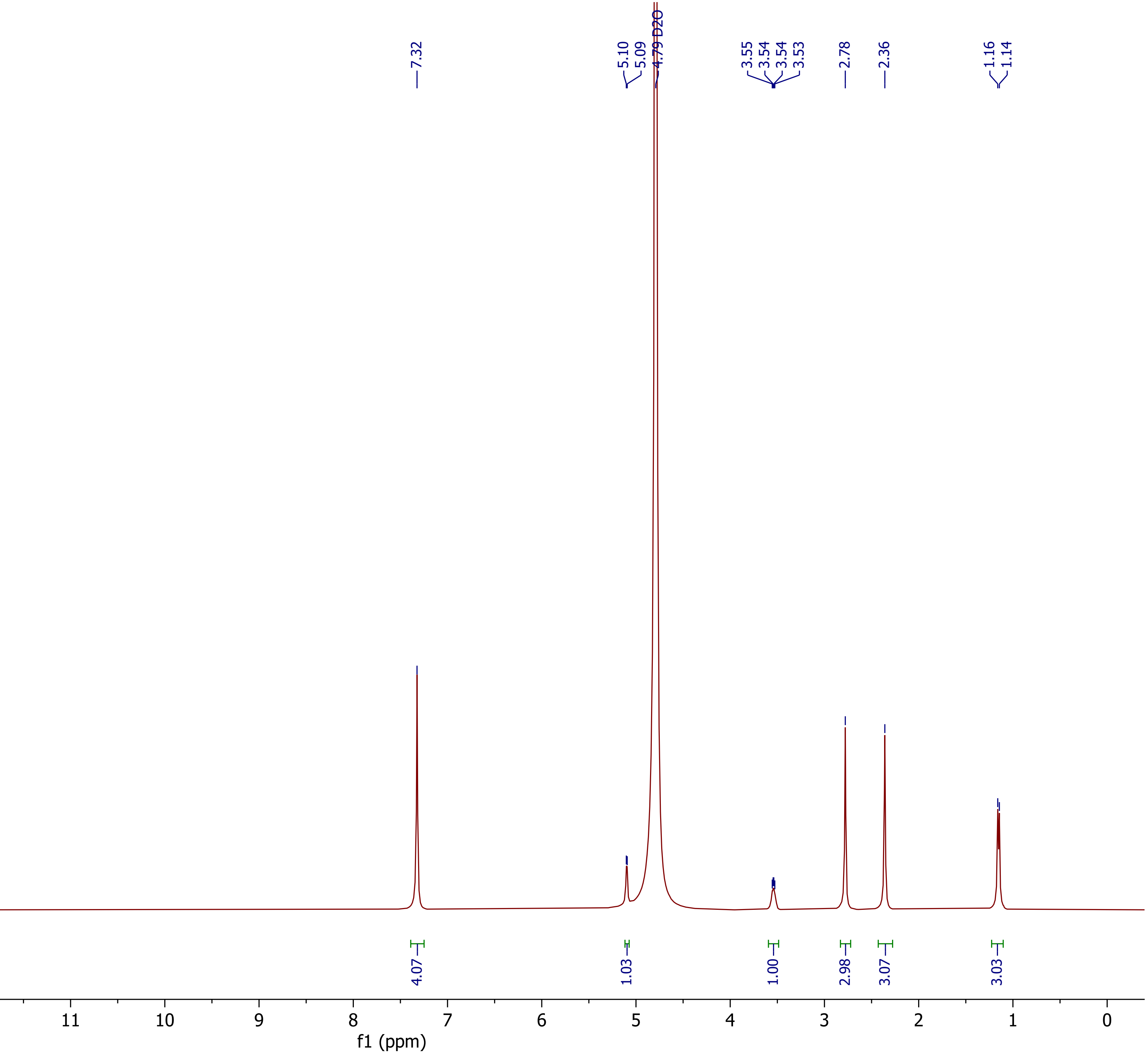

7.32
5.10
5.09
4.79 D2O
3.55
3.54
3.54
3.53
2.78
2.36
1.16
1.14
4.07
1.03
1.00
2.98
3.07
3.03
15
14
13
12
11
10
9
8
7
6
5
4
3
2
1
0
f1 (ppm)

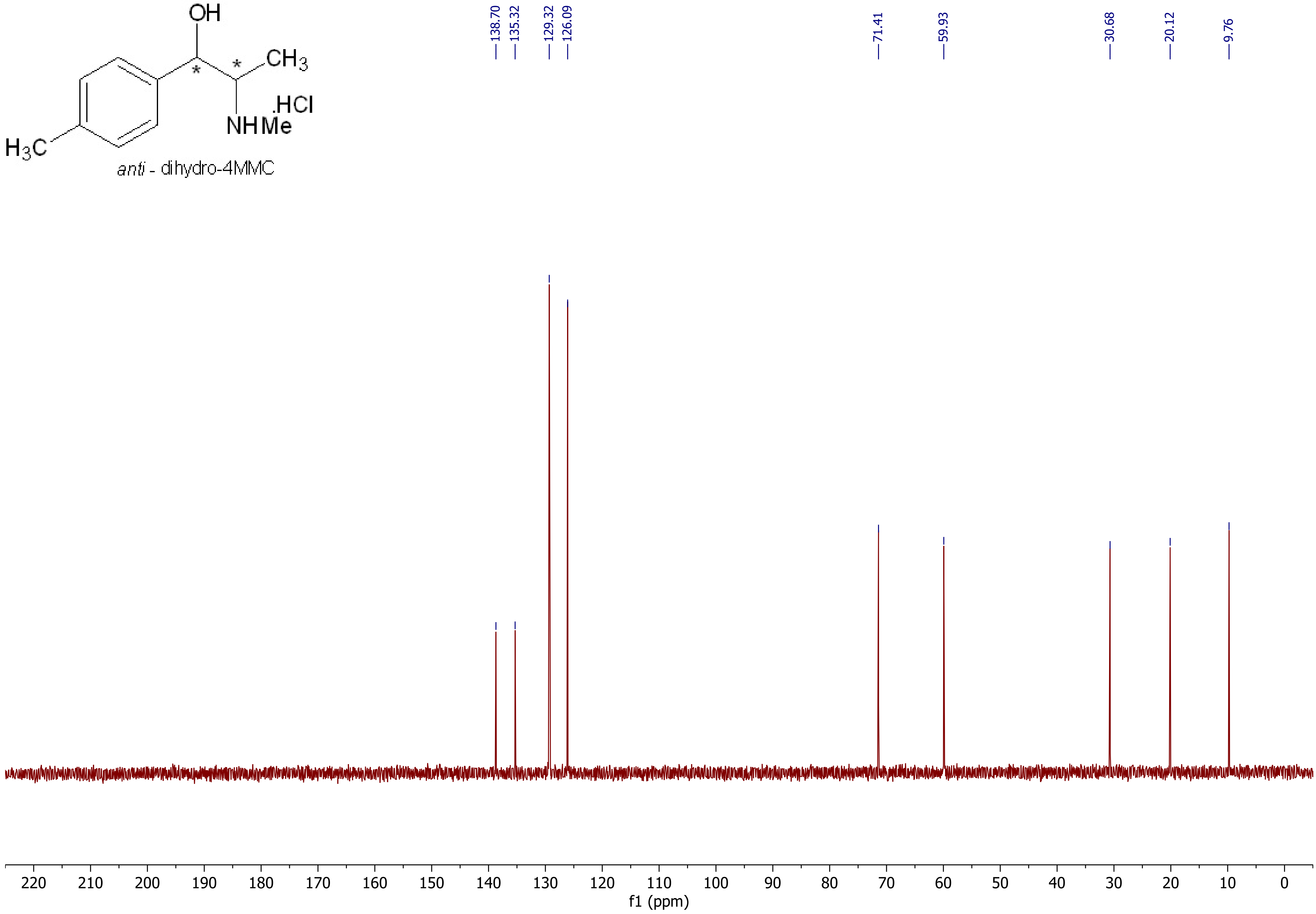
OH
CH3
.HCl
NH Me
H3C
anti - dihydro-4MMC
138.70
135.32
129.32
126.09
71.41
59.93
30.68
20.12
9.76
220 210 200 190 180 170 160 150 140 130 120 110 100 90 80 70 60 50 40 30 20 10 0
f1 (ppm)

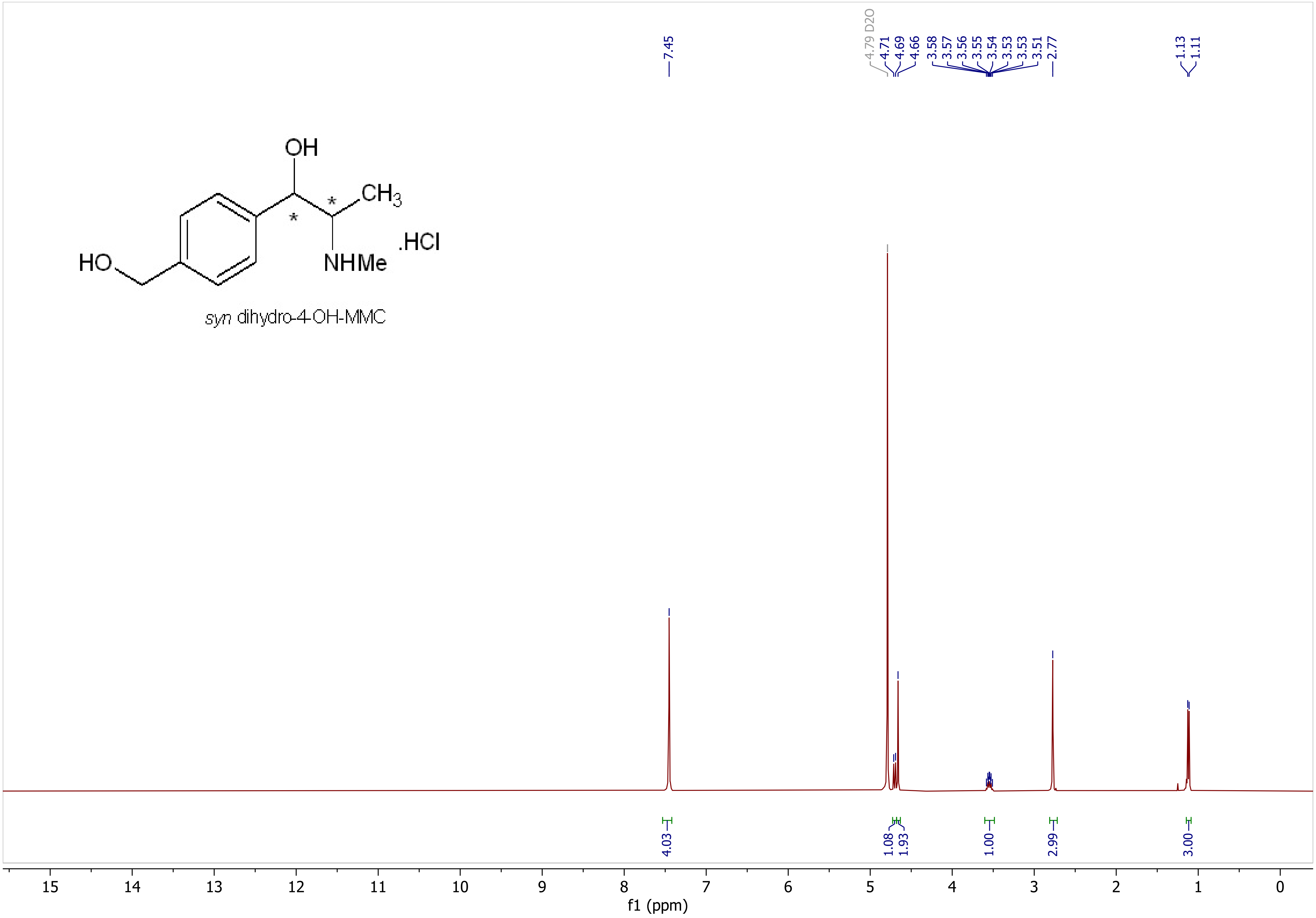

OH
CH3
HO
NHMe
.HCl
syn dihydro-4-OH-MMC
7.45
4.79 D2O
4.71
4.69
4.66
3.58
3.57
3.56
3.55
3.54
3.53
3.53
3.51
2.77
1.13
1.11
4.03
1.08
1.93
1.00
2.99
3.00
15 14 13 12 11 10 9 8 7 6 5 4 3 2 1 0
f1 (ppm)

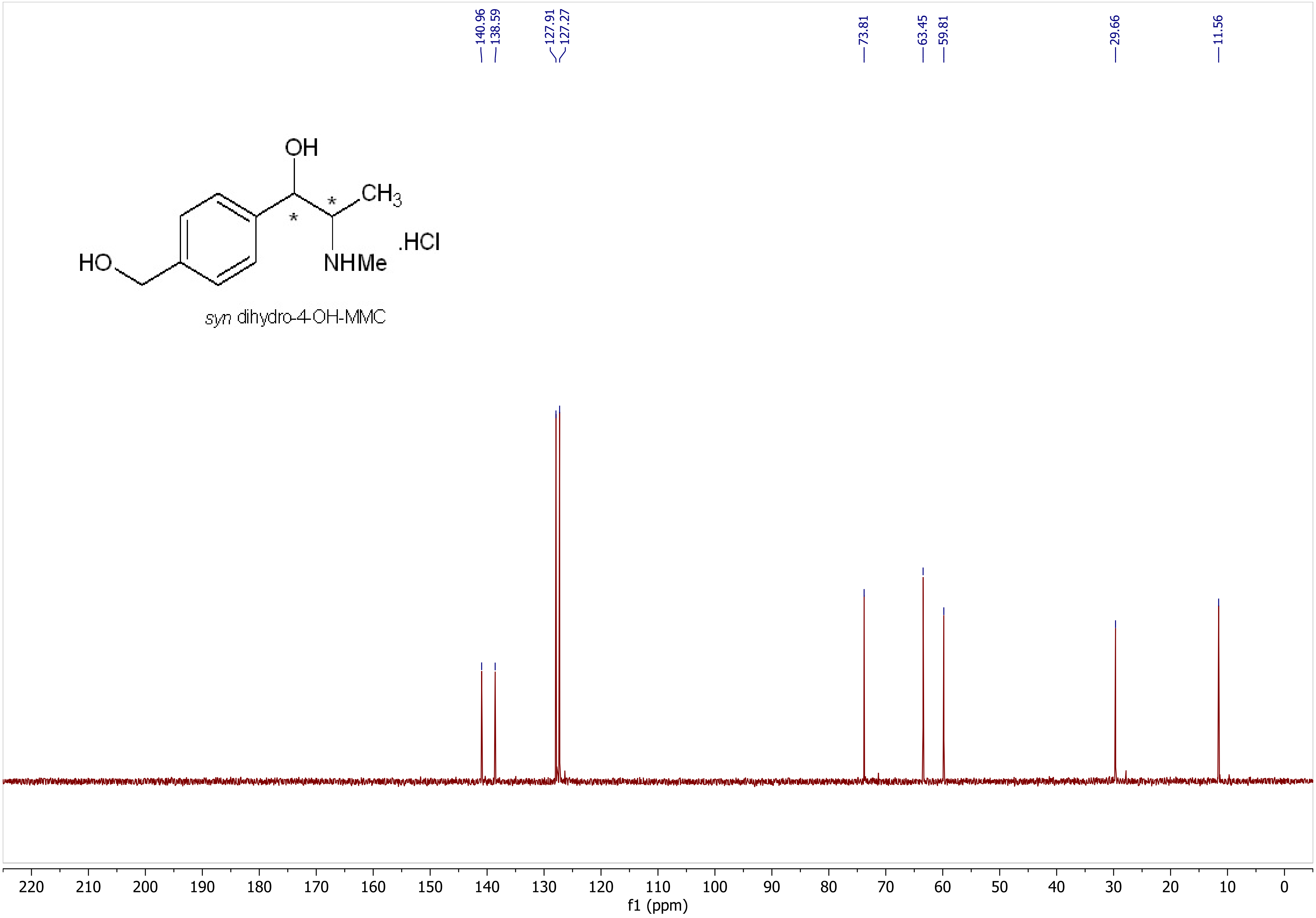
140.96
138.59
127.91
127.27
73.81
63.45
59.81
29.66
11.56
OH
CH3
HO
NHMe
.HCl
syn dihydro-4-OH-MMC
220 210 200 190 180 170 160 150 140 130 120 110 100 90 80 70 60 50 40 30 20 10 0
f1 (ppm)

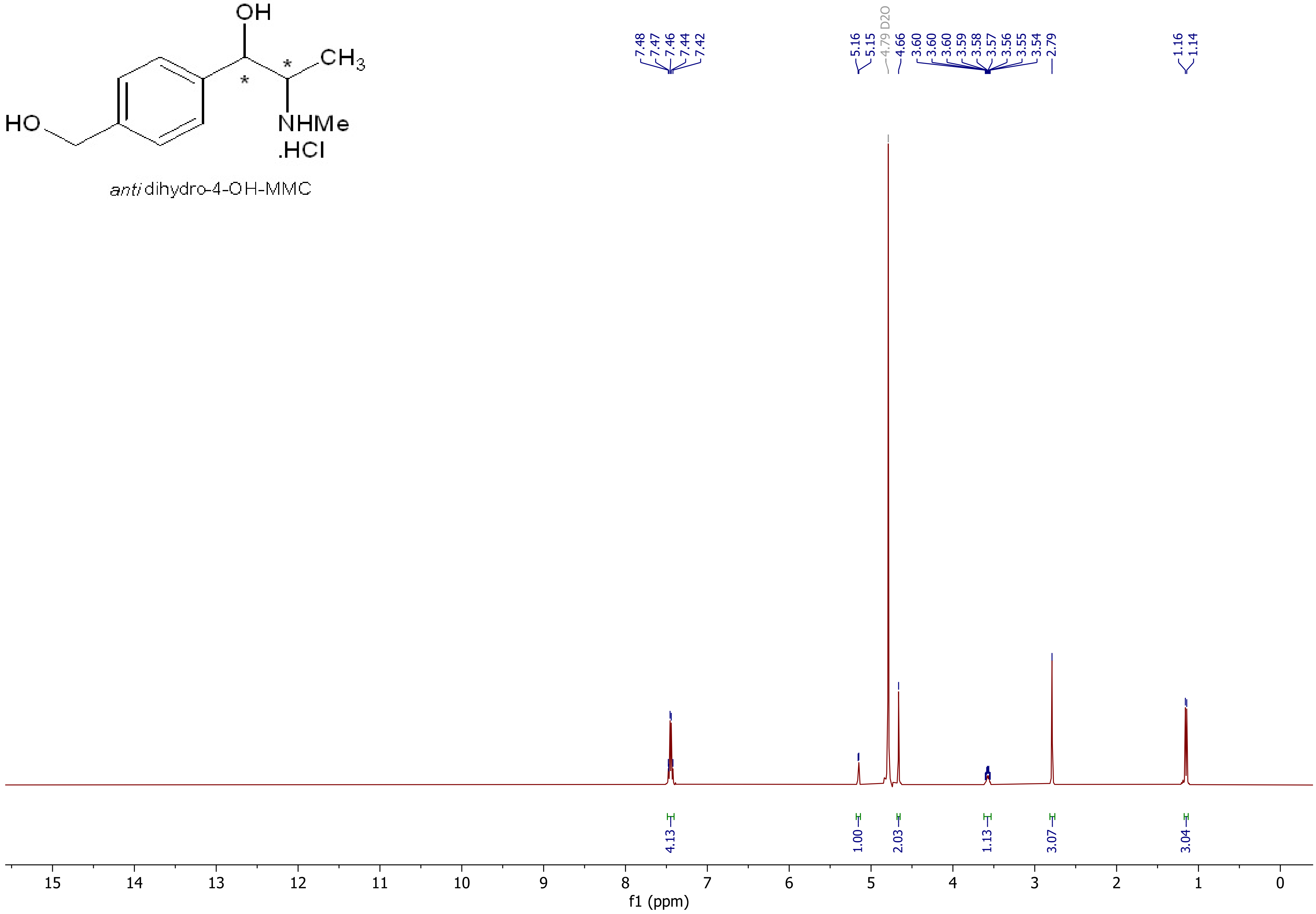
OH
CH3
HO
NHMe
.HCl
anti dihydro-4-OH-MMC
7.48
7.47
7.46
7.44
7.42
5.16
5.15
4.79 D2O
4.66
3.60
3.60
3.60
3.59
3.58
3.57
3.56
3.55
3.54
2.79
1.16
1.14
4.13
1.00
2.03
1.13
3.07
3.04
15
14
13
12
11
10
9
8
7
6
5
4
3
2
1
0
f1 (ppm)

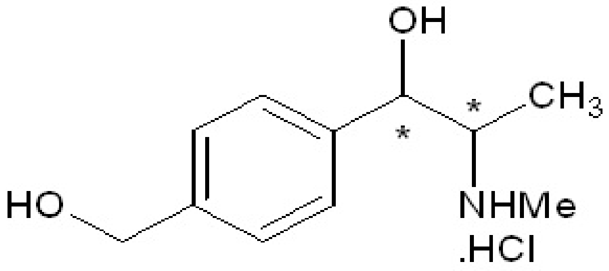


*anti* dihydro-4-OH-MMC

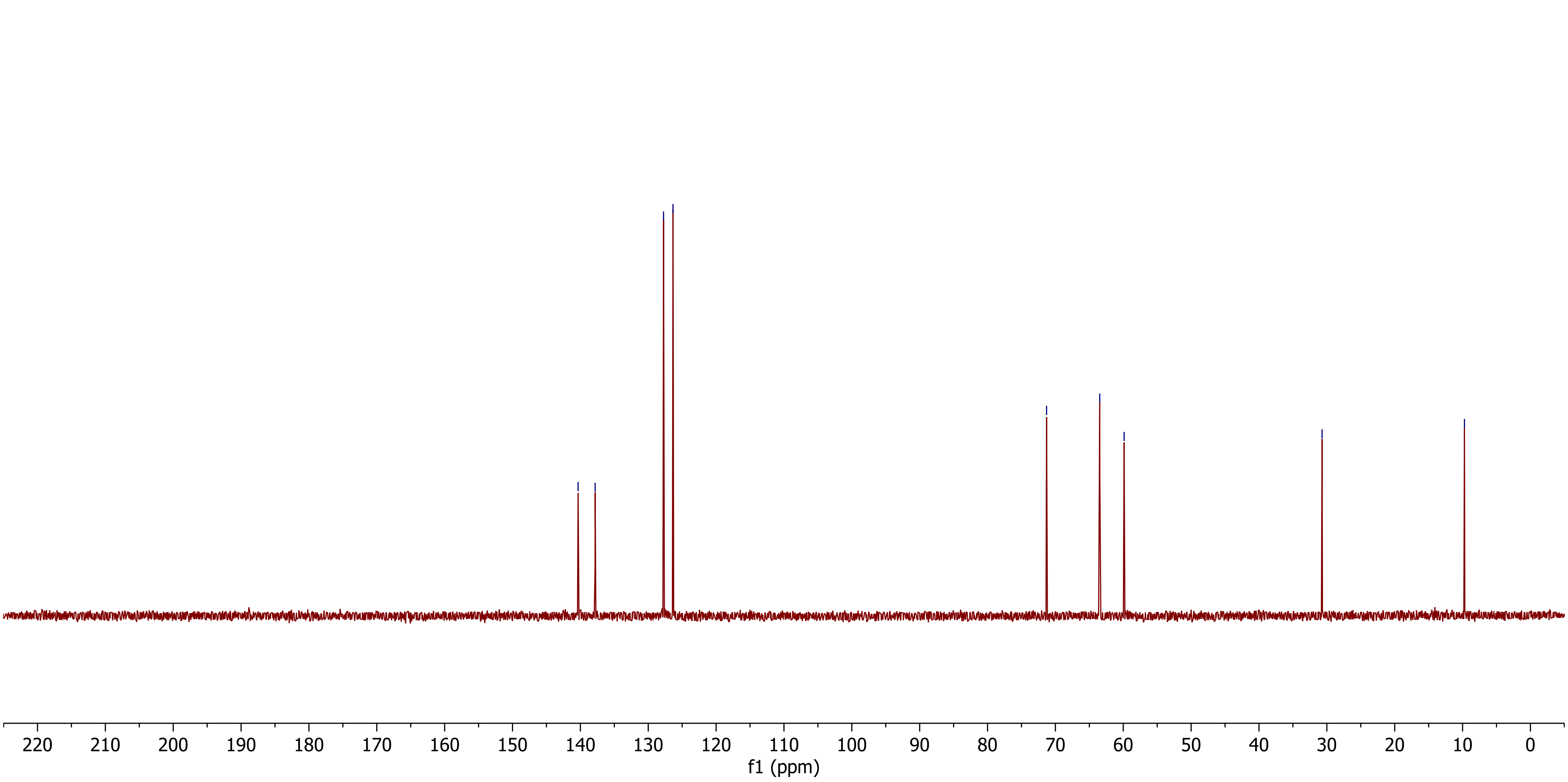

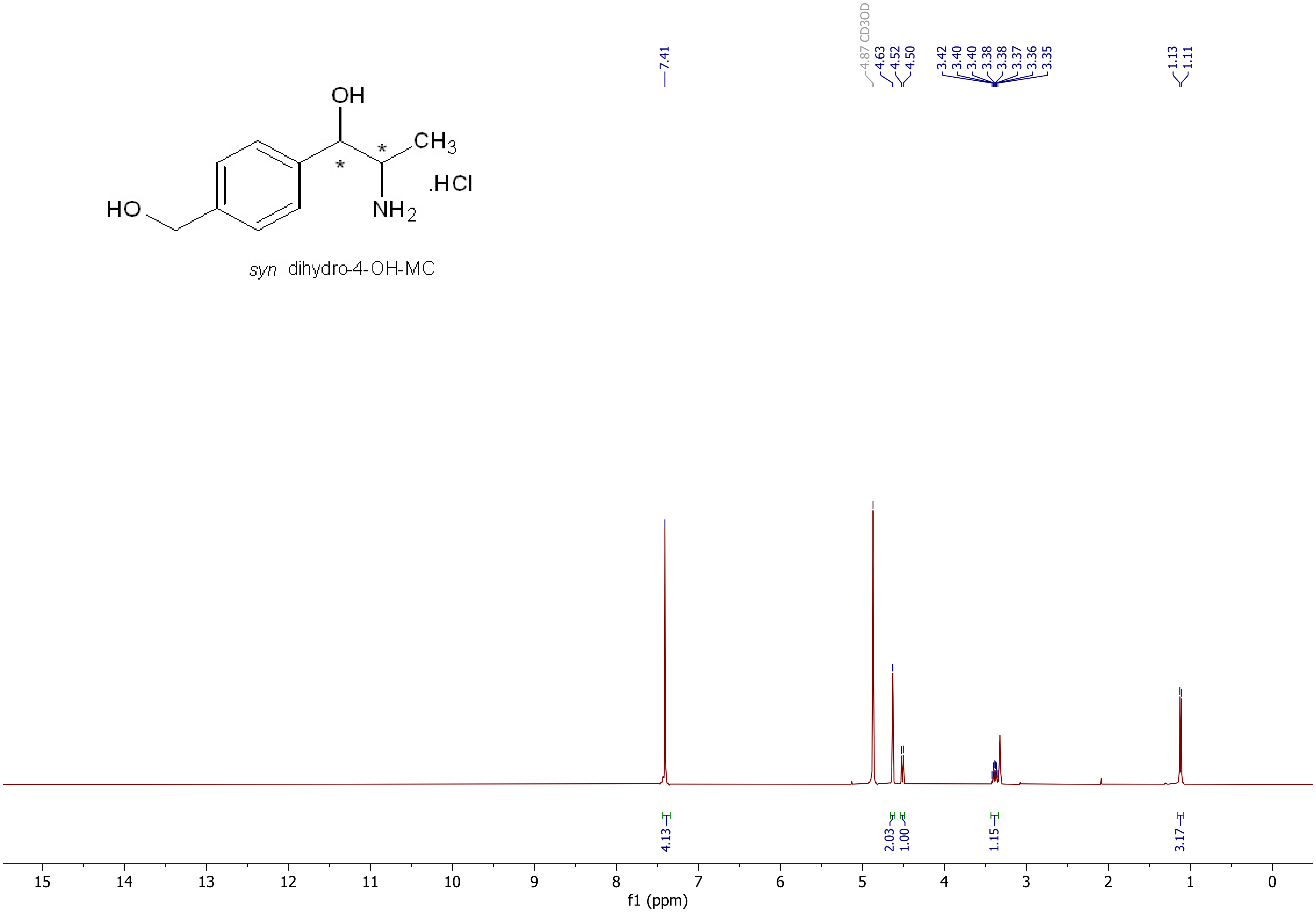
OH
CH3
.HCl
HO
NH2
syn dihydro-4-OH-MC
7.41
4.87 CD3OD
4.63
4.52
4.50
3.42
3.40
3.40
3.38
3.38
3.37
3.36
3.35
1.13
1.11
4.13
2.03
1.00
1.15
3.17
15
14
13
12
11
10
9
8
7
6
5
4
3
2
1
0
f1 (ppm)

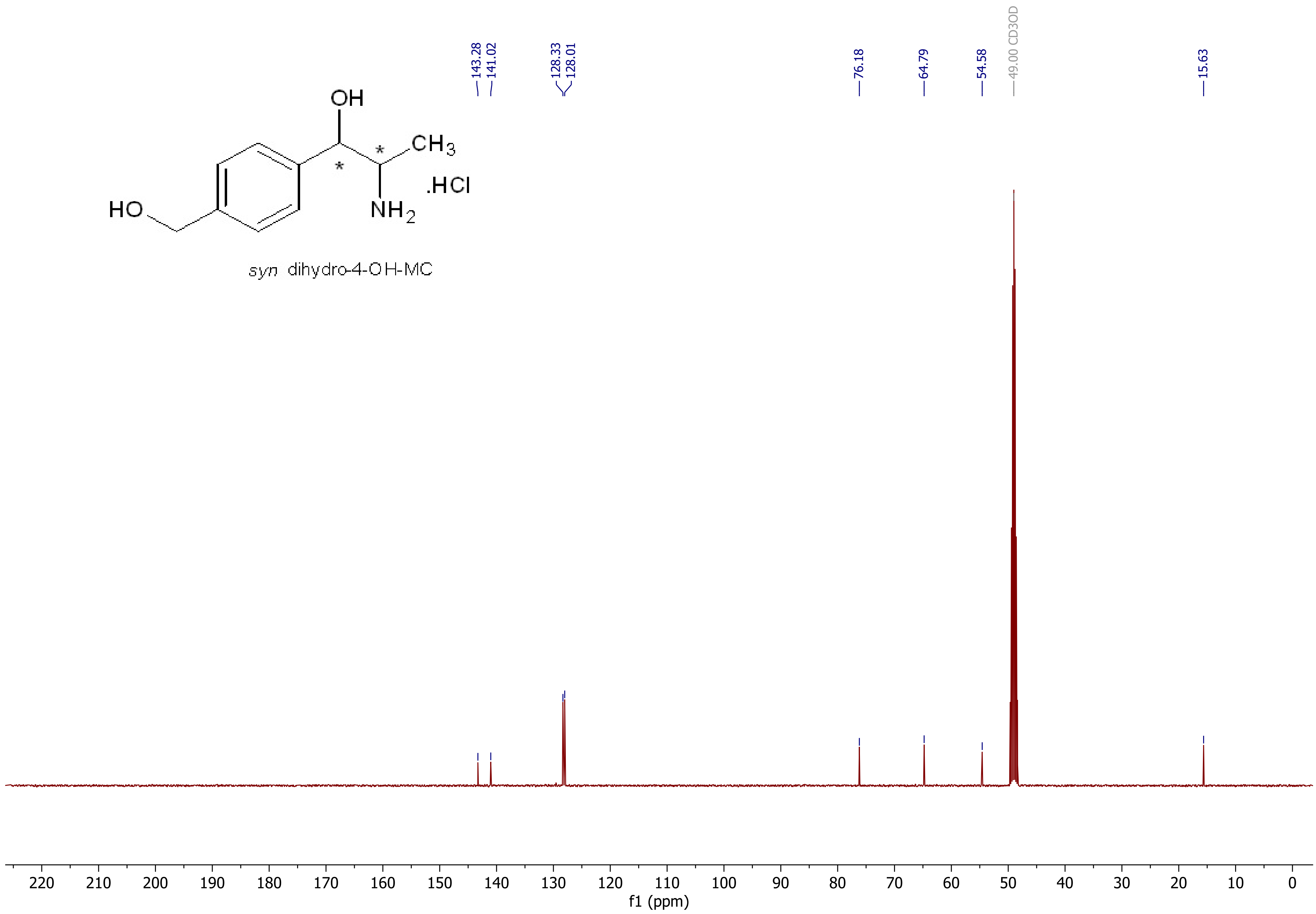

OH
CH3
.HCl
HO
NH2
syn dihydro-4-OH-MC
143.28
141.02
128.33
128.01
76.18
64.79
54.58
49.00 CD3OD
15.63
220 210 200 190 180 170 160 150 140 130 120 110 100 90 80 70 60 50 40 30 20 10 0
f1 (ppm)

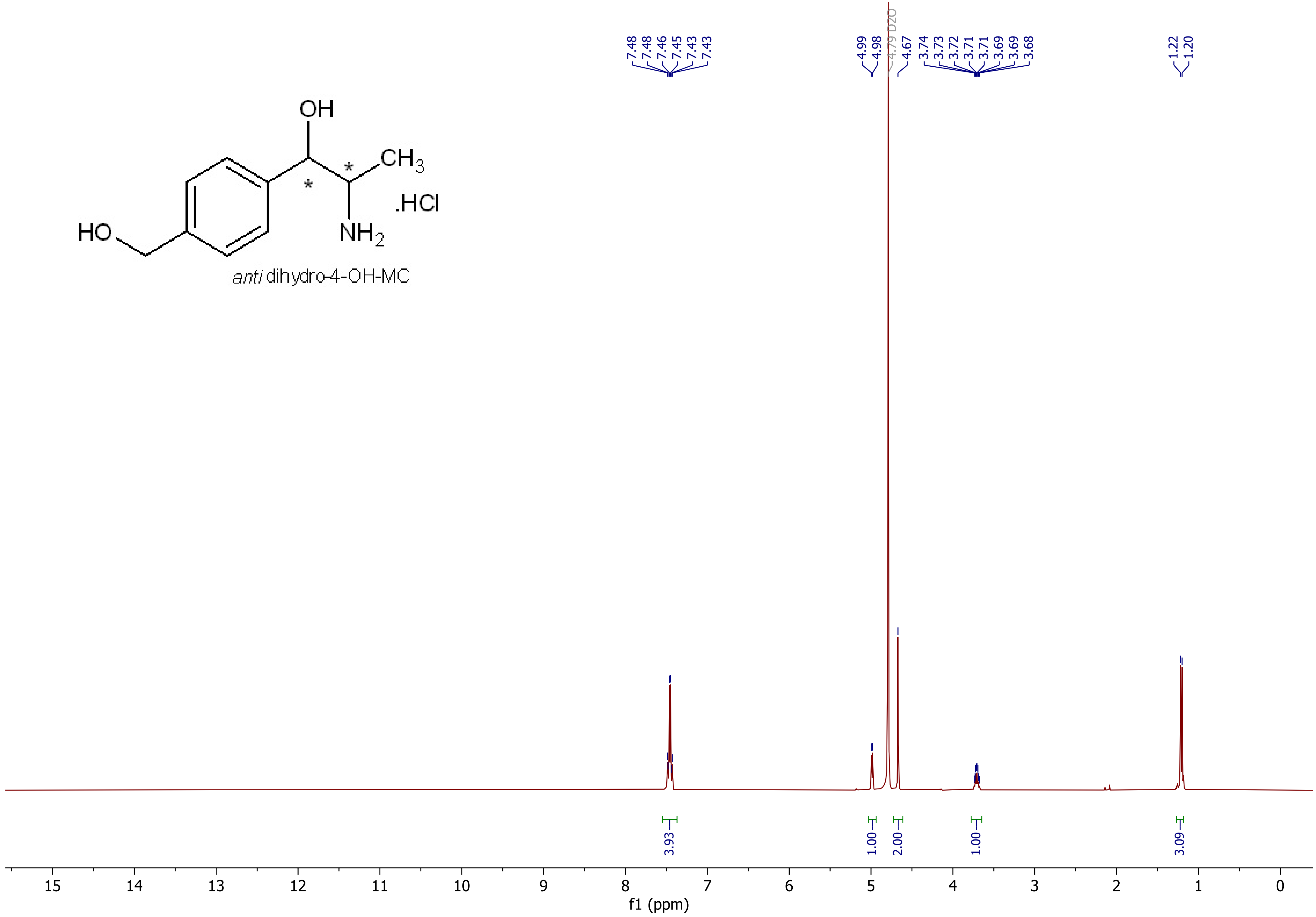

7.48
7.48
7.46
7.45
7.43
7.43
4.99
4.98
4.79 D2O
4.67
3.74
3.73
3.72
3.71
3.71
3.69
3.69
3.68
1.22
1.20
OH
CH3
.HCl
HO
NH2
anti dihydro-4-OH-MC
3.93
1.00
2.00
1.00
3.09
15
14
13
12
11
10
9
8
7
6
5
4
3
2
1
0
f1 (ppm)

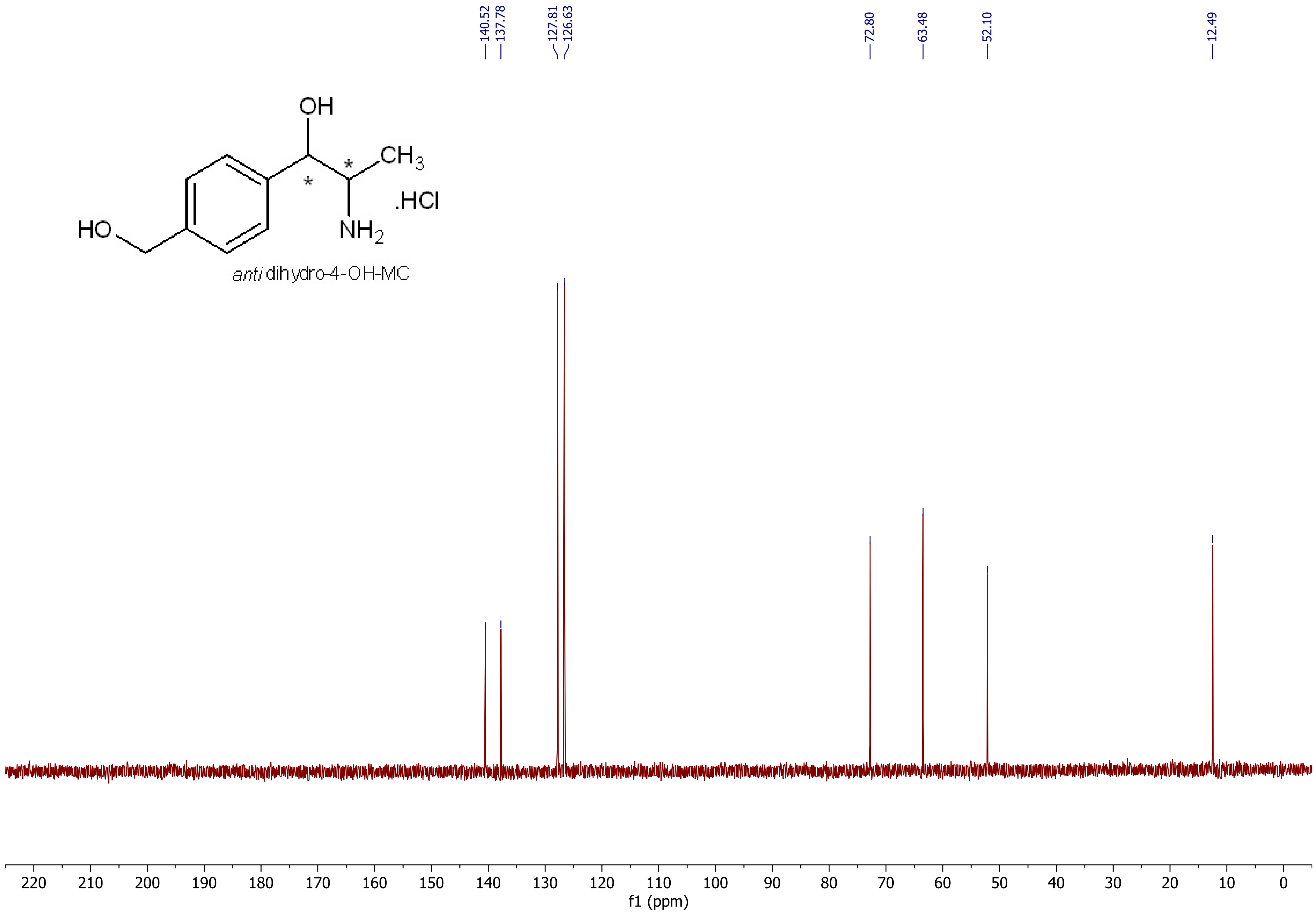
140.52
137.78
127.81
126.63
72.80
63.48
52.10
12.49
OH
CH3
.HCl
HO
NH2
anti dihydro-4-OH-MC
220 210 200 190 180 170 160 150 140 130 120 110 100 90 80 70 60 50 40 30 20 10 0
f1 (ppm)

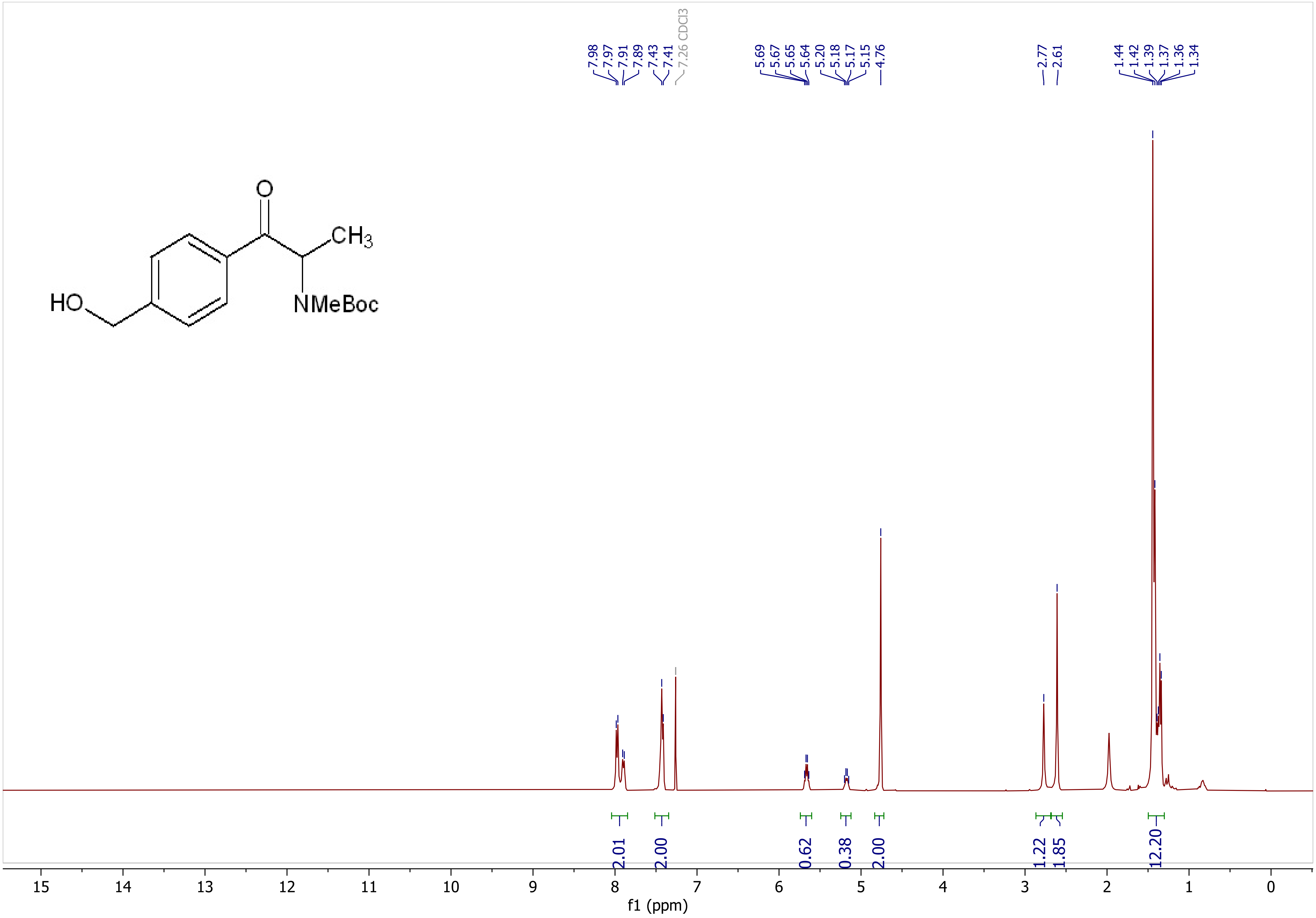

HO
O
CH3
NMeBoc
7.98
7.97
7.91
7.89
7.43
7.41
7.26 CDCl3
5.69
5.67
5.65
5.64
5.20
5.18
5.17
5.15
4.76
2.77
2.61
1.44
1.42
1.39
1.37
1.36
1.34
2.01
2.00
0.62
0.38
2.00
1.22
1.85
12.20
15
14
13
12
11
10
9
8
7
6
5
4
3
2
1
0
f1 (ppm)

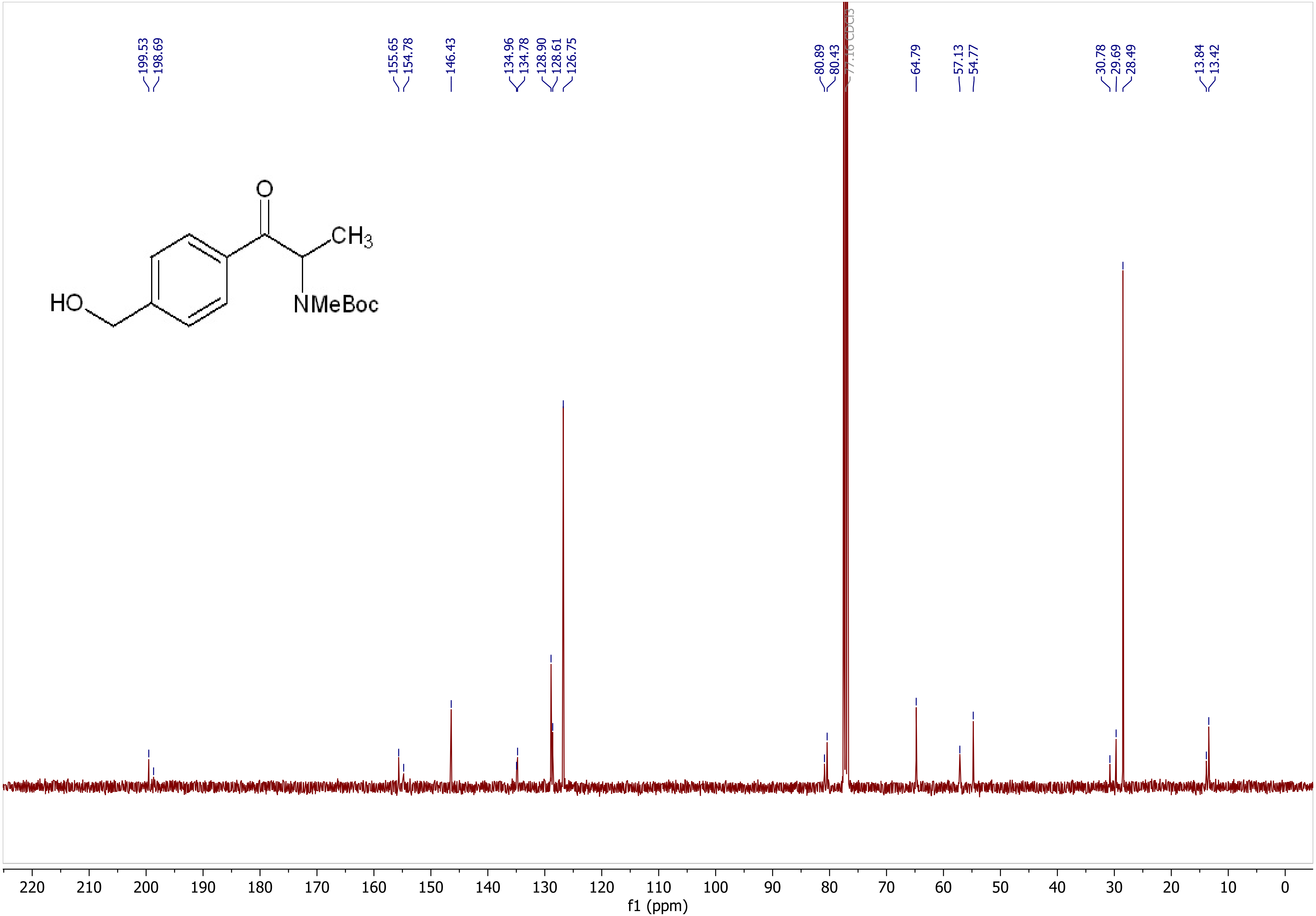

199.53
198.69
155.65
154.78
146.43
134.96
134.78
128.90
128.61
126.75
80.89
80.43
77.16 CDCl3
64.79
57.13
54.77
30.78
29.69
28.49
13.84
13.42
O
CH3
HO
NMeBoc
220 210 200 190 180 170 160 150 140 130 120 110 100 90 80 70 60 50 40 30 20 10 0
f1 (ppm)

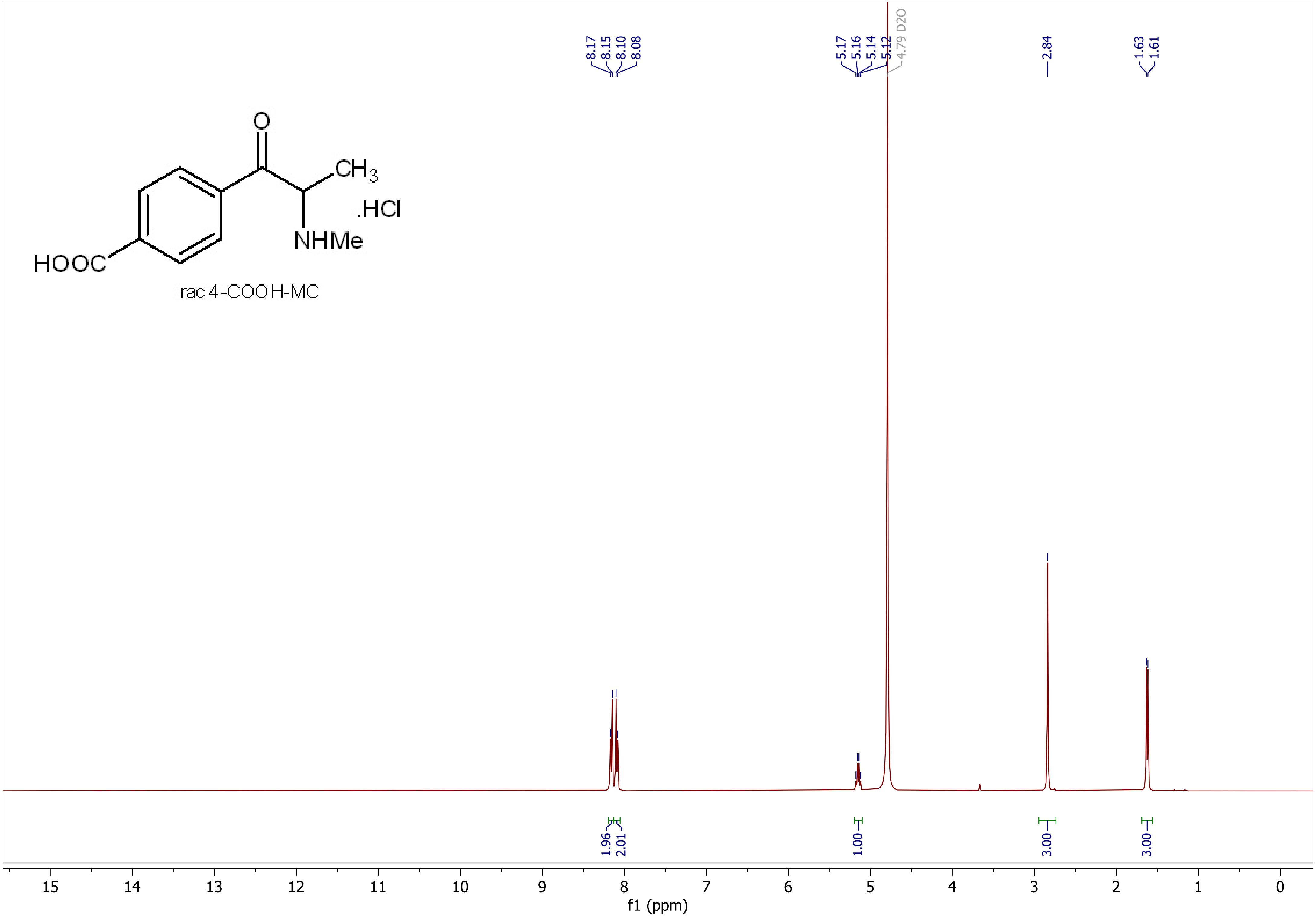

O
CH3
.HCl
NHMe
HOOC
rac 4-COOH-MC
8.17
8.15
8.10
8.08
5.17
5.16
5.14
5.12
4.79 D2O
2.84
1.63
1.61
1.96
2.01
1.00
3.00
3.00
15 14 13 12 11 10 9 8 7 6 5 4 3 2 1 0
f1 (ppm)

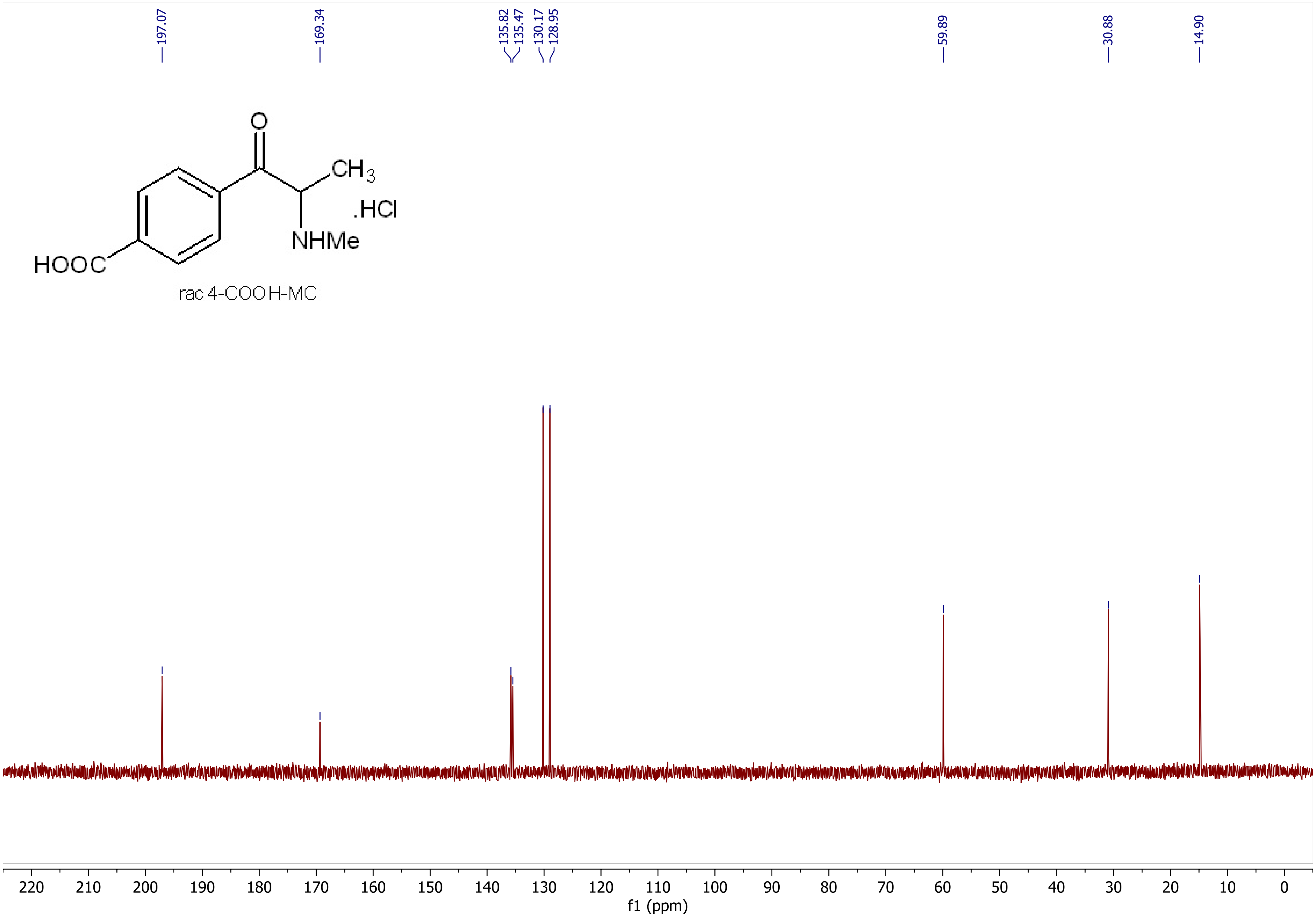

197.07
169.34
135.82
135.47
130.17
128.95
59.89
30.88
14.90
O
CH3
.HCl
NHMe
HOOC
rac 4-COOH-MC
220 210 200 190 180 170 160 150 140 130 120 110 100 90 80 70 60 50 40 30 20 10 0
f1 (ppm)

## Urine analysis

Remark: The experimental set-up of this section is located at Department of Chemical Analytics, Seibersdorf Labor, GmbH.

### Technical parameters

The samples for urine analysis were analyzed using a CTC HTS PAL autosampler (CTC Analytics, Zwingen, Switzerland) and a Thermo Surveyor LC system (Thermo, Austin, TX, USA) interfaced to a TSQ Quantum Discovery Max triple quadrupole mass spectrometer (Thermo, Austin, TX, USA). A C18 security guard cartridge (4 × 2mm) (Phenomenex, Torrance, CA, USA) was used for sample clean-up and the analytical HPLC column was a Kinetex 1.7 µm C18 100 Å 150 x 2.1 mm (Phenomenex, Torrance, CA, USA) and Cortecs T3, 2.7 µm C18 120 Å 100 x 2.1 mm (Waters, Milford, MA, USA).

Column selection was performed by a Maylab Mistraswitch column selector (6 column selection system) (Maylab Analytical Instruments, Vienna, Austria). The solvents were 0.2% formic acid in water (A) and acetonitrile (B). The temperature of the analytical column was maintained at 40 °C. The mass spectrometer was equipped with heated electrospray ionization (ESI) source and was operated in positive ionization mode with a spray voltage set at 4500 V. The capillary temperature was adjusted to 320 °C. The sheath and auxiliary gas (nitrogen) flow rate was 25 and 10 arbitrary units, respectively. The system was operated in selected ion monitoring (srm) mode with argon as the collision gas at a pressure of 1.5 mTorr.

### Achiral separation

Method development

Stem solutions were prepared by weighing the reference substances in volumetric flasks and filling them up with MeOH (99.9%; HPLC grade), creating average concentration of 1 mg/mL. Then dilution solutions were prepared as follows: 10 µL of stem solution was filled up to 5 mL MeOH (99.9%, HPLC grade), creating dilution 1:1000. Samples were prepared from dilution solutions by taking out 25 µL and mixing it together with 25 µL of ISP, 50 µL ISS and filled up to 1 mL with Mili-Q water. The final sample solvent was water/MeOH 9/1.

**COLUMN A**: Kinetex® 2.6 µm Biphenyl 100 Å, LC column 50 x 2.1 mm, Ea, Phenomenex, Solid support: core-shell Silica; Stationary phase: Biphenyl with TMS endcapping (for 4-MMC, 4-OH-MMC, 4-MC, dihydro-4-MC, dihydro-4-MMC)

**COLUMN B**: Cortecs T3, 2.7 µm C18 120 Å, 100 x 2.1 mm, Waters (for 4-COOH-MC, dihydro-4-OH-MC, dihydro-4-OH-MMC, 4-OH-MC)

| ACHIRAL | | | |
|---|---|---|---|
| METHOD -M2 gradient | | | |
| | time | %B (MeOH+0.1% FA) | %A ($H_2O$+0.2% FA) |
| 1 | 0 | 0 | 100 |
| 2 | 2 | 0 | 100 |
| 3 | 7 | 50 | 50 |
| 4 | 10 | 100 | 0 |
| 5 | 13 | 100 | 0 |
| 6 | 13,1 | 0 | 100 |
| 7 | 16 | 0 | 100 |

Sample preparation

SM-MEPH stem solution: 20 µL of every stem solution (4-OH-MMC, 4-OH-MC, 4-MMC, 4-MC, dihydro-4-MC, dihydro-4-MMC, dihydro-4-OH-MC, dihydro-4-OH-MMC, 4-COOH-MC) filled up to 10 mL with MeOH.

**Blank**: (50 µL blind urine, 25 µL ISP, 50 µL ISS, filled up to 1mL with MQ-water)

**SP01** (spike 1): (50 µL blind urine, 20 µL SM-MEPH, 25 µL ISP, 50 µL ISS, filled up to 1 mL with MQ-water)

**SP02** (spike 2): (50 µL blind urine, 50 µL SM-MEPH, 25 µL ISP, 50 µL ISS, filled up to 1 mL with MQ-water)

**FD 828 (urine sample)**: (50 µL, 25 µL ISP, 50 µL ISS, filled up to 1mL MQ-with water)

Enzymatic workup

To all the above-mentioned solutions phosphate buffer (1 mL, 0.8M, pH 7), β-glucuronidase/arylsulfatase (25 µL) and β-glucuronidase (25 µL) were added. The solution was heated at 50 °C during 1 h upon which EtOAc (7 mL) was added. The solution was put into shaker for 10 min, following centrifugation for 5 min. The organic phase was separated and evaporated into dryness in stream of pressurized nitrogen. The residue was taken up in 150 µL MQ-water/MeOH 9/1 + 0.4% formic acid, heated at 50 ° for 5 min and subjected to LC-MS analysis.

**Code legend:**

| | |
|---|---|
| DC 208/210 | syn-dihydro-4MC |
| DC 133/137 | anti-dihydro-4MC |
| DC 287/276 | syn-dihydro-4-OH-MC |
| DC 293/294 | anti-dihydro-4-OH-MC |
| DC 253/254 | syn-dihydro-4-OH-MMC |
| DC 278/277 | anti-dihydro-4-OH-MMC |
| DC 242 | rac-COOH-4MC |
| DOM 14/32 | 4-OH-MC |
| LAU 506 | 4-OH-MMC |
| LAU 511 | 4-MC |
| MEN 29/30 | syn-dihydro-4MMC |
| DC 146/147 | anti-dihydro-4MMC |

# Mephedrone Metabolites Detection in Urine



| | | | | | |
|---|---|---|---|---|---|
| File | F_MEPH_biphen_FD828A_A_01 | Name: | 250 µl Sample derivatised | Acqu. Date: | 12/18/17 08:43:08 |
| Vial: | CStk1-01:42 | Path | M:\C4LC156\Daten\171216kh | Calibration File: | N/A |
| Dil. Factor: | 4.00 | Instr. Method: | D:\Forschung\Daniela\MEPH Metaboliten_Biphenyl | Proces. Method: | N/A |

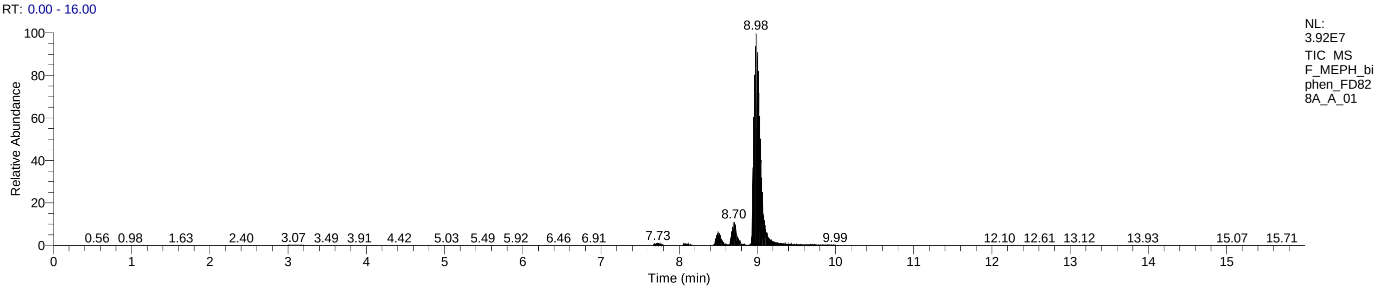


## Mephedron S/R

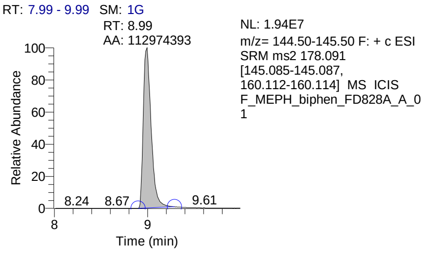


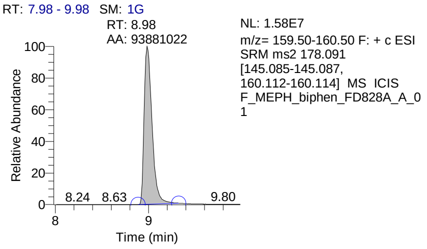


## LAU 506

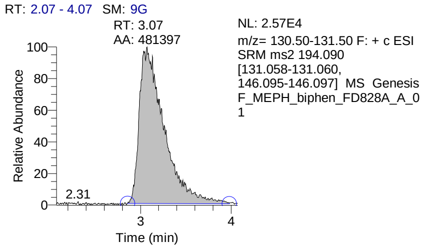


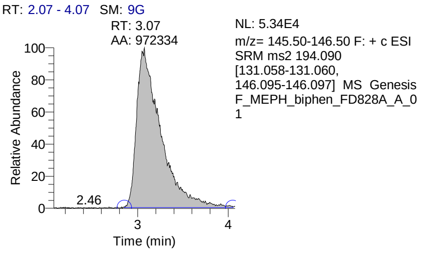


## LAU 511

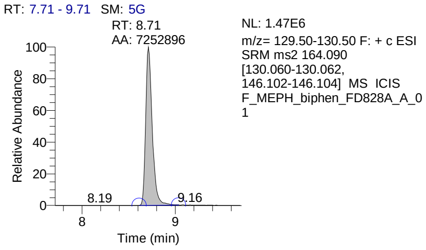


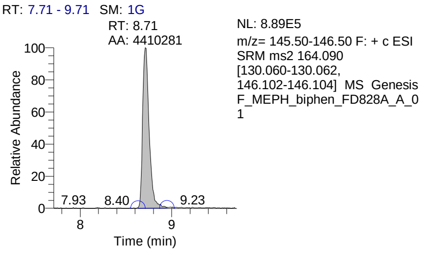

# Mephedrone Metabolites Detection in Urine



| | | | | | |
|---|---|---|---|---|---|
| File | F_MEPH_biphen_FD828A_A_01 | Name: | 250 µl Sample derivatised | Acqu. Date: | 12/18/17 08:43:08 |
| Vial: | CStk1-01:42 | Path | M:\C4LC156\Daten\171216kh | Calibration File: | N/A |
| Dil. Factor: | 4.00 | Instr. Method: | D:\Forschung\Daniela\MEPH Metaboliten_Biphenyl | Proces. Method: | N/A |

## DC 146/147

RT: 7.50 - 9.50 SM: 5G

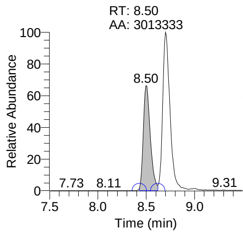


NL: 8.93E5
m/z= 90.50-91.50 F: + c ESI SRM ms2 180.103 [91.009-91.011, 115.027-115.029] MS ICIS F_MEPH_biphen_FD828A_A_01

RT: 7.49 - 9.49 SM: 1G

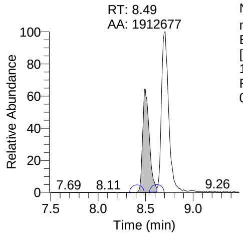


NL: 5.90E5
m/z= 114.50-115.50 F: + c ESI SRM ms2 180.103 [91.009-91.011, 115.027-115.029] MS ICIS F_MEPH_biphen_FD828A_A_01

RT: 7.50 - 9.50 SM: 5G

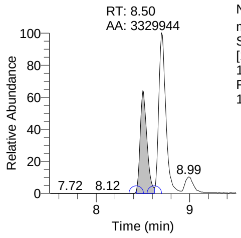


NL: 1.02E6
m/z= 146.50-147.50 F: + c ESI SRM ms2 180.104 [147.101-147.103, 162.130-162.132] MS ICIS F_MEPH_biphen_FD828A_A_01

RT: 7.50 - 9.50 SM: 1G

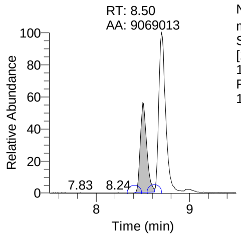


NL: 3.34E6
m/z= 161.50-162.50 F: + c ESI SRM ms2 180.104 [147.101-147.103, 162.130-162.132] MS ICIS F_MEPH_biphen_FD828A_A_01

## MEN 29/30

RT: 7.70 - 9.70 SM: 5G

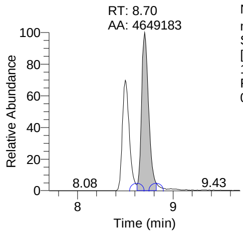


NL: 8.95E5
m/z= 90.50-91.50 F: + c ESI SRM ms2 180.111 [91.019-91.021, 147.102-147.104] MS ICIS F_MEPH_biphen_FD828A_A_01

RT: 7.70 - 9.70 SM: 1G

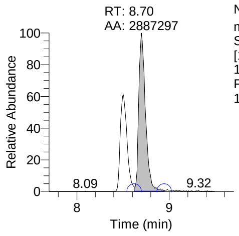


NL: 5.74E5
m/z= 114.50-115.50 F: + c ESI SRM ms2 180.102 [115.032-115.034, 162.134-162.136] MS ICIS F_MEPH_biphen_FD828A_A_01

RT: 7.70 - 9.70 SM: 5G

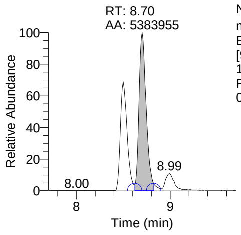


NL: 1.03E6
m/z= 146.50-147.50 F: + c ESI SRM ms2 180.111 [91.019-91.021, 147.102-147.104] MS ICIS F_MEPH_biphen_FD828A_A_01

RT: 7.70 - 9.70 SM: 1G

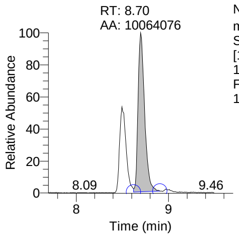


NL: 2.06E6
m/z= 161.50-162.50 F: + c ESI SRM ms2 180.102 [115.032-115.034, 162.134-162.136] MS ICIS F_MEPH_biphen_FD828A_A_01

## DC 133/137

RT: 6.73 - 8.73 SM: 1G

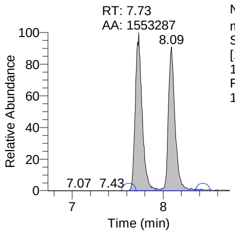


NL: 1.56E5
m/z= 114.50-115.50 F: + c ESI SRM ms2 166.109 [115.047-115.049, 131.080-131.082] MS Genesis F_MEPH_biphen_FD828A_A_01

RT: 6.72 - 8.72 SM: 5G

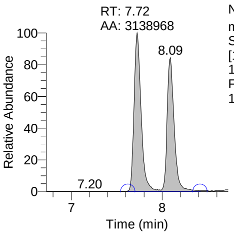


NL: 3.13E5
m/z= 130.50-131.50 F: + c ESI SRM ms2 166.109 [115.047-115.049, 131.080-131.082] MS Genesis F_MEPH_biphen_FD828A_A_01

# Mephedrone Metabolites Detection in Urine



| | | | | | |
|---|---|---|---|---|---|
| File | F_MEPH_biphen_FD828_02 | Name: | 100 µl Sample | Acqu. Date: | 08/02/18 06:47:53 |
| Vial: | CStk1-01:44 | Path | M:\C4LC156\Daten\180802kh | Calibration File: | N/A |
| Dil. Factor: | 1.00 | Instr. Method: | D:\Forschung\Daniela\2018\MEPH Metaboliten_C18 | Proces. Method: | N/A |

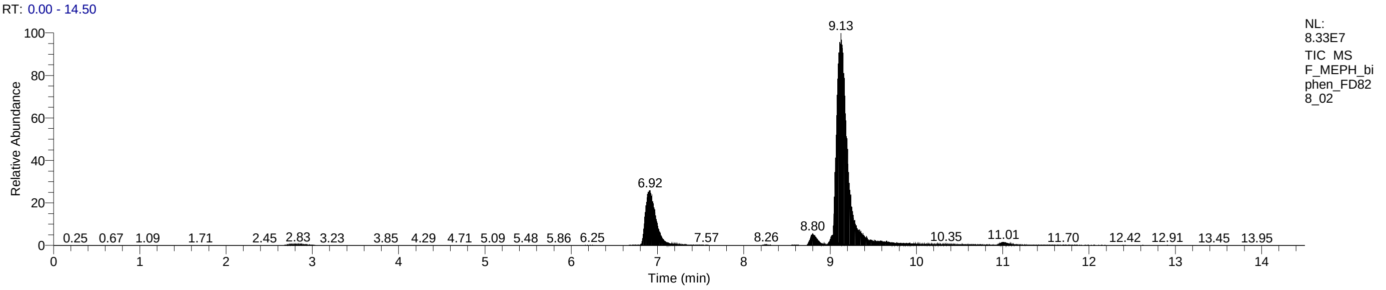


## Salbutamol_IS_D9

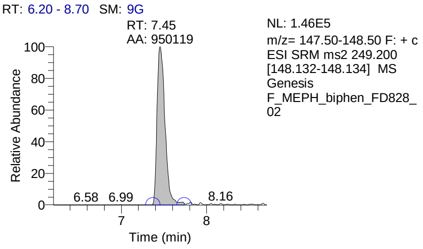


## Methyltestosteron_IS

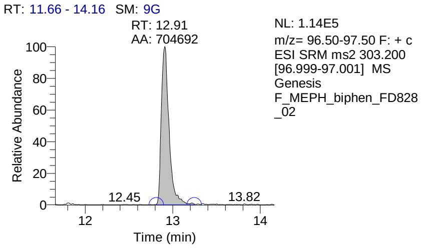


## Mephedron_IS_D3

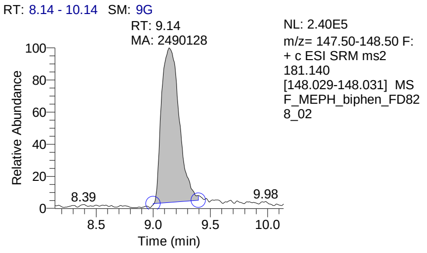


## DC 208/210

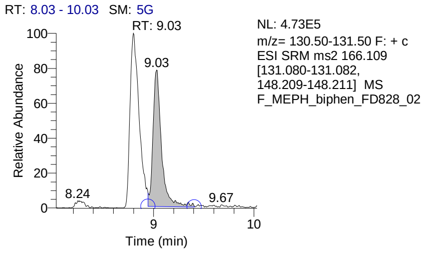


## DC 133/137

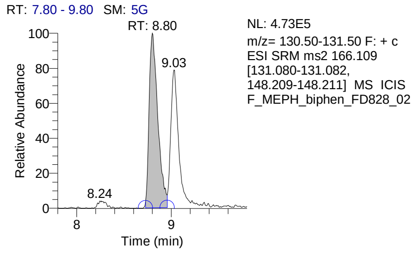


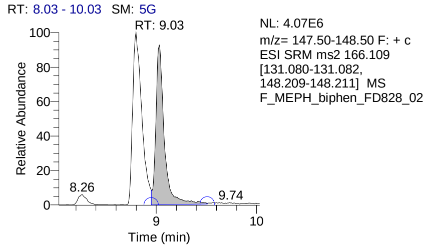


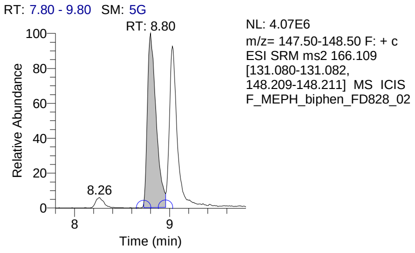

# Mephedrone Metabolites Detection in Urine



| | | | | | |
|---|---|---|---|---|---|
| File | F_MEPH_biphen_FD828_02 | Name: | 100 µl Sample | Acqu. Date: | 08/02/18 06:47:53 |
| Vial: | CStk1-01:44 | Path | M:\C4LC156\Daten\180802kh | Calibration File: | N/A |
| Dil. Factor: | 1.00 | Instr. Method: | D:\Forschung\Daniela\2018\MEPH Metaboliten_C18 | Proces. Method: | N/A |

## DC 287/276

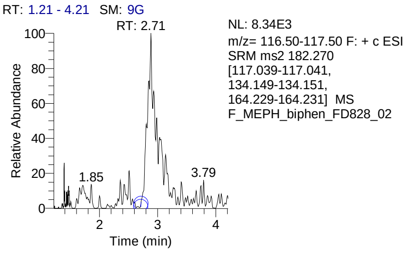


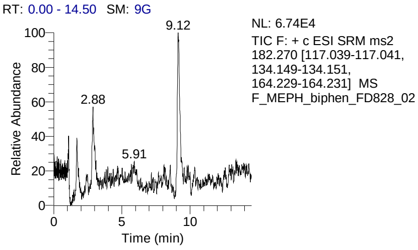


## DC 294/293

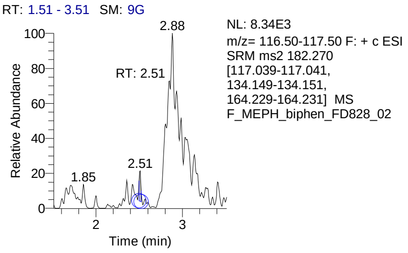


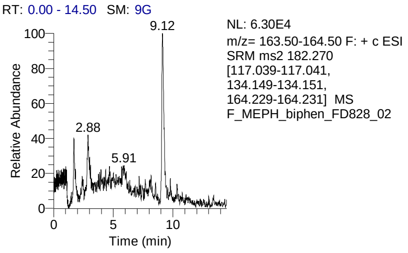


## DC 253/254

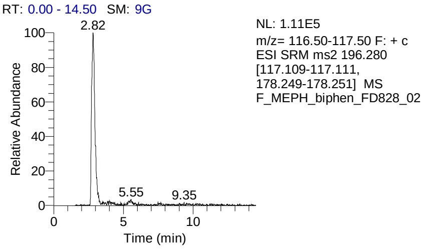


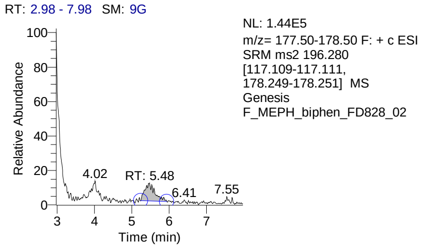


## DC 278/277

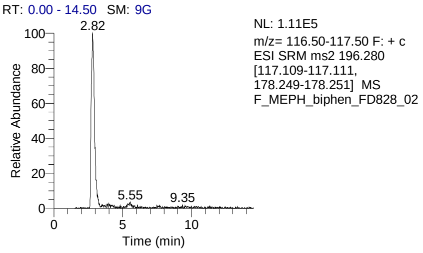


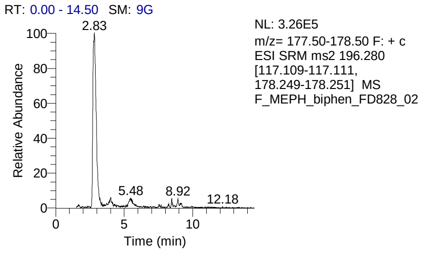


## DC 242 (rac)

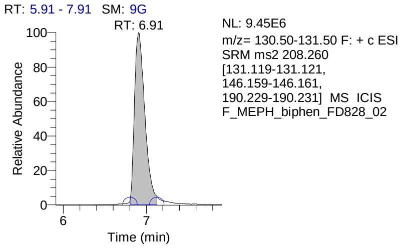


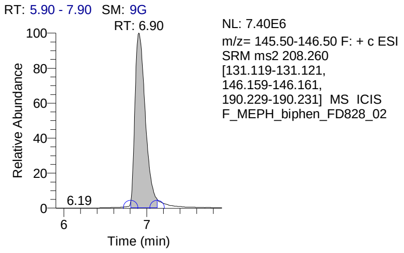

Supplement: Supplementary file 1 [file datasheet1.pdf]
